# Process Simulation and Optimization on Ionic Liquids

**DOI:** 10.1021/acs.chemrev.3c00512

**Published:** 2024-02-06

**Authors:** Jose Palomar, Jesús Lemus, Pablo Navarro, Cristian Moya, Rubén Santiago, Daniel Hospital-Benito, Elisa Hernández

**Affiliations:** † Chemical Engineering Department, Autonomous University of Madrid, Calle Tomás y Valiente 7, 28049 Madrid, Spain; ‡ Departamento de Tecnología Química, Energética y Mecánica, Universidad Rey Juan Carlos, 28933 Madrid, Spain; § Departamento de Ingeniería Eléctrica, Electrónica, Control, Telemática y Química aplicada a la Ingeniería, ETS de Ingenieros Industriales, Universidad Nacional de Educación a Distancia (UNED), 28040 Madrid, Spain

## Abstract

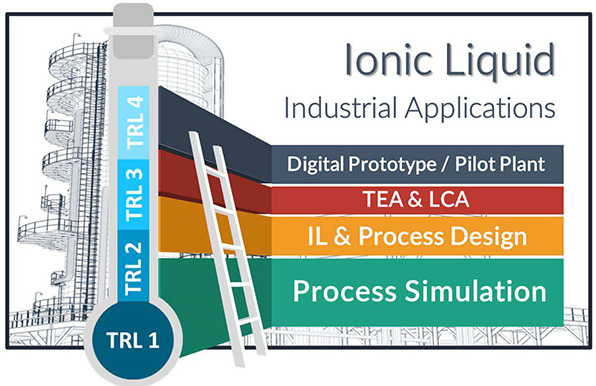

Ionic liquids (ILs) are promising alternative compounds that enable the development of technologies based on their unique properties as solvents or catalysts. These technologies require integrated product and process designs to select ILs with optimal process performances at an industrial scale to promote cost-effective and sustainable technologies. The digital era and multiscale research methodologies have changed the paradigm from experiment-oriented to hybrid experimental–computational developments guided by process engineering. This Review summarizes the relevant contributions (>300 research papers) of process simulations to advance IL-based technology developments by guiding experimental research efforts and enhancing industrial transferability. Robust simulation methodologies, mostly based on predictive COSMO-SAC/RS and UNIFAC models in Aspen Plus software, were applied to analyze key IL applications: physical and chemical CO_2_ capture, CO_2_ conversion, gas separation, liquid–liquid extraction, extractive distillation, refrigeration cycles, and biorefinery. The contributions concern the IL selection criteria, operational unit design, equipment sizing, technoeconomic and environmental analyses, and process optimization to promote the competitiveness of the proposed IL-based technologies. Process simulation revealed that multiscale research strategies enable advancement in the technological development of IL applications by focusing research efforts to overcome the limitations and exploit the excellent properties of ILs.

## 1 Introduction

Owing to their unique properties, ionic liquids (ILs) emerged several years ago as promising potential replacements for conventional solvents or catalysts in industrial chemical processes^1,2^ and changed the paradigm by enabling the use of solvents that simultaneously exhibited liquid and nonvolatile characteristics.^3,4^ The other highlighted property that made ILs unique was their high degree of property tunability, which was achieved by changing the structural features of anions, cations, and/or their substituents.^5^ This tunability enabled ILs to change from hydrophobic to hydrophilic or even from high to moderate viscosity to exhibit a wide range of properties in a series of ILs.^6^ Consequently, the fundamental research in this field has commonly focused on the selection of a cation–anion combination to obtain proper ILs for specific applications.^7^ Simultaneously, these possibilities ushered in a new era in the integrated design of chemical products (solvents or catalysts) and processes to obtain new IL-based technologies^8,9^ that were more efficient, cost-effective, and sustainable than the previous industrial benchmarks.^10−12^ For the scientific community, particularly for chemical engineers working in the IL-research field,^13,14^ this implied both a clear advantage and a limitation because although the range of potential industrial applications (gas separation,^15−17^ liquid–liquid extraction,^18−20^ advanced distillation,^21^ catalysis,^22,23^ carbon capture^24,25^ and utilization,^26,27^ biomass processing,^28,29^ etc.) is vast and a huge number of cations and anions is available for potentially enabling the synthesis of millions of ILs,^30^ the assurance for achieving the desired criteria was relatively low, even when performing long and costly experimental screenings and pilot plant tests.^31^

Process simulation—a professional computational tool decisively involved in the conceptual design and basic engineering stages for developing chemical processes—^32^ has substantially contributed to the main challenges of IL research. Thus, steady-state process simulations have supported the computational synthesis and analysis of new processes comprising the use of ILs, thereby enabling the mass–energy balance, equipment design, and evaluation of the energy requirements and economics of new processes at the industrial scale.^33−35^ In addition, process simulations have enabled the scientific community to perform the difficult task of the selection of ILs with favorable solvent/catalyst behaviors by combining the specifications of a concrete operation with the most relevant well-known IL constraints, namely, the melting point, thermal stability, viscosity, and price.^36−39^ For example, although ILs are thermally stable, the design of the IL regeneration operation was drastically conditioned by the temperature range allowed for each IL, which ultimately determined the IL selection or required an operating vacuum.^33^ Regarding viscosity, the mass transfer limited some IL-based operations, such as most gas absorptions near room temperature;^35,40^ however, in other applications (e.g., distillation, reactions, and liquid–liquid extraction),^41−43^ this limitation may not have controlled the operation. The question that emerges here is how to know what an application demands from an IL? Process simulation definitively contributes to state the key IL properties determining the process performance at industrial scale, guiding the IL product design. Thus, any separation or reaction involving ILs required solvent recovery and reuse for economics and sustainability. The limitations of the regeneration of a mixture of ILs and some solutes could only be evaluated based on the required vacuum, energy consumption, and/or operating costs, for which process simulation required mass and energy balances through the use of simple industrial (flash distillation units)^44^ or complex (distillation or stripping columns) devices.^45^ Therefore, process simulation has enabled the conformity of multifactorial and consistent sets of criteria (thermodynamic, kinetic, technical, energetic, environmental, and economic) for selecting ILs with optimized properties for improving the process performance of specific industrial applications.^36−38,46−48^

The scientific community, on the other hand, required suitable prospective tools to evaluate not only the suitability of an IL-based process but also the feasibility of IL-based technologies, and process simulation offered an ideal solution for narrowing the range of potential ILs.^9,13−15,17,25^ Although experimental efforts were essential to expand knowledge boundaries, laboratory data were ineffective for making decisions on the process performance; for example, although ILs could be selected for liquid–liquid extraction to increase the solubility or improve the selectivity of interactions, measured extractive properties alone were not criteria for anticipating improved process performance for a specified productivity and product quality. In this respect, process simulations have also been used as a computational tool to guide fundamental experimental studies in the research of IL applications.^39,49,50^

Nevertheless, the use of commercial process simulators for modeling IL industrial processes was a difficult task comprising several straightforward methodological questions.^51^ First, ILs were scarcely included in the databases of commercial process simulators. Second, the lack of available experimental information on IL-based systems has limited the application of regressive thermodynamic models that were traditionally used in process simulation. Third, the development of cost-effective and sustainable IL-based industrial applications required alternative evaluations of numerous systems (solvents, reaction media, etc.) and processes, which were severely conditioned by the huge number of available cation–anion combinations. An affordable solution, provided by researchers in the field, was the combination of predictive methods to estimate IL-system properties using process simulation tools. The multiscale methodology concept emerged as a flexible and multilevel strategy that combined computer-aided product (IL) design and (IL-based process design) simulations to improve the IL features and, thus, key performance indicators (KPIs) within an experimentally validated model that linked the molecular and process scales. This solution was widely addressed by several chemical engineering research groups in many different IL application fields using a wide variety of computational strategies involving differently formulated predictive thermodynamic models, such as the predictive COSMO-SAC/RS^7,33,35−78^ and UNIFAC^79−108^ methods, and those based on equation of state, such as PRK^109−113^ and PC-SAFT.^7,52−64,114−116^ Predictive methods combined with process simulations have also enabled the integration of IL product and IL-based process designs to obtain the minimal solvent and energy requirements and process costs. In fact, effective optimization methods have been successfully applied to IL-based processes in complex multiscale approaches obtaining significant cost savings.^117,118,73,119^ Interestingly, process simulation results have been used in life-cycle assessments (LCAs)^14,48,77,120^ by extending the analysis of new IL-based processes to the evaluation of their environmental impacts by emphasizing the role of IL synthesis and process efficiency, compared to those of conventional technologies. The growing fundamental research focused on IL performance at process scale is in line with the current enlightenment of IL market development, being reported 57 implemented IL applications, already commercialized, or developed at pilot plant scale.^121^

State-of-the-art IL process simulations have exponentially increased in the past few years, showing the wider picture for potential applications and complexity in unit operations and processes descriptions, thus motivating this Review. The key IL-applications evaluated using process simulations have involved carbon capture by physical or chemical absorption, carbon utilization, gas purification, separation of aromatic–aliphatic or aqueous mixtures by liquid–liquid and extractive distillation, absorption refrigeration cycles, biomass treatment, etc. The notable contributions of these studies could be advanced as follows: (i) the preliminary computational evaluation of the IL performance as a solvent or catalyst at the industrial scale; (ii) the introduction of several criteria (physicochemical, thermodynamic and kinetic properties, price, thermal stability, melting point, environmental concerns, etc.) for selecting ILs based on the KPI improvement in the IL-based process; and (iii) the feasibility analysis and optimization of the new IL-based process, considering technoeconomic and environmental KPIs, compared to the results obtained using available technologies and conventional solvents. Nowadays, the massive-scale applications of ILs are still limited and the market remind behind the midterm forecasts, owing to different technical and economic reasons.^1,2,122^ However, there are many favorable indicators, as the increasing number of commercialized or pilot plant IL applications, the huge market penetration potential of ILs, the continuously growing number of patents, or the significant price decrease of ILs with scaled-production.^121^ To this respect, the future application of ILs at the industrial scale will be strictly determined by the quality and robustness of advances in Technological Readiness Levels (TRLs). In this sense, process simulations have contributed to the movement from TRL1 to TRL4 in IL-based technological developments. Process modeling should contribute to different digital transitional goals in the field, from the development of digital twin prototypes to the acceleration of technological development to higher TRLs. The application of artificial intelligence for developing models of properties, operations, and systems will be a breakthrough in process simulation applications and will promote disruptive advances in research, development, and innovation in IL-based application fields.

The objectives of this Review are as follows: (i) summarizing the most widely applied and successful strategies and procedures used to perform process simulations and optimize ILs, mainly centered on the use of the commercial process simulator Aspen Plus and predictive thermodynamic models (Sections 2 and 3, respectively); (ii) highlighting the main contributions in the literature on process simulations for advancing the knowledge of the key IL applications (Section 4); and (iii) emphasizing the main limitations and proposing future developments of process simulations to advance the development of IL-based technology.

## 2 Process Simulation Strategy in Research on Ionic Liquids

**Figure 1:**
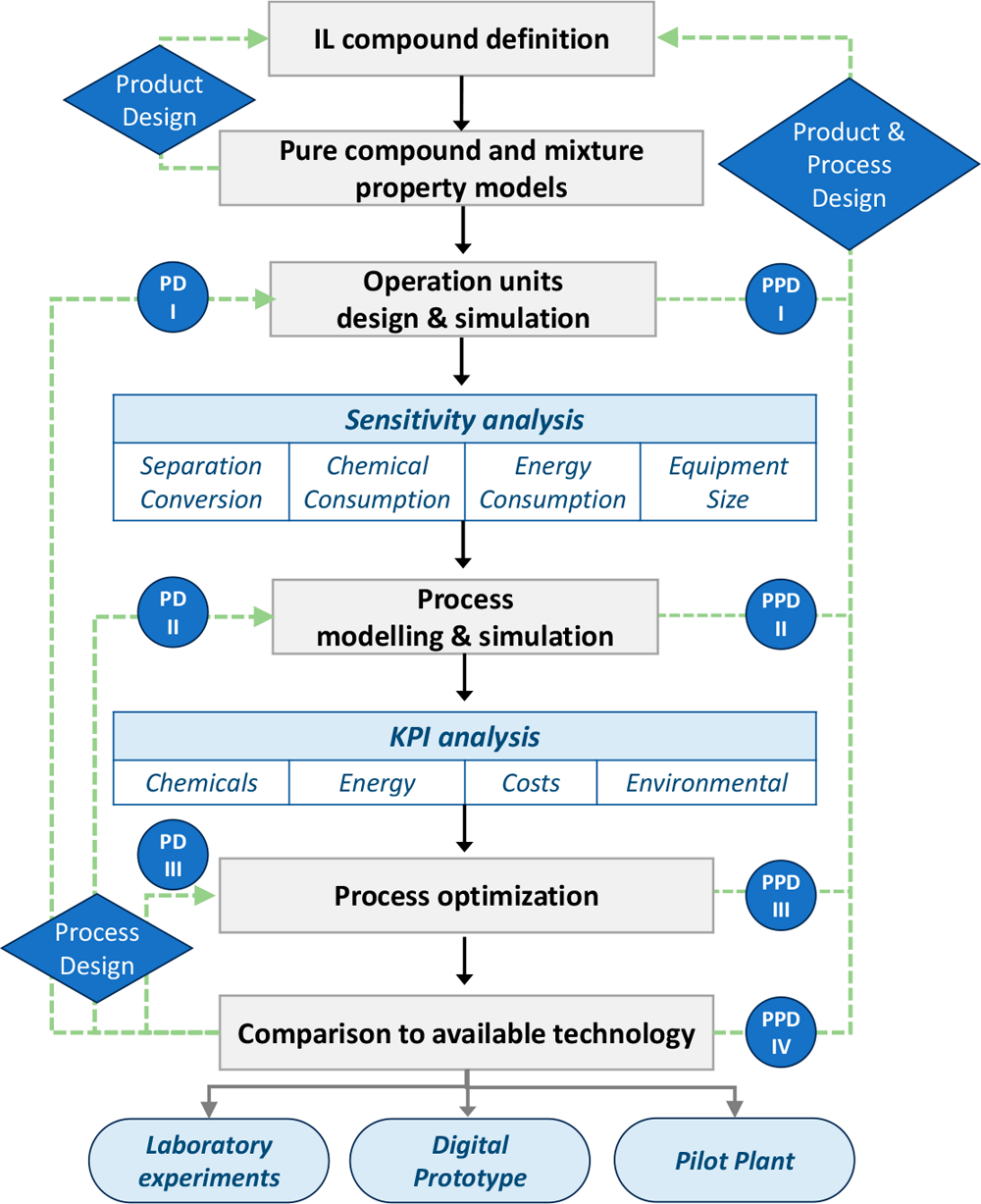
Step-based strategy for applying process simulation in conceptual design, analysis, and development of IL-based applications at process scale.

This section focused on describing the strategies implemented by researchers to apply process simulation for developing IL-based industrial applications. Figure 1 summarizes the successive steps commonly followed in these studies, which nearly correspond to the methodology used to complete the main tasks of conceptual and basic engineering during the development of a chemical process. In the computational activity of engineering projects, the use of commercial process simulators is predominant.

In this respect, most (>90%) process simulation studies on ILs have been performed using the widely professionally applied Aspen Plus or Aspen Hysys process simulators; therefore, the following description mainly considers the computational approach developed using this commercial software (of course, there exists excellent alternative software successfully applied for modeling IL-based process, as gPROMS). Section 3 describes the methodological details and literature related to each stage of the research strategy shown in Figure 1.

### 2.1 IL Compound Definition

In process simulations, the work begins by identifying and defining the system components, which may or may not be included in the pure-component databases of the commercial process simulators. In Aspen Plus version 12.0, the Aspen Properties database contains more than 10,000 pure components; however, the IL class of compounds is barely represented. Consequently, process simulations of IL-based processes using commercial software imply the generation of additional nondatabank components to incorporate the IL compound into the process simulator’s property system. The generation of nondatabank components essentially proceeds through two stages as follows: (i) The user specifies the minimum amount of information related to certain properties of the component and/or its structure, and (ii) the program uses the available empirical and/or predictive models to calculate the rest of the properties of the pure component that are necessary to estimate the properties of the mixtures involved in the process later in the simulation environment. For researchers in the IL field, the first success was overcoming the challenge for introducing these characteristic chemical compounds to process simulator databases. IL compounds are entered in Aspen Plus as (i) pseudocomponents or (ii) user-defined components. Section 3 details the information and procedure for each of these entry methods.

### 2.2 Pure Compound and Mixture Property Models

Regarding the description of the properties of pure components, the property system of commercial process simulators includes models for estimating the thermophysical, volumetric, and transport properties that were not included during the generation of the IL component. Although process simulators offer an implicit default path for calculating a specific property, several alternative models used to be available. In each case, the most adequate option should be selected based on proper validation through comparison with the available experimental data.

In the modeling of the IL-based process using commercial software, the next step to advance is the selection of the thermodynamic model that is used to describe the mixture behavior and equilibrium between immiscible fluid phases (gas/vapor/liquid–liquid). In process simulators, the property estimation system is organized around the thermodynamic models that play the leading role in organizing the property systems of multicomponent schemes. Thus, thermodynamic models acquire the category of the Base property method in Aspen Plus or Hysys process simulators. Therefore, the availability of an adequate thermodynamic model to describe IL-based systems is crucial for the success of the simulation. Considering their physically determined developments, the thermodynamic models available in commercial process simulators (such as Aspen Plus/Hysys) can mainly be classified as equations of state (such as Peng–Robinson (PR), Redlich–Kwong (RK), or Soave–Redlich–Kwong (SRK)) and activity models (such as nonrandom two-liquid (NRTL), universal quasichemical (UNIQUAC), UNIFAC, conductor-like screening model for real solvents (COSMO-RS), and COSMO segment activity coefficient (COSMO-SAC)). Thermodynamic models, on the other hand, are usually classified as regressive (when their adjustable binary interaction parameters are obtained by fitting experimental data, as in PR, RK, NRTL, or UNIQUAC) and predictive (which can be used for any mixture without using its experimental information because its parametrizations are derived from experimental data obtained from a wide range of samples of different mixtures, as in UNIFAC, COSMO-RS, and COSMO-SAC). Notably, predictive models have been, by far, the most used in IL-based process simulations mainly owing to (i) the lack of experimental data for systems containing IL components and (ii) the require for extensive screening to select ILs that have the best properties for a specific application. In this regard, the main advantage of COSMO-based models is that they can be easily used for any new IL containing novel cations and/or anions because COSMO-based methods only require the electronic information on the IL molecular structure provided by quantum chemical calculations; therefore, it is a useful a priori predictive method to be used in process simulations with ILs never synthesized before. Furthermore, COSMO-RS/SAC provided reasonable predictions of the thermodynamic properties of mixtures containing IL and any kind and number of chemical compounds (aromatics, aliphatics, water, alcohols, ketones, gas solutes, etc.), which could move through different IL molecular models (ion pairs, independent ions, and ion clusters) to improve the descriptions of IL systems. In fact, in several process simulations, a common strategy is enormous preliminary COSMO-RS/SAC screenings, including a huge number of cations and anions to select a limited number of ILs that have favorable thermodynamic properties (separation capacity and selectivity), which are later used to analyze the process simulation. This corresponds to the product design approach depicted in Figure 1. As this Review reports, the COSMO-based/Aspen methodology proposed by Ferro et al.^33,51^ has been the most used approach in IL process simulations. Remarkably, no consistency problems or calculation errors have been found in COSMO-based/Aspen process simulations that describe multicomponent mixtures in complex processes that have several interconnected units. Lei et al.^86,107,123^ also widely applied the UNIFAC model by extending it from conventional compounds to IL systems and presented the advantages of its easy and effective use in the Aspen Plus commercial simulator and its adequate predictability in the conceptual design of IL-based processes. Several proposals to describe the IL structure (cations, anions, and substituents) by the group contribution (GC) method (as described in more detail in Section 3) have been reported, and the UNIFAC/Aspen approach reasonably predicted the thermodynamic properties of IL-based systems. An alternative product design approach (Figure 1) has been the combination of COSMO-RS/SAC predictions with the UNIFAC model to estimate the GC parameters used to define the IL compound when a lack of experimental data were available, for example, for previously unidentified cations or anions.^80^ Then, IL processes could be simulated using commercial Aspen software by applying UNIFAC as the property method.^89,124^ Regressive thermodynamic methods (mainly PR, SRK, and NRTL), on the other hand, have also been used with well-known IL-based systems in process simulations according to the available experimental data. Because experimental thermodynamic data are commonly limited to a biphasic system in specific temperature and pressure ranges, regressive-model-based process simulations have been used to design specific operational units (for example, extraction columns) when NRTL binary parameters are obtained from liquid–liquid equilibrium curves of ternary systems. Because of the independence of the employed model, the predicted values of the thermodynamic properties of IL-systems must be validated compared to the available experimental data, particularly for the key parameters determining the IL-process design, for example, the miscibility between phases, separation capacity, and selectivity in separation processes. Once the properties of the pure IL compound and thermodynamic model describing the IL-based system have been defined, the property system of the commercial process simulator, such as Aspen Plus, uses empirical mixing rules, kinetic models, etc. to estimate the thermophysical, volumetric, transport, and other properties of IL-based mixtures that are required to design the process operations. Thermochemical properties are also involved in process simulations for some applications where IL is a reactant, such as in chemical absorption. In these process simulations, the IL-based reaction product must be defined based on its original role in the reaction (a pseudocomponent or user-defined component) and must be entered into the process simulation with the standard formation enthalpy and Gibbs energy of formation of the IL and its products. In process simulators, reactions involving operation models (reactors and columns) include different alternatives to specify the reaction types, stoichiometry, and thermodynamic and/or kinetic relationships of the reaction. In this respect, process simulation studies involving IL reactions have used quantum-chemical calculations to screen thermochemical data and preliminarily select ILs that have favorable reaction equilibrium constants and enthalpies; for example, the reaction between CO_2_ and IL generated a product but was reversible at relatively low temperatures and had relatively low reaction exothermicity.^54,125^

### 2.3 Operation Unit Design and Simulation

The next step in using process simulators for modeling an IL-based process is to choose the operation or block model for designing each specific operational unit (absorber, distillation column, reactor, heat exchanger, etc.) involved in the process (Figure 1). Various models for the most common operations in chemical engineering are available in commercial process simulators and are widely classified as simplified (which use a user-specified operation design parameter to characterize the models’ behavior or are built based on simplifications) and rigorous (which can simultaneously resolve material and enthalpy balances, phase/reaction equilibrium relationships, and heat/mass transfer kinetic equations by component and stage by stage). IL process simulation studies have used rigorous operational unit models, as described in detail in Section 3. Rigorous models, such as the RADFRAC column in Aspen Plus, have enabled simulations to be conducted using an equilibrium mode (where the separation is controlled by the thermodynamic equilibrium) or a rate-based mode (where mass-, energy-, and momentum-transfer kinetic equations are introduced, enabling the analysis of a possible kinetic control in the operation). These simulation alternatives are important in IL-based processes because these high-viscosity solvents may limit the process kinetics and determine the IL selection criteria. Once the operational unit model has been selected, the process simulations enabled the preliminary balancing of the material and enthalpy and the initial decisions to be made regarding the operational design for treating complex multicomponent mixtures. Thus, the sensitivity of the operating variables (temperature, pressure, number of stages, etc.) are commonly analyzed to evaluate the separation efficiency (recovery and purity) or reaction conversion (Figure 1). The results of enormous and systematic sensitivity analyses have enabled the selection of adequate operating conditions for modeling the process and, of considerable interest in this field, for comparing the process performance of different ILs. Alternatively, design specifications can be established (for example a fixed solute recovery or reaction conversion) by the process simulator, which can set the variable values (temperature, pressure, number of stages, etc.) that are guaranteed to achieve the specification. This approach is also especially useful for comparisons and enables the evaluation of the process performance of different ILs according to the specific solvent and energy consumptions required to achieve identical separation or conversion. Regarding IL consumption, the use of mass units is important to avoid misleading conclusions related to different IL molar weights. Some studies have used only the process simulation results of the main operational unit (absorber, reactor, etc.) for selecting ILs based on a wide range of samples of new or known cations and anions (corresponding to the simultaneous Product and Process Design I (PPD I) approach in Figure 1). Finally, processes can be simulated to select and size equipment. Thus, rate-based models can be used to select the best internal column (packing or tray type) to be used with a specific IL under defined operating conditions to maximize the mass-transfer rate and minimize the pressure drop. In addition, rate-based calculations have been used to calculate the column diameter, obtain a reasonably fractional capacity (60–80%), and specify a column height that guaranteed the desired separation under fixed operating conditions that fulfilled the industrial height/diameter standards. The design of the main application operation (carbon-capture absorber, carbon conversion reaction, extractive distillation column, etc.) by process simulation is considered as a process design stage for continuously improving the IL-based process performance (Process Design I (PD I) approach in Figure 1).

### 2.4 Process Modeling and Simulation

Once the main operational unit (absorber, extractor, reactor, etc.) has been designed, the next step is to model the complete base process to advance the conceptual and basic engineering of the evaluated IL industrial application. The development of a process model comprises the convenient articulation of the sequence of operational models involved in the complete process, including the main operation, IL regeneration section units, heat exchangers, pumps, and compressors. This stage is required for reliably evaluating the KPI of the IL-based process, including the chemical and energy consumptions, environmental impacts, and process costs. One main issue is that the IL regeneration stage plays a key role in the global energy demand of the process owing to the required temperature or pressure swing. Some process simulation models use open-cycle processes (without recycling) for preliminarily evaluating the process performance. However, modeling the almost complete IL-based process, including recycling, is crucial for IL reuse and product recovery to enable the description of the effects of the partial IL regeneration on the solvent flow and energy requirements. In addition, additional operations are common for conditioning recycling, which change the stream temperature and/or pressure and imply further energy duties. Additionally, recycling is used to imply larger equipment, which increases both the operating and capital costs. In complete process modeling, other relevant information to consider is whether to include makeup and purges so that both describe a more realistic process and facilitate simulations convergence. In process simulation analyses, the IL makeup is important for evaluating different IL replacement scenarios owing to the uncertainty in the IL stability in industrial operations. To model the complete process, the heuristics suggest following the following design sequence: Reactor → Separation and purification units → Thermal and pressure conditioning → Energy integration → Closing process (recycling and makeup). Following this approach, very complex IL-based processes have been modeled as integrated carbon-capture and conversion processes to produce cyclic carbonates using the COSMO-based Aspen approach. Once the complete process has been modeled, additional simulations are conducted for reliably analyzing the technoeconomic at the conceptual/basic engineering level. Thus, the chemical and energy consumptions can be calculated by considering the realistic IL flow and cyclic separation capacity/selectivity or reaction conversion/selectivity. In addition, the equipment sizing considers realistic flow rates. Capital and operating expenditures (CAPEX and OPEX, respectively), which comprise the total annualized cost (TAC) of IL-based processes, are now estimated. Some process simulation studies have analyzed partial cost estimations as process equipment and variable operating costs instead of CAPEX and OPEX to find more sensible changes in operating variables. Increasing the inlet flow of the treated streams (or the outlet flow of the obtained product(s)) has enabled economies of scale to be analyzed in IL-based processes. In addition, the combination of process simulations with the LCA methodology has enabled the estimation of the environmental impacts of IL-based processes, such as global warming, human toxicity, water ecotoxicities, and terrestrial acidification. This approach extends the assessment of promising ILs in large-scale industrial applications to environmental sustainability. In this respect, process simulations have also enabled the estimation of the CO_2_ equivalent emissions associated with IL-based processes. At this process design stage (Figure 1), the sensitivity of the different unit operating variables can be analyzed and process configuration can be redesigned to improve one or more KPIs of the IL process. This approach is consistent with the iterative activity of the synthesis and analysis of alternatives to improve the process design in an engineering project as follows: Conceptual design → Dimensioning and basic design of the equipment → Estimation of costs → Improvement of the conceptual design, and repeating the sequence as many times as necessary (Process Design II (PD II) in Figure 1). Remarkably, this stage enabled the proper selection of the IL by attending to its key properties for enhancing the process performance. In fact, at this point, a multicriterion IL can be selected based on not only KPI values but also IL compound constraints (thermal stability, melting point, and viscosity), environmental impacts related to IL synthesis and IL-based processes, IL price and availability, etc. Therefore, simulations of the complete IL-based process clearly contribute to the simultaneous Product and Process Design II (PPD II), as depicted in Figure 1.

### 2.5 Process Optimization

In process simulation studies, an effective engineering strategy is the optimization of the key operating variables of IL-based processes for minimizing the solvent and utility consumptions, equipment size, and, hence, CAPEX and OPEX, which constitute the TAC. The formulation of an optimization problem includes: (i) the definition of the independent variables and their variation intervals; (ii) the response or objective function selected as an optimization criterion; (iii) the type of extrema (maxima or minima) of the objective function for which the optimization algorithm must search; (iv) the definition of a set of fixed physical, technological, economic, and other restrictions; and (v) the selection of the optimization method. The independent variables must be carefully selected based on those that determine the studied response. The chosen response function must represent the essence of the studied relationship. Economic variables are often used as optimization criteria. In process simulation studies on ILs, the TAC has been widely used as the objective function to optimize IL-based processes (Process Design III (PD III) in Figure 1). Relevant studies have integrated IL design optimization and IL-based process design by employing molecular simulations or surrogate models, demonstrating the strongly interlinked molecular, phase, and process levels of IL-based processes (Product and Process Design III (PPD III) approach in Figure 1).

### 2.6 Comparison with Available Technology

Once the technoeconomic and environmental analyses have been performed and the process has been optimized, the last step in process simulation studies is commonly to compare the KPIs of the IL-based process to those of the benchmark industrial process to assess the feasibility (competitiveness and sustainability) of the new IL-based technology. Several studies have compared the specific KPIs (expressed based on the mass of the recovered or produced compound) obtained by simulating IL-based processes to the available KPI data reported in the literature, which were obtained for different inlet streams and separation or conversion grades and implied that the specifications were neither the same nor commercial. A more reasonably approach would also be to modeling the benchmark industrial process in comparable inlet and outlet stream specifications, to obtain draw reliable conclusions about the potential advantages of the proposed IL-based processes. A detailed comparison with the current industrial technology may guide further improvement in the IL design and/or IL-based process design (PPD IV depicted in Figure 1).

Finally, the prospective analysis of the IL-based process performance conducted using process simulations based on predictive thermodynamic methods (Figure 1) can be used to (i) guide experimental research on IL designs by focusing on the key properties for enhancing the IL-based process performance or operation conditions to be experimentally validated in the design of enhanced processes; (ii) develop a digital prototype of IL-based processes to be used in future technological developments; and (iii) focus on the design, construction, and operation of pilot plants to validate the technology at the laboratory scale (TRL 4) or in a relevant industrial environment (TRL 5) and save time and costs in the development and marketing of proposed IL-based technologies.

## 3 Methodology for Applying Process Simulations to Ionic Liquids

**Scheme 1:**
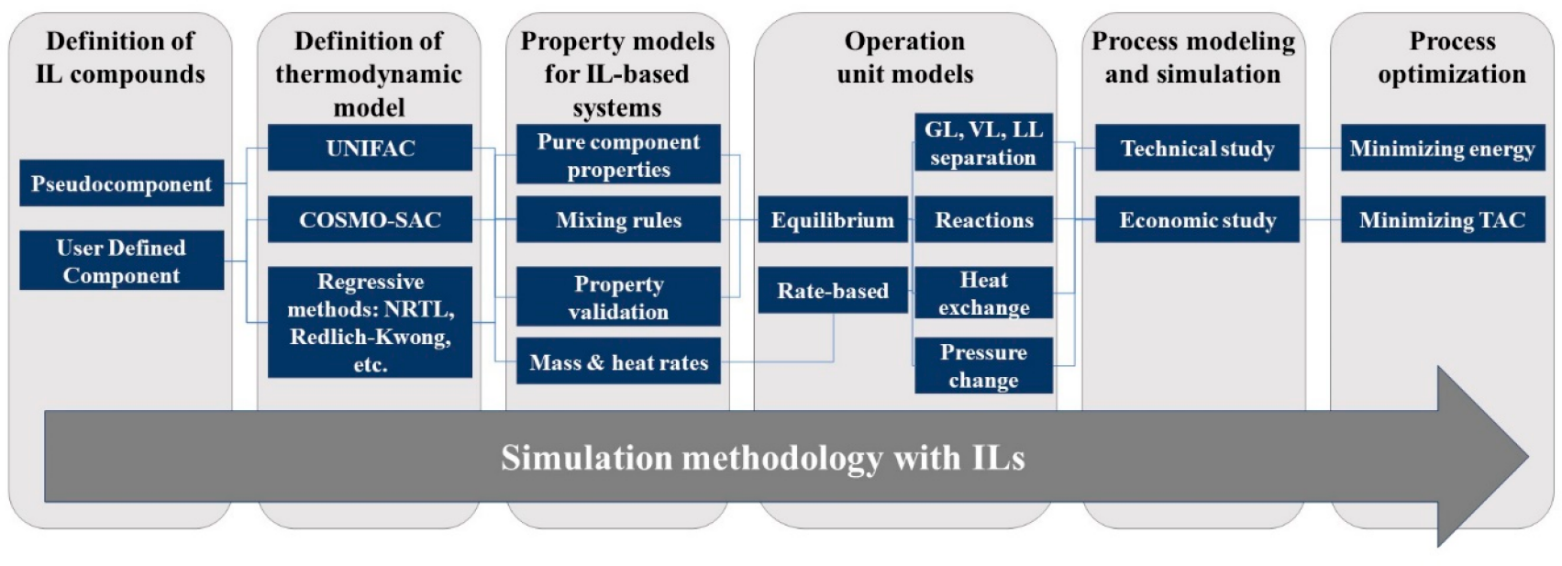
Methodology for Simulating IL-Based Chemical Processes

In this section, the typical methodology for simulating IL chemical processes was overviewed (Scheme 1) based on the steps required to simulate these IL-based processes using Aspen Plus and/or Aspen Hysys software, both of which are market-leading process simulators that are employed worldwide by chemical companies and university students. One of the most important advantages of these software programs is that they have enabled the integration of rigorous process modeling with economic, energy, safety, and emissions analyses and, therefore, have been the preferred option for studying massive IL-based systems, as reported in Tables 1, 2, 4, 5, 6, 7, 8, 9, 10, 13, and 14. Notably, in some studies, other commercial software (as gPROMS) or in-house mathematical models comprising equations that describe corresponding operations have been used for modeling IL chemical processes.^8,117−119,126^ The gray boxes in Scheme 1 indicate the phases of the typical methodology for simulating chemical processes with ILs that will be explained so that readers can address process simulations with ILs.

**Table 1:**
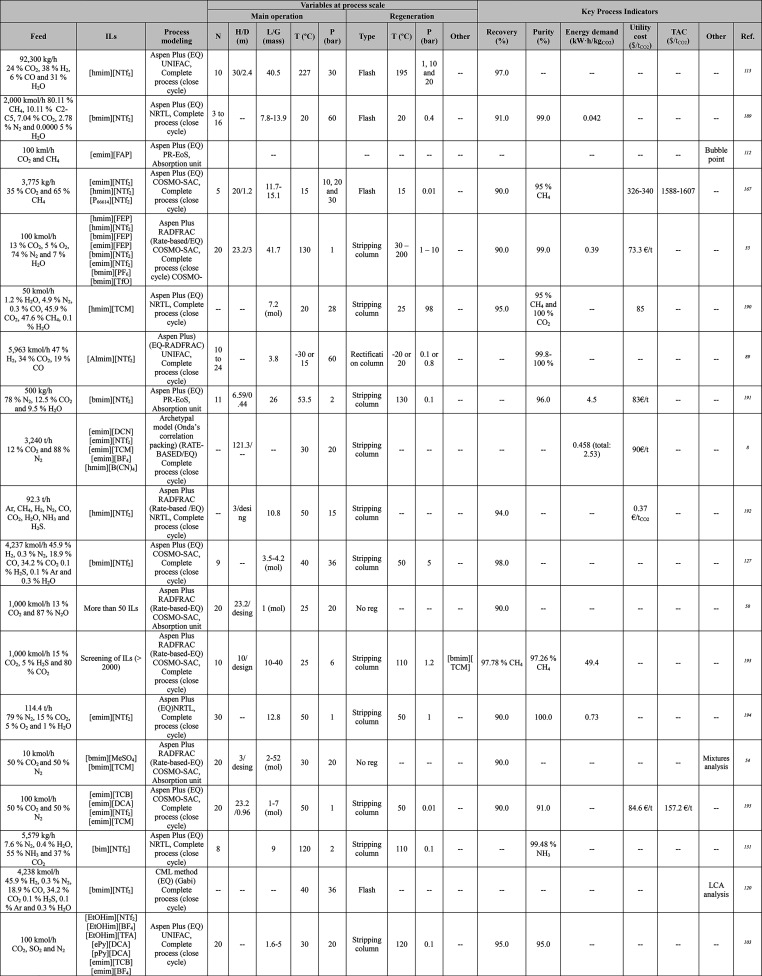

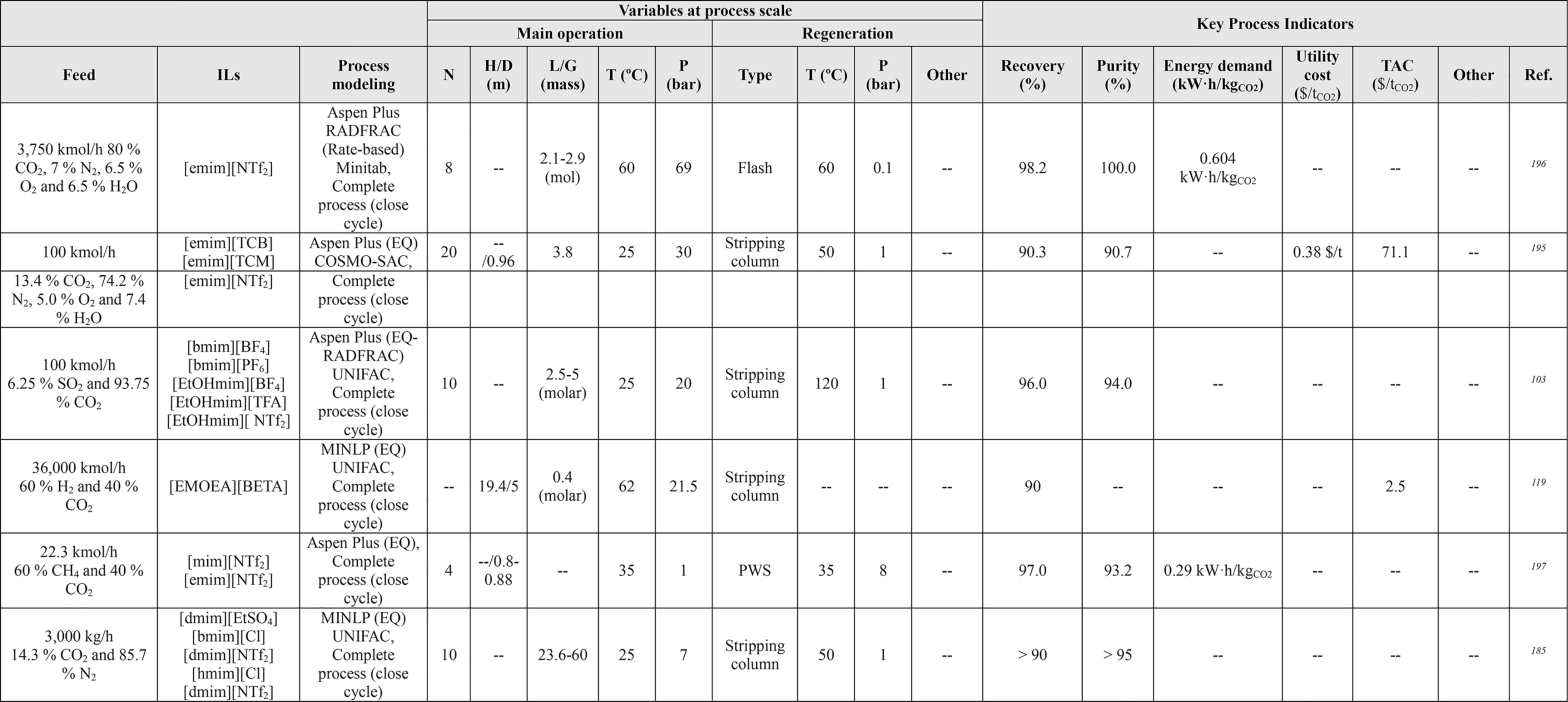
Different Systems Evaluated Using Simulation Processes for IL-Based Physical Capture of CO_2_, Including Process Modeling, Operating Variables, and KPIs

**Table 2:**
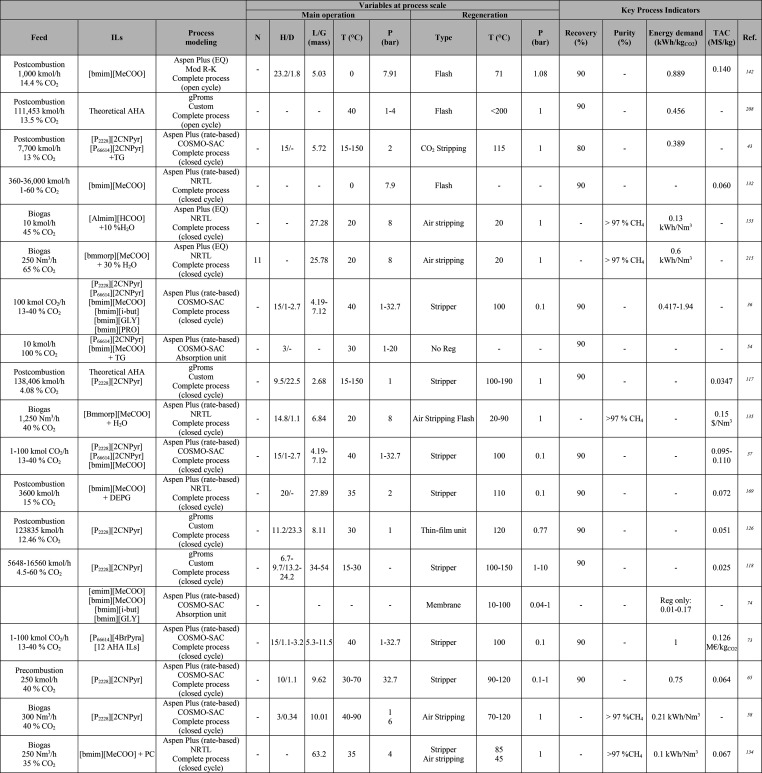
Process-Simulation Studies on IL-Based Chemical CO_2_ Capture, Including Process Modeling, Operating Variables, and KPIs

### 3.1 Definition of Ionic Liquid Compounds

The initial stage for addressing any kind of process simulation is to define the properties of the studied systems (Scheme 1). First, if ILs are unavailable by default as conventional components in simulators’ databanks, they must be generated by the user. New IL components can be easily included as pseudocomponents in Aspen Plus by specifying their molecular weight (MW), normal boiling point (NBP), and density (at 60 °F).^51,106^ These properties can be experimentally measured or estimated using predictive methods that are described below as COSMO-RS.

Otherwise, the IL may be added as a user-defined conventional component.^89,127^ The User-Defined Component Wizard can be used to define the properties required for components that are not contained in any pure-component databanks of Aspen simulators. This wizard helps the user to enter property data that are commonly available for the components. The component’s formula and molecular structure can also be introduced. The molecular structure, MW, and NBP are the most fundamental information required for group-contribution and corresponding-state methods used for estimating properties. If the molecular structure is specified, MW can be calculated based on the atoms. Although NBP is not required for property calculations, it is used to estimate many other properties, such as the critical temperature and pressure, if they are missing. Additional property information, such as the density/specific gravity (at 60 F), vapor pressure, ideal-gas heat-capacity data, and standard formation enthalpy or Gibbs energy of formation, can be entered. However, not all these properties must be specified. All the property parameters can be estimated based on the molecular structure using either the NIST TDE (ThermoData Engine) or Aspen property estimation system. For ILs, user-introduced property parameters are the preferred option of researchers. If no experimental data are available, properties can be estimated using applied methods reported in the literature. In that regard, critical properties can be obtained using the GC method developed by Valderrama et al.,^128^ and ideal gas and liquid heat-capacities can be calculated using the Joback model extended for ILs, as developed by Ge et al.,^129^ among the multiple methods available in the literature for predicting these properties.

Notably, in chemical processes in which the IL is a reactant—in other words, a chemical reaction occurs between the IL and other compound in the simulated process—the product of the IL-based reaction must be defined as either a pseudocomponent or user-defined component (Scheme 1).^36,43,68,73,75,125^

### 3.2 Definition of Thermodynamic Model

Once IL compounds have been generated, multiple thermodynamic methods are used for properly establishing the properties of IL systems in Aspen simulators, which is the next step according to Scheme 1. The selection of the thermodynamic method is crucial for correctly describing binary or multicomponent systems, such as gas–liquid, vapor–liquid, or liquid–liquid equilibria, depending on the operational units to be modeled. Although equations of state (EoS) are used in the literature, activity coefficient models are the most extended (Tables 1, 2, 4, 5, 6, 7, 8, 9, 10, 13, and 14). Briefly, the available thermodynamic models could be classified as predictive or regressive. The former can estimate thermodynamic properties without requiring any previous data related to the system that is being evaluated, whereas the latter must be fitted to experimental or calculated data ad hoc for the studied system, depending on the data availability. Thus, when regressive methods, such as the Wilson equation, NRTL, and UNIQUAC models,^130−139^ and EoS, like PR, RK, SRK,^140−142^ or cubic plus associated (CPA),^59^ are used, either experimental or predicted data must be given to determine the binary interaction parameters by model fitting. Aspen Plus has a regression tool that has enabled the calculation of binary parameters. Presently, these state-of-the-art thermodynamic methods are a useful approach for describing the behavior of well-known ILs in experimental-data-based process simulations. Nevertheless, the gap between the volume of information required to evaluate the potential industrial applications of ILs and the current data acquisition limits the use of these methods. Therefore, predictive methods are much more widely used because they do not rely on very time-consuming experimental measurements.

Regarding purely predictive thermodynamic models, UNIFAC- and COSMO-based methods are the most employed during modeling to determine IL properties.^123^ UNIFAC is currently very popular in process simulations, as suitable as NRTL or UNIQUAC for both nonpolar and polar systems, and widely applied for quantitatively predicting the gas–liquid, vapor–liquid, and liquid–liquid thermodynamics of IL-containing systems. UNIFAC estimated the liquid-phase activity coefficient of binary or multicomponent systems, even when experimental equilibrium data were nonexistent.^143^ The original UNIFAC model was extended from conventional solvents to IL systems by Lei et al.^86,107^ to further develop more complete UNIFAC models for ILs comprising 75 main groups and 130 subgroups and currently covering broad pressure (0.01–500 bar) and temperature (from −30 to 180 °C) ranges.^123^ Modifications of the original UNIFAC model were also proposed to improve the prediction accuracy for activity coefficients and, thus, the description of the IL behavior. Dortmund modified the UNIFAC-GC activity coefficient model developed by Gmehling et al.,^144−147^ and Lyngby modified the UNIFAC activity coefficient model developed by Larsen et al.^148^ These modifications are included in Aspen Plus software as UNIF-DMD and UNIF-LBY, respectively. In addition, a UNIFAC model that has group interaction parameters specifically designed for liquid–liquid systems is available. As a GC method, UNIFAC has a predictive capability that relies on experimentally measured property data to estimate the group contribution parameters and extrapolation limits and IL systems. First, IL groups must be split before applying UNIFAC to ILs, as described in detail elsewhere in the literature. In summary, three main methods are used for decomposing IL groups as follows: (i) ILs are divided into cations and anions; (ii) ILs are decomposed into several individual groups comprising the anion, cation skeleton, and other parts excluding the cation skeleton; and (iii) although ILs comprise several groups, as proposed by Lei et al.,^107^ the anion and cation skeletons comprise one electrically neutral group. Despite all the published group parameters and group binary parameters that are stored in the Aspen Physical Property System for most conventional components, for nondatabank ILs, all the UNIFAC groups must be added to, and the functional groups required to make each component must be defined in the system to model the process after selecting one of the available UNIFAC-based thermodynamic methods. For UNIFAC groups, the user must enter the group volume (GMUFR), surface area (GMUFQ), and group interaction parameters. Detailed instructions can be found elsewhere.^89,106^

**Scheme 2:**
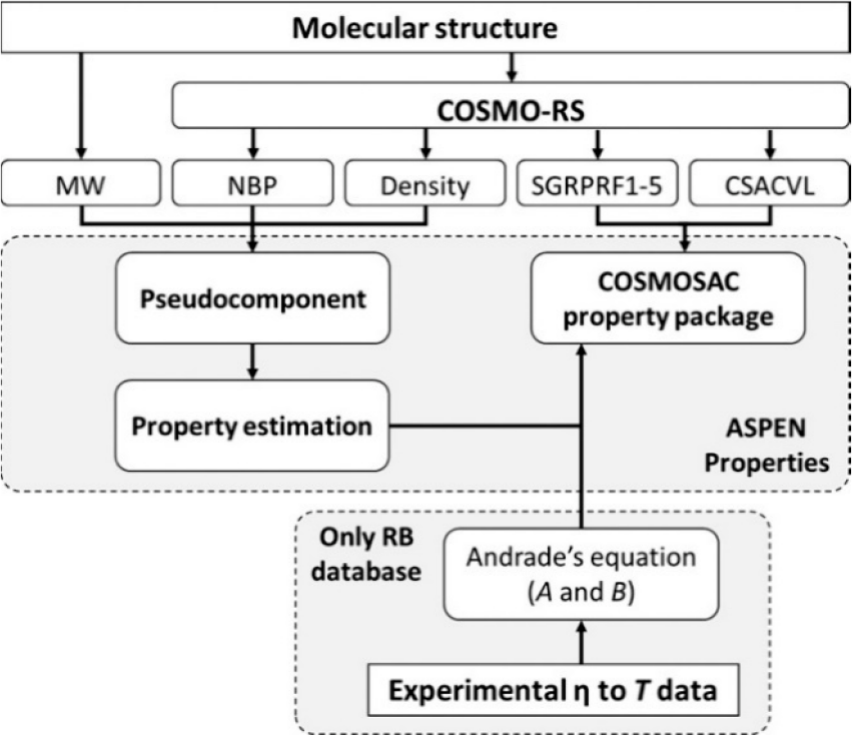
Information Flow Used for Both Generating Pseudocomponents and Specifying COSMO-SAC Property Method in Aspen Plus. Reproduced from Ref (51). Copyright 2018 American Chemical Society.

Based on the COSMO continuum solvation method, the COSMO-RS and COSMO-SAC models are quantum-chemistry-based predictive methods for estimating the chemical potentials of liquids.^123,149−151^ COSMO-RS, proposed by Klamt,^149^ computes the charge density polarity (σ) in the solute–solvent context. A σ-profile histogram, which reports the discretization of the molecule in different segments of the polarized charge surface with the estimated chemical potential of each segment is created. Hence, the σ-profile represents the affinity of one or more molecules to a determined polarized segment and, together with the thermodynamic relationships, enables the calculation of the chemical potential of the solute in the solvent, i.e., the activity coefficients.^123,149^ In addition to activity coefficients, COSMO-RS has been applied to determine the VLE, LLE, gas solubility, etc. of a broad set of IL-associated systems. In fact, COSMO-RS can be used to determine the MW, NBP, and density of ILs to be defined as pseudocomponents, as shown in Scheme 2. Lin and Sandler, on the other hand, developed the COSMO-SAC method using a COSMO-RS-framework-based GC solvation method.^151^ This model has been applied to many IL-based systems and has several posterior versions. Concerning process simulations in Aspen Plus, the COSMO-SAC property method has three user-selected COSMO equations as follows: code 1 represents the original COSMO-SAC model proposed by Lin and Sandler^151^ and is the default model in Aspen Plus; code 2 represents the original COSMO-RS model proposed by Klamt;^149^ and code 3 represents the modified Lin and Sandler model.^152^

Ferro et al.^51^ proposed a procedure to develop additional nondatabank compounds and specify the COSMO-SAC property model, as depicted in Scheme 2. This approach is referred as the COSMO-based/Aspen Plus multiscale methodology. By following this procedure, an enterprise IL database (ILUAM) containing 100 ILs was developed in 2018 for use with COSMO-based property methods in the Aspen ONE program suite.^51^ The ILUAM database is available online free of charge for the scientific community.

As shown in Scheme 2, the property computation following this approach is based on the COSMO-SAC property package regardless of the chosen code (1, 2, or 3), i.e., COSMO equation.^51^ To support COSMO-SAC property calculations, only the molecular volume (CSACVL) and σ-profile (SGPRF) remain. The former is added as the scalar parameter (CSACVL) to the Aspen Property System, whereas the latter is entered as a set of five temperature-dependent parameters, designated from SGPRF1 to SGPRF5. Therefore, the COSMO-SAC property model can be specified solely based on the information generated using the COSMO-RS computational method (MW, NBP, density, CSACVL, and σ-profile) for neat ILs (and their reaction products if a chemical reaction is involved).^36,43,68,73,75,125^ For describing IL compounds, two different molecular models, including ion pairs (CA model) and independent ions (C+A model), can be considered when obtaining these data through the use of the COSMOtherm program package.^33,51^ Because each model generates different results in the predicted properties, the models (CA and C+A) should be validated using experimental data.^33^ The remaining physical and thermodynamic properties, which are necessary for fully defining the IL components, can be estimated using the API-recommended procedures and Aspen Physical Property System modifications implemented by default in Aspen Plus.

COSMO-based models, both COSMO-RS and COSMO-SAC, have also been combined with the original UNIFAC method.^80,88,153^ This approach extends UNIFAC by regressing the activity coefficients estimated using COSMO-based models to cover the binary interaction parameters that are missing in UNIFAC.^123^ Then, the process can be simulated using commercial Aspen software by applying UNIFAC as the property method.^80,88,124,153^

According to the literature, although it is not as widely used as the UNIFAC- and COSMO-based methods, the perturbed-chain statistical associating fluid theory (PC-SAFT) EoS has also been used as a thermodynamic model to simulate processes in Aspen Plus,^115,154^ especially gas-separation processes, owing to its good prediction capacity of gas solubility.^116,123^ However, despite the predictive ability of the PC-SAFT model, PC-SAFT parameters are often obtained from experimental data. PC-SAFT requires parameters for the number of spherical segments forming the chain, the hard sphere segment diameter, the segmental energy parameter, and the association energy and volume for the components used in simulations.^115,154^ On the other hand, data-driven quantitative structure–property relationship (QSPR) models, using machine learning techniques for the molecular representation, have gained attention for predicting key thermodynamic properties in IL-based process, due to their efficient implementation in process optimization methods.^119,155,156^

### 3.3 Property Models for Ionic Liquid Systems

Sometimes accounting for other relevant aspects for modeling IL-based processes may be interesting because these aspects might be related to the pure component, binary interaction properties, mixing rules, and/or mass-transfer kinetics for rate-based calculations, as depicted in Scheme 1.

For pure components, the IL viscosity is a good example of this because it is a key property in gas-absorption processes. As shown in Scheme 2, in Aspen calculations, experimentally measured ILs viscosity to temperature dependent data can also be accounted in the Andrade equation^35,43,51^ to improve the description of the mass-transfer process. Because ILs are nondatabank compounds, the required parameters are missing from the databanks and must be entered by the user. After they have been fitted using the Andrade equation, the property parameters for the IL viscosity are introduced to Aspen calculations.^51^ This approach can be extended for other temperature-dependent properties, such as the liquid molar volume, liquid surface tension, or heat capacity.^131^ Scalar parameters can also be defined for pure components. In addition to the critical properties or MW^131,132,136^ that are specified when defining IL compounds, another example is the enthalpies defined for the reactions between the IL and other components. de Riva et al.^43^ and Hospital-Benito et al.^36,73^ solved this problem by varying the IL formation enthalpy to adjust the reaction enthalpy by following Aspen Tech’s recommended procedure and introduce it to Aspen Properties. Aspen simulators have a help resource in which the user can consult the models and equations for each property. Predictive property methods are useful for predicting the properties of IL compounds when experimental data are missing. Quantitative structure–activity relationship (QSAR) or QSPR approaches^157^ and GC models^158−160^ are useful computational tools that have been developed and employed to estimate many IL physicochemical properties, such as viscosity, density, and heat capacity. Moreover, artificial intelligence techniques such as machine learning are today worthy of attention for QSPR modeling in predicting properties of ILs.^161,162^

Similarly, scalar and temperature-dependent binary interaction properties, such as Henry’s law constants, can both be entered. To use the experimental data or solubilities predicted using the QSPR, GC, EoS, or PC-SAFT methods^123^ rather than the selected property method, namely UNIFAC or COSMO-SAC, the gas’ physical solubility can be specified based on Henry’s law in the Aspen Property System.^132^ To do so, the gas must be defined as a Henry component, which requires parameters to be defined for the temperature-dependent expression. Notably, the Aspen Plus software considers the activity coefficient of the gas when computing the molar fraction according to Henry’s law. Hospital-Benito et al. detailed the procedure elsewhere in the literature.^36^

Mixing rules are particularly important for accurately representing the properties of IL-containing mixtures. For properly describing the density or viscosity of IL-containing blends the default methods should not be used. The quadratic mixing rule for the liquid volumes of pure components (VLMXQUAD) is recommended for calculating the molar volume of liquid mixtures. To calculate the density of binary IL mixtures, the VLMX26 method has been used to ensure consistency with the density calculated for pure ILs.^53^ The Wilke–Chang correlation is widely adopted to estimate the infinite-dilution diffusion coefficient of gas components in ILs (DL0WCA and DL1WCA).^8,35,50^ The MULXASTM liquid mixture viscosity method for viscous hydrocarbons and Andrade model for the viscosity of pure liquids are suitable for computing the viscosity of IL blends, whereas the molar enthalpy of liquid mixtures can be calculated using an asymmetric method and the ideal gas, RK, Henry’s law, and NRTL models (HLMX30).

**Figure 2:**
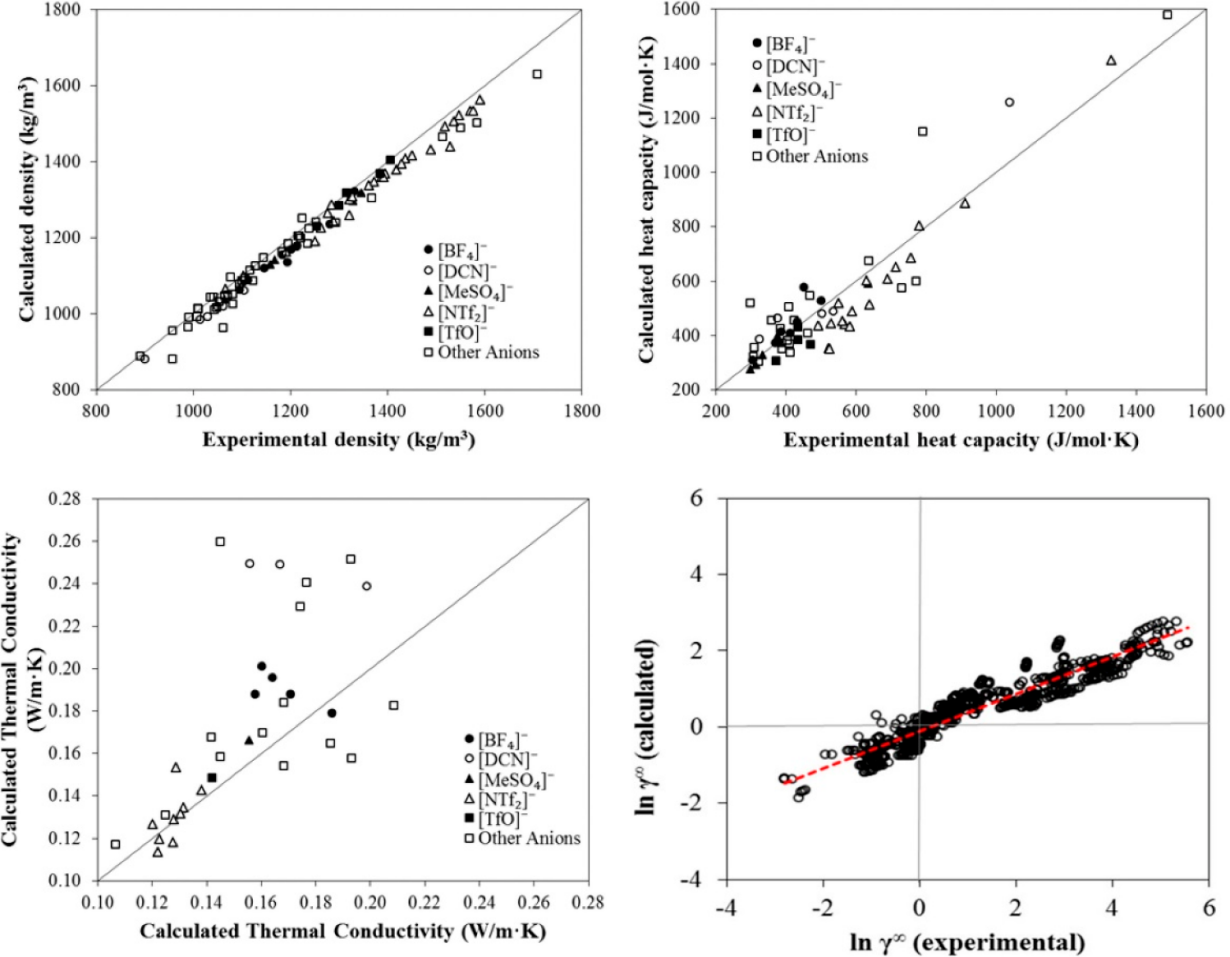
Comparison of experimental and COSMO-SAC-calculated property and activity coefficients of ILs. Reproduced from ref (51). Copyright 2018 ACS.

Whenusing only predictive methods to estimate the properties of IL components and mixtures, validating the results and comparing them to the available experimental data are always convenient. To do so properly, a wide representative range of values should be considered for the property being validated. Therefore, for validating properties and thermodynamic methods, the inclusion of the largest possible number of ILs and compounds might be crucial to evaluate the prediction capability and accuracy. For regressive thermodynamic models, predictions are evaluated using statistical parameters, such as the correlation coefficient (R^2^), mean absolute and/or relative errors, and average absolute relative deviation.^51,136^ In summary, validation must ensure an adequate level of accuracy for the predictive method to be used in simulations for the conceptual design of IL-based processes. For instance, COSMO-SAC-estimated pure component properties commonly used in process simulations corresponding to conceptual and basic engineering, as density, heat capacity and thermal conductivity have been validated, as shown in Figure 2. Additionally, activity coefficients at infinite dilution predicted by COSMO-SAC are compared to experimental data (780 data points) for 11 representative chemical compounds in 21 ILs. Without using any experimental data, COSMO-SAC clearly and reasonably predicts the properties and thermodynamic behaviors of IL-based systems. Small differences were observed, on the other hand, when the CA and C+A molecular models were used to describe the IL compound; however, compared with the C+A model, the CA molecular model more accurately predicted the properties of the pure IL overall.^51^

**Figure 3:**
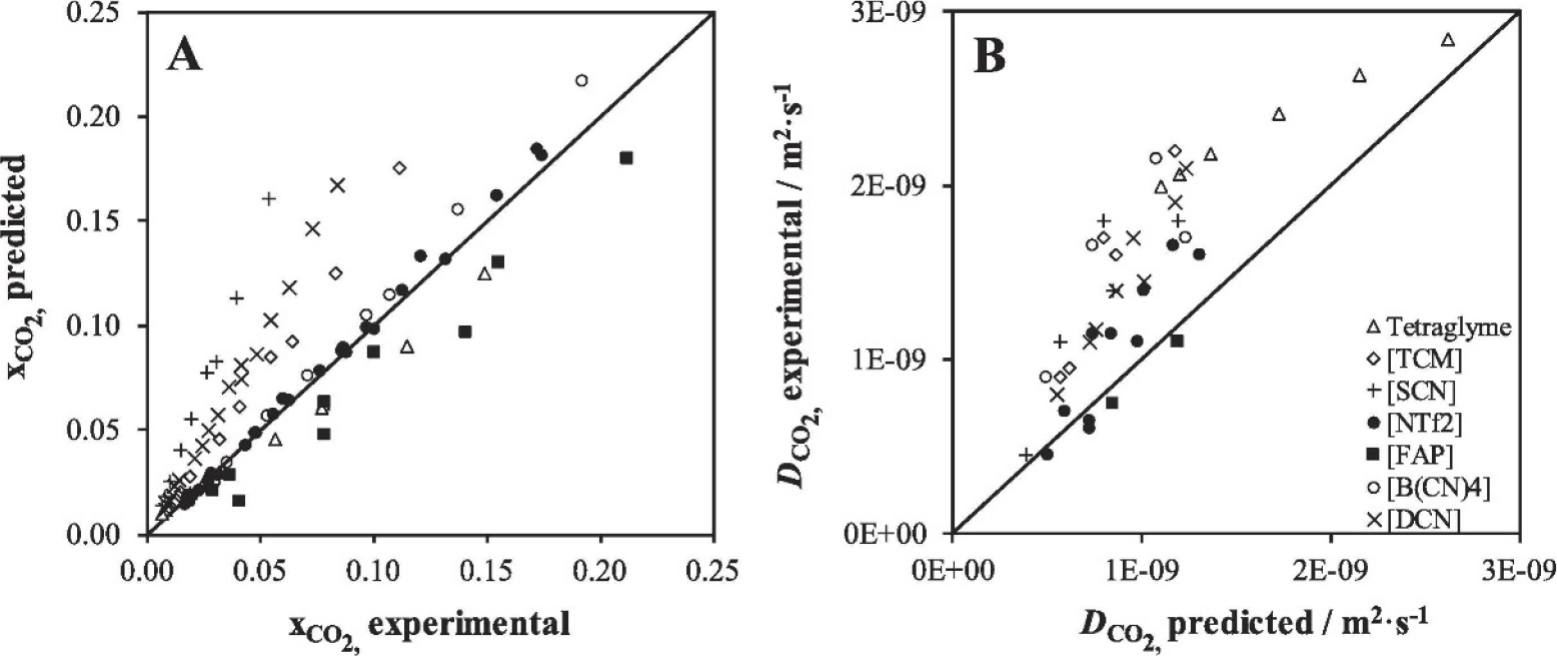
COSMO-SAC-predicted molar solubility (A) and diffusivity (B) of CO_2_ in ILs vs. experimental values. Symbols indicate anion forming IL. Reproduced from ref (50). Copyright 2019 Elsevier.

Validations are also recommended for predicting the relevant thermodynamic and kinetic parameters of IL-based mixtures for designing specific process operations. For instance, Palomar et al.^50^ validated the COSMO-SAC/Aspen predictions for the CO_2_ molar solubility and CO_2_ diffusivity in ILs, both of which are quite relevant for physical CO_2_ absorption. Figure 3A compares the CO_2_ solubilities measured at various temperatures and pressures in ten different ILs and tetraglyme to the corresponding solubilities calculated using COSMO-SAC and Aspen Plus, whereas Figure 3B compares the CO_2_ diffusivities in ILs experimentally measured at different temperatures and 1 bar with the corresponding diffusivities calculated using the Wilke–Chang correlation.^50^ Moreover, the COSMO-SAC/Aspen approach has also been validated for the enormous amount of vapor–liquid and liquid–liquid equilibria data available in the literature.^59^

Finally, correlation methods for mass- and heat-transfer coefficients are relevant for rate-based calculations. For separation units, which comprise the vast majority of Aspen Plus-supported IL simulation studies, the Chilton and Colburn method is usually used for the heat-transfer coefficients,^163^ whereas for the mass-transfer coefficients, multiple correlations are available depending on whether it is a random-packed or structured.^164−166^ The details for representing IL-involved reactions in Aspen Plus are provided below.

In studies in which Aspen simulators were not used, the developed modeling tool included the thermodynamic method and mathematical expressions for describing the properties of the pure components and mixtures, reaction model, and rate equations required to describe the mass and heat transfers.^8,117−119,126^

### 3.4 Operation Unit Models

Subsequently, once the IL system’s properties were ready, the process could be simulated (Scheme 1). According to the literature, gas separation, liquid–liquid extraction, extractive distillation, distillation, stripping, and heat exchange were the main operations units comprising the IL-based processes for which modeling was conducted. These processes were reviewed in the following sections. Thus, a model was required for describing each of these units. For the popular Aspen Plus commercial process simulator, a Model Palette with simple and rigorous unit models is available for each operation unit.

Regarding single-stage unit models for simulating IL separation units in Aspen Plus, FLASH2 is the most accepted option for modeling the IL regeneration stage in gas separation^35,131,136,141,167^ and/or extraction processes^45,53,168^ owing to the low volatility of ILs. FLASH2 comprises one-equilibrium-stage flash separators and two output streams, which enable the resolution of the material and energy balances, and phase equilibrium equations.

Additionally, Aspen Plus has rigorous multistage column models that are suitable for modeling IL-involved vapor–liquid, gas–liquid, and liquid–liquid separations and can resolve material and enthalpy balances componentwise and stagewise, including the equilibria that are involved.

EXTRACT is a rigorous model for describing liquid–liquid extractors.^53,91,168^ EXTRACT enables multiple feeds, heaters/coolers, and side streams and typically calculates distribution coefficients based on the activity coefficient model or EoS chosen in previous steps for representing both liquid phases. Although equilibrium stages are assumed, component or stage separation efficiencies can also be specified. Because EXTRACT cannot be used for rate-based calculations, it does not support the use of the Column Internals tool for sizing and designing processes.

The RADFRAC column is a rigorous model that is used for simulating all types of multistage gas–liquid and vapor–liquid separations but also resolves the vapor–liquid–liquid equilibrium. For ILs, this model has been used to simulate both physical and chemical absorption columns^75,76,134,135,169^ as well as stripping^36,45,76^ and distillation columns (including extractive^59,110^ and reactive^170^) with and without the use of a condenser and reboiler, one or more feeds, different extractions on the top and bottom stages, and side streams.^43^ The RADFRAC column model includes two calculation modes. In the equilibrium calculation mode, the separation is controlled by the thermodynamic equilibrium, whereas in the rate-based mode, the kinetic equations for the mass, energy, and momentum transfers are introduced in addition to the Column Internals tool, which enable the analysis of the possible kinetic control during the process^35,43^ and the sizing of the column, which is usually packed.^35,43,110,167,170^ The rigorous multistage RADFRAC column model requires the specification of the number of stages assumed when operating in the equilibrium mode. For rate-based calculations, the height and diameter of the packing section are defined. To guarantee the desired separation, the height is specified based on the results of previous studies^50^ or height-to-diameter ratios.^37^ The diameter is commonly calculated to maintain a fractional capacity in the range 60–80%.^35,43,50,58,132^ With this model, either random^50,167^ or structured^58,132^ packings have been used for ILs. For operations in which chemical reactions occur, such as CO_2_ chemical absorption or reactive distillation, the Reactive-Distillation (React-Dist) reaction form has been employed to specify the reaction types, stoichiometry, and rate parameters to be used with the RADFRAC column model. This combination has enabled IL reactants to enter RADFRAC equilibrium reactions according to an Arrhenius-type equation that is used to compute the equilibrium constant.^36,43,132,169^

Aspen Plus, on the other hand, also has some unit operation models for reactors. Although all the reactor models can solve material and enthalpy balances in the chemical-reaction stage of a process, only the continuously stirred tank reactor (RCSTR) and plug flow reactor (RPLUG) can be used for preliminarily designing and sizing. Furthermore, RCSTR and RPLUG are the only models to which a reaction set must be introduced. Reactions sets contain the models used to represent chemical reactions. Although numerous chemical reaction models have been implemented in Aspen Plus, the Powerlaw model is the most widely adopted for IL reactants.^68,132,142^ As for React-Dist or whichever reaction set, the Powerlaw model is defined by the user in the Aspen Plus Simulation environment, which includes all the information about the stoichiometric, thermodynamic, and/or kinetic relationships of the reaction. In this model, kinetic reactions are specified based on the power law model (Powerlaw in Aspen Plus), whereas equilibrium reactions are entered by applying the same Aspen built-in expression as React-Dist for calculating equilibrium reaction constants.

Although the RCSTR model has been widely adopted for simulating the IL regeneration as a flash desorption stage for reversible reactions between IL and other compounds, such as in chemical CO_2_ capture,^68,132,142^ it has also been used for producing cyclic propylene and polypropylene carbonates from CO_2_ and propylene oxide, where [emim][Cl] catalyzes the reaction,^140^ or producing IL-catalyzed algal biodiesel.^171,172^ The RCSTR model handles both the kinetic and equilibrium reactions.

In contrast, among the remaining models that do not require a reaction set, the stoichiometry reactor (RSTOIC) model has been applied to simulate reactors in which the IL acts as catalyst or solvent, such as CO_2_ conversion,^60,66,78^*n*-butyl acetate manufacture,^170^ or 2,5-furandicarboxylic acid production,^173^ because it is suitable for modeling a reactor if only the stoichiometry and molar extent or conversion are known for the reactions. The RSTOIC model handles reactions that occur simultaneously or sequentially. The equilibrium reactor (REQUIL) model, on the other hand, can be used to simulate reactors when the reaction stoichiometry is known, and the reaction reaches chemical equilibrium. Because the REQUIL model calculates the simultaneous phase and chemical equilibrium for given operating conditions, it has been employed to determine the formation enthalpy of the IL that fits the enthalpy for the reaction between the IL and CO_2_.^36^

Secondary blocks for the conditioning temperature and pressure are common in IL-based processes. Aspen Plus has models for heat-exchange operations. The HEATER model is the simplest and a one-sided heat exchanger that is adequate for simulating thermal and phase changes in the heat exchange for either heaters or coolers and enables the computation of the utility consumption. Therefore, the HEATER model has been used for easily calculating the heating and cooling duties in IL-involved heat-exchange operations^53,59,132^ and the utility-associated consumption and cost and even lets the Aspen Process Economic Analyzer (APEA) estimate a preliminary cost for the exchanger.^37,167^ In contrast, HEATX is the most complete and versatile heat-exchanger model available in Aspen Plus. HEATX is a two-sided heat exchanger for modeling a wide variety of shell and tube heat exchangers, including cocurrent or countercurrent heat exchangers. HEATX has been used to represent the heat transfer between hot and cold IL-containing streams^35,43,46,53,77^ and for integrating energy, i.e., heat recovery, and running simplified shortcut rating calculations, i.e., heat- and material-balance calculations only. For rigorous heat-transfer and pressure-drop computations, the user must supply the exchanger geometry. HEATX also enables design calculations, mechanical-vibration analyses, and the determination of fouling factors. To perform these calculations, the HEATX model is based on a rigorous heat-exchanger program named “Aspen Exchanger Design and Rating,” (EDR). However, this kind of analysis has not been addressed yet for IL heat-exchange operations.

A PUMP model is also available for simulating the pressurization of IL-containing streams. In that regard, the PIPELINE model implemented in Aspen Plus has been applied to compute the discharge pressure required for overcoming column pressure drops and the column height for pumping ILs under given operating conditions, including the pipe length, diameter, roughness, and angle.^37^

Notably, according to the literature, many authors do not use Aspen Plus for process simulations and, instead, replace the previously described Aspen Plus unit models using alternative software (as gPROMS) or mathematical models comprising equations that describe the corresponding operation.^8,117−119,126^

### 3.5 Process Modeling and Simulations

Once the operation unit models have been selected and defined, the complete process can be designed and simulated, as per Scheme 1. Each unit model/operation can be studied before simulating the complete process.^36^ For absorption or distillation separation units, for example, few alternatives are available for designing and sizing rate-based operations. One alternative is the establishment of the purity or recovery,^37^ and another is fixing the column dimensions;^35,43^ usually a combination of both is used.^36^ The design can be addressed through sensitivity analyses, in which the user screens the design variables to study their influence on the desired separation,^68,131^ or the use of a design specification, which is an Aspen Plus-implemented tool that determines the value of a design variable to satisfy the desired value for another variable, such as the product purity or recovery.^60^

Then, the process is simulated by interconnecting the models in each operation unit, when several factors may affect the process design. Closing the recirculation of the process stream is important because it could affect the simulation results, as Hospital-Benito et al. demonstrated for CO_2_ capture processes.^36^ In this sense, defining the right tear stream and calculating the make-ups that solve the material balance are crucial for convergence. The inclusion of the process utilities is also relevant. In Aspen Plus, some utilities comprising cooling water, different types of steam, etc. are defined by default. Users can even vary the inlet and outlet conditions, price, and associated CO_2_ emissions of the utility. Owing to the thermal stability of ILs, the energy demand associated with operating under vacuum is an important aspect for computing the utility consumption in the IL regeneration stage. Navarro et al.^45^ and Hospital-Benito et al.^36,37^ used the Aspen Plus compressor model that emulates the drop from vacuum pressure (<1 bar) to atmospheric pressure in the vapor stream to calculate the energy required to operate under vacuum in stripping columns. Energy integration is another critical aspect to investigate for improving the robustness of the design and energy consumption results. In this sense, Aspen Plus integrates an energy-saving tool that finds design changes to reduce energy consumption and perform modifications automatically by adding or relocating heat exchanges, if accepted. Although many studies have included energy integration,^134,169^ the rigorous design and sizing of IL-involved heat exchangers has not been properly investigated yet.

Process design and simulation usually aim to understand the IL behavior at the process scale and improve the process performance by enhancing either the IL properties or operating conditions. The process performance is assessed based on monetized and nonmonetized KPIs.^8^ Typical KPIs include the IL consumption, energy demand, equipment size, and costs (CAPEX, OPEX, and TAC). The sensitivities of these KPIs have been analyzed to evaluate the adequacy of the process design. IL properties and, thus, KPIs have been demonstrated as being primarily important for process designs.^8,73^ For example, the IL’s thermal stability determines the temperature at which the IL can be regenerated.^36^ Therefore, an effective engineering strategy is to optimize the operating conditions for minimizing the solvent and utility consumptions, equipment size, and, hence, CAPEX and OPEX, which comprise the TAC. Consequently, technoeconomic and environmental analyses and process optimization are recommended for assessing the feasibility of the proposed process compared to current technologies.

The evaluation, optimization, and comparison of the KPIs obtained for both the IL-based process and current technology are the last steps for completing a rigorous study of the simulations. The evaluation and comparison of the technoeconomic results obtained using process simulations withother alternatives or benchmark technologies has been widely addressed in the literature for multiple IL-involved chemical processes, as will be discussed in detail in the following sections.

In addition to KPIs, such as solvent or energy demands, which are process simulation results that can be directly evaluated and compared after they are executed, Aspen process simulators can be further used for calculating the CO_2_-equivalent emissions from the utilities used to supply the energy demand and estimating the costs, but also for cost estimations including capital and operating costs. Regarding environmental concerns, Hernández et al. applied the carbon-tracking tool from the Aspen Plus utility to compute the CO_2_ emissions associated with electricity and LP steam and used the CO_2_-emission factor from US-EPA Rule-E9-571.^60^

Regarding economics, the APEA tool has enabled the estimation of OPEX and CAPEX. In the most complete factorial methods for estimating costs, CAPEX is divided into capital direct and indirect costs. The former computes not only the cost of the purchased equipment but also the piping, civil, structural steel, instrumentation, electrical, insulation, paint, and manpower costs associated with the in-plant installation, whereas the latter accounts for the engineering or contingency costs. OPEX, on the other hand, comprises the cost of utilities, or variable operating costs, and some fixed operating costs, including maintenance, supervision, operating labor, operating charges, plant overhead, and administrative expenses. Finally, the TAC is calculated as the sum of the annualized OPEX and CAPEX and often uses a capital recovery factor^37,167,174^ or another factor that annualizes CAPEX and addresses the return on investment, tax, depreciation, and maintenance.^117,118,126^ García-Gutierrez et al.,^167^ Akinola et al.,^174^ and Hospital-Benito et al.^37^ detailed the procedure for estimating the costs of IL-based CO_2_ capture processes using APEA to determine the equipment cost. García-Gutierrez et al.^167^ and Akinola et al.^174^ used a factorial method for assessing costs based on a percentage of the equipment cost, while Hospital-Benito et al. used APEA rather than a percentage of the equipment cost for estimating all the capital direct cost and later employed the factorial method to compute the indirect costs.^37^

Nevertheless, other approaches for estimating costs based on process simulation results are common in the literature. Huang et al.,^175^ for example, similarly applied a factorial method for estimating costs based on a percentage of the equipment cost but obtained the equipment costs for the columns, heat exchangers, and pumps according to the NETL report. Alternatively, Xie et al.^133^ used specific equations for calculating the cost of each piece of equipment for their factorial method for a biogas upgrading process developed in Aspen Plus. Mota-Martínez et al.,^8^ Seo et al.,^117,118,126^ and Ashkanani et al.^154^ also based the CAPEX calculation per unit on correlations that link the cost to the key properties of the equipment used for CO_2_ capture processes. In any case, rigorous rate-based simulations coupled with detailed cost models for process equipment (APEA or specific equations) is a robust approach for costing IL-based processes in detail. In contrast, simple economic models do not allow for detailed economic analyses, but they are rather useful for identifying the cost range, the trends between costs and the key cost components. As example of the latter, Hospital-Benito et al. identified the major contributors to the total cost of IL-based direct air capture (DAC) processes through a simplified economic model that computed the total cost by simply considering the cost of utilities and an assumed air contactor cost range.^68^

The IL price and decision of whether it is computed as OPEX or CAPEX are the keys for estimating the cost of IL-based processes. Although in the literature, multiple different price scenarios have been considered,^37,176^ prices of approximately 10–50 $/kg, corresponding to scaled-up IL productions, have been the most popular assumption. It agrees with the actual prices of standard ILs supplied at a larger scale (>150 kg) by Proionic.^121^ If ILs can be well or completely regenerated, the IL cost is assumed as CAPEX.^35,167^ In other cases, although the initial IL investment is considered as CAPEX, the presumed IL replacements over time are computed as OPEX.^8,37^ The IL capital investment can be calculated by multiplying the IL price by the IL circulation^174^ or by the IL hold up in the process instead.^35,37^ The operating cost associated with the IL amount that is annually replaced is computed as a percentage (∼10%) of the previously calculated IL capital investment.^8,37^

In addition, process simulation results can be the input for rigorous LCAs.^48,77,177^ This approach can easily extend the large-scale assessment of promising ILs solvents to environmental sustainability. Cuellar-Franca et al.^177^ used the Aspen Plus-supported process design developed by Shiflett et al.^142^ to assess the environmental sustainability of [bmim][MeCOO] applied in power plants that had carbon capture and storage (CCS). Hernández et al.^48^ used diverse environmental indicators based on mass and energy balances calculated using Aspen Plus simulations to evaluate and compare IL-based CO_2_ conversion processes. Some relevant factors should be considered for conducting LCAs. First, a “cradle-to-gate” life-cycle model, which is the most popular option, should be chosen, and system boundaries must be properly defined. The combination of the process simulation and LCA methodology has enabled the estimation of the environmental impacts of [bmim][MeCOO]-based CCS both by considering only the CCS stage and its inclusion in the entire H_2_ production plant.^77^ This implied different system boundaries, which, therefore, varied the environmental impact values. Thus, to compare systems, they must have the same boundaries and include equal assumptions (utilities, chemical consumption, waste treatment, etc.). In that regard, considering the amount of IL that will be used or replaced over time is very relevant for assessing how the IL synthesis could affect the process sustainability. However, in addition to the software (SimaPro,^48^ GaBi,^177^ openLCA,^77^ etc.), the database that is applied for the LCA inventory (Ecoinvent is the most used database^48,77,177^) and the impact calculation method are important. With respect to the latter, methods, such as ReCiPe^48,77^ or CML,^177^ have been used to calculate several impact categories, such as the human toxicity, water ecotoxicity, terrestrial acidification, and CO_2_-equivalent emissions that lead to global warming; on the contrary, the IPCC impact assessment only computes the global warming potential.

### 3.6 Process Optimization

Optimizing IL-based processes is crucial to propose economically feasible alternatives to benchmark technology in view of their industrial deployment (see Scheme 1). The first step on the formulation of the optimization problem is defining the design and operational independent variables (e.g., column stages, flow rates and process pressures and temperatures),^136,178−180^ their range of variation and restrictions to determine the optimal process design and operating conditions.

Second, the objective function must be selected. Depending on the optimization criteria the optimization algorithm would search for a maximum or a minimum. Economic variables such as TAC have been widely used as optimization criteria of these systems based on ILs to unlock their economic viability, which is the case of multiple optimization studies for IL-based ammonia gas separation,^136^ CO_2_ capture,^118,119,126^ and extractive distillation processes.^179,181−184^ However, other KPIs as purity and/or recovery of a component (maximization algorithm), environmental impacts or energy and/or IL consumption (minimization algorithms) have been considered too. In that respect, Tian et al.^178^ performed an optimization of the 1,3-butadiene production process using [emim][PF_6_] as an additive that was meant for maximizing the purity, and the recovery of 1,3-butadiene, while minimizing the energy demand. Furthermore, Zhan et al. and Deshpande et al. performed an optimization including not only TAC but also CO_2_ emissions of an IL-based ammonia-containing purification process^136^ and algal biodiesel processes where the IL acts as a catalyst,^171,172^ respectively.

Finally, an optimization method must be chosen. The mathematical model used in most studies is a multiobjective mixed-integer nonlinear^119,185^ or nonlinear numerical problem^178^ for which the optimization method can be global (genetic algorithm,^136,178,183^ simulated annealing^179^) or specific (sequential quadratic programming).^181,182^ Tian et al., Zhan et al., Deshpande et al., Li et. al, and Ma at al. applied a multiobjective genetic algorithm [the nondominated sorting genetic algorithm (NSGA) II] to perform the multiobjective optimization of their 1,3-butadiene production,^178^ ammonia gas separation,^136^ biodiesel,^172^ and extractive distillation^180,184^ processes based on ILs. Similarly, Zhang et al.^179^ minimized the TAC of an IL-based extractive distillation plant by programing a simulated annealing algorithm. In contrast, Li et al.^182^ and Wei et al.^181^ optimized the TAC of IL-based extractive distillation processes by adopting a sequential iterative method.

If Aspen Plus software is used to carry put process simulations, it can be connected to an artificial neural network^178^ or other software as Excel or MATLAB, in which the solution algorithm is programed. ActiveX allows for linking the optimization algorithm in MATLAB with Aspen Plus,^136,179^ whereas an Excel-based program is interfaced with Aspen Plus using the visual basic application.^171,172^ Among many other alternatives, it is worth mentioning gPROMS software has been successfully used to implement both the flowsheet model and the optimization problem for IL-based processes.^171,172^

On the other hand, the use of detailed process flowsheet models with rigorous thermodynamic and mass transfer models entails several computational challenges for solving the nonlinear optimization of the whole process. These complexities can be overcome using a pseudotransient modeling approach, that enhances convergence. The pseudotransient approach was developed by Seo et al.^117^ and it has been demonstrated to be a useful tool for both modeling and optimizing IL-based CO_2_ capture systems. Instead, Seo et al.^118^ also employed stochastic programming for the optimization of adaptable IL-based CO_2_ capture processes integrated into power plants.

Moreover, in the case of IL-based processes, the selection of an adequate IL and the design and operating conditions of the process are closely related to each other. Therefore, simultaneously optimizing the IL molecular structure and the corresponding process design has gained attention to enhance the performance (e.g., energy demand, economics) of the IL-based system. Valencia-Marquez et al.^185^ and Zhang et al.^119^ successfully applied systematic multiscale design methods, the latter using surrogate modeling,^119^ to CO_2_ capture using ILs with physical absorption aiming at maximizing the amount of captured CO_2_ and minimizing TAC, respectively.

In summary, multiobjective optimization is key to design new energy-efficient, cost-effective, and even environmentally friendly IL-based processes.

## 4 Key Applications of Ionic Liquids Analyzed Using Process Simulation

### 4.1 Carbon Capture by Physically Absorption

Carbon capture by physically absorbing CO_2_ is one of the most widely evaluated applications of ILs, owing to their promising absorbent properties, such as their structural tunability, negligible vapor pressure, nonflammability, high CO_2_ absorption capacity, and selectivity.^186^ Thousands of fundamental studies have been reported, mainly focused on analyzing the gas solubility of CO_2_ in ILs.^16^ In fact, Henry’s constants and absorption isotherms have been commonly used as the key criteria for selecting ILs to develop physical absorbents that have increased CO_2_ absorption capacity and expected reduced regeneration heat.^187^ To estimate CO_2_ diffusivity in ILs, absorption rates have also been measured to obtain a wide variety of kinetic and thermodynamic behaviors depending on the selected cations and anions. In addition, the relevance of other absorbent properties (such as MW, heat capacity, thermal stability, availability, biodegradability, and toxicity)^5,8,9,187^ has been pointed out.

Therefore, the selection of ILs that have the most promising properties for physical CO_2_ absorption processes has become a challenging task in fundamental studies.^12,188^ Comparatively few process simulations have been analyzed for initially assessing the performance of ILs in physical CO_2_ absorption at the industrial scale for different carbon-capture systems (postcombustion, precombustion, biogas, etc.). Table 1 lists the main information reported for process simulation studies, including the systems, process modeling details, specified and studied variables of the main operations (absorption and regeneration stages), and evaluated KPIs.

Despite the limited number of reported studies, physical CO_2_ absorption by ILs is a paradigmatic case of how process simulation can contribute to the knowledge and development of the IL application field.^12−15^ This section summarizes the relevant contributions of the scientific community for identifying the key IL properties that determine the process performance and designing the main operational units (absorber and regeneration stages) and process configuration to enhance the carbon capture while minimizing the chemical and energy consumptions, process costs, and environmental impacts.

**Scheme 3:**
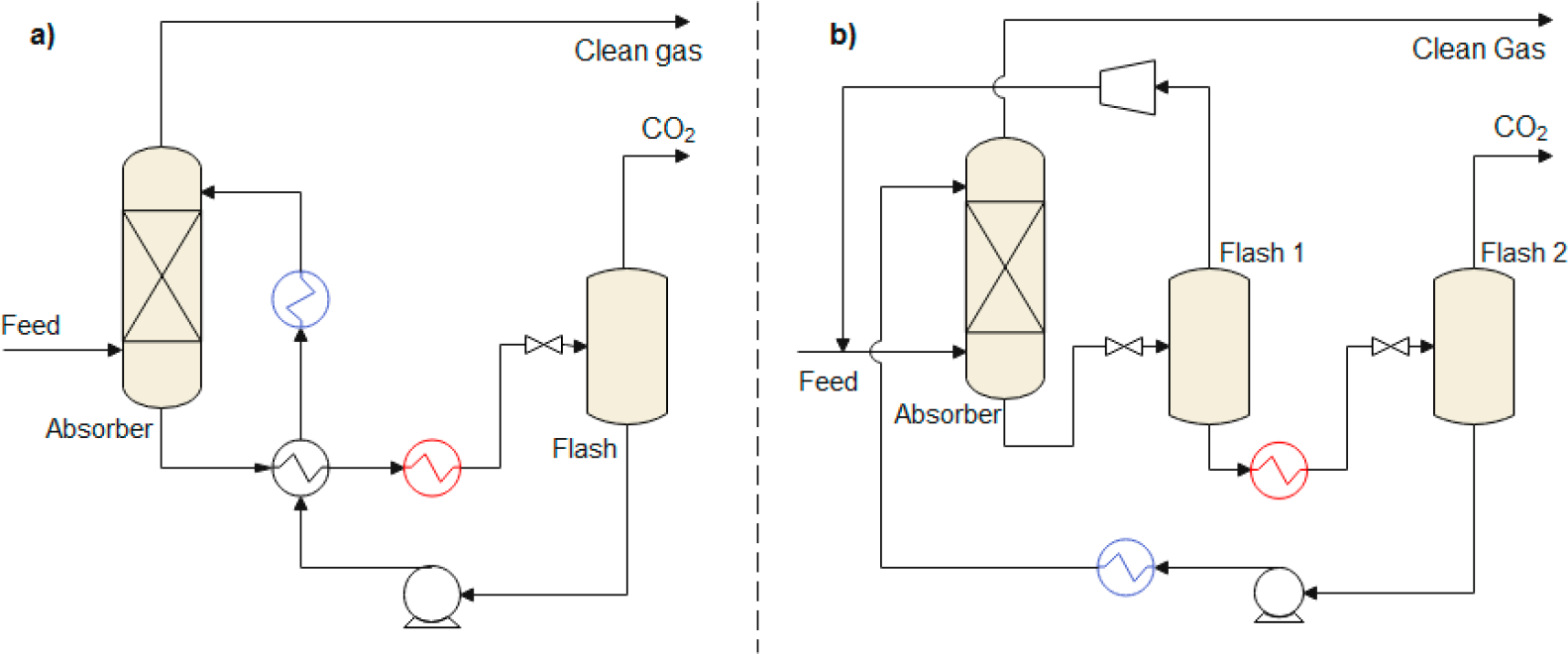
Typical Flowsheet for Complete IL-Based Physical Capture of CO_2_

Process simulations have also been used to evaluate the technoeconomic feasibility and sustainability of IL-based physical CO_2_ absorption processes compared with the current industrial carbon-capture technologies. Scheme 3 shows a representative flowsheet used to model carbon capture by physical absorption using ILs. First, the inlet gas is fed to the bottom of the main absorption tower and countercurrently contacts the IL stream to promote the physical absorption of CO_2_. Different carbon-capture systems have been evaluated from precombustion to biogas to postcombustion, mainly by modifying the inlet stream’s CO_2_ partial pressure. The absorber has been commonly designed using rigorous packing column models available in commercial process simulators and equilibrium or rate-based modes to evaluate the roles of thermodynamics and kinetics in CO_2_ capture efficiency. Because physical absorption usually operates at high pressures to reach relevant CO_2_ recoveries, an initial section can be included to condition the pressure (and/or temperature) of the inlet gas stream before it enters the absorber. The clean gas exits the top of the absorption column, and the CO_2_–IL stream is exhausted to the regeneration section.

Different separation approaches have been proposed to recover and recycle the IL absorbent and produce a CO_2_ stream. They involve one or more flash separators (depending on the specified quality of IL- and CO_2_-rich streams and the desired recovery of the important components of the inlet gas stream, such as CH_4_ or H_2_), which operate at higher temperatures under vacuum or by combining temperature and vacuum swing processes. The recycled streams (regenerated IL and, in some cases, gas components other than CO_2_) are conveniently conditioned (cooled and pumped under absorber operating conditions). Commonly studied design variables have included the IL structure (which determines the absorption thermodynamics and kinetics), operating temperatures and pressures of the absorption and regeneration stages, gas–liquid contactor type (packed or tray), and process configuration (regeneration units, recycles, conditioning operations, energetic integration, etc.).

To evaluate common KPIs, technoeconomic analyses have been performed for several parameters, including the absorbent flow; equipment sizes; energy demand; operating, capital, and total process costs; and CO_2_ equivalent emissions. In most studies, the Aspen Plus commercial simulator was used for designing and modeling processes, and different predictive or regressive thermodynamic models (COSMO-SAC/RS, UNIFAC, NRTL-RK, and PR-RSK) (Table 1) have been applied. The process costs were estimated using APEA or a homemade traditional-method-based approach for estimating costs. For additional KPIs, the LCA method was used to evaluate the environmental impacts of carbon-capture technology based on IL-based physical CO_2_ absorption.

#### 4.1.1 Ionic Liquid Performance in CO_2_ Physical Absorption Unit

One of the first strategies employed in process simulation studies was the use of ILs that had high CO_2_ absorption capacities and selectivities for evaluating IL-based carbon capture.^89,189,198,199^ These studies screened several IL candidates by bibliographic analysis,^199^ experimental measurements,^127,194,198−200^ and/or computational predictions (COSMO-RS or UNIFAC methods)^89,189,199^ to select promising CO_2_ physical absorbents that satisfied these thermodynamic criteria. Thus, most of the first steady-stage process simulation studies selected NTf_2_-based ILs to evaluate their process performance in physical CO_2_ absorption, operating at elevated pressures and mild temperatures (5–120 bar and 25 °C–60 °C). Liu et al.^189^ performed process simulations using [bmim][NTf_2_] to evaluate a new IL-based decarbonization technology for treating shale gas with 7 mol % CO_2_ in an absorption column (at 60 bar and 20 °C) using an equilibrium-based design model and the NRTL-RK thermodynamic model in Aspen Plus. The IL-rich liquid stream was fed to a multistage flash operation to regenerate the IL absorbent and recover the CO_2_ and other absorbed components of shale gas. The carbon-capture process was designed to obtain purified gas with less than 3% CO_2_. Xie at al.^199^ evaluated three imidazolium-based ILs ([hmim][NTf_2_], [bmim][NTf_2_], and [bmim][PF_6_]) for biogas upgrading with 45 mol % CO_2_ using steady-stage process simulations and the NRTL-RK thermodynamic model in the Aspen Plus simulator, with a flowsheet, including an absorption column (20 °C, 8 bar, and a product purity of 97 mol % CH_4_), two flash separators (3 and 0.2 bar) for IL (98.5% pure) and CH_4_ recovery and recycling, and heat exchangers and compressor pumps for gas/liquid stream conditioning. The simulation results showed that the required solvent and total energy consumptions followed the trend [bmim][NTf_2_] < [bmim][PF_6_] < [hmim][NTf_2_] related to the ILs CO_2_ absorption capacity and CO_2_/CH_4_ selectivity. Therefore, the first steady-stage process simulation studies concluded that ILs could be conveniently selected based on their CO_2_ absorption capacity for efficiently developing carbon-capture processes for different systems (from shale gas to biogas and postcombustion).

**Figure 4:**
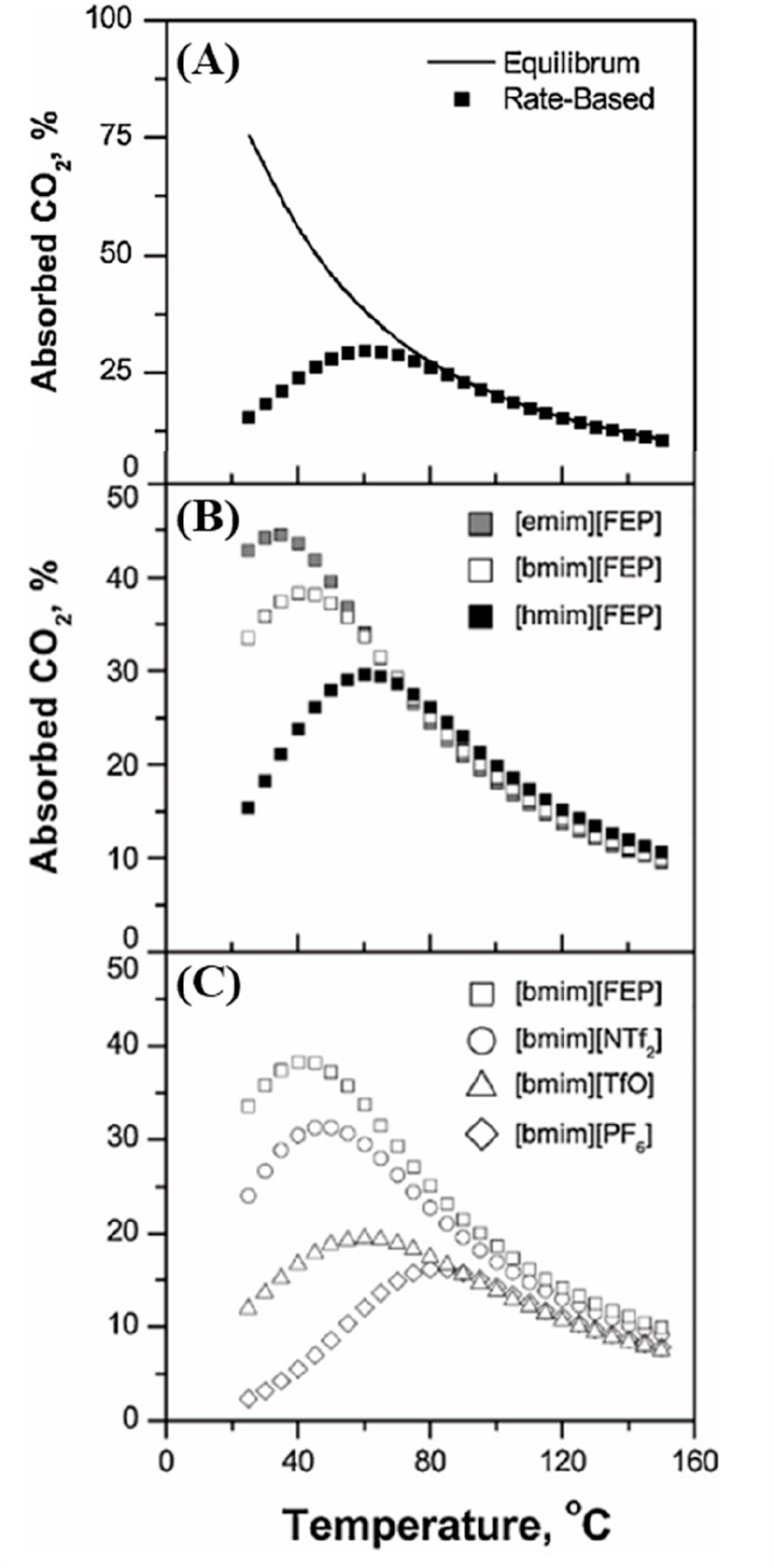
Percentage (%) of CO_2_ captured from 1,000 kmol/h of flue gas (13 mol % CO_2_) plotted as a function of IL inlet temperature using packed absorption column (23.2 m, Flexiring 0.625 in. internals) at constant pressure of 20 bar and L/G ratio of 1 mol/mol when (A) RADFRAC model that represents absorber is in equilibrium and rate-based operating modes and for different (B) cation-based IL series using common anion [NTf_2_] and (C) anion-based IL series using common cation [bmim]. Reproduced from ref (35). Copyright 2017 Elsevier.

**Figure 5:**
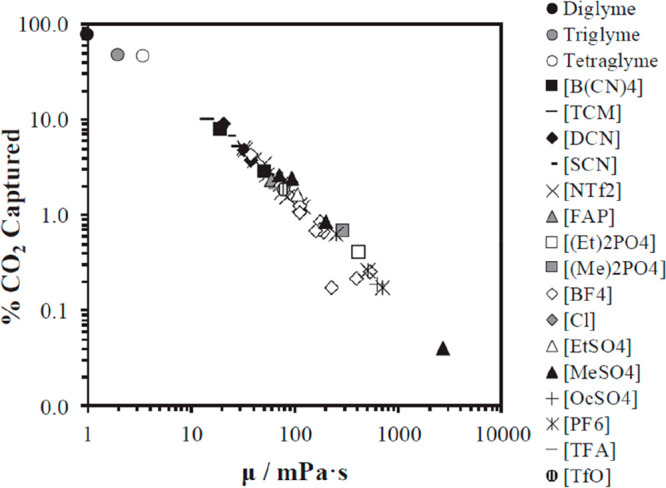
Percentage (%) of CO_2_ absorbedby ILs and glymes from postcombustion flue gas (1,000 kmol/h, 13 mol % CO_2_) in packing absorption column plotted as a function of viscosity, using rate-based RADFRAC model and IL mass flow of 400 ton/h, fractional capacity of 62%, column height of 1 m, and 10 equilibrium stages. Symbols indicate glyme or anion forming IL. Reproduced from ref (50). Copyright 2019 Elsevier.

Further simulation studies have evaluated the mass-transfer role in IL-based physical CO_2_ absorption by modeling rate-based adiabatic columns.^8,35,50,54,72,113^ Basha et al.^113^ developed a conceptual CO_2_-capture process from a multicomponent fuel gas stream with 23.9 mol % CO_2_ using the selected IL [hmim][NTf_2_] by employing a PR EoS and Billet and Schulte’s correlations accounting for the gas–solvent mass transfer to estimate the mass-transfer coefficients and effective gas–liquid interfacial area in packed beds. The process had four parallel adiabatic packed-bed absorbers, three flash drums in series for solvent regeneration, and two pressure/intercooling systems to separate and pressurize the carbon dioxide. The absorber operation was designed using a packed absorption column operating at 30 bar and inlet gas and liquid temperatures of 500 and 25 °C, respectively. These rigorous process simulations indicated the suitability of [hmim][NTf_2_] as a physical absorbent for carbon capture, capable of achieving over 97 mol % of CO_2_ recovery under reasonable absorber operating conditions with a negligible loss of the IL absorbent. García-Gutierrez et al.^167^ applied the COSMO-based/Aspen Plus simulation methodology to evaluate the plant efficiencies and production costs of large-scale CO_2_-capture processes from biogas streams using different [NTf_2_]-based ILs as physical absorbents. The absorption operation in a packed absorption column was described using the RADFRAC model and a rate-based calculation type. The process simulations demonstrated efficient biogas upgrading using ILs to produce biomethane stream with a purity of 95 vol % in all cases, with the solvent requirements decreasing in the order [emim][NTf_2_] > [hmim][NTf_2_] > [P_66614_][NTf_2_] (for L/G = 1, 0.8, and 0.5 mol/mol, respectively), which agreed with the higher experimental CO_2_ absorption capacities of the ILs. A later study by de Riva et al.^35^ thoroughly analyzed the roles of thermodynamics and kinetics in physical CO_2_ absorption by ILs in packed columns for postcombustion systems. Figure 4A compares the CO_2_ recoveries as a function of the IL inlet temperature when the RADFRAC column model of the Aspen Plus simulator was in the equilibrium and rate-based operating modes, using the IL [hmim][FEP] to treat a flue gas stream in a packed absorption column.^35^ Strong kinetic control was observed at typical temperatures (20 °C–60 °C) of the absorber tower, achieving a remarkably lower carbon-capture efficiency than expected based on the CO_2_ absorption capacity of the IL. This effect was attributed to the high viscosity of the ILs, highlighting it as a key selection criterion for this kind of solvent. In contrast, with increasing operating temperature up to typical values (80 °C–120 °C) in the regeneration stage, the absorbed CO_2_ in the IL was determined by the thermodynamics, as the IL viscosity decreased. Figure 4B,C shows the percentage of CO_2_ absorbed under the same process conditions using other ILs with a different cation or anion, revealing similar mass-transfer limitations in the absorption column for all cases. A higher CO_2_ capture was achieved using ILs with shorter alkyl chains on the imidazolium cation owing to their lower viscosity. In addition, the [FEP] anion-based IL had the lowest viscosity and presented the highest degree of CO_2_ capture, followed by [NTf_2_], [TFO], and [PF_6_], corresponding to the IL viscosity trend. With this criterion in mind, in a later study, the ILs evaluated in physical CO_2_ absorption using rate-based column models for postcombustion systems were extended to ILs with favorable transport properties, that is, low viscosity. Figure 5 shows a linear relationship between the CO_2_ recovery and absorbent viscosity for 50 ILs with remarkably different viscosities to treat postcombustion flue gas in a packed column at an L/G ratio of 1 mol/mol, gas inlet temperature of 25 °C, and pressure of 20.3 bar.^50^ The wide viscosity range of the absorbents led to the conclusion that postcombustion CO_2_ capture by physical absorption with ILs was kinetically controlled and mainly determined by the rates of gas–liquid absorption under common operating conditions in commercial packed columns and that the most promising ILs for physical CO_2_ absorption were [emim][TCM], [emim][DCN], and [emim][SCN]. This validated that low viscosity was a more suitable criterion for selecting IL absorbents than high CO_2_ solubility, in contrast to findings commonly reported in literature.^187^ Importantly, when using three glymes (benchmark industrial absorbents) under the same operating conditions, a remarkably higher carbon-capture efficiency was obtained (Figure 5). This was attributed to the lower viscosity of the organic solvents, even when certain ILs, such as [emim][NTf_2_], presented higher CO_2_ solubilities than glyme solvents per unit of mass. Therefore, rate-based process simulations revealed that ILs did not seem to present better absorbent properties in the CO_2_ absorption stage for postcombustion carbon capture than traditional organic solvents, such as glymes (components of the Selexol commercial process). However, the need for further process studies must be emphasized to evaluate the role of the inlet CO_2_ partial pressure (postcombustion, biogas, precombustion, and natural gas purification systems) in the kinetic control of the absorption stage and complete the process analysis by considering the regeneration stage, life-cycle analysis, etc.

**Figure 6:**
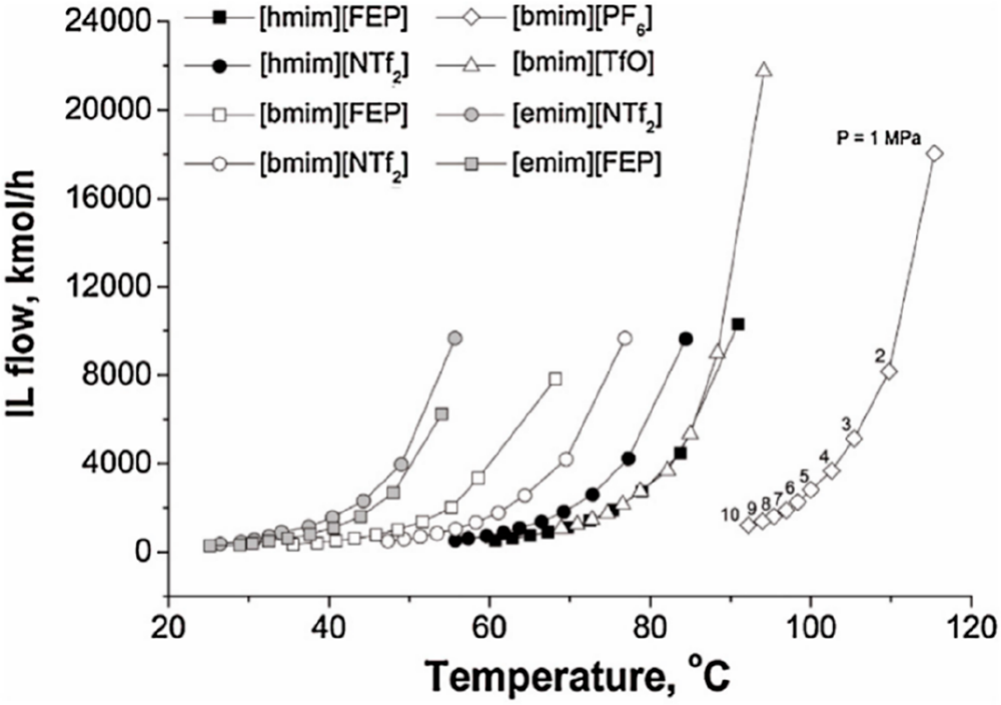
IL required to reach 90% of CO_2_ absorbed in rate-based RADFRAC column operating at different temperatures and pressures ranging from 10 to 100 bar for 1,000 kmol/h of flue gas (13 mol % CO_2_). Inlet temperature of IL is optimized for each point on the graph. Reproduced from ref (35). Copyright 2017 Elsevier.

Process simulations have been used to analyze the sensitivities of several design variables of the physical CO_2_ absorption unit. Thus, Figure 6 shows the absorbent flow required to achieve 90% CO_2_ absorption when treating a postcombustion flue gas stream in a packed absorption column using different ILs, as simulated using the RADFRAC rate-based column model in Aspen Plus.^35^ The points of each IL curve represent different absorber operating pressures (10–100 bar) and temperatures (25 °C–120 °C). Increasing the absorber operating pressure implied reducing both the amount of IL required to reach the desired separation and the temperature at which the maximum CO_2_ amount (Figure 4) was absorbed. The most favorable ILs, [emim][NTf_2_] and [emim][FEP], required lower molar flows and temperatures to achieve the same separation degree, which was ascribed to the combined effect of their high CO_2_ absorption capacities and CO_2_ diffusion coefficients in these ILs.

#### 4.1.2 Complete Carbon Capture Process Modeling for Technoeconomical Analysis

After individually analyzing the main carbon-capture operation (i.e., the absorption column), the complete carbon-capture process could be modeled. This involved designing the IL regeneration section and incorporating the recycling, heat exchangers, compressors, pumps, etc., required to complete the flowsheet of the carbon-capture process. These process simulations enabled the technoeconomic analysis to evaluate the influences of the IL properties, design variables, and process configuration on the KPIs of the carbon-capture process by IL-based physical CO_2_ absorption.

**Figure 7:**
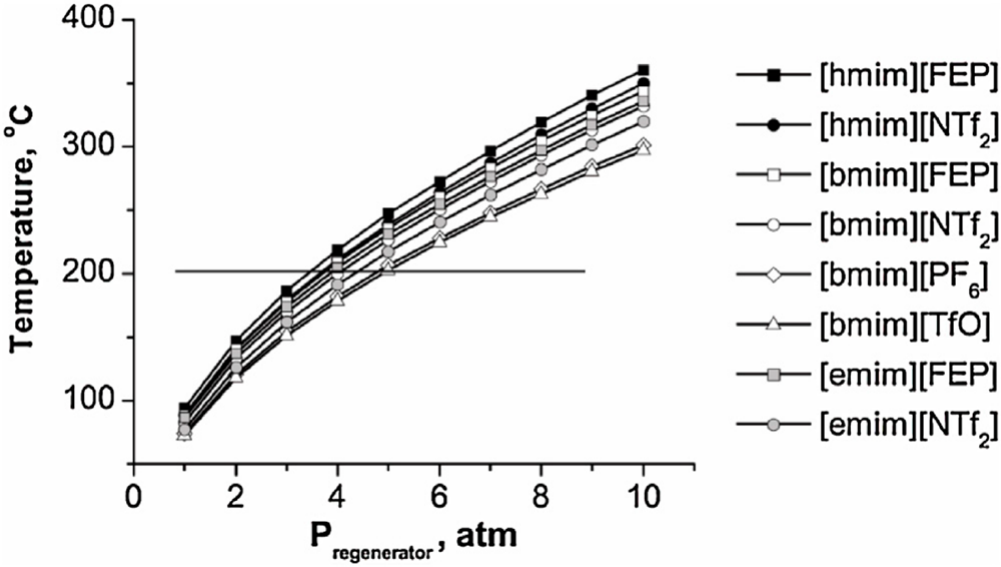
Temperatures required to recover 90 mol % ILs at different decompression pressures in flash regeneration stage. Horizontal line represents reference maximum operating temperature to avoid thermally decomposing ILs. Mixture containing 10 mol % CO_2_ and 90 mol % IL (flowing at 1,000 kmol/h) at 50 bar and 50 °C in all cases. Reproduced from ref (35). Copyright 2017. Elsevier.

Different alternatives for IL regeneration have been analyzed using process simulations to exploit the negligible vapor pressure of the IL absorbents under operating process conditions. In the first approach, an adiabatic flash evaporator was proposed for the regeneration stage, where the IL solution pressure was reduced (or the temperature was increased). This process yielded the regenerated the IL at the bottom, which could be recirculated to the absorption column, and generated a CO_2_-rich gas stream at the top.^35,167,196,198^ Xie et al.^198^ performed a simulation analysis for CO_2_ separation processes using an imidazolium-based IL as the absorbent and a pressure-swing and/or temperature-swing flash unit for the IL regeneration stage. By fixing the inlet exhaust IL stream at 25 °C and 10 bar, the solvent was efficiently regenerated by decreasing the pressure to 1 bar at the same temperature or by desorption at 50 °C and 10 bar. Gutierrez et al.^167^ applied a one-stage pressure swing solvent regeneration option whereby physical absorption was conducted at a high pressure (30 bar), whereas the IL (gas desorption) was regenerated under vacuum (0.01 bar) in an adiabatic flash evaporator. de Riva et al.^35^ systematically analyzed the regeneration of CO_2_-exhaust IL using a combined pressure- and temperature-swing technology in an individual flash unit. The liquid stream inlet to the swing operation presented a molar composition of 10% CO_2_ at 50 bar and 50 °C. The combined flash temperature and pressure required to regenerate the 99 mol % IL was evaluated in a wide range of operating conditions (50 °C–400 °C and 1–10 bar) for different ILs (Figure 7). The authors concluded that the regeneration process could be performed at very moderate temperatures (72 °C–94 °C) when decompressing the system to 1 bar.^35^ Therefore, from an energetic and, consequently, economic perspective, the advantage of ILs with respect to amines (∼120 °C) is that the regeneration occurred at lower temperatures, i.e., between 50 and 90 °C, which enabled the technical use of waste heat from the power plant.^167^ Decompression to higher pressures implied strongly increasing the regeneration temperature above the set maximum operating temperature (200 °C) at flash pressures above 5 bar for all the evaluated ILs. From this individual flash unit analysis, [emim][NTf_2_] was presented as the most adequate IL absorbent owing to both its low solvent consumption in the absorber stage and the mild flash temperature required for its efficient regeneration.^35^ These process simulations indicated the suitability of using a simple flash separator for efficiently regenerating the exhausted IL in the physical CO_2_ absorption-based carbon-capture process, revealing that a combination of pressure and temperature swings was an energetically and economically feasible option.

Alternatively, multistage flash separation systems with two, three, or more units operating at different pressures and/or temperatures have been proposed for the IL regeneration stage to ensure the desired CO_2_ stream purity (for example, suitable for transport and storage) and the recovery of the other relevant absorbed gas components (CH_4_, H_2_, etc.) from the IL-rich stream.^8,89,113,127,189−191,193,194,199−203^ Thus, in the previously cited study by Basha et al.,^113^ the IL-rich stream (at 30 bar) was regenerated using the pressure-swing option with three adiabatic flashes operating at 20, 10, and 1 bar. This flash train enabled the efficient regeneration of the exhausted IL and the separation of the absorbed gases from the IL and generated a CO_2_-rich gas stream. Further flash and compression operations were included in the process flowsheet for conveniently separating the components (CO_2_, H_2_, and H_2_O) from the gas stream. Liu et al.^189^ used different regeneration configurations, comprising either two or four flash separators. In the first configuration, the IL-rich solvent (60 bar and 20 °C) was directed to the first flash unit (15 bar) to recycle the light hydrocarbons; then, in the second flash unit, the CO_2_ was released at a slightly increased temperature (27 °C) and reduced pressure (1 bar). The second regeneration approach comprised a reduction in the pressure of the IL-rich solvent stream in a series of multistage flash vessels (55, 40, 30, and 0.4 bar) to consume less energy than the two-stage configuration at a fixed separation temperature. Xie et al.^199^ used two-stage separation for recovering CH_4_ and IL in a [bmim][NTf_2_]-based biogas upgrading process by decreasing the pressure of the IL-rich stream from 8 bar (absorber) to 3 bar (first flash)—to recover the absorbed CH_4_—to 0.2 bar (second flash)—to regenerate the IL (98.5% purity) at 20 °C. The sensitivity analysis showed that the CH_4_ yield and CO_2_ removal efficiency increased while increasing the pressure from 1 bar to the optimal pressure of 3 bar. Similarly, other authors employed a two-flash-based IL regeneration section, where the operating pressure of the first and second flashes (from 55 to 0.01 bar) was determined by the initial pressure of the IL-rich stream and the desired purity of the IL or CO_2_ stream. Another study was performed by Zubeir et al.^190^ for sweetening synthetic natural gas and producing a liquefied CO_2_-enriched stream using a low-viscosity IL [hmim][TCM] versus the established physical absorbent Selexol (DEPG, a mixture of dimethyl ethers of polyethylene glycol), used as a benchmark. In addition to the commonly used pressure-swing process configuration, different configurations for the IL regeneration were evaluated by combining temperature and pressure swings. The results revealed that by reducing the pressure of the absorber (from 28 to 9.2 bar at 20 and 320 °C, respectively) in the lowest-pressure flash tank, the recompression costs were substantially reduced, which emphasized that ensuring the thermal stability of the ILs was crucial for preventing their degradation during the regeneration process. As an additional contribution, Amiri et al.^204^ simulated the physical CO_2_ absorption process from a feed gas containing methane and CO_2_ using the [hmim][TCB] IL for treating an inlet gas feed with CO_2_ concentrations between 5 and 30 mol % and using from two to four flash units in the IL regeneration stage. When the feed gas mixture was highly concentrated in CO_2_, a high-purity solvent was regenerated by reducing the pressure using flash drums, whereas when the CO_2_ concentration was below 5 mol %, the pressure–temperature swing absorption–regeneration process was preferred.

**Figure 8:**
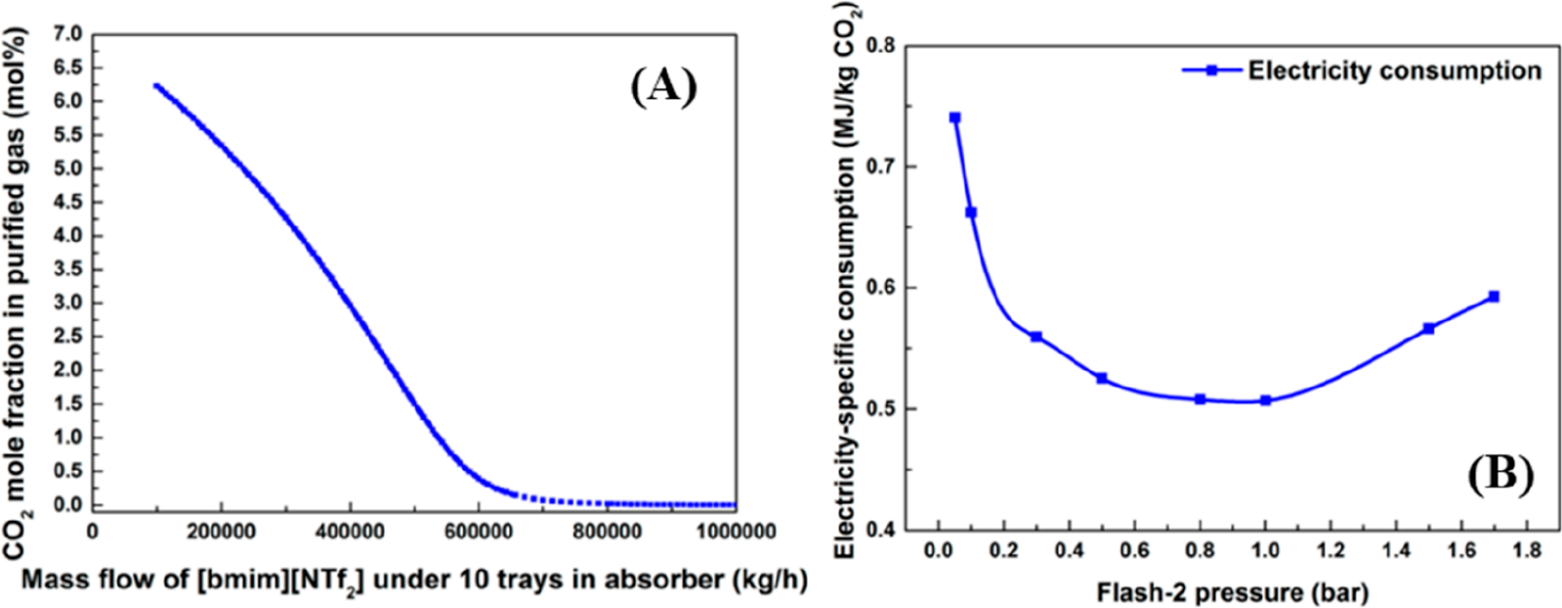
(A) Molar fraction of CO_2_ flowing out of absorber versus mass flow of ILs for 10 trays and (B) electricity consumption at different flash-2 pressures. Flue gas of2,000 kmol/h containing 7 mol % CO_2_ at 60 bar and 227 °C was used in all cases. Reproduced from ref (189). Copyright 2016 ACS.

**Figure 9:**
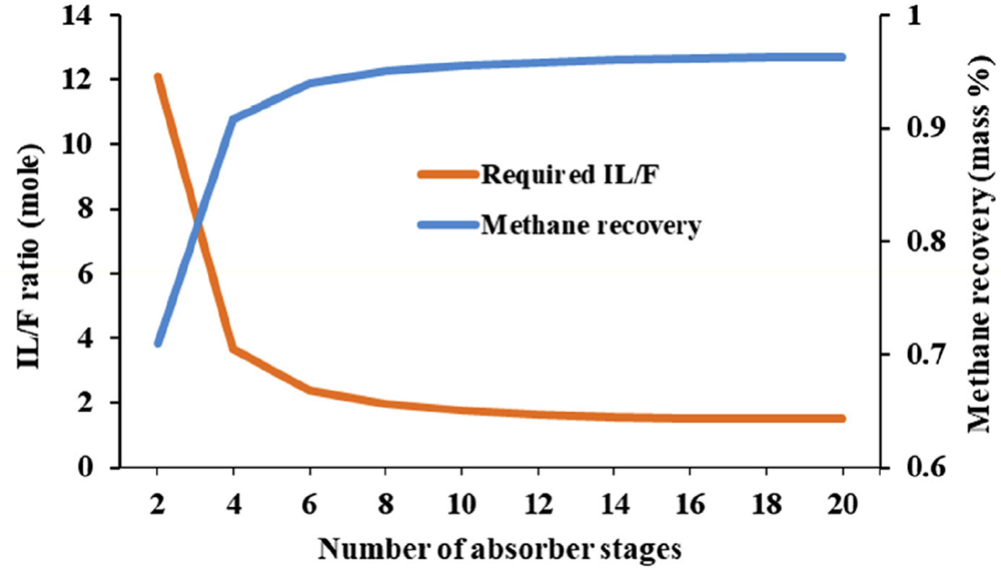
Simultaneous effects of number of absorption stages and IL/F ratio on biomethane recovery during treatment of biogas flowing at 2,851 kmol/h using [bmim][PF_6_] flowing at 4,394 kmol/h, 30 °C, and 40 bar. Reproduced from ref (200). Copyright 2019 Elsevier.

By focusing on the complete carbon-capture process, sensitivity analyses have usually been applied as a preliminary approach to establish the operating condition ranges and compare different systems. In the steady-stage design of the carbon-capture process by Liu et al.,^189^ the expected relationship between the [bmim][NTf_2_] flow rate and CO_2_ concentration was obtained for the purified gas (Figure 8A). In addition, the optimized pressure in the second flash of the IL regeneration section minimized the electricity consumption (Figure 8B). In the previously described study by Xie et al.,^199^ which focused on the equilibrium-based design of a biogas upgrading process using [bmim][NTf_2_], the CH_4_ yield and CO_2_ removal efficiency increased with increasing absorber pressure and decreased with increasing absorber temperature. Consequently, for 97% pure CH_4_, the amount of the recirculated solvent increased with increasing temperature and decreasing pressure in the absorber, which was consistent with the findings of later steady-stage process simulations of IL physical-absorbent-based carbon capture.^191,201^ Haider et al.^200^ developed a biogas upgrading process based on biomethane (99 mol % purity) liquefaction using the [Bmim][PF_6_] IL by simulating steady-state processes with the PR-SRK thermodynamic model in Aspen Plus software. The sensitivities of the operating temperature, pressure, number of stages, and IL flow were analyzed to design the absorber unit with a high CH_4_ recovery rate (Figure 9) by optimizing the number of stages at 14 and a low IL/F ratio of 1.57 at 40 bar and 30 °C.

**Figure 10:**
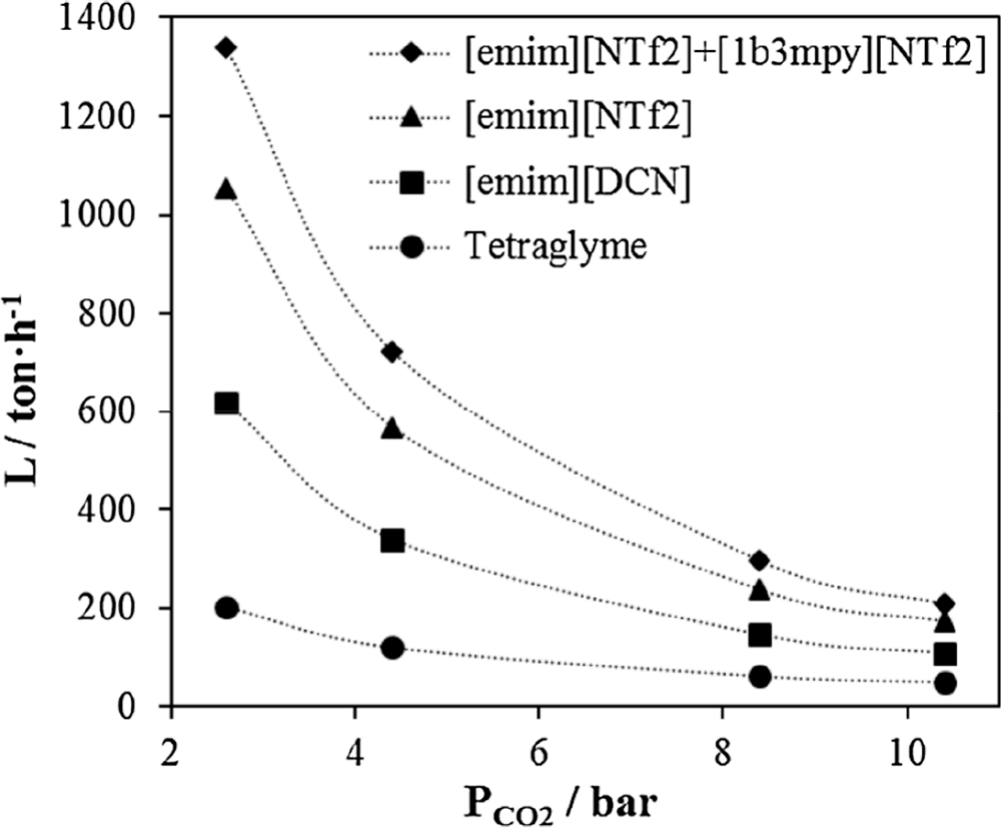
Solvent flow required to capture 90% CO_2_ from flue gas (1,000 kmol/h) plotted as a function of CO_2_ partial pressure in gas feed, as calculated using rate-based RADFRAC model at 25 °C and 20 atm. Reproduced from ref (50). Copyright 2019 Elsevier.

A rate-based process was rigorously analyzed by Palomar et al.^50^ for treating a realistic flue gas stream (flowing at 1,000 kmol/h with a molar composition of 13.4% CO_2_) by evaluating the solvent mass flow required to recover 90% CO_2_ in the absorption packed column at 25 °C and 20 bar (Figure 10) for different IL-based absorbents (including two representative ILs with very favorable thermodynamic or kinetic absorption and an optimized mixture comprising ILs and tetraglyme, a benchmark industrial solvent used in CO_2_ capture).^50^ As expected, the absorbent consumption decreased with increasing CO_2_ partial pressure in the inlet gas stream for all the absorbents (from postcombustion to biogas and precombustion or natural gas purification) according to the most favorable thermodynamics (higher gas solubility) and kinetics (stronger driving force). However, the study concluded that the mass transfer kinetics control the CO_2_ recovery throughout the entire pressure range. This was evidenced by the diminishing solvent requirement, which followed the same order as that of the decreasing absorbent viscosity. In fact, tetraglyme-based physical CO_2_ absorption presented remarkably lower solvent consumptions than any of the IL-based absorbents and mixtures. Notably, from an industrial perspective, the absorbent consumption should be reported in mass units to avoid misunderstandings about the impacts of the MW.^187^

**Figure 11:**
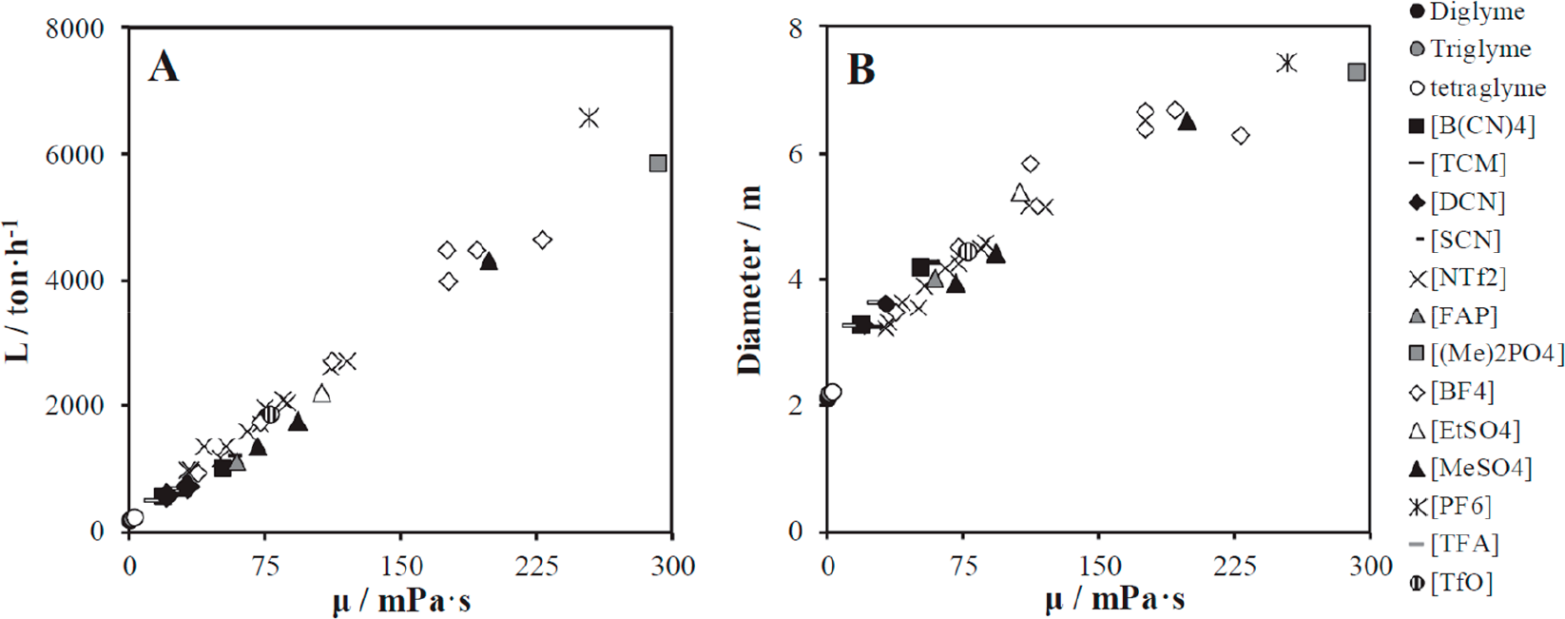
(A) Solvent flow and (B) column diameter required to capture 90% CO_2_ from flue gas (1,000 kmol/h) in postcombustion gas comprising 13.4 mol % CO_2_, N_2_, H_2_O, and O_2_ at 25 °C and 20 bar plotted as a function of viscosity in rate-based RADFRAC model. Symbols indicate glyme or anion forming IL. Reproduced from ref (50). Copyright 2019 Elsevier.

**Figure 12:**
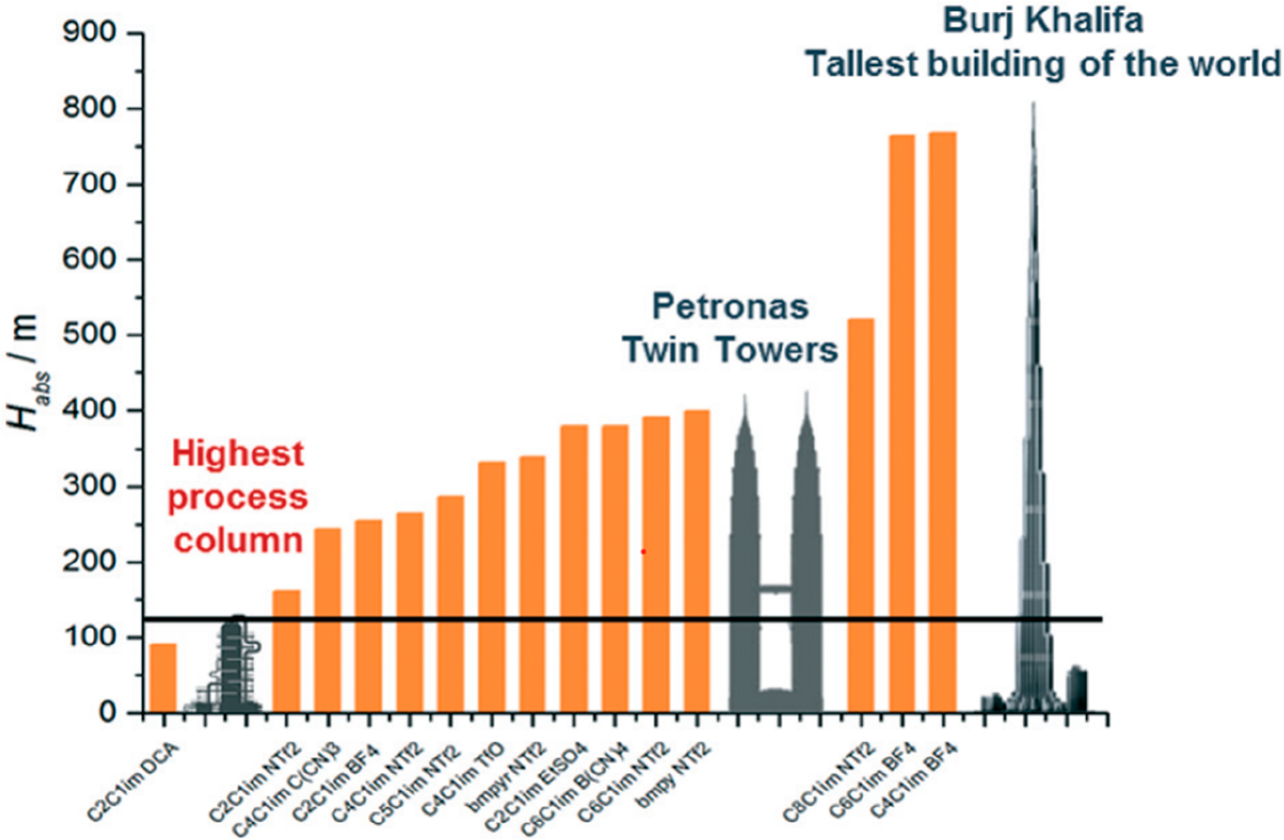
Heights of CO_2_ absorbers required using 15 ILs as solvents at 20 bar and 30 °C for treating flue gas containing 12% (v/v) of CO_2_ and flowing at 3,240 t/h. Absorber heights are compared to heights of tallest process column (121.3 m), Petronas Towers (451.9 m), and Burj Khalifa Tower (828 m). Reproduced from ref (8). Copyright 2018 RSC.

Figure 11 shows the effect of the IL viscosity on the IL mass flow and column size required to absorb 90% CO_2_ using a packed column at 25 °C and 20 bar to treat the flue-gas stream (13.4 mol % CO_2_) indicated in Figure 10.^50^ A clear relationship existed between the IL viscosity and required solvent flow (Figure 11A), confirming that the mass-transfer kinetics of the CO_2_ in the IL was controlling the process.^54^ The [emim][DCN] IL required the lowest consumption because it displayed the lowest viscosity. Using the most-promising cyano-based IL, the required flow was at least double that required for any of the evaluated glymes. The required column diameter (designed for maintaining a fractional capacity of 62%) of the absorption column, on the other hand, decreased for less-viscous absorbents (Figure 11B), which agreed well with previous simulations conducted by Xie et al.^199^ In fact, the column sizes designed using ILs were always larger than those designed using conventional glyme absorbents. In this regard, Mota-Martínez et al.^8^ used rigorous rate-based process simulations to present a visual representation of mass-transfer limitations of the IL-based physical CO_2_ absorption processes. Thus, Figure 12 shows that a high packed column was required to absorb 90% CO_2_ from a flue gas (containing 12% CO_2_) using 15 different ILs operating at 20 bar and 30 °C. Owing to its favorable low viscosity and high CO_2_ absorption capacity (in mass units), the [emim][DCN] IL required the shortest absorption tower. However, the optimal [emim][DCN] IL required a column that was only slightly shorter than the tallest process column in the world (121.3 m), whereas the remaining promising preliminary IL absorbents required column sizes that could not be deployed on an industrial scale, closer to the height of the Petronas Towers (451.9 m) and even the Burj Khalifa Tower (828 m).^8^

In addition to the solvent consumption and equipment size of the absorber (main operation), process-simulation-based technoeconomic analyses provided other relevant KPIs for evaluating the feasibility of IL-based physical CO_2_ absorption processes, enabling the comparison of the energy consumptions and process costs to those of the available industrial technologies. Xie et al.^198^ modeled the completely closed postcombustion carbon-capture process by physical absorption with different imidazolium-based ILs by employing an experimentally validated NRTL-RK thermodynamic model and an equilibrium-based absorber design model. The solvent and energy consumptions were analyzed using a pressure- and/or temperature-swing flash unit for the IL regeneration stage. The process simulations showed that solvent and energy requirements were determined by both the IL structure and flash operation conditions. Thus, by feeding the exhausted IL at 25 °C and 10 bar to the flash separator, when the solvent was regenerated by decreasing the pressure to 1 bar at the same temperature, the [emim][EtSO_4_] IL consumed the least energy (0.2 GJ/t_CO2_) owing to its low CO_2_ desorption enthalpy. In contrast, for the temperature-swing process (50 °C and 10 bar at the flash regenerator), the energy demand remarkably increased to 20.2 GJ/t_CO2_ for the optimized ([emim][PF_6_]) IL because the IL heat capacity played a more key role. Finally, the authors proposed the combination of [bmim][NTf_2_] with solvent regeneration by both releasing the pressure and increasing the temperature to achieve a promising compromise between a relatively low energy demand (1.3 GJ/t_CO2_) and the lowest solvent consumption (3 mol IL per mol of absorbed CO_2_) compared to only the pressure or temperature swing (24 and 125 mol IL per mol of absorbed CO_2_, respectively). Xu et al.^197^ simulated equilibrium-based processes to compare the performances of IL-based physical absorption, pressured-water scrubbing, and MEA-based scrubbing biogas upgrading processes. The sensitivities were analyzed to optimize the key design parameters (absorber pressure and number of stages and flash pressure), and 50% less energy was consumed for IL-based physical absorption and pressured-water scrubbing than for MEA-based scrubbing. Although more solvent circulated in the IL-based process, the ILs comprised a negligible amount of solvent. High-purity CO_2_ was produced using the IL method, whereas no pure CO_2_ was recovered with the water solvent. Remarkably, the green degree (GD) method proposed by these authors was used to assess the environmental impacts, and the IL-based biogas upgrading process was the most environmentally benign. The equilibrium-based process simulation analyzed by Liu et al.^189^ for capturing CO_2_ from shale gas using [bmim][NTf_2_] indicated that the IL-based CO_2_ capture process reduced the required total energy (including thermal and electric) by 40–66% (depending on the regeneration section configuration) compared with that of the methyldiethanolamine (MDEA)-process operating at the same high absorber pressure (60 bar). The advantage of the [bmim][NTf_2_]-based CO_2_ capture process was the lower energy duty in the IL-regeneration system, which used two flash tanks that required low amounts of heat. In the MDEA-based process, higher thermal energy was required for the stripper, and more energy was required to break the chemical bonds between the CO_2_ and MDEA. A similar qualitative conclusion regarding the IL-based energy duty was obtained by Xie et al.^199^ in the steady-stage process analysis of biogas upgrading using [bmim][NTf_2_]. IL scrubbing showed 11% reductions in energy consumption compared to water scrubbing. Ma et al.^201^ conducted steady-stage simulations and concluded that the energy consumption of the [bmim][BF_4_]-based process was 27% lower than that of the monoethanolamine (MEA)-based process. In addition, Taheri et al.^89^ modeled CO_2_ capture and simulated steady-stage processes using the UNIFAC-Lei thermodynamic model in Aspen Plus to compare the process performances obtained using methanol and [AlmimAmim][Tf_2_N] IL physical absorbents at low operating absorption temperatures (from −30 to 15 °C) and a high pressure (50 bar) while fixing L/G between 3.5 and 7.5 (mass/mass). Compared with the methanol-based process, the IL-based processes required much less energy to capture 1 kmol of CO_2_ while producing nearly the same amount of fuel gases. Remarkably, in this study, the indirect CO_2_ emission processes resulting from the electricity supply, cooling, heating, and compression were calculated and substantially reduced with respect to those obtained using the methanol absorbent. Ma et al.^191^ used equilibrium-based COSMO-SAC/Aspen Plus simulations to evaluate IL-based CO_2_ capture processes in power plants for low carbon emissions. Again, using ILs instead of MEA-based absorbents meant a lower thermal energy duty but higher electricity consumption, which reduced the total energy consumption by 30%. The novelty of the study was that the steady-stage analysis estimated the process cost, revealing that the IL-based carbon-capture process enabled a 30% savings on the primary cost. Similar conclusions were drawn by Li et al.^194^ through an equilibrium-based Aspen simulation and the evaluation of [emim][NTf_2_]-based CO_2_ capture in flue gas generated from coal-fired power plants, which obtained an energy consumption of 2.2 GJ/t_CO2_ in the optimized IL-based carbon-capture process with respect to the benchmark (3.6 GJ/t_CO2_) for MEA-based technology. Wang et al.^127^ used the equilibrium-based COSMO/Aspen Plus methodology, a room-temperature process for removing sour gas from syngas using the physical [bmim][NTf_2_] IL absorbent for simultaneously removing H_2_S (95.3%) and CO_2_ (97.6%). The sensitivity analysis was carried out to study the influence of the operating conditions on the CO_2_ and H_2_S recovery and solvent consumption, and the following optimized values were proposed for enhancing the process performance: L/G ratio = 3.8 mol/mol; absorber at 12 bar and 25 °C; flash 1 at 3 bar and 30 °C; flash 2 at 0.05 bar and 60 °C. The main advantage of the IL-based process was that it could be operated at room temperature, which saved considerable energy compared to the energy required for refrigeration in the benchmark Rectisol process. Later, Wang et al.^193^ performed a multilevel COSMO-SAC/Aspen Plus screening of IL absorbents for simultaneously removing CO_2_ and H_2_S from natural gas by integrating molecular and process simulations for simultaneously selecting the solvent and designing the process to enhance the natural gas purification. A thermodynamic absorption–selectivity–desorption index was defined as main IL selection criterion for preliminarily screening 1643 cation–anion combinations; later, other relevant properties (melting point, viscosity, and thermal stability) were considered for the final IL selection. The sensitivity was systematically analyzed using rate-based process simulations in Aspen Plus for a wide range of operating variables for the selected [bmpyr][H_2_PO_4_] IL to design an optimized IL-capture process for simultaneously removing CO_2_ and H_2_S with a RADFRAC column in the Aspen Plus simulator. Thus, a 14 m high packed column operating at 6 bar and L/G = 23 (mass/mass) and two flash tanks operating at 1/0.3 bar and 80/100 °C, respectively, were proposed for recovering high-purity CH_4_ and efficiently removing CO_2_ and H_2_S. The selected ILs ([bmpyr][H_2_PO_4_], [pmmim][H_2_PO_4_], [eepyr][H_2_PO_4_], and [emim][H_2_PO_4_]) all had notably higher process performances (lower column height and solvent and energy consumptions) than the benchmark ILs ([bmim][MeSO_4_], [bmim][PF_6_], and [bmim][TCM]) previously proposed in the literature. Wang et al.^103^ rationally designed an IL for simultaneously capturing CO_2_ and SO_2_ from flue gas by integrating UNIFAC predictions for the vapor–liquid equilibrium-based absorption–selectivity–desorption index, physical property (melting point and viscosity) constraints, and steady-stage process simulations of simultaneous and stepwise separations and then analyzed the sensitivity to fix the main operating variables of the absorber and regeneration flash units. The [EtOHmim][NTf_2_] IL was selected as the optimal physical absorbent for removing CO_2_ and SO_2_ from flue gas using the simultaneous separation flowsheet, for which the processes saved 56 and 71% of the solvent and energy compared to the amounts consumed by the benchmark [emim][TCB] IL. For stepwise separation, [epy][DCA] had the lowest solvent requirement and energy consumption, with savings of 31 and 24%, respectively, compared to the requirements of the previously selected [emim][BF_4_]. Furthermore, stepwise separation saved at least 50% more energy compared to the simultaneous separation of CO_2_ and SO_2_ in the same absorber, indicating the relevance of adequate process designs for separating multicomponent gas mixtures.

In the previously cited equilibrium-based process-simulation study by Haider et al.,^200^ a process optimization was developed using a built-in tool in the Aspen software based on a sequential quadratic programming algorithm and using the TAC objective function and operating variables for the upgrading (flash separators, temperature, and pressure) and liquefaction processes. The authors obtained a specific TAC of 2.6 M$/t_CO2_ for upgrading biogas based on biomethane liquefaction using [bmim][PF_6_]. Using steady-stage COSMO-SAC/Aspen Plus process simulations, Kazmi et al.^203^ evaluated the energy, exergy, and economic feasibility of CO_2_ capture from natural gas using pyridinium-functionalized ILs. By fixing the CH_4_ recovery and CH_4_ purity at 99%, the sensitivity analyses were analyzed to optimize the operating conditions. The IL-based carbon capture designed using the [3mpy][NTF_2_] solvent provided 90.1 and 80.3% overall energy savings relative to the energy consumed using the MEA chemical absorbent and 1,2-dimethoxyethane (DME) physical absorbent, respectively. Competitive results were also obtained for the process exergy ([3mpy][NTF_2_]: 13.3 MW; MEA: 57.4 MW; DME: 31.0 MW) and process cost savings (capital cost: 66%; operating costs: 81%; TACs: 78%) compared to MEA. Amiri et al.^204^ simulated the physical absorption of CO_2_ from a feed gas containing methane and CO_2_ using the [hmim][TCB] IL and an equilibrium-based model based on the NRTL-RK thermodynamic model for treating an inlet gas feed containing CO_2_ concentrations between 5 and 30 mol %. The operating conditions of the absorption column and regeneration system were optimized by analyzing the sensitivity for the benchmark solvent, dimethyl ether of polyethylene glycol (DEPG). The use of ILs demonstrated important advantages, including reductions of more than 27 and 37% in solvent and energy consumptions, compared to the DEPG solvent. In summary, all these technoeconomic analyses based on equilibrium-based process models revealed that IL-based carbon capture by physical CO_2_ absorption is a promising technology that could replace existing industrial processes, such as those that use physical absorbents (Rectisol and Selexol) or amine-based chemical absorbents. The IL-based approach yielded lower chemical and energy consumptions and reduced process costs for a wide range of CO_2_ partial pressures at the inlet (postcombustion, biogas, precombustion, etc.).

**Figure 13:**
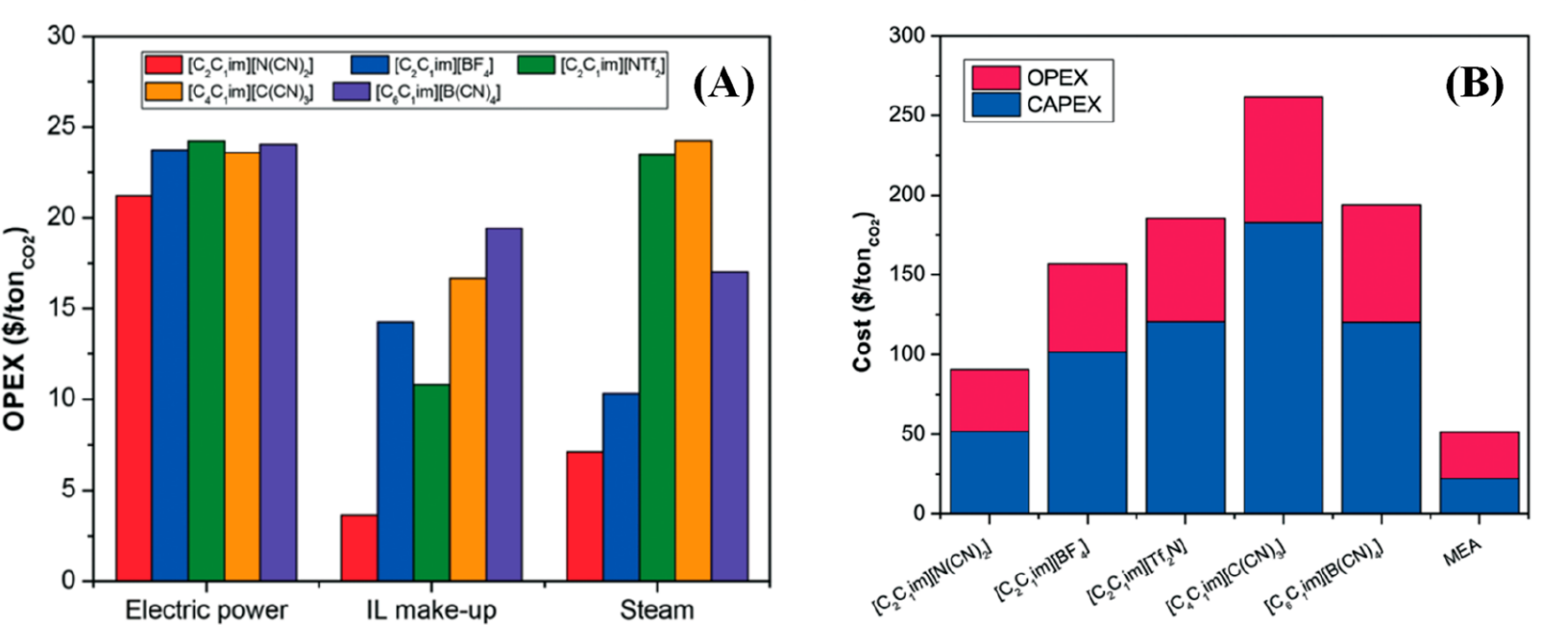
(A) Operating costs and (B) cost per tonne of captured CO_2_ using five ILs solvents for treating 3,240 t/h of flue gas containing 12% (v/v) of CO_2_ at 20 bar and 30 °C. Reproduced from ref (8). Copyright 2018 RSC.

Different conclusions were drawn when rigorous rate-based simulations were used in technoeconomic analyses. Garcia-Gutierrez et al.^167^ used the COSMO-SAC/Aspen methodology to compare the economic performances of three [NTf_2_]-based ILs to that of an MEA-based CO_2_ capture process for treating a biogas stream (35% CO_2_; 15 °C; 30 bar) to upgrade biomethane (95 vol %). The highest plant efficiency and lowest production costs were obtained using [emim][NTf_2_], even though it had the lowest CO_2_ absorption capacity of the three evaluated ILs. This was because [emim][NTf_2_] had the highest CO_2_/CH_4_ selectivity, which implied the highest biomethane production rate and, thus, decreased capital and operating costs. These results demonstrated the requirement for holistically evaluating ILs for capturing CO_2_ and clearly illustrated how process-simulation studies could contribute to the assessment of the IL absorbent performance for specific industrial CO_2_-capture applications. The sensitivity analysis conducted in this study^167^ determined that the optimal operating condition for the absorber was 20 bar, which provided the most balanced performance. Increasing the pressure to 30 bar increased the capital investment and electricity consumption, while decreasing the absorption pressure to 10 bar substantially reduced the IL absorption capacity. Garcia-Gutierrez et al.^167^ also performed a detailed technoeconomic assessment of the three biogas-upgrading plants designed using IL-based physical CO_2_ absorbents. APEA was used to estimate the operating and capital costs based on a percentage of the delivered-equipment cost and by considering a selling price of 34 $/kg and no IL losses (through evaporation or degradation) during the life of the plant for estimating the IL cost. Notably, even when IL-based carbon-capture processes required lower total energies, rate-based designs using IL-based physical absorbents provided higher process costs than the MEA-based process (MEA costs: CAPEX = 421 $/t_CO2_ and OPEX = 245 $/t_CO2_; [Emim][NTf_2_] costs: CAPEX = 1436 $/t_CO2_ and OPEX = 293 $/t_CO2_), which was explained by the additional biogas-compression-related equipment and energy costs in the IL-based processes. Again, using rate-based simulations, de Riva et al.^35^ extended the economic analysis for a postcombustion system to a wide range of operating conditions in the absorber and regeneration stages to evaluate the electrical, refrigeration, and steam requirements and costs of the global CO_2_ capture process using the [emim][NTf_2_] IL. A completely closed process for capturing carbon from flue gas was simulated using a packed absorber column at 20–50 bar, to recover 90% CO_2_, and a regeneration flash unit decompressing between 1 and 10 bar, to regenerate high-purity (>98 mol %) IL, which was recirculated—after conditioning—to the absorption tower. OPEX was calculated as the sum of the costs of the electricity used by the pumps and compressors in the process, the refrigeration water used as a coolant, and the high-pressure steam used prior to the temperature-swing unit to heat the IL + CO_2_ mixture to the regeneration temperature; whereas CAPEX was estimated by considering only the absorption column, compressors and spent IL (estimated based on the absorbent hold up). Increasing the absorber pressure to 20 bar implied higher pressurization costs that directly translated into higher overall OPEX. Higher regeneration pressures, on the other hand, always required higher regeneration temperatures, which increased OPEX. The proposed optimized conditions corresponded to those of an absorption column operating at 20 bar and 33.3 °C and a regeneration flash operating at 1 bar and 168.4 °C for achieving an IL purity of 99.5 mol %. Under these operating conditions, the total energy invested in the process was 1.4 GJ/t_O2_, which agreed well with the results obtained in the pioneer study by Xie et al.^199^ (1.3 GJ/t_O2_) and were remarkably lower than those obtained for conventional absorbents in other processes reported in the literature, such as 4.2 GJ/t_CO2_ for an amine-based system and 4.07 GJ/t_CO2_ for the aqueous ammonia process, or for other ILs used for CO_2_ chemical absorption, such as 3.2 and 3.6 GJ/t_CO2_ for [bmim][MeCOO]^142^ and aprotic heterocyclic anion (AHA)-based^205^ ILs, respectively. However, the total calculated OPEX of the optimal scenario was 73.3 €/t_CO2_, which was higher than the estimations presented in literature for other chemical-absorption-based processes (62 $/t_CO2_ using AHA-ILs^205^ and 25 $/t_CO2_ using amine-based absorbents^142^), which operate at lower absorption pressures. The calculated CAPEX ascended to 6.82 M€, a reasonable value for the treated flue gas flow, determined based on the high compressor cost (3.60 M€) compared to that of the absorption column (1.59 M€) or solvent (1.63 M€, considering a favorable IL price of 20 €/kg).^35^ Mota et al.^8^ presented a detailed process economic assessment of IL-based physical CO_2_ physical capture from flue gas. The monetized KPIs, CAPEX, and OPEX, were systematically evaluated as a function of the thermodynamic and transport characteristics of the ILs. The rate-based processes demonstrated that although the IL viscosity and CO_2_ solubility in ILs decisively impacted the performance of the carbon-capture process, other absorbent properties, such as heat capacity, absorption enthalpy, density, and surface tension, must also be considered. These results revealed that multicriterion-analysis-based KPIs were required for designing ILs that have enhanced absorbent properties in the temperature and composition ranges of interest for each carbon-capture system. As in previous rate-based process simulation studies, Mota et al.^8^ found that the electric energy consumed in compression operations mainly contributed to OPEX for IL-based carbon-capture processes (Figure 13A), whereas the heat-exchange- and IL-related process costs composition could be substantially decreased by properly selecting the IL absorbent.

As the latest step in the economic analysis of IL-based physical CO_2_ absorption processes, the TAC was estimated based on rigorous rate-based process simulation results and globally monetized to assess the economic performance of the carbon-capture process. Thus, Mota-Martínez et al.^8^ used the selected ILs and CAPEX and OPEX contributions to analyze the TAC per tonne of CO_2_ captured from postcombustion flue gas and compared it to the process costs for using an aqueous MEA solution as the benchmark industrial absorbent (Figure 13B). Higher costs in the IL-based carbon-capture process (90 $/t_CO2_ for the optimized IL [emim][DCN]) were obtained compared to the MEA-based technology (30–50 $/t_CO2_).^188^ Therefore, the rigorous rate-based process simulations by de Riva et al.^35^ and Mota et al.^8^ revealed that the two main drawbacks of the postcombustion carbon-capture process using IL-based physical CO_2_ absorption were the mass-transfer limitations, related to the high IL viscosity, and the energy penalty, due to the required high operating pressure. Similar conclusions regarding the economic feasibility of IL-based physical CO_2_ absorption processes were reported for a biogas upgrading application based on rate-based process designs.^167^ Thus, the total biomethane production costs were substantially higher for the studied IL absorbents (9.2–11.3 $/GJ) than for the current industrial MEA-based process (5.4 $/GJ),^8^ mainly owing to the additional capital and costs related to the compression units in IL-based processes.

#### 4.1.3 Process Optimization in IL-Based Carbon Capture by CO_2_ Physical Absorption

Processes have also been optimized to advance the development of IL-based physical CO_2_ absorption technology. Valencia-Marquez et al.^185^ simultaneously optimized the product and process design for CO_2_ postcombustion capture by employing ILs as physical absorbents and using a mixed-integer nonlinear programming method. The IL properties were estimated based on GC methods combined with equilibrium empirical correlations, whereas the steady-stage process simulations involved an absorber and flash regeneration stage, as described in Scheme 3, Owing to the complexity of the underlying MINLP problem, only local optimal solutions were sought. A multiobjective optimization framework enabled the consideration of conflicting energy-consumption- and CO_2_-recovery-related design objectives, providing a Pareto curve for different technological solutions, where increasing the CO_2_ recovery implied higher IL quantities. Interestingly, the optimized IL chemical structure was different for each point on the Pareto curve, demonstrating that the IL and process design were strongly related. Leonzio et al.^202^ used the ([hmim][NTf_2_]) IL to optimize the carbon-capture process from flue gas, Aspen Plus software for rate-based process simulations with the APEA PR thermodynamic model for estimating process costs, and Minitab for the response-surface methodology to minimize the costs and maximize the amount of CO_2_ captured through the response-surface methodology.^192^ The inlet temperature of the flue gas, absorption column pressure, CO_2_ composition of the flue gas, and height of the absorption column were the considered factors, while the CO_2_ recovery percentage, operating costs, and capital costs were the analyzed responses. The response-surface methodology was applied to identify important factors and understand their relationship with the performance criteria. The study results suggested the suitability for employing a face-centered central composite design. Under the optimal conditions, the flue gas inlet temperature was 227 °C, the column pressure was 30 bar, the CO_2_ concentration in the feed gas was 24%, and the height of the absorber was 1.36 m. These conditions ensured 93.7% CO_2_ recovery, the specific operating costs were 0.66 million €/t_CO2_, and the capital costs were 52.2 €/t_CO2._ Similarly, the costs of the IL-based carbon-capture process were clearly higher than those reported for the benchmark MEA-based technology. Zhang et al.^119^ proposed a computational approach for simultaneously optimizing the IL and process design for enhancing the efficiency of the overall physical CO_2_ absorption process by integrating rigorous rate-based process simulations and hybrid models (UNIFAC-PR and ANN-based GG) to predict the physical, kinetic, and thermodynamic properties and the MINLP optimization method. The flowsheet of the modeled process matched that depicted in Scheme 3, mainly including an absorption packing column and flash regeneration stage, for treating a precombustion flue gas flowing at 10 kmol/s and containing 40 mol % CO_2_ at 20 bar and 40 °C. As a result of the comprehensive computer-aided IL and process design (The optimization problem involved 52 discrete variables, 3086 single variables, 3121 equations, and 58,032 nonlinear matrix entries.), an [EEOMA][BETA] IL that had enhanced absorbent properties was designed, which enabled a CO_2_ absorption process (based on the flowsheet depicted in Scheme 3) to be obtained, saving 14.8% of the total cost compared with the that of the Selexol process. Further experimental studies must be conducted to determine the feasibility of the synthesis and expected CO_2_ absorbent properties of the designed IL.

In this respect, Wang et al.^195^ used a support vector machine model, which is a machine-learning approach, and COSMO-RS to select, from among 29 cations and anions, suitable ILs that had favorable absorption, selectivity, and desorption properties and constraints for IL viscosity and melting points. Finally, through rate-based process simulations using Aspen Plus, [emim][TCM] was selected as optimal IL, enabling a 12.9% savings for the TAC in postcombustion carbon-capture processes, compared to the TAC for the [emim][NTf_2_] IL, which was previously selected by de Riva et al. The reduced TAC was ascribed to the smaller MW and viscosity and higher CO_2_ solubility of cyano-based ILs.^35^

#### 4.1.4 Environmental Impact Analysis of IL-Based CO_2_ Physical Absorption Process

**Figure 14:**
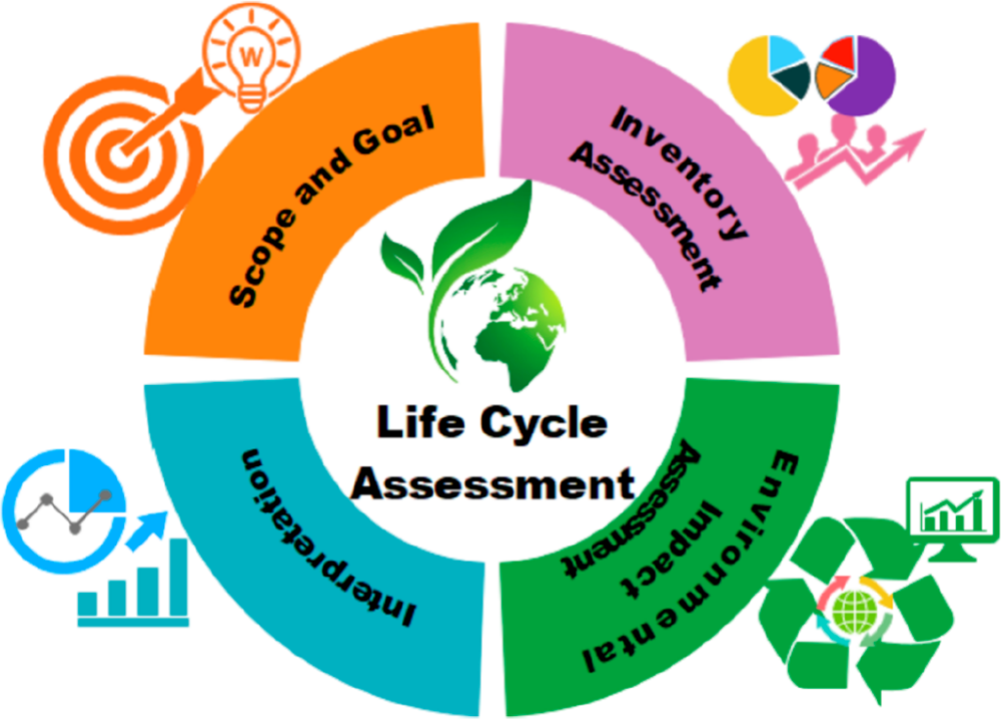
Approach for life-cycle-analysis (LCA) methodology. Reproduced from ref (14). Copyright 2022 Elsevier.

In the final step, process simulation studies have been applied to evaluate the environmental impacts of IL-based practical industrial applications. In this regard, LCA has been proposed as a strategic tool that can quantitatively evaluate the environmental impact risk, ecological performance, and environmental consequences and provide a basis for developing an alternative method for improving IL absorbents for effectively capturing CO_2_ (Figure 14); however, to the best of our knowledge, available studies are scarce in the literature.^120,177,206^ Cuellar-Franca et al.^206^ presented a methodology for estimating the LCA impacts of ILs based on the synthesis route, cycle tree, and life-cycle assessment steps for practical application to IL-based CO_2_ capture technology and found that the cradle-to-gate production of the CO_2_-absorbent [P_66614_][124Triz] IL presented much higher impacts compared to that of the benchmark MEA absorbent, including the global warming potential, mainly owing to the many precursors and considerable energy used for synthesizing ILs. In this regard, Zhang et al.^120^ analyzed cradle-to-grave LCA for the CCS process previously developed using process simulations with a [bmim][NTf_2_]-based physical CO_2_ absorbent and steady-stage modeling with COSMO-SAC/Aspen Plus methodology.^127,193^ The IL-based CCS process included the following four stages: dehydration, syngas purification, solvent regeneration, and gas separation and storage units. Three configuration processes, requiring different solvent and energy consumptions, were evaluated.^127,193^ The system boundary included IL solvent production and absorbent use in CO_2_ capture and storage. According to the estimated global warming potential (GWP) the LCA results showed that the studied IL-based carbon capture could not reduce carbon emissions owing to the main environmental impacts of [bmim][NTf_2_] production in the life cycle of the IL-based carbon-capture process, which accounted for approximately 90% of the entire life cycle owing to the considerable raw material inputs, complex synthesis steps, and strict synthesis requirements. Therefore, the LCA results indicated that future research should focus on designing green IL compounds through sustainable synthesis schemes to accelerate the industrialization of IL-based carbon-capture processes. As an additional contribution, the LCA results revealed that the optimization of the CO_2_-capture process, focusing on solvent and energy savings, could reduce the environmental burden of the entire process and that the specified CO_2_ recovery was a key aspect to consider when designing IL-based carbon-capture processes because although it reduced the overall environmental impacts, it directly emitted more greenhouse gases. This pioneer process simulation-based study by Zhang et al.^120^ demonstrated that by helping to develop designs to improve its environmental performance, LCA is a useful tool for analyzing the environmental impacts of each stage of the IL-based carbon-capture process.

In summary, process simulation analyses have remarkably contributed to the knowledge and development of carbon capture through IL-based physical CO_2_ absorption. Equilibrium-based process modeling has revealed the technical suitability for using ILs to efficiently recover CO_2_ from postcombustion, biogas, precombustion, and capture systems, which have enabled the improvement of the key process performance values (energy and solvent consumption, costs, and environmental impacts) by properly designing the absorption and IL-regeneration stages through using sensitivity analysis or process optimization. Remarkably, however, rigorous rate-based process simulations have exhibited severe kinetic control in absorption columns using commercial packings, which has implied much higher solvent requirements, energy duties, equipment sizes, and, consequently, operating and capital costs. In fact, most rate-based studies have concluded that carbon-capture technology using IL-based physical CO_2_ absorption is not competitive compared to current industrial physical absorbents (i.e., the Selexol process) or amine-based chemical absorbents, at least for relatively low-CO_2_-partial-pressure carbon-capture systems, such as postcombustion and biogas. Moreover, LCA analysis based on rigorous rate-based process simulations revealed higher environmental impacts for IL-based carbon-capture processes than for conventional industrial technologies, which is mainly related to the low degree of sustainable IL synthesis. Recent integrated IL and process design optimizations, however, have provided opportunities to find improved technical solutions, which must be experimentally validated. Some other main contributions of these process-simulation analyses are as follows: (i) To obtain ILs that have favorable absorption kinetics and thermodynamics, viscosity is a key property to consider for IL selection in combination with CO_2_ gas solubility. (ii) The use of solvent mass units is convenient for avoiding misleading molar-weight-related effects on IL selection. (iii) The vacuum requirement is the main contributor to the process costs, which emphasizes the importance for designing high-thermal-stability ILs to enable the use of more favorable temperature–pressure swing regeneration.

### 4.2 Carbon Capture by Chemical Absorption

**Scheme 4:**
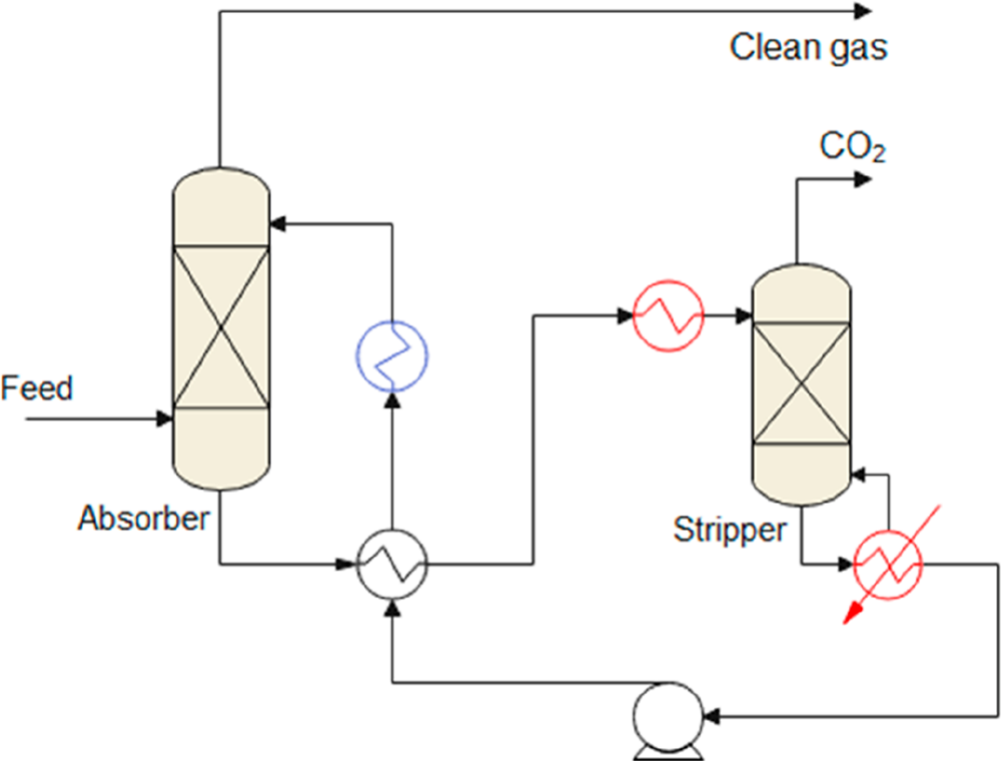
Simplified Process Diagram for CO_2_ Chemical Capture Using ILs

This section focuses on the main contributions of process simulations over the past few years for designing chemical-absorption-based CO_2_-capture processes using ILs, which can chemically react with CO_2_ as an alternative to physical absorption. Chemisorptive ILs have the advantage of higher CO_2_ solubilities than physical absorbents, especially at low CO_2_ partial pressures.^207^ For this process, the usual configuration is the same as that for physical CO_2_ capture, which is performed in an absorption column and then the saturated IL is regenerated and recirculated to the absorption column (Scheme 4). For the regeneration step, the most common alternatives are the use of flash separation by temperature^208^ or/and vacuum^36^ or the use of a stripper column,^169^ where the IL is introduced at the top stage and heated in a reboiler. To reduce the CO_2_ partial pressure further and facilitate the IL regeneration, stripping agents, such as air,^134^ have been used in carbon-capture applications, such as biogas purification, where the main objective is not the CO_2_-rich stream, but the efficient carbon capture in the absorber.

**Figure 15:**
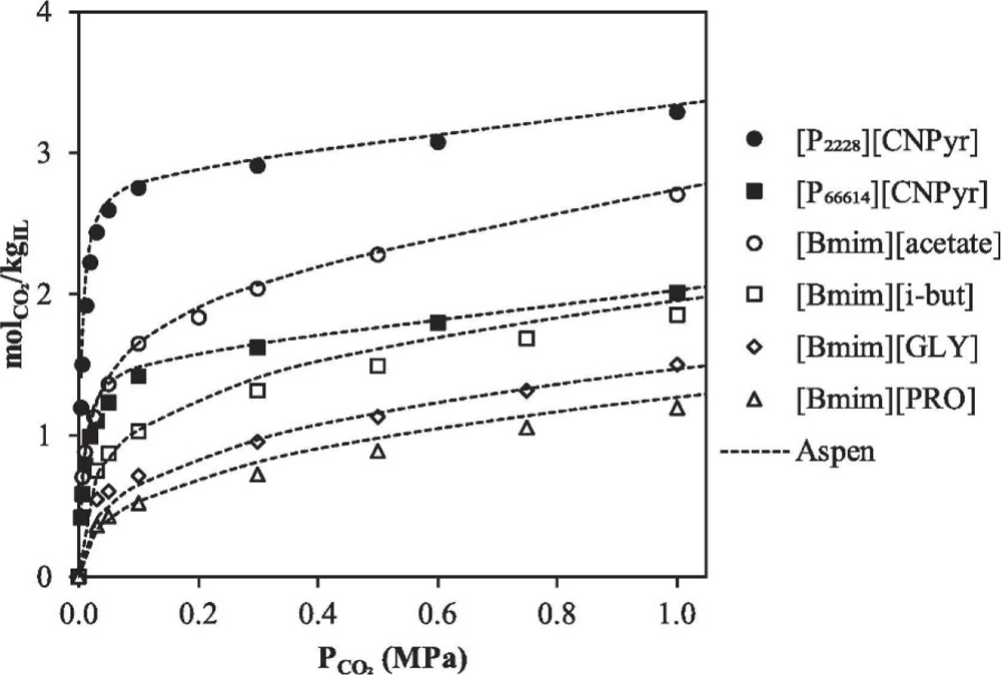
Experimentally measured CO_2_ absorption isotherms for several ILs at 40 °C. Reproduced from ref (36). Copyright 2020 Elsevier.

The gas feed that is used depends on the studied system, most of which are in one of the following scenarios: postcombustion, (N_2_/CO_2_ mixture containing 10–15% CO_2_ at atmospheric pressure), precombustion (H_2_/CO_2_ mixture containing 40–55% CO_2_ at 30–40 bar), or biogas (CH_4_/CO_2_ mixture containing 35–45% CO_2_ at 4–8 bar). Several ILs presenting CO_2_ chemical absorption, including carboxylate-based,^209^ aprotic heterocyclic anion (AHA)-based,^210^ amino acid-based,^211^ amine-functionalized,^212^ and DBU-based ILs,^213^ have been reported. However, as the data listed in Table 2 show, most process simulation studies on CO_2_ chemical absorption have been conducted based on acetate anion ([MeCOO])- and AHA-based ILs, which present the most promising properties for industrial-scale applications (Table 3). In acetate ILs, carbon dioxide usually reacts with the imidazolium cation in a 2:1 (IL:CO_2_) stoichiometry.^214^ At lower CO_2_ partial pressures, this reaction is displaced toward the product side. However, the chemical reaction further increases the already initially high viscosity of this family of ILs. AHA ILs are usually paired with tetraalkylphosphonium cations, where the heterocyclic nitrogen atom of the anion reacts with CO_2_ in a more favorable 1:1 stoichiometry without further increasing the viscosity of the IL.^210^ The CO_2_ absorption isotherms of representative IL chemical absorbents are compared in Figure 15. Clearly, [P_2228_][2CNPyr] presents the highest solubility, owing to its favorable 1:1 stoichiometry, followed by [bmim][MeCOO]. Despite having almost the same reaction capacity (mol/mol) as [P_2228_] [2CNPyr], [P_66614_][2CNpyr] is the least soluble owing to its high MW (Table 3).

**Table 3:** Physical Properties and Physical and Chemical CO_2_ Solubilities of Most Relevant ILs Evaluated in Chemical CO_2_ Absorption

ILs	MW (g/mol)	ρ (kg/m^3^)	μ (mPa·s)	*K*_H_ (bar)	*K*_eq_	ΔH_R_ (kJ/mol)	mol_CO2_/kg_IL_ (1 bar, 40 °C)
[P_2228_][2CNPyr]	322.47	940	163.45	6.7	1,389.84	–47.72	–47.72
[P_66614_][2CNPyr]	574.95	900	166.42	3.93	421.61	–39.77	–39.77
[bmim][MeCOO]	198.26	1,030	179.79	9.31	65.56	–35.12	–35.12
[bmim][*i*-but]	226.32	1,010	198.02	13.57	25.4	–19.38	–19.38
[bmim][GLY]	213.28	1,030	423.87	18.06	6.78	–22.92	–22.92
[bmim][PRO]	253.34	1,060	891.32	17.15	5.6	–14.03	–14.03

#### 4.2.1 Ionic Liquid Performance in CO_2_ Chemical Absorption Unit

The inclusion of the chemical absorption reaction in the simulation environment remains a challenge that must be overcome. Usually, descriptions of both the chemical and physical mechanisms are required. The first step involves the attempt to reproduce the CO_2_ absorption isotherms, which requires the inclusion of the reaction products and their corresponding equilibrium reactions. For this purpose, most available process simulation studies have fitted reliable experimental absorption data to thermodynamic models. For the reaction kinetics, experimental data are almost nonexistent. Because the requirement for experimentally measured equilibrium data to define ILs has limited the use of process simulations for screening , process simulations are usually used as a benchmark tool for performing technoeconomic studies and comparing the performances of experimentally evaluated ILs with those of conventional CO_2_-capture technologies (such as absorption with aqueous amine solutions).

**Figure 16:**
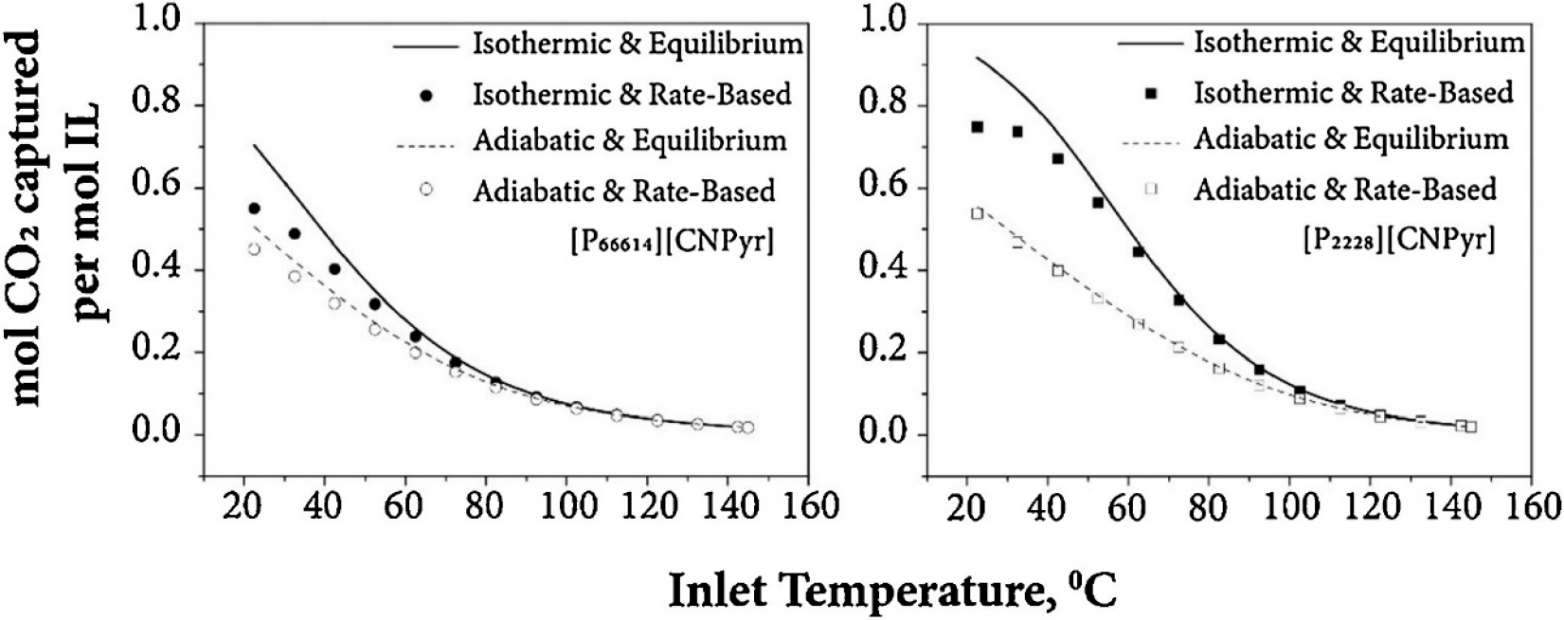
CO_2_ load per mol of IL under adiabatic and isothermal conditions vs. inlet absorber temperature at constant inlet CO_2_ partial pressure of 0.26 bar. Reproduced from ref (43). Copyright 2018 Elsevier.

Several studies have revealed that the IL selection substantially affects CO_2_ chemical absorption. A pioneer study, including a process simulation, was reported by Shiflett et al.^142^ In that study, [bmim][MeCOO] was used as the chemical absorbent. The equilibrium isotherms were fitted to a modified RK model and introduced to the Aspen Plus process simulator. The study objective was to compare the IL-based process with commercial MEA technology under postcombustion conditions. A complete technoeconomic analysis using RADFRAC equilibrium columns found that the IL could reach high CO_2_ removal rates (>90%) while reducing the energy duty and economic investment. However, because the physical absorption did not include the mass transfer when highly viscous media, such ILs, were used, the results may have been inaccurate. In fact, Krupiczka et al.^216^ modeled a packed bed column, compared [bmim][MeCOO], [emim][MeCOO], and MEA solutions under the same conditions, and concluded that although the absorption capacities were very similar, the contact times required for the ILs were much higher than that for the MEA, which suggested that the inclusion or exclusion of the mass transfer was a critical decision that may substantially impact the results of process simulations. de Riva et al.^43^ analyzed CO_2_ capture in [P_2228_][2CNPyr] and [P_66614_][2CNPyr] under postcombustion conditions by applying a rigorous rate-based model that included potential mass-transfer limitations. Figure 16 shows that operation under rate-based isothermal conditions reduced the CO_2_ loading in the IL, especially at lower operating temperatures, which is the same effect as that previously described in the physical absorption section (Section 4.1). However, the study also considered the use of adiabatic packing columns. Under adiabatic conditions, the reaction enthalpy increased the solvent temperature in 20 °C in certain cases, resulting in reduced viscosity and eliminating the mass-transfer limitations (Figure 16).

#### 4.2.2 Complete Carbon Capture Process Modeling for Technoeconomical Analysis

Some of the advantages of process simulations are that the solvent performance can be evaluated under more realistic conditions and that the effects of different operating conditions on the technical and economic feasibilities of the carbon-capture process can be determined. The input CO_2_ partial pressure is one of the parameters that substantially impacts the performance of the carbon-capture process. As listed in Table 2, most simulation studies have been conducted based on postcombustion streams (10–15% CO_2_ at atmospheric pressure), followed by biogas streams (35–45% CO_2_ at 4–8 bar). Hospital-Benito et al.^36^ analyzed the complete CO_2_-capture process using six ILs (two AHA ILs, [P_2228_][2CNPyr] and [P_66614_][2CNPyr]; two acetate ILs, [bmim][MeCOO] and [bmim][*i*-but]; and two amino acid-based ILs, [bmim][GLY] and [bmim][PRO]) in three CO_2_-capture scenarios, including postcombustion (at 1 bar and 13% CO_2_), biogas (at 3.2 bar and 38% CO_2_), and under precombustion (at 32.7 bar and 40% CO_2_) operating conditions. The process was evaluated using adiabatic columns modeled as RADFRAC packed columns under rate-based conditions.

**Figure 17:**
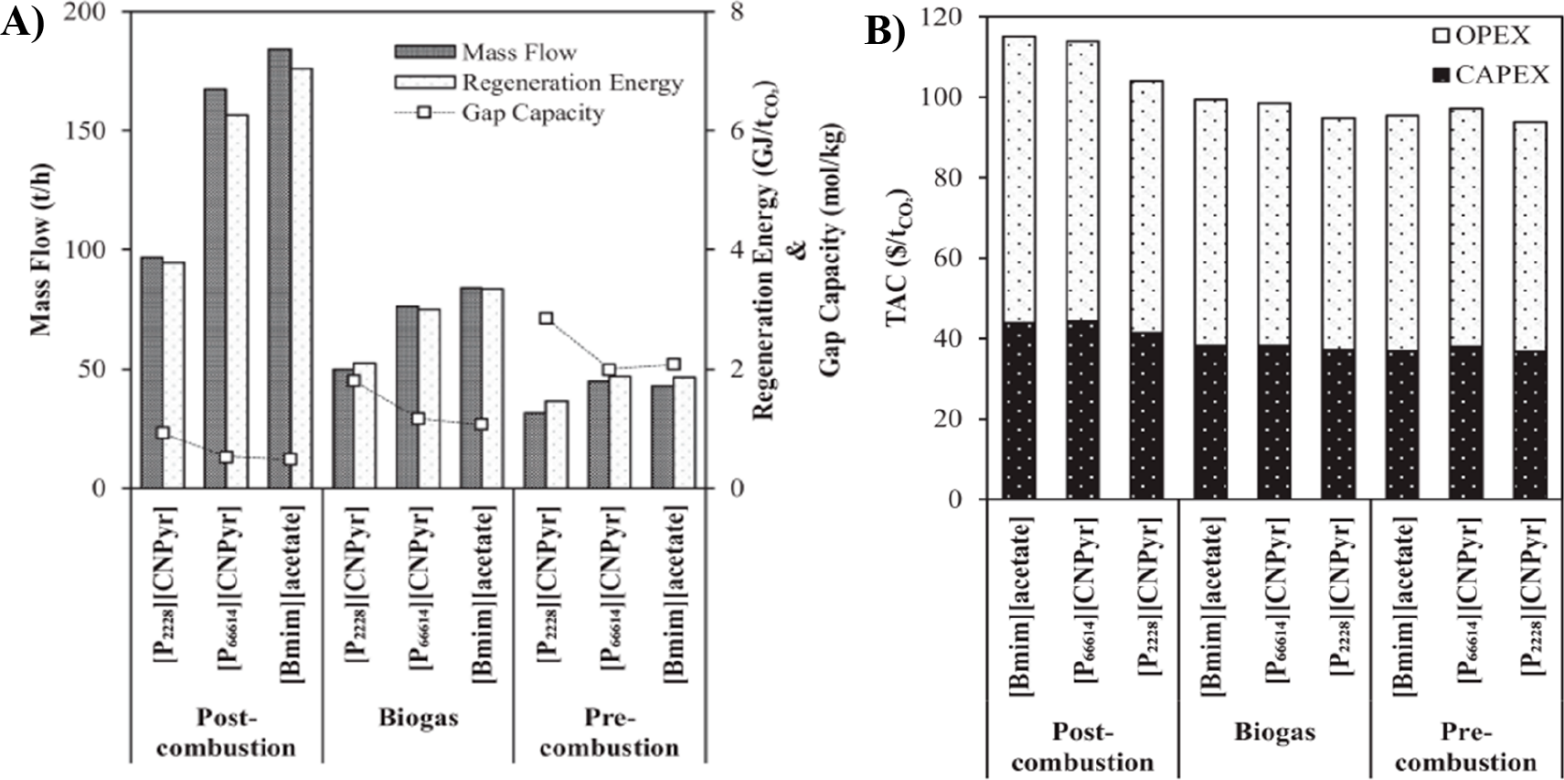
Correlations between IL mass flow and (A) energy requirements and (B) total annualized costs of ILs in postcombustion (at 13% CO_2_ and 1 bar), biogas (at 38% CO_2_ and 3.2 bar), and precombustion (at 40% CO_2_ and 35.7 bar) CO_2_-capture processes. Reproduced from refs (36, 37). Copyright 2020, 2021 Elsevier.

Figure 17 shows the results for the three optimal ILs. The results showed that high viscosity (400–900 mPa·s at 40 °C) highly impeded amino acid-based ILs processes, far more ILs were required to achieve the required recoveries, and AHA-based ILs were the optimal candidates (Figure 17A). In addition, operation at higher CO_2_ partial pressures improved the IL performance and substantially reduced the IL flow required to capture CO_2_, which consequently reduced both the energy consumption and equipment sizes. Under postcombustion conditions, [P_2228_][2CNPyr] was by far the optimal candidate, consuming almost half the solvent compared to that consumed by the other ILs. A technoeconomic analysis of these processes^37^ revealed a TAC in the range 90–110 $/t of captured CO_2_, indicating that precombustion was the most feasible scenario (Figure 17B). A detailed cost analysis revealed that the vacuum in the regeneration column was the factor that was the most responsible for the high costs. For [P_2228_][2CNPyr], the elimination of the vacuum requirement under postcombustion conditions reduced the TAC from 104 to 85 $/t, which was much closer to the TAC of the current amine technology (75 $/t) under similar conditions.^37^

**Figure 18:**
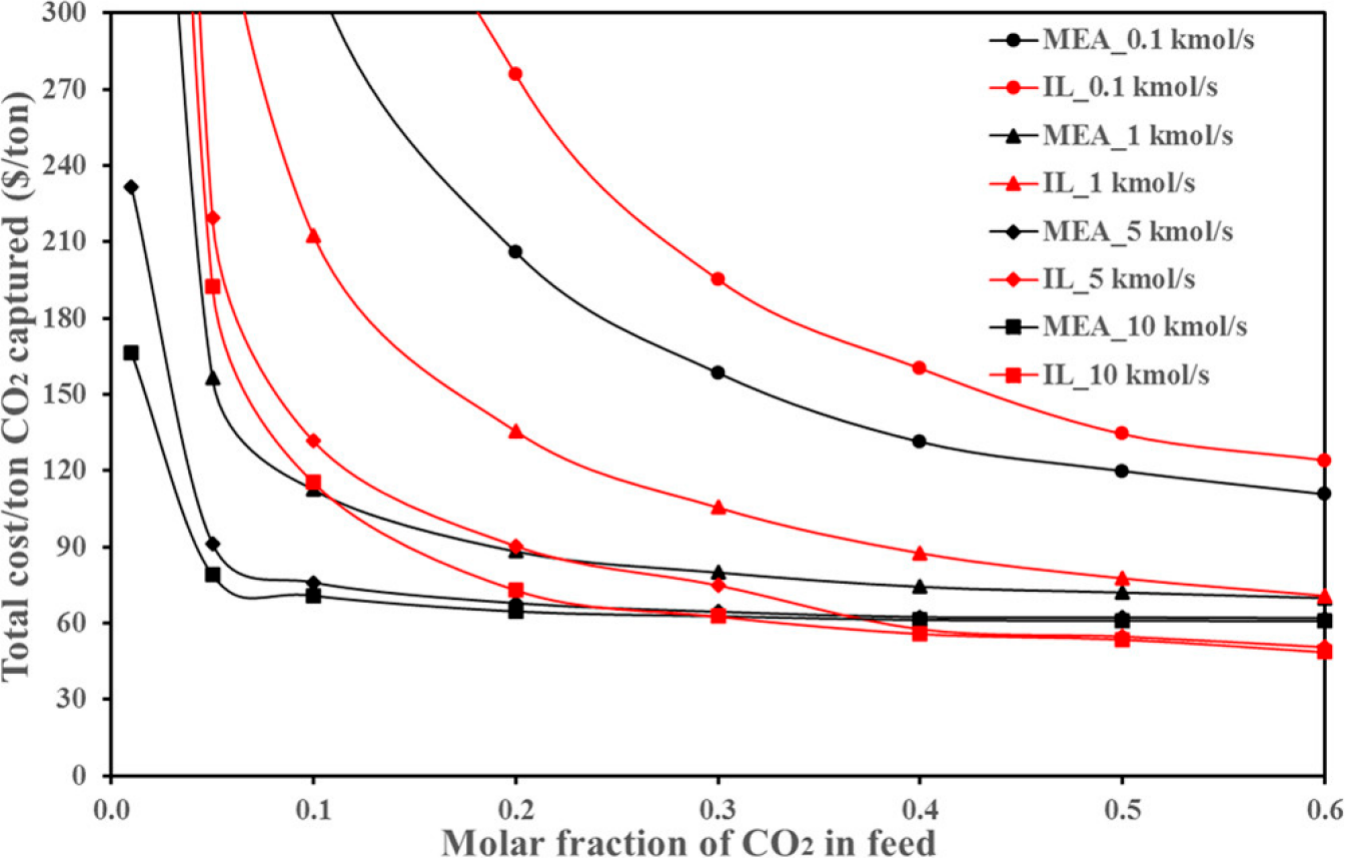
Comparison of total annualized costs for MEA and [bmim][MeCOO] over wide range of CO_2_ feed contents (from 1 to 60%) and flow rates (0.1, 1, 5, and 10 kmol/s) at 7.9 bar. Reproduced from ref (132). Copyright 2018 ACS.

Nguyen et al.^132^ simulated a complete carbon-capture process using [bmim][MeCOO], including the flue gas pretreatment and subsequent compression of the captured CO_2_. In addition, the authors performed a sensitivity analysis on the process scale and the inlet CO_2_ concentration by maintaining a constant feed at 7.9 bar. At each point, the TAC was minimized. The results in Figure 18 show that the cost increased when the inlet CO_2_ concentration was reduced, rendering MEA absorption as the most feasible technology until the inlet concentration exceeded 40%.

Figure 18 shows the economy of scale for the process; for example, at an inlet CO_2_ concentration of 0.2, raising the molar flow from 0.1 to 10 kmol/s reduced the specific cost from 280 to 85 $/t_CO2_. Similar findings were obtained by Hospital-Benito et al.,^37^ where an increase in the scale from 1 to 100 kmol/h reduced the specific cost from 5,800 to 93.9 $/t_CO2_ under postcombustion conditions.

**Figure 19:**
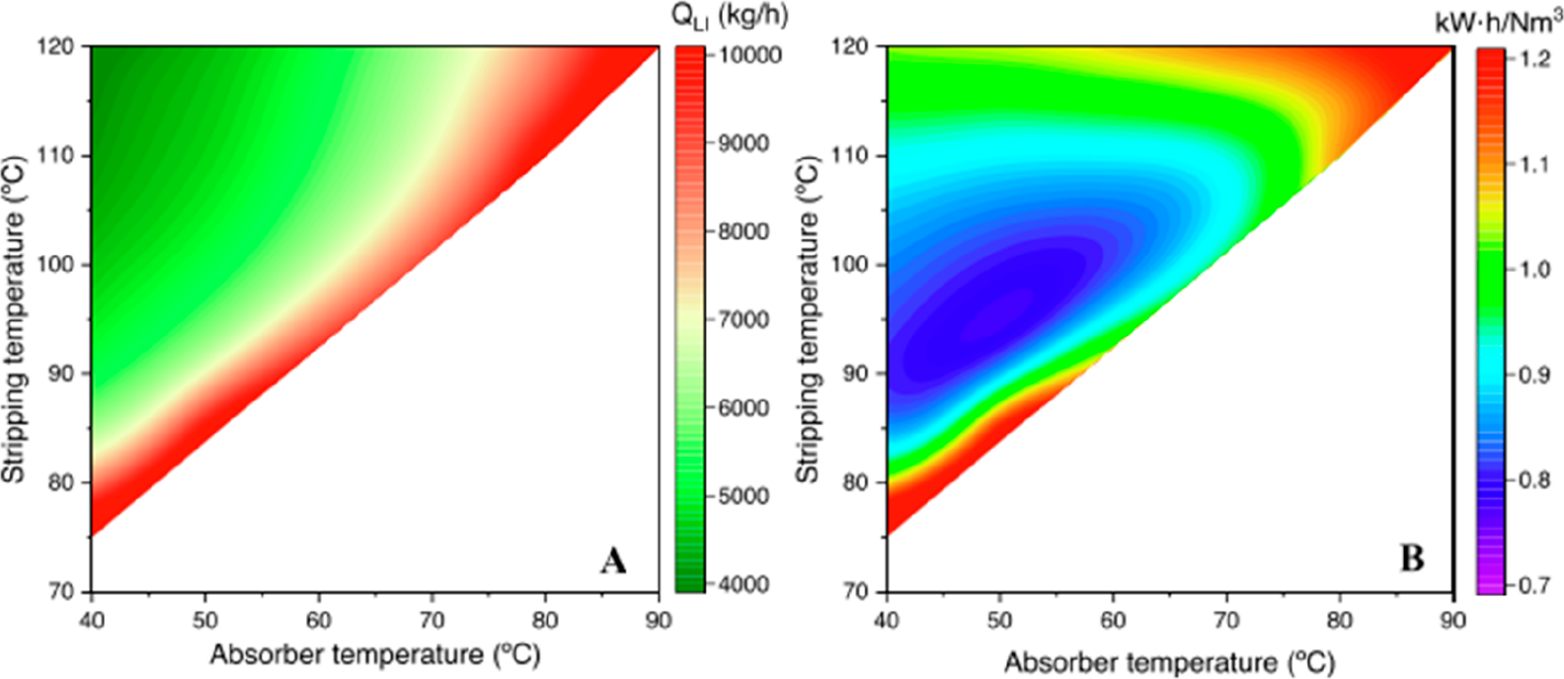
(A) Solvent requirements and (B) energy consumption plotted as functions of operating temperature in biogas purification (at 300 N m^3^/h, 40% CO_2_, and 1 bar) using [P_2228_][2CNPyr]. Reproduced from ref (58). Copyright 2022 Elsevier.

Process simulations have been performed to evaluate the role of the operating conditions in IL-based CO_2_ chemical capture. The operating temperatures in the absorption and regeneration columns were the parameters that were analyzed. Because of CO_2_ chemical absorption, temperatures higher than those for physical absorption are usually required to reverse the reaction. In addition, an upper limit of 150 °C is usually considered to avoid thermal degradation. In Figure 19, the effects of the operating absorber temperature on the solvent and energy consumption are clearly depicted for CO_2_ capture using [P_2228_][2CNPyr] for upgrading biogas.^58^

Clearly, as expected, a reduction in the absorption temperature reduced the solvent requirements and, consequently, the global energy consumption. Operation at higher temperatures improved the quality of the regenerated IL and reduced the solvent requirements. However, further temperature increases implied that more energy was consumed for optimizing the regeneration temperature. Additionally, operation under adiabatic conditions raised the IL temperature at the absorber outlet, which reduced the amount of energy required for regeneration, which was critical for reducing the thermal requirements of the CO_2_-capture process. Additionally, by operating at higher absorption pressures, the reaction enthalpy could heat the IL to the regeneration temperature, reaching a nearly autothermic process in which almost no thermal energy was consumed while operating at 69 °C at the absorber and 100 °C during the IL regeneration step.^58^

**Figure 20:**
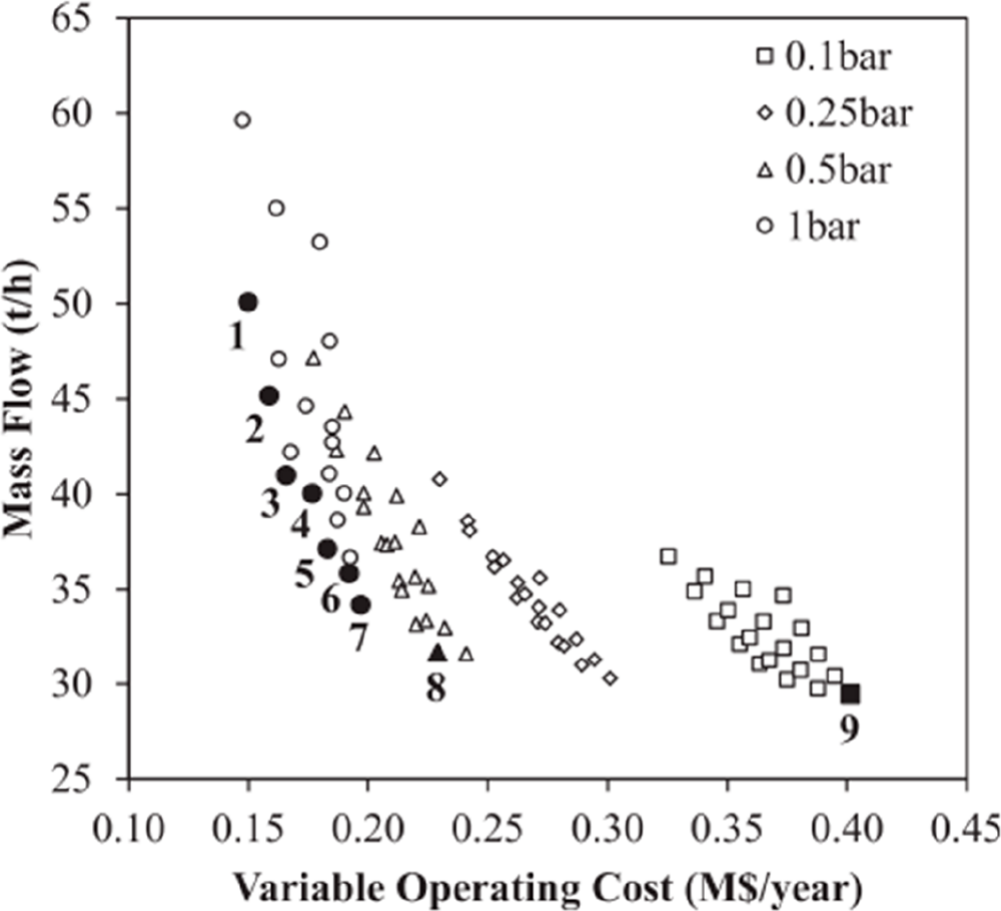
Required IL mass flows and variable operating costs at different vacuum regeneration levels in precombustion CO_2_ capture (at 250 kmol/h, 40% CO_2_, and 32.7 bar) using [P_2228_][2CNPyr]. Reproduced from ref (65). Copyright 2022 Elsevier.

To decrease the temperature required in the regeneration step, vacuum is commonly used to reduce the CO_2_ partial pressure. In the regenerator, operation at lower pressures (up to 0.1 bar) improved the regeneration grade of the IL, which reduced the solvent requirements. However, vacuum is an energetically demanding operation and can account for more than 50% of the total operating cost of the carbon-capture process.^37^ As shown in Figure 20, Hospital-Benito et al.^65^ studied the regeneration pressure for a precombustion CO_2_-capture process at different vacuum pressures using the [P_2228_][2CNPyr] IL. Clearly, although operation at higher vacuum pressures decreased the IL solvent requirements, it increased the operating cost of the CO_2_-capture process. Despite almost double the IL mass flow, a minimum operating cost of 64.1 $/t_CO2_ was achieved during operation at 1 bar.

#### 4.2.3 Process Optimization in IL-Based Carbon Capture by CO_2_ Chemical Absorption

Seo et al.^126^ obtained similar results where although a vacuum of 0.3 bar reduced the required temperature almost 40 degrees (from 140 to 95 °C) during regeneration, it increased the process TAC by 70%. For their process, the authors found that the optimal pressure was 0.77 bar.

According to these results, vacuum pressure is only desirable when other regeneration options are not available, such as when the IL has a low thermal stability or the absorption occurs at low CO_2_ partial pressures and is necessary to achieve a feasible gap capacity. Other regeneration alternatives have also been presented. For example, in biogas upgrading, air is usually used as a stripping agent to enable the CO_2_ partial pressure to be reduced and the solvent to be partially cooled, which improves the global economy of the carbon-capture process.^58^ Recently, for IL regeneration, the use of a hollow-fiber-membrane vacuum has been identified as a promising alternative simulation-analysis-based process.^74^ In that study, after columnar absorption under postcombustion conditions, a membrane contactor was modeled and included as a custom model in Aspen Plus, which approximated the complete process. Because no heat was required, the proposed model achieved a high regeneration efficacy with 30% less energy consumption than conventional thermal regeneration. Seo et al.^126^ developed a thin-film unit by rigorously simulating a regeneration unit, where a saturated IL descended as a thin film over the wall of a vertical bundle of tubes, which improved the heating efficacy, shortened the residence time, and reduced the thermal degradation of the IL. An economic optimization was conducted by considering the dimensions of the regeneration unit and the operating conditions, and the cost of the carbon-capture process was reduced from 61.1 to 50.1 $/t_CO2_ compared to the cost of thermal regeneration.

**Figure 21:**
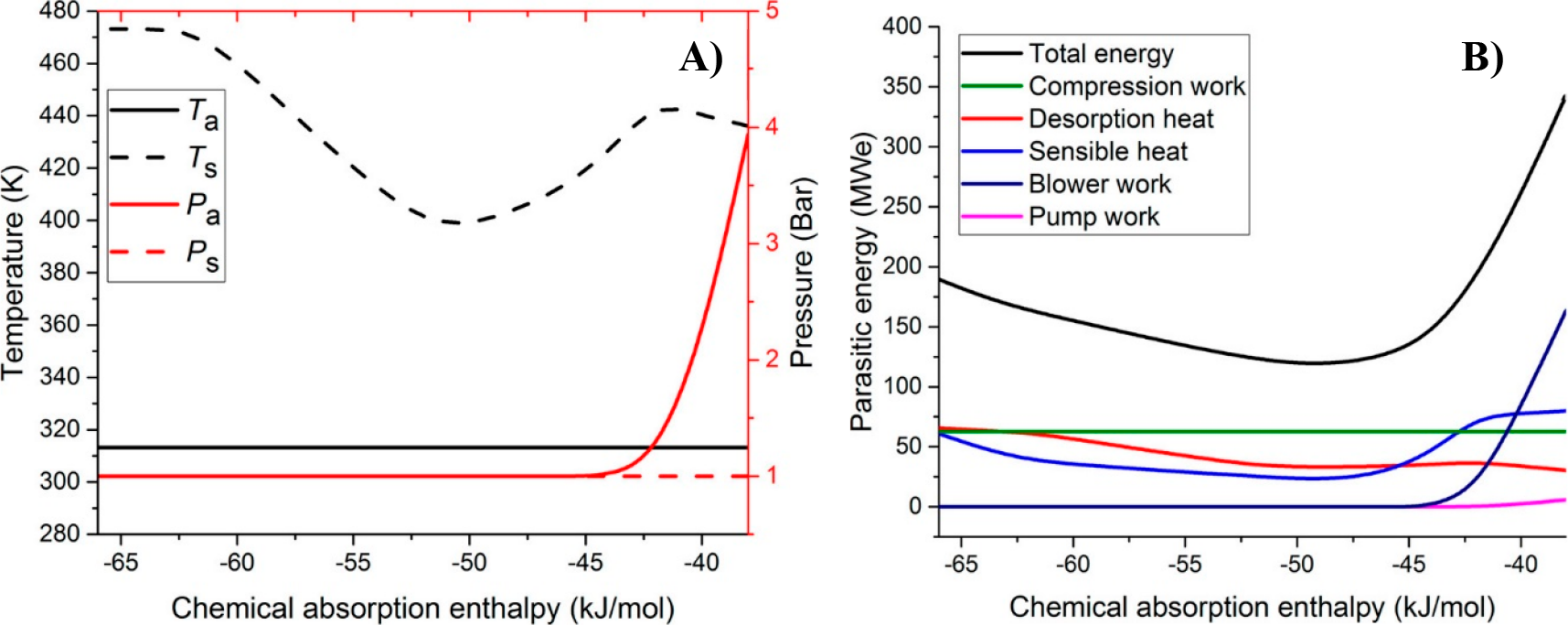
Optimized (A) absorber and stripper conditions and (B) parasitic energy and its components vs. reaction enthalpy of theoretical AHA-based IL under postcombustion conditions (at 111,453 kmol/h, 13.5% CO_2_, and 1 bar). Reproduced from ref (208). Copyright 2016 ACS.

A further step was the use of process simulations as a prospective tool for designing potential ILs possessing enhanced process performances for CO_2_ chemical absorption. To satisfy this objective, the IL properties were optimized at the process scale. In chemical absorption, aside from typical molecular and physical properties (MW, viscosity, heat capacity, density, etc.), one of the main properties that controls the CO_2_ uptake is the reaction enthalpy. The study published by Hong et al.^208^ focused on the optimization of the thermodynamic properties of the chemical reaction (enthalpy and entropy) to achieve a minimum in the parasitic energy at the capture process. Using a rigorous thermodynamic model without considering mass-transfer limitations, the authors optimized the complete CO_2_-capture process, including absorber and stripper conditions (equilibrium stages, P and T) and using a theoretical AHA-based IL to minimize the energy consumption as a function of the reaction enthalpy (Figure 21) and found that this parameter was crucial in the process performance. Although highly exothermic reactions required less IL, the stronger binding required higher temperatures in the regeneration step. On the contrary, lower reaction enthalpies required higher pressures during absorption to reach the desired recoveries. Finally, the authors found that for the ideal process performance, the optimal range was between −54 and −48 kJ/mol (Figure 21B).

In a later study, Seo et al.^117^ included the mass-transfer kinetic limitations using postcombustion (at 1 bar and 4.5% CO_2_) and the [P_2228_][2CNPyr] IL as a reference. In this case, the objective function is the total annualized cost (TAC) of the process using as decision variables the reaction enthalpy of the IL, in addition to the process variables; IL flow rate and absorber and stripper conditions (height, diameter, P and T). After that, a sensitivity analysis of the reaction enthalpy and physical properties (viscosity, molar volume, and heat capacity) on the TAC was performed around the optimum. The optimized reaction enthalpy was −54.1 kJ/mol (vs. – 45 kJ/mol for [P_2228_][2CNPyr]) and was highly impacted by the MW of the IL (see comparison of [P_2228_][2CNPyr] and [P_66614_][2CNPyr] in Figure 17). In a subsequent study, Seo et al.^118^ extended their analysis to higher CO_2_ inlet partial pressures. When the inlet CO_2_ concentration was increased from 4.5 to 60%, the optimal reaction enthalpy reduced to −43.4 kJ/mol, which increased the required regeneration temperature to 145 °C at 1 bar.

Although a desirable reaction enthalpy is advantageous for distinguishing ILs, this parameter cannot be freely manipulated during the design process. However, with desirable reaction enthalpy ranges in mind, additional AHA-based ILs can be sought with the aid of quantum chemical calculations for subsequent laboratory synthesis and characterization. Moya et al.^125^ proposed a methodology that combined quantum chemistry calculations with COSMO-RS solvation theory for fully predicting the CO_2_ absorption isotherms of AHA-based ILs based solely on theoretical information. This molecular simulation approach combined with process simulations^73^ could screen a tremendous number of ILs to find the most promising candidates, which is one of the formidable challenges in the development of such processes.

**Figure 22:**
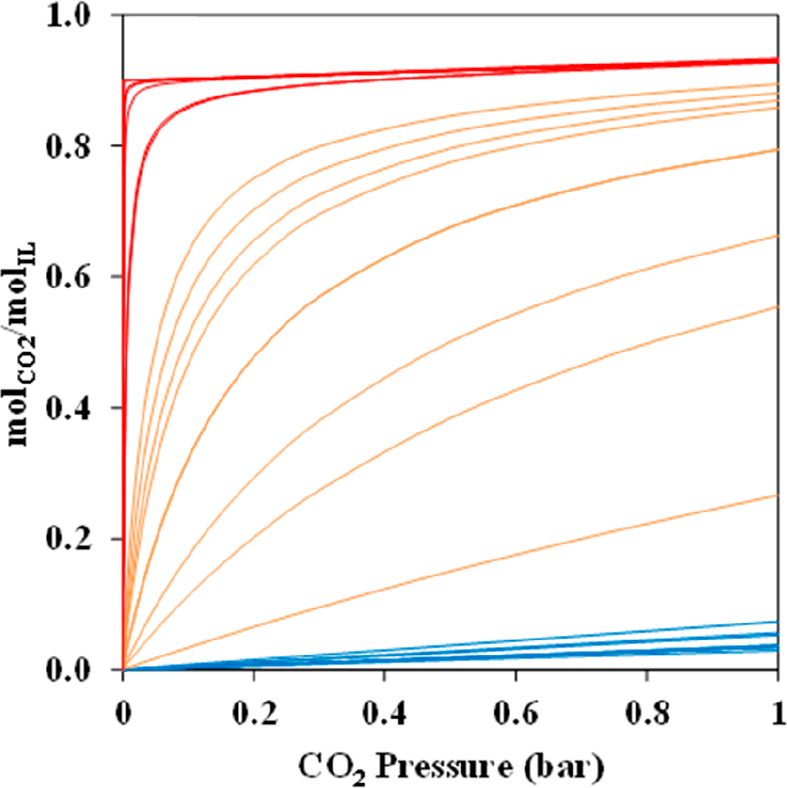
Theoretical DFT-calculation-based CO_2_ absorption isotherms. Red: nearly irreversible chemical absorption; orange: reversible chemical absorption; blue: physical absorption. Reproduced from ref (125). Copyright 2020 Elsevier.

A wide range of isotherms could be obtained using this methodology by tuning the AHA-based IL structure with different functional groups as ring substituents. Figure 22 shows a few representative examples ranging from ILs that cannot chemically bind with CO_2_ (ΔH_*R*_ < −30 kJ/mol, blue lines) to ILs that achieve complete saturation at very low CO_2_ partial pressures (ΔH_R_ > −50 kJ/mol, red lines). Using this theoretical approach, Hospital-Benito et al.^73^ evaluated 93 AHA molecular structures from eight anionic head groups functionalized with six substituents using a common [P_66614_] cation. Among the different head groups, highly exothermic reactions were observed with CO_2_ (up to −88.44 kJ/mol for [pyr]), which could be adjusted to the desirable range by different functionalizations. The substituent effect followed the same trend for all the different heterocyclic rings, from the methyl group having almost no effect on the reaction enthalpy, to the cyano group reducing the reaction exothermicity between 30 and 40 kJ/mol. Several proposed structures could not chemically bind with CO_2_ and were discarded. Finally, 12 AHA-based ILs were selected in the reaction enthalpy range between −30 and −65 kJ/mol and were evaluated using process simulations under different conditions.

**Figure 23:**
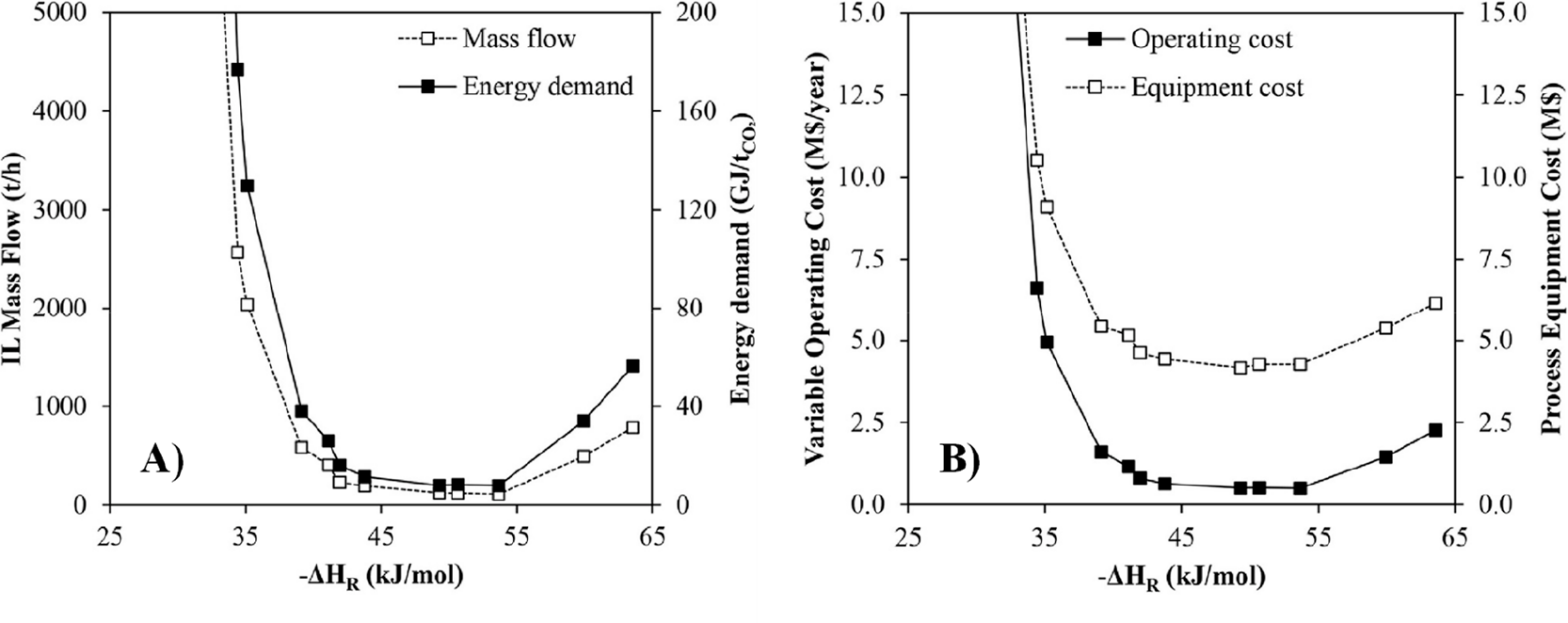
(A) Solvent requirements and energy demand and (B) variable and operating costs of CO_2_-capture process under postcombustion conditions (at 769.2 kmol/h, 13% CO_2_, and 1 bar) for proposed AHA-based ILs, depending on reaction enthalpy. Reproduced from ref (73). Copyright 2022 Elsevier.

The analysis conducted under postcombustion conditions, shown in Figure 23, provided all the minimum KPIs in the reaction enthalpy range between −45 and −55 kJ/mol. The [P_66614_][4BrPyra] IL, which had a reaction enthalpy of −49.3 kJ/mol, reduced the solvent and energy consumptions by almost half compared to those of the benchmark AHA ([P_66614_][2CNPyr]), which rendered the [P_66614_][4BrPyra] IL a good candidate and validated the results. These results fell within the range proposed by Seo et al.,^118^ where the optimal theoretical absorption enthalpy was −48.4 kJ/mol for postcombustion processes.

#### 4.2.4 Environmental Impacts Analysis of IL-Based CO_2_ Chemical Absorption Process

LCA has also been proposed as a tool for analyzing CO_2_ chemical capture. To the best of our knowledge, few LCA studies are available in the literature. Farahipour et al.^217^ and Cuellar-Franca et al.^177^ analyzed the [bmim][MeCOO] process previously published by Shifflet et al.^142^ Compared with traditional MEA absorption processes, [bmim][MeCOO] showed less carbon-mitigating potential, reducing the GWP of a coal power plant from 927 kg_CO2eq_./MWh to 328 kg_CO2eq_./MWh in the case of the amine and 468 kg_CO2eq_./MWh for the IL. For ILs, the switch in energy requirements from thermal to electrical energy imposed a penalty on the GWP. Additionally, the route for synthesizing ILs was far from optimized and much larger than that for producing MEA (except for its impact on human toxicity). Recently Hospital-Benito et al.^77^ simulated the complete process for producing hydrogen by steam reforming and evaluated the CCS unit using [bmim][MeCOO] and MDEA. Under these conditions, the LCA analysis revealed that the impact of the solvent production was 3.5 times higher for the IL than for MDEA, which indicated the need to search for more environmentally sustainable ILs. In contrast, in the IL-based CCS stage, the environmental impacts calculated for all the categories were lower than those obtained using the MDEA-based absorbent owing to the higher energy efficiency of the former, which could be improved by considering the expected lower IL losses owing to the higher stability.

##### 4.2.4.1 Other IL-Based Carbon Capture Processes by CO_2_ Chemical Absorption

**Figure 24:**
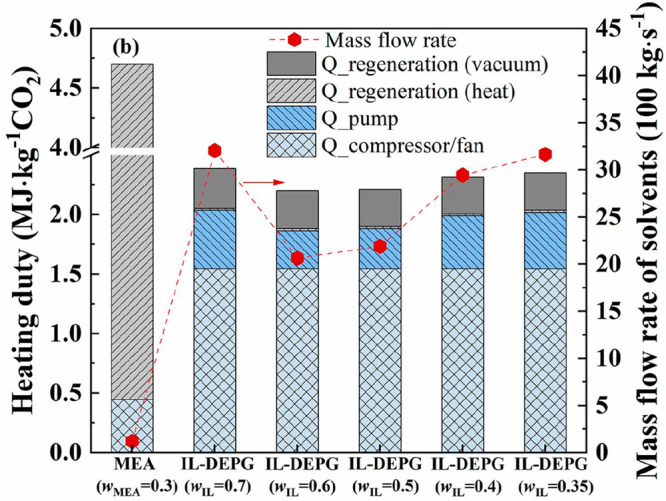
Heating duty and solvent requirements plotted as functions of DEPG content in postcombustion capture process (at 3,600 kmol/h, 15% CO_2_, and 1 bar). Reproduced from ref (169). Copyright 2021 Elsevier.

Reportedly, a different approach for improving the absorbent properties is the blending of ILs with another solvent to reduce the viscosity and improve the mass-transfer kinetics. This approach has been demonstrated as a valid alternative for designing CO_2_-capture processes. Water is one of the most used cosolvents because it is the least expensive green solvent and is already present in most gas streams subject to CO_2_ capture.^215^ Other options include the use of traditional physical CO_2_ absorbents, such as tetraglyme (TEG)^54^ or propylene carbonate (PC).^134^ The different solvents had different effects on the CO_2_ absorption capacity. Ma et al. experimentally studied the combined effects of different solvents with [bmim][MeCOO] on the CO_2_ absorption capacity.^169^ Water had the most notorious effect on the absorption capacity, reducing it by almost half when combined with 30% of the solvent. The use of traditional physical CO_2_ absorbents, such as PC or DEPG, on the other hand, increased the physical CO_2_ solubility, which implied higher amounts of CO_2_ were available in the fluid phase to react with the IL. The [bmim][MeCOO] + DEPG isotherms were modeled in NRTL and introduced to Aspen Plus simulations in a postcombustion capture process.^169^ The optimum ratio of 40 wt % DEPG (Figure 24) reduced the energy consumption from 4.7 to 3.1 MJ/kg_CO2_ for the MEA and IL-DMPG mixture, respectively. The total cost was 72 $/_tCO2_, which was 10% less than that of the MEA process under the same conditions (81 $/t_CO2_).

Ma et al.^215^ modeled a series of morpholinium acetate-based ILs and their mixtures with water in a biogas upgrading process and compared them to models of traditional solvents (PC and Selexol). A complete biogas separation process was simulated using an equilibrium approach by adjusting the solvent flow and flash pressure for efficiently removing all the absorbents. The authors found that the mixture comprising [bmmorp][MeCOO] + 20 wt % H_2_O substantially reduced the solvent and energy requirements. In a subsequent process optimization, Honglin et al.^135^ simulated the process by considering the mass-transfer limitations, different heat-transfer integrations, and recovery of waste heat from other sources to optimize the economics of different scenarios and calculated a reduction of 25% in the biogas cost with respect to water scrubbing for a final cost of 0.15 $/Nm^3^. In a study using the tetraglyme (TG) solvent and [bmim][MeCOO] and [P_66614_][2CNPyr] ILs, Hospital-Benito et al.^54^ simulated a series of processes for a packed absorption column at a fixed temperature and different pressures and TG concentrations and calculated the amount of solvent required to remove 90% of the CO_2_. The authors found that 25 wt % TG was optimal for reducing the amount of required solvent by improving the transport properties of the mixture.

**Figure 25:**
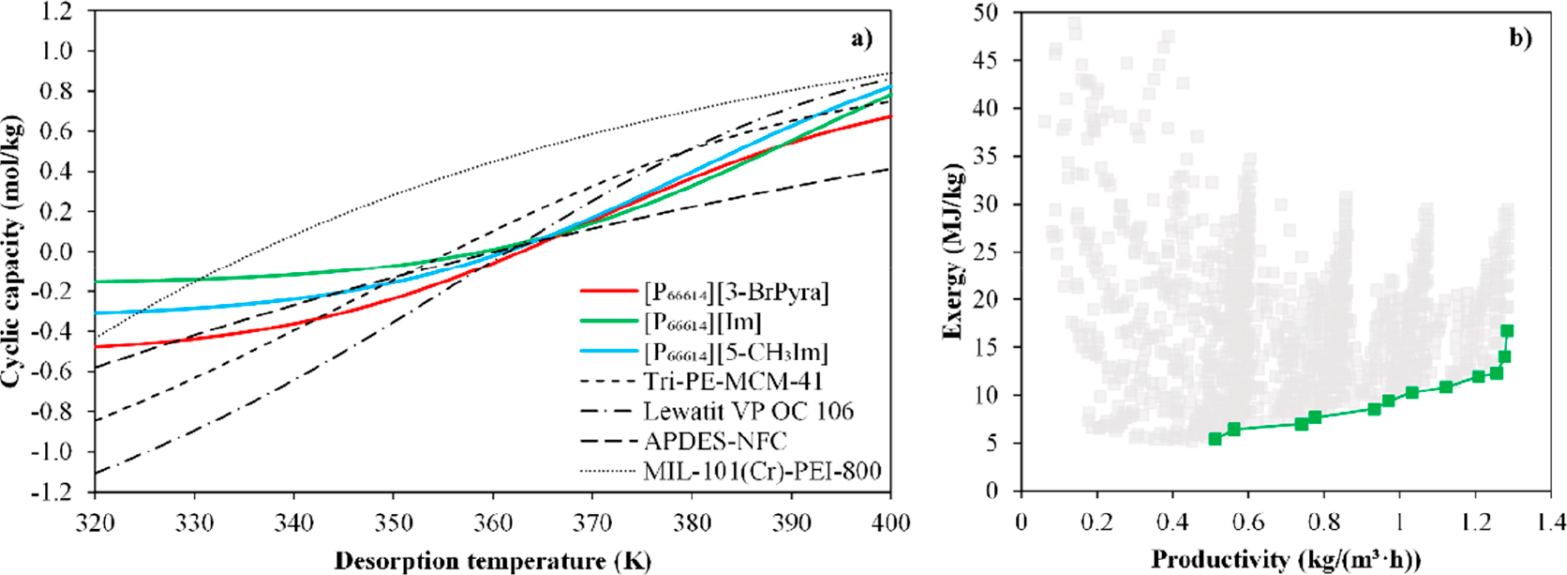
(A) Cyclic capacity of ILs plotted as function of desorption temperature and (B) exergy vs. productivity of Pareto front for [P_66614_][Im]-based DAC process. Reproduced from ref (68). Copyright 2023 Elsevier.

Recently, the use of ILs for DAC has been addressed by Hospital-Benito et al.^68^ by connecting their material design to process modeling through molecular simulations. With respect to the cyclic operation capacity, the most promising ILs, for which the capacities were comparable with those of other materials evaluated for application to DAC (see Figure 25A), were assessed using process simulations in Aspen Plus. A wide range of operating configurations were screened by modifying the air velocity (1–3 m/s), IL mass flow (5–50 t/h) and temperature (20 °C–50 °C), and regeneration pressure (0.1–1 bar) and temperature (100 °C–120 °C). The authors computed the exergy, energy, and productivity to optimize the operating conditions by representing the Pareto front (see Figure 25B) and conducted a simplified economic analysis to highlight the major cost components. The [P_66614_][Im]-based DAC system exhibited a minimum exergy of 5.44 GJ/t, which was slightly better than that of alkali scrubbing (6.21 GJ/t) and in line with that of amine scrubbing (5.59 GJ/t) for achieving a similar productivity. Moreover, the assessed DAC process could operate at approximately 200 $/t_CO2_ and had reasonable energy and plant expenses.^68^

In conclusion, process simulations offer great opportunities for designing CO_2_ chemical capture processes, enabling preliminary technoeconomic evaluations to be conducted to compare the designed processes with actual technologies. However, in process simulations, chemical absorption presents formidable challenges for developing proper methodologies for adequately describing chemical reactions and the possible changes in the solvent properties owing to the reaction media. Although quantum chemistry has aided in the description of chemical equilibria, it is currently limited to only a few systems. In addition, the effects that some impurities, especially water, have on chemical reactions have hardly been described, especially considering that few experimental data are available for those systems. Even with those limitations, the preliminary results show that IL-based chemical CO_2_ capture is a promising alternative to traditional technologies for describing absorption operations.

### 4.3 Carbon Conversion

As the next step after CO_2_ capture, ILs have been experimentally proved as efficient catalysts for CO_2_ conversion.^27^ However, despite the availability of many experimental studies, process simulation studies on this topic are scarce, as summarized in Table 4. Experimental studies on CO_2_ valorization have focused on the use of ILs as catalysts to obtain products containing C–O bonds,^27^ and this applies to the literature for process simulations, as listed in Table 4. The available literature for simulated IL-based CO_2_ conversion processes can be grouped according to the obtained products. Although the most studied products are cyclic carbonates and their derivatives, diethyl carbonate, formic acid, and methanol have been also evaluated.^218,219^ Although ILs can also be used as electrolytes in electrochemical reactions, only one process-scale example involving electroreduction from CO_2_ to CO could be found.^220^

**Table 4:**
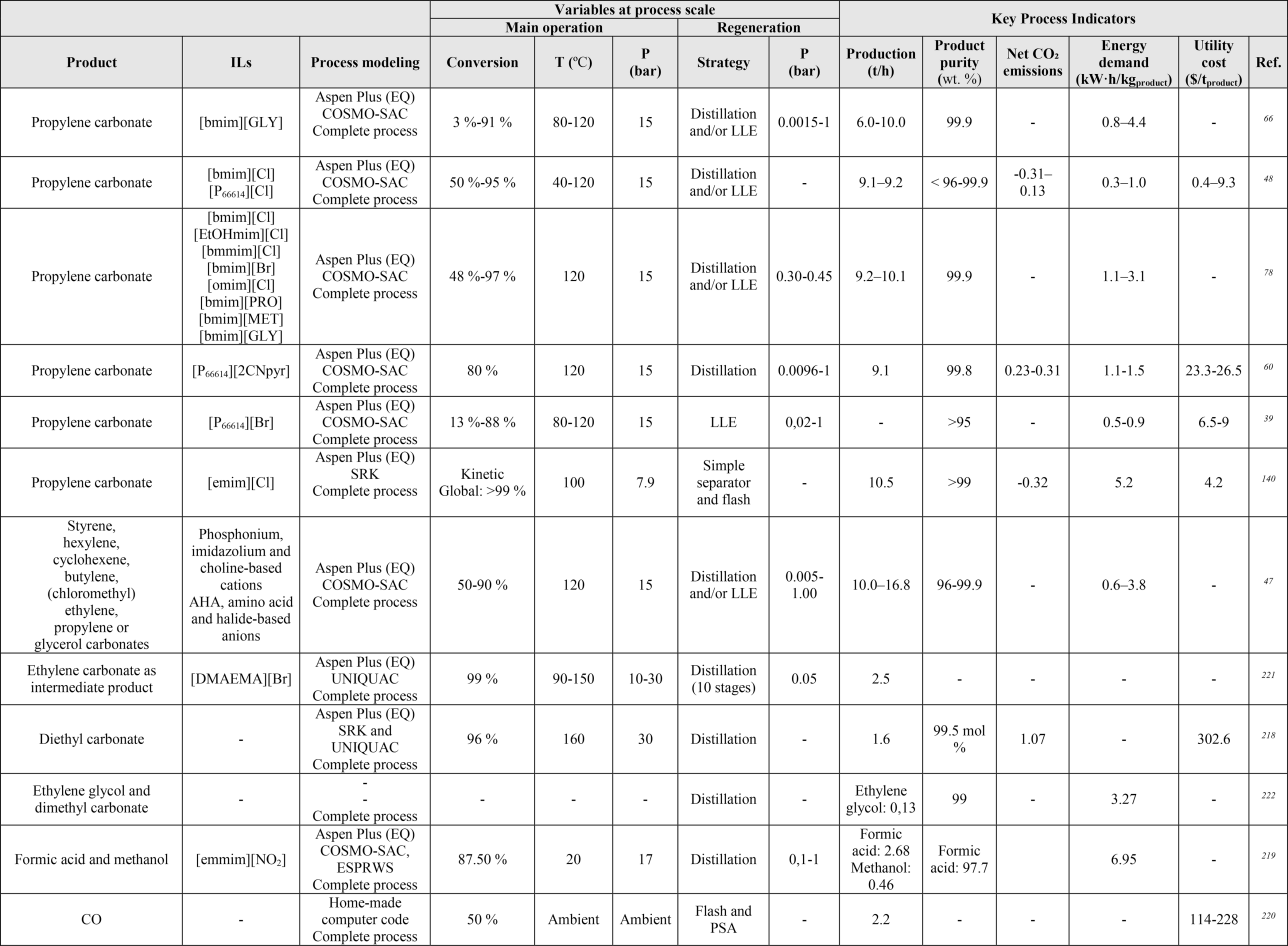
Main Process Indicators of Different Simulation Studies Involving IL-Based CO_2_ Conversion^a^

a For unknown cases, data were estimated assuming operating time of 8,000 h/year.

**Scheme 5:**
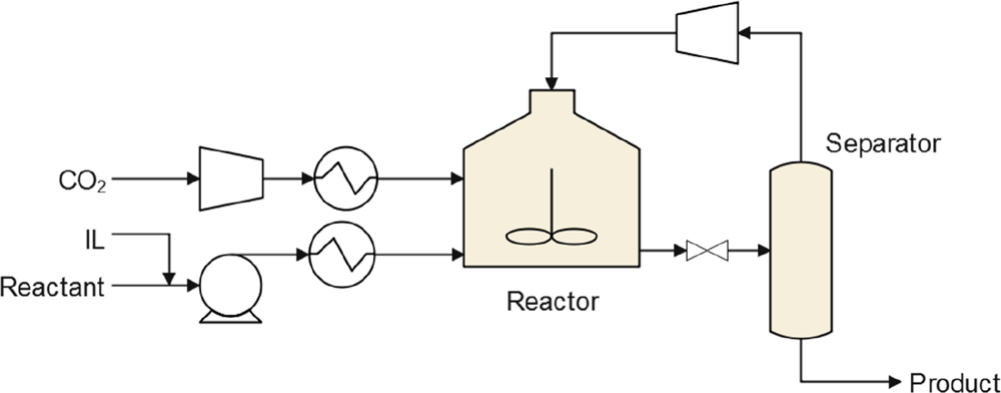
Typical Flowsheet of IL-Based CO_2_ Conversion Process

The typical process diagram followed in these studies is shown in Scheme 5. The IL, CO_2_, and reactant/s are usually conditioned prior to being fed into the reactor. Different reactor designs are found in the literature, from rigorous designs, including kinetics, to simplified models specifying experimental or benchmark conversions. Afterward, the unreacted reactants are separated from the product and recycled back to the reactor. Additionally, further separations are often required for IL regeneration and product purification. Once again, the separation section shows different levels of refinement and complexity, depending on the compounds that are present. In addition, some products obtained directly from this processing scheme are further transformed into other products of greater interest.^221,222^ The main contributions of process simulation studies in this field are focused on calculating the energy demands, costs (centered on utility costs), and environmental impacts of the production of different compounds from CO_2_.^9,48^ In addition, some process variables that can provide guidelines for designing optimal IL catalysts have also been studied either for their contribution to the reaction or their easy separation and reuse.^47,78^

#### 4.3.1 Organic Carbonate Production

**Figure 26:**
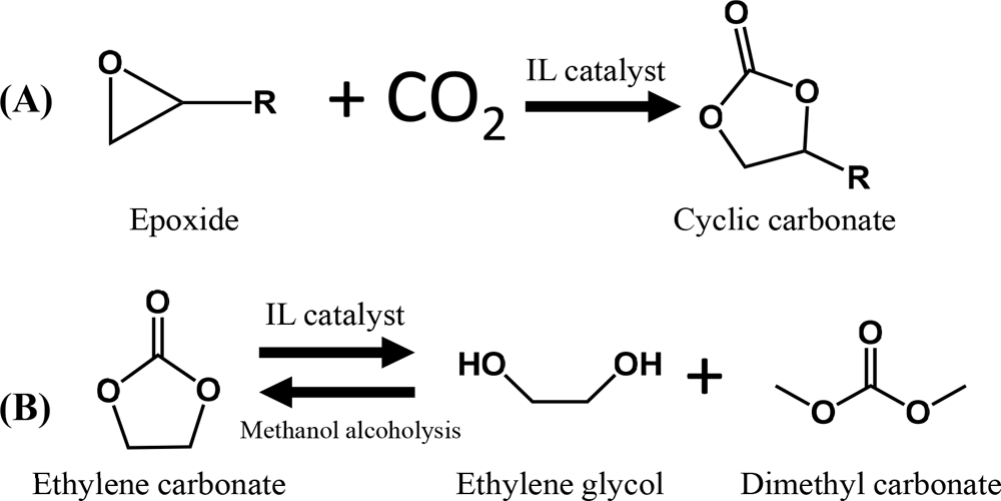
(A) IL-catalyzed synthesis of cyclic carbonates from CO_2_ and epoxides and (B) subsequent transformation from ethylene carbonate to ethylene glycol and dimethyl carbonate.

As mentioned, organic carbonates, especially cyclic carbonates, are among the CO_2_ conversion products that have been the most studied using process simulations. The literature on these products is diverse in terms of the studied variables and number of available studies, as listed in Table 4. Cyclic carbonates can be synthesized from epoxides and CO_2_ using an IL catalyst,^48,66^ as shown in Figure 26A. In addition, other value-added products can be generated utilizing these CO_2_-derived cyclic carbonates as reactants. For instance, ethylene carbonate can be further transformed into ethylene glycol and dimethyl carbonate via trans-esterification (Figure 26B). This subsequent reaction, originally modeled by Tian et al.,^223^ also employs an IL catalyst.

**Figure 27:**
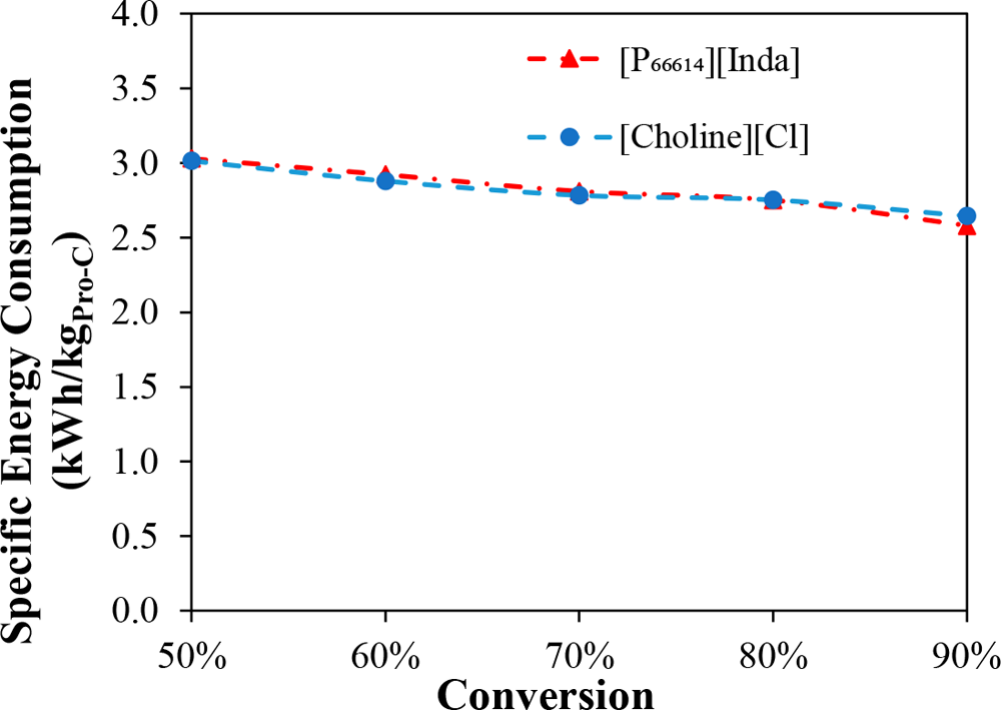
Specific energy consumption of CO_2_ conversion processes plotted as functions of CO_2_ conversion for two ILs. Reproduced from ref (47). Copyright 2022 Elsevier. Reaction was modeled at 120 °C and 15 bar in RSTOIC unit for molar ratios of 200:1 and 1:1 for epoxide:IL and epoxide:CO_2_, respectively, and separation conducted by distillation (similar to that shown in Scheme 6A).

**Figure 28:**
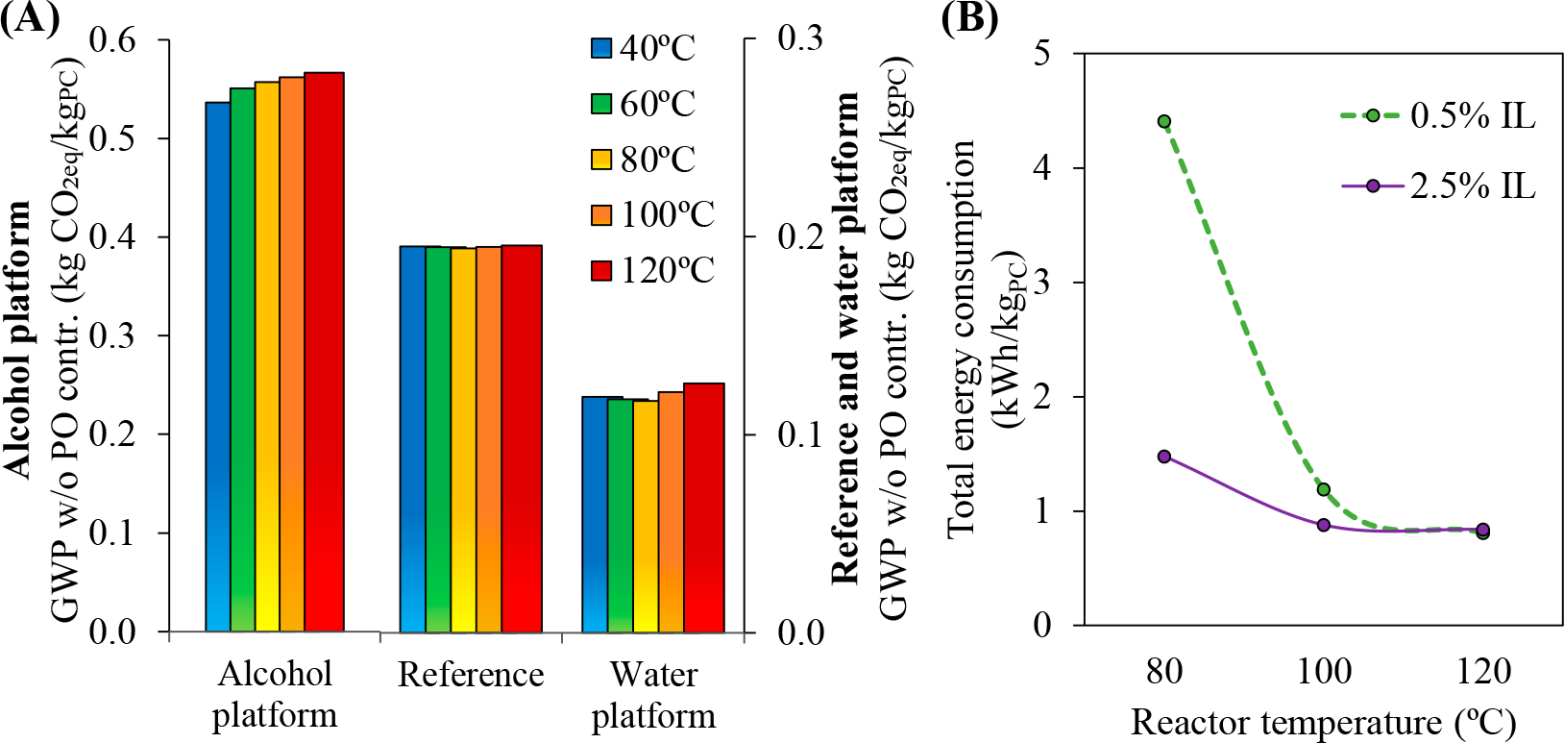
Influences of reaction temperature on (A) global warming potential for three conversion process using distillation and liquid–liquid extraction (fatty alcohol and water) platforms. Reproduced from ref (48). Copyright 2022 Wiley. (B) Energy consumption for water platform. Reproduced from ref (66). Copyright 2021 Elsevier. In both cases, reaction was conducted at 15 bar in RSTOIC model for molar ratio of 1:1 epoxide:CO_2_. In (A), the epoxide:IL molar ratio was 200:1.

For producing cyclic carbonates, the process diagram is similar to the one shown in Scheme 5. Although the different process simulation studies usually only slightly differed in the conditioning of the raw materials that were used, they did diverge in the description of the CO_2_ conversion reaction. Some authors opted to use a conversion reactor (RSTOIC model in Aspen Plus) with an experimental or benchmark value^66,78^ because in some cases, either the conversion was arbitrarily set^221^ or the process behavior was studied according to this variable.^47,48^ In the latter case, when the conversion was varied from 50 to 95%,^47,48^ a higher conversion rate reduced the energy consumption of the overall process owing to a lower recirculation flow rate, as shown, for example, in Figure 27, where the specific energy consumption decreased from 3.0 to 2.6 kWh/kg_product_ when the conversion increased from 50 to 90%. Moreover, this fact was independent of the selected IL. The study by Demirel et al.^140^ was noteworthy because an Arrhenius-type kinetic equation —acquired from a previous experimental study—^224^ was used to describe the [emim][Cl]-catalyzed CO_2_ conversion. Studies on the influence of the reactor temperature on the process, on the other hand, are scarce. Hernández et al.^48^ recently conducted a sustainability study and concluded that for the same conversion between 40 and 120 °C, a lowertemperature was not always beneficial for the energy demands and sustainability, as shown in Figure 28A, which contradicted the conventional criteria for designing catalysts to operate at mild temperatures. However, a previous study has shown that higher temperatures (120 °C versus 80 °C) could be balanced by increasing the IL load in the reactor to ensure the same conversion without altering the energy consumption,^66^ as shown in Figure 28B. Additionally, for a high catalyst loading (2.5 mol % of IL), the energy demand of the process shifted less with changing temperature (1.5 and 0.8 kWh/kg_product_ at 80 and 120 °C, respectively) compared with the shift in the energy demand of the process for a low catalyst loading (0.5 mol % of IL) (4.4 and 0.8 kWh/kg_product_ at 80 and 120 °C, respectively). As listed in Table 4, on the other hand, the reactor pressure is usually not studied and is maintained between 8 and 30 bar. This is presumably because the most studied product is PC, which is derived from the very volatile compound propylene oxide. Therefore, at high temperatures, high pressures are also necessary to operate in the liquid phase. However, this presents a clear challenge in process simulations involving IL catalysts for converting CO_2_ to organic carbonates.

**Scheme 6:**
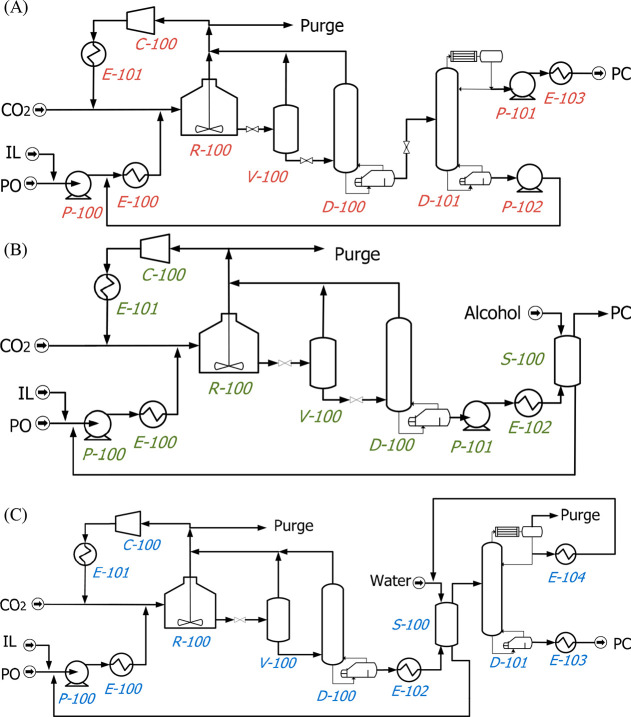
Different Process Flow Diagrams for Producing PC from CO_2_ Using Only (A) Distillation or Distillation and Liquid–Liquid Extraction Using Different Solvents: (B) Fatty Alcohol and (C) Water. Reproduced from Ref (48). Copyright 2022 Wiley.

During the separation of the reaction mixture, major differences begin to emerge, as depicted in the three most differentiated flow diagrams for this process in Scheme 6. Distillation isthe most common strategy for separating the reactants, which are compounds typically more volatile than carbonates. In some cases, distillation was also used to separate the carbonate from the IL. Additionally, this separation process presented different levels of rigor, from flash operations—operating under vacuum conditions—^140,222^ to distillations^218^ or both combined.^66,221^ Thus, Scheme 6A depicts the most common strategy, where the CO_2_ and volatile reactants are separated using a flash and/or distillation and then the carbonate is purified, and the IL is simultaneously regenerated through another distillation. In some studies, on the other hand, the IL/product separation was neither detailed^218,221^ nor necessary because the IL was subsequently used together with the product.^222^ However, Hernández et al. found that owing to the high boiling point of the carbonates and thermal stability of the ILs, the separation of the carbonates from the ILs via vacuum distillation was very energy intensive.^66^ Other authors have supported this finding for producing ethylene glycol and dimethyl carbonate from ethylene carbonate, where the distillation columns were the units that consumed the most energy in the process.^9^ Thus, alternatively, liquid–liquid extraction has been proposed for separating the ILs from the carbonates.^57,66^Scheme 6B,C depicts the flowsheet related to this separation approach, where different extraction solvents can be used, and their selection determines the final carbonate purity.

**Figure 29:**
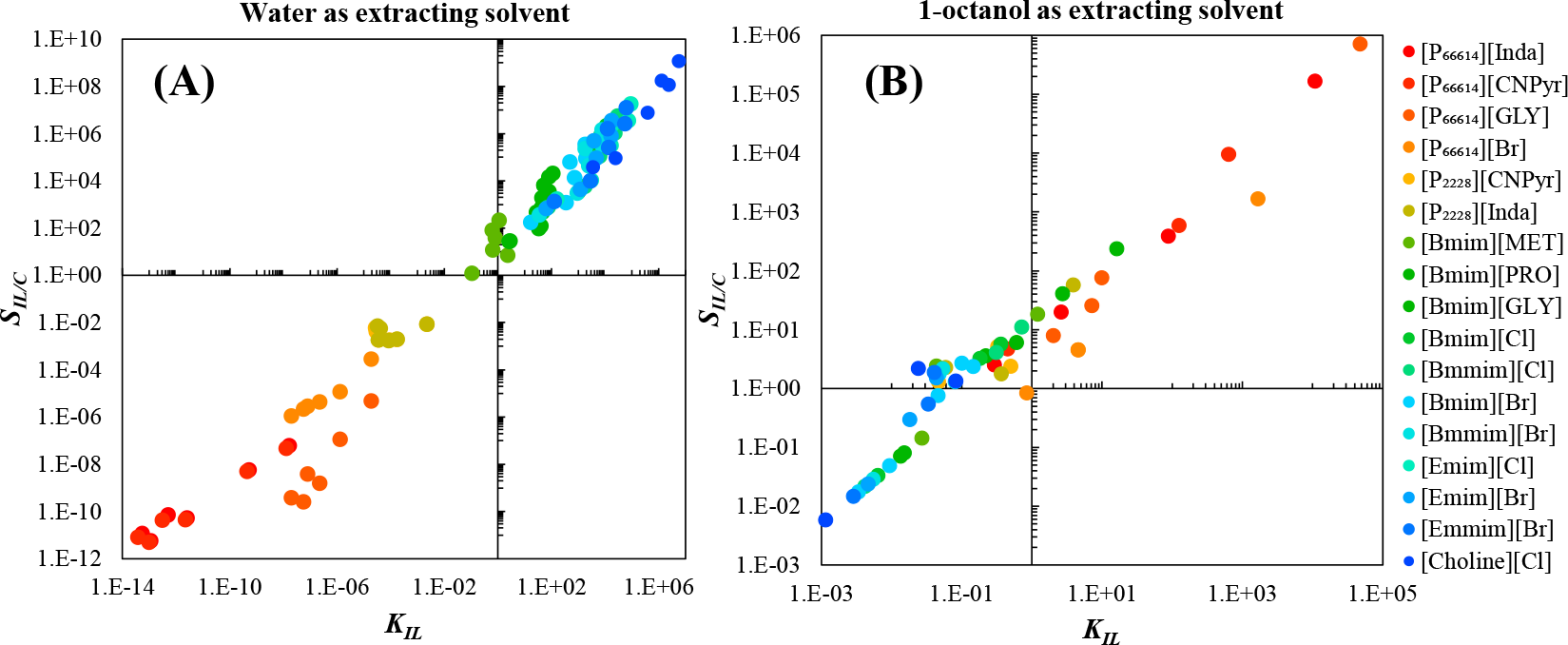
IL/Carbonate selectivity versus IL distribution ratio for different ILs when water or 1-octanol was used as extracting agent. Reproduced from ref (47). Copyright 2022 Elsevier. Single-stage liquid–liquid extraction model (DECANTER) calculated at 1 bar and 25 °C for cyclic carbonates and 1-octanol mixed in equimolar ratio.

**Figure 30:**
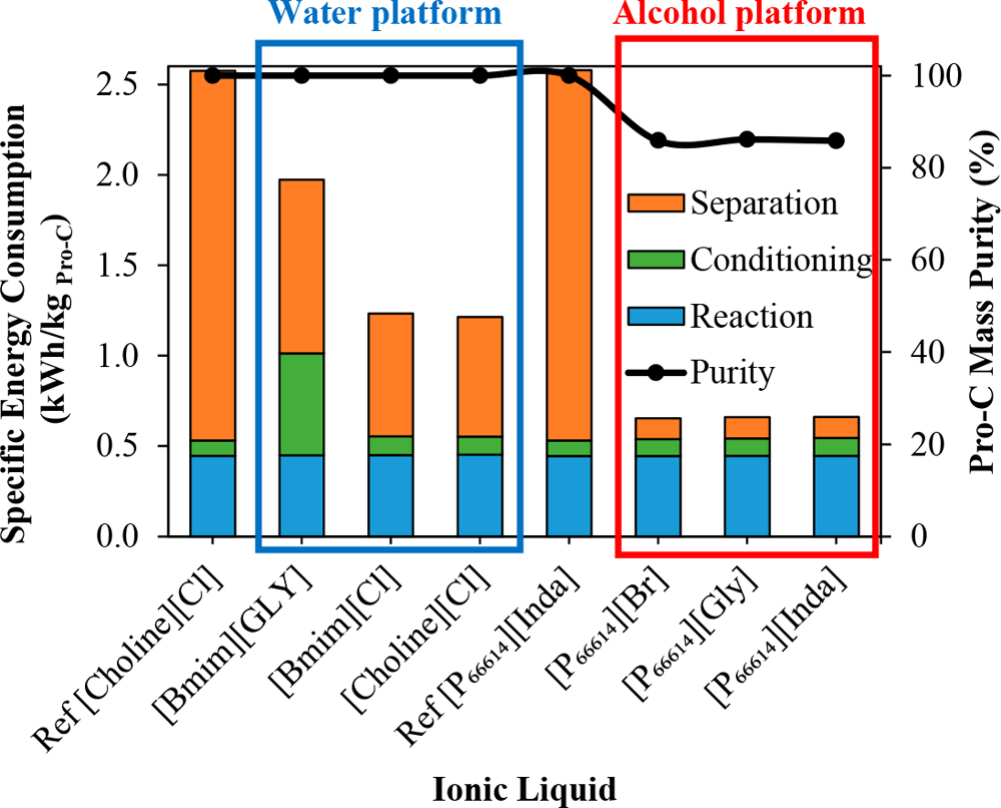
Specific energy consumption and PC (Pro-C) mass purity (%) plotted as functions of IL for distillation-based process, water-based platform, and alcohol-based platform (with 1-decanol as solvent). Reproduced from ref (47). Copyright 2022 Elsevier. Reaction performed at 120 °C and 15 bar in RSTOIC model at fixed epoxide conversion of 90%, using molar ratios of 200:1 and 1:1 for epoxide:IL and epoxide:CO_2_, respectively.

Regarding liquid–liquid extraction as a carbonate/IL separation strategy, the use of water as extracting solvent was first reported as an efficient and sustainable method for recovering and reusing hydrophilic IL catalysts^66,47,78^ following the flowsheet shown in Scheme 6C. Then, Belinchón et al.^47^ found that long-chain alcohols were a suitable class of extracting solvents for separating hydrophobic ILs from cyclic carbonates (Scheme 6B), while water remained the most useful solvent for extracting hydrophilic ILs.^47,78^ Hence, the three separation strategies or platforms could be differentiated as follows: distillation (Scheme 6A), fatty-alcohol-based LLE (Scheme 6B), and water-based LLE (Scheme 6C). In the case of liquid–liquid extraction strategies (Scheme 6B and 6C), both fatty alcohols and water exhibit lower densities than cyclic carbonates, leading to an overhead stream containing the extracting solvent and the ionic liquid, along with a carbonate-rich bottom stream. For a wide range of ILs, the extractive properties of water and fatty alcohols were obtained based on process simulations using a single equilibrium-stage decanter and a S/F mass ratio of 0.5 and 0.5 mol % of IL in the carbonate feed stream (Figure 29). The remarkably high IL/carbonate selectivity and IL distribution ratio revealed that the proposed water/fatty alcohol platforms were suitable separation strategies for efficiently recovering hydrophilic and hydrophobic ILs, respectively. The higher the hydrophilicity of the ILs, the better their extractive properties were with water (with K_IL_ and S_IL/C_ values of 10^6^ and 10^10^, respectively), and the same trend applied to the hydrophobicity of ILs and their extractive properties with fatty alcohols (with K_IL_ and S_IL/C_ values reaching 10^6^ and 10^9^, respectively, for the most hydrophobic alcohols). This behavior affected the energy consumption of the process, as shown in Figure 30. Clearly, distillation-based processes implied substantially higher energy demands for producing PC than the extraction-based separation train. However, the better the extractive properties of the IL, the lower the energy demands and the higher the product purity; for example, selecting [choline][Cl] instead of [bmim][GLY] or selecting [P_66614_][Inda] rather than [P_66614_][Br], results in lower energy demands, because [choline][Cl] and [P_66614_][Inda] presented the highest S_IL/PC_ and K_IL_ values with water and 1-octanol, respectively, as shown in Figure 29. In contrast, the distillation process-related energy consumption was nearly independent of the IL. Additionally, process simulations have enabled the evaluation of energy consumption distributions according to the conditioning, reaction, or separation (Figure 30). Clearly, separation contributed the most to both the water platform and distillation-based process, followed by the reactor cooling. For the alcohol platform, on the other hand, the separation minimally contributed to the energy consumption, which implied substantially lower global energy requirements than those of the water-based platform. Clearly, for the conversion from CO_2_ to PC, the reaction contribution to the global energy consumption remained nearly constant for the three proposed process configurations because the conversion was fixed at 90%.

**Figure 31:**
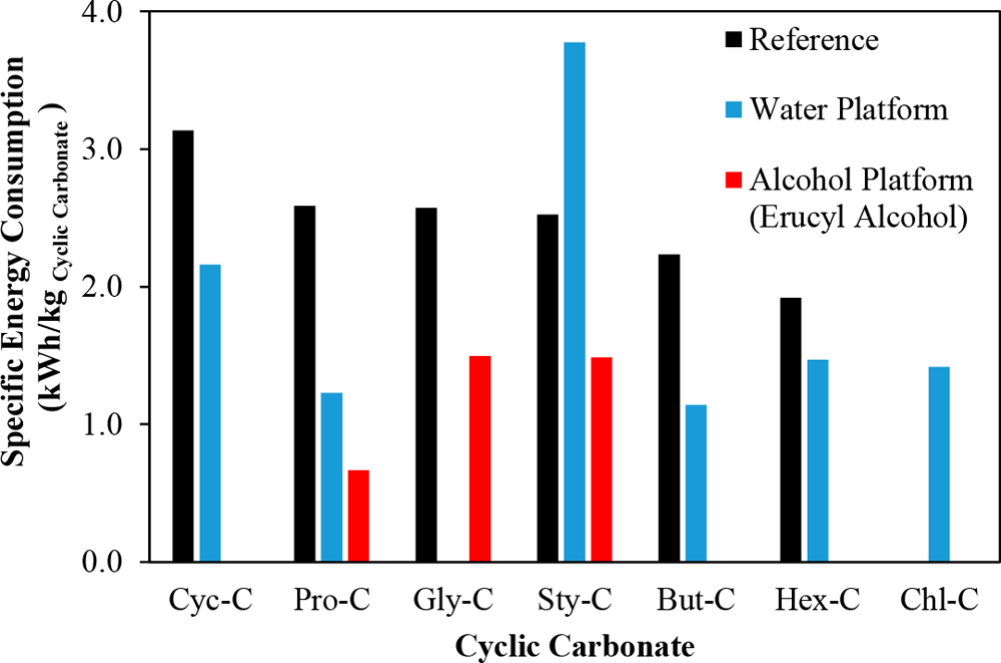
Energy consumption to produce different carbonates using distillation as separation strategy or liquid–liquid extraction with different solvents. Reproduced from ref (47). Copyright 2022 Elsevier. Reaction performed at 120 °C and 15 bar in RSTOIC model with fixed epoxide conversion of 90% and molar ratios of 200:1 and 1:1 for epoxide:IL and epoxide:CO_2_, respectively.

Furthermore, the hydrophilicity of cyclic carbonates varied based on the functional groups they possessed, requiring distinct LLE-based separation approaches to achieve optimal results. Process simulations have revealed that the production of hydrophilic carbonates (glycerol carbonate, PC, etc.) was optimized by employing a hydrophobic IL catalyst and fatty alcohol extraction solvent. The production of hydrophobic carbonates (such as styrene or hexylene carbonate), on the other hand, was optimized when a hydrophilic IL and water solvent were utilized.^47^ Using liquid–liquid extraction, the authors reduced the energy consumption in the range 23–74%, depending on the carbonate, compared with the energy consumed during separation by distillation, as shown in Figure 31. Again, process simulations could guide the selection of ILs as a function of the product and its separation from the IL, imposing additional criteria for experimental developments.

Recently, Hernández et al.^39^ have developed an iterative methodology that combined experiments and process simulations to exploit process simulations and obtain more representative experimental data and, thus, synergistically improve the simulation rigor. The initial experimental study of the different reaction conditions (temperature and presence of fatty alcohols and water) was performed using [P_66614_][Br] to determine the optimal combination of the solvent and catalyst for converting CO_2_ to PC. Simulations were then conducted to optimize the solvent amounts in the different parts of the process, enabling lower energy consumption. Afterward, the experimental reactions were successively conducted for converting CO_2_ under these conditions to combine computational and experimental tools and efficiently improve the process. In fact, the final iteration enabled the process simulation conditions to be emulated in the reactor, enabling ad hoc experimental validation. Owing to this technique, two strategies were identified for reducing energy consumption during the separation process. First, the amount of alcohol required to recover the IL could be minimized without compromising the recovery effectiveness. Additionally, in the separation stage, water reduced the energy and vacuum requirements. The study conducted by Hernández et al.,^39^ on the other hand, rigorously addressed the simulation, including the byproducts, which imposed a more challenging step in the process engineering but showed the potential of process simulations for describing complex processes involving byproducts.

As anticipated from the data listed in Table 4, although neither balancing CO_2_ emissions nor calculating utility costs have been common, the norm has been to calculate energy consumption. Table 4 lists the main performance indicators of different studies involving the production of carbonates from CO_2_. Clearly, most studies have focused on PC because it is a suitable commercially available benchmark carbonate. Additionally, the study conducted by Belinchón et al.^47^ revealed that the same flowsheet could be applied to the production of different carbonates, including minor modifications owing to the different physical properties of the carbonates, especially the boiling points. In most cases, the typical process capacity was approximately 10 t/h, and the processes had energy consumptions, CO_2_ emissions, and utility costs that were within the same ranges. Nevertheless, the most recent studies have reported a substantial reduction in energy consumption, namely those processes that use liquid–liquid extraction as an IL separation strategy.^47,48,66,78^

Regarding the cost calculation, only four studies calculated the utility costs (for producing propylene and diethyl carbonates),^39,48,140,218^ and only two examples of detailed capital costs (for producing PC) were available in the literature.^48,140^ For the utility costs calculated for producing PC, the values obtained in these studies were consistent and in the same range (0.4–9.3 $/t_product_).^39,48,140^ The utility costs for producing diethyl carbonate, on the other hand, were considerably higher. When ILs were used, the utility costs amounted to 303 $/t_product_,^218^ which was similar to that of other technologies that do not involve IL (e.g., the utility cost for producing dimethyl carbonate with catalytic membrane reactors was 209 $/t_product_).^225^ For capital costs, Hernández et al. estimated that the direct cost of the equipment used for producing PC was between M$2.4 and M$3.3,^48^ while Demirel reported this value at M$18.2,^140^ which was substantially higher, at a similar production rate of approximately 10 t_product_/h, as listed in Table 4.

**Figure 32:**
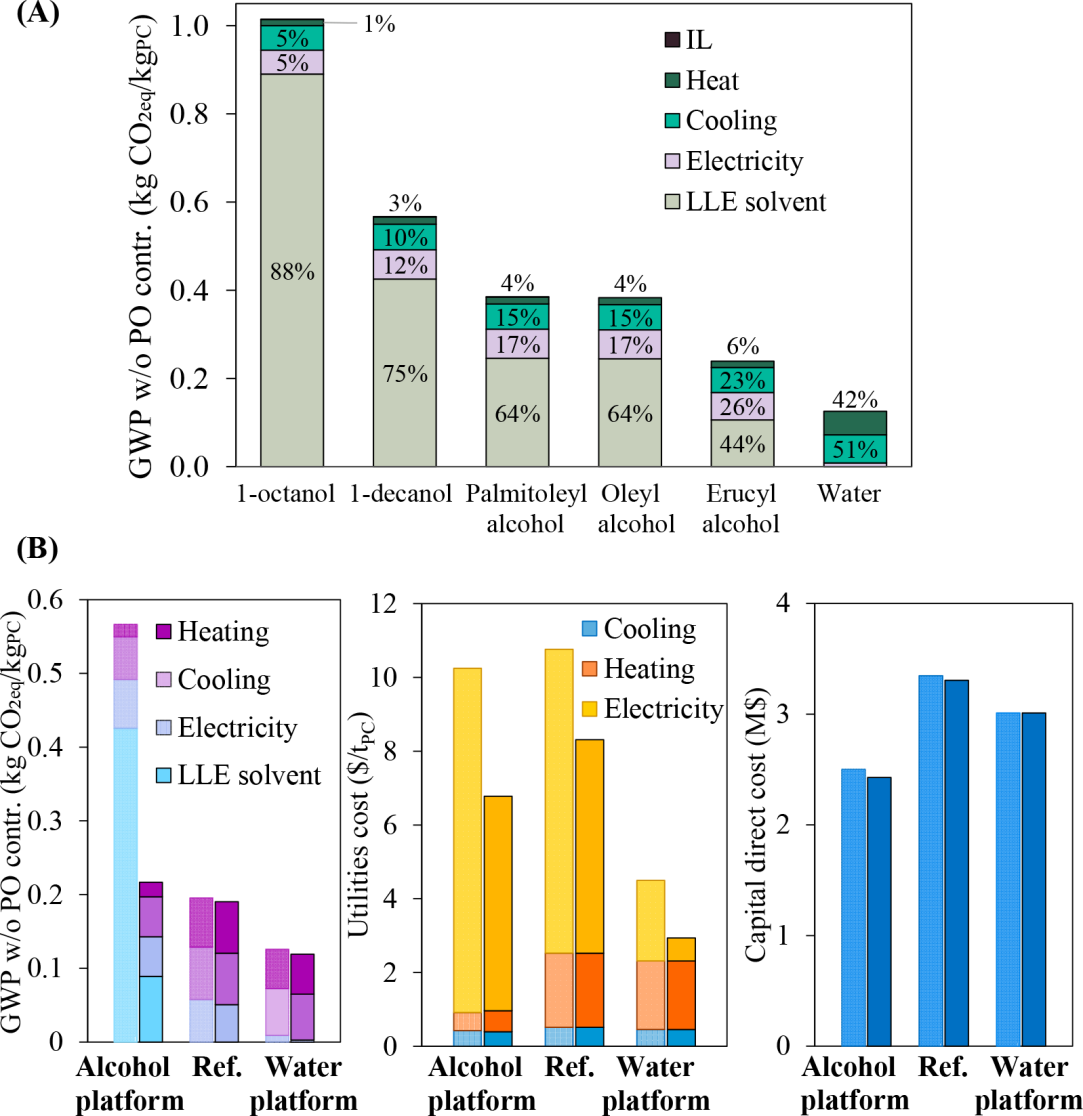
(A) Influence of different solvents in LLE-based separation strategies on GWP to produce 1 kg of PC. The reaction was conducted at 15 bar in RSTOIC model for molar ratios of 1:1 and 200:1 for epoxide:CO_2_ and epoxide:IL, respectively. (B) Environmental and economic indicators of three CO_2_ conversion processes before (left, soft colors) and after (right, dark colors) optimization of environmental impact. Reproduced from ref (48). Copyright 2022. Wiley.

With respect to sustainability analyses, process simulation enables their performance, which is a very important factor because CO_2_ balances are especially relevant in these processes. When sustainability is analyzed, utility-related CO_2_ emissions are usually considered but not the emissions associated with the synthesis of raw materials or ILs. For the first time, Hernández et al.^48^ evaluated the IL-related environmental impact at the process scale and discovered that when an effective IL recovery was designed, the impact of the IL on the environment was negligible. In addition, the combination of process simulations and life-cycle assessment tools has enabled the identification of sustainability-related key points in these processes. Although the use of fatty alcohols in LLE-based processes led to the lowest energy consumption, these processes exhibited high CO_2_ emissions mainly due to the synthesis of these solvents, accounting for 44–88% of the contribution of CO_2_ emissions to the GWP, as shown in Figure 32A. Propylene oxide (the main reactant studied in these processes), on the other hand, presented high synthesis-related CO_2_ emissions, rendering impossible the design of a positive-balance CO_2_-conversion process in which propylene oxide was the reactant, hence, the necessary search for alternative, more sustainable reactants. Thus, when a fatty alcohol was selected as a solvent, the key parameters were the raw materials, CO_2_ conversion, and S/F ratio.^48^ Finally, an interesting conclusion of the sustainability study was that improvement in the environmental friendliness of the processes was also associated with improved energy and economic indicators, as shown in Figure 32B, which revealed how process-associated CO_2_ emissions were reduced while simultaneously reducing the utility and equipment costs.

#### 4.3.2 Production of Other Compounds: CO, Formic Acid, and Methanol

In addition to carbonates, the evaluation of CO_2_-derived products synthesized using ILs has been limited to CO,^220^ formic acid, and methanol (Table 4).^219^ Both processes followed base flowsheets similar to the one shown in Scheme 3 and focused on economic evaluation. These studies assessed the impacts of the capital costs on the product pricing. For CO, the selling price ranged from 0.47 to 5.82 $/kg_CO_,^220^ while for formic acid, it was between 0.9 and 1 $/kg_FA_.^219^ For perspective, in carbonate production, the operating cost for producing CO previously ranged from 171 to 1,085 $/t_CO_, which was considerably higher than the operating cost for producing carbonates (Table 4).

**Figure 33:**
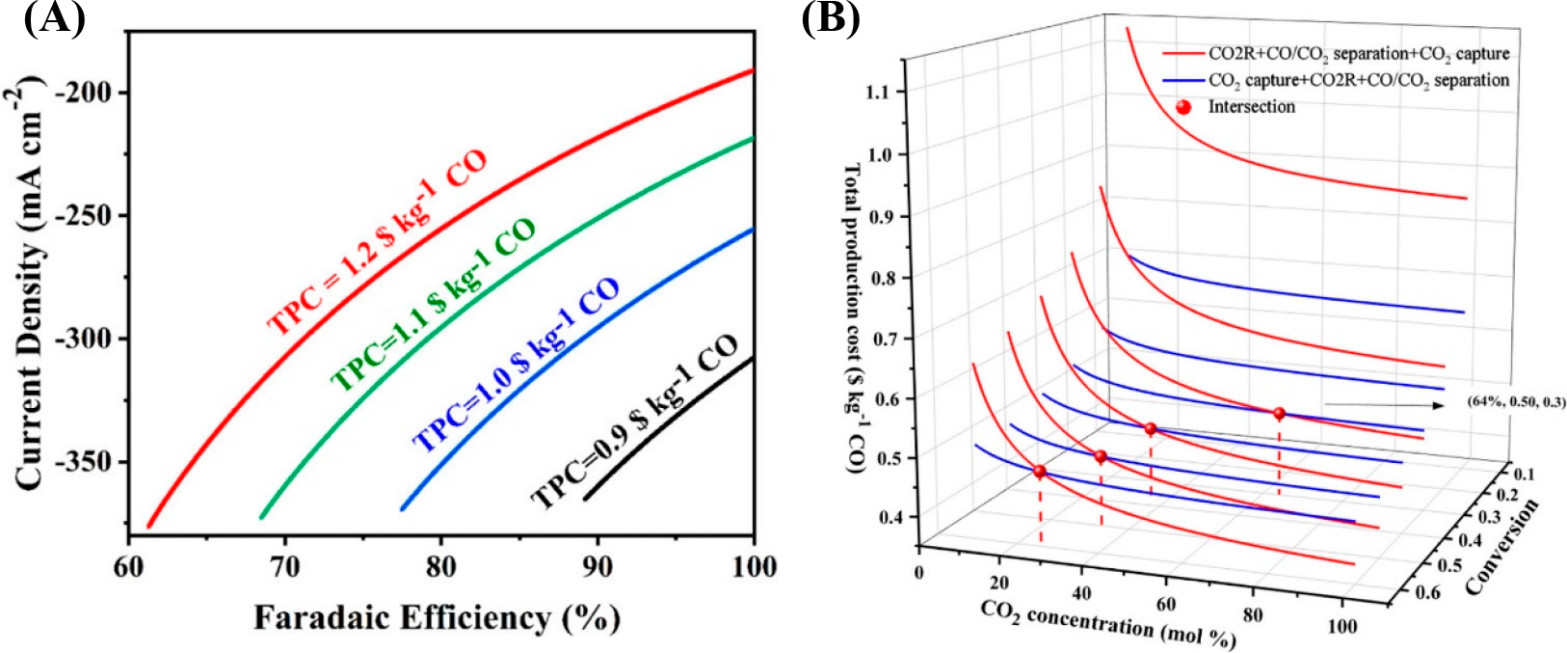
(A) CO total production cost (TPC) plotted as functions of current density and faradaic efficiency for determining profitability by assuming plant lifetime of 20 years and electrolyzer cost of 10,849 $/m^2^. (B) CO TPC plotted as a function of CO_2_ concentration, assuming single-pass conversion, current density of 300 mA/cm^2^, and faradaic efficiency of 99%. Reproduced from ref (220). Copyright 2021 ACS.

Contrary to the production of formic acid and methanol, CO production required an electrolytic reactor in which the IL functioned as an electrolyte, replacing the use of a thermocatalytic reactor. However, Chang et al.^220^ considered the electrolyzer as a black-box model with a given CO_2_ conversion; its main parameters, such as current density or efficiency, were drawn from previous studies, and other secondary parameters were only measured at the laboratory scale. Thus, although the lack of adequate reaction system descriptions was also a concern, the reaction system in that study was different. The separation section comprised gas–liquid separation in a flash vessel and PSA-induced CO_2_/CO separation. Because the study focused on the economic feasibility and not the design of the process, the PSA was also based on data obtained from the literature and assumed an optimistic case in which impurity-free CO_2_ could be recirculated. However, the authors subsequently analyzed the sensitivity of the parameters obtained from the literature, which was novel with respect to other studies in which data were obtained from the literature. Certain guidelines were provided for designing further electrochemical CO_2_ conversion processes, such as the requirement for increasing the faradaic efficiency at low current densities for the product to be cost-effective, as shown in Figure 33A, or the requirement for a minimum CO_2_ concentration in the electrolyzer, as shown in Figure 33B. In addition, the consideration of the capital costs enabled the identification of the electrolyzer as the greatest contributor to those costs. For producing formic acid and methanol, although Bello et al. designed a process similar to the one shown in Scheme 6A, an additional purification step was required because two reaction products were generated.^219^ As in other studies, the authors first experimentally studied this reaction^226^ and then used the fixed conversion value in the reaction. To the best of our knowledge, this was the first and only study in which the process for producing formic acid and methanol simultaneosuly from CO_2_ and using ILs were modeled, opening the door for designing improvements to this process to enhance the profitability of these products.

#### 4.3.3 Integrated CO_2_ Capture and Conversion

Owing to the potential of ILs as physical and chemical CO_2_ absorbents and their application as catalysts, the integration of absorption and conversion is appealing. Unfortunately, computational studies for integrating CO_2_ capture and conversion are scarce. In most CO_2_ conversion studies, pure CO_2_ stream^47,48,219−221^ was used for simplicity because captured CO_2_ stream usually contains impurities, namely N_2_, H_2_, and CH_4_. Two studies have approached a simplified CCU integration. Chang et al.^220^ considered the cost of the capture operation—from previous literature—which was a step forward in this type of study because it is not usually performed although CO_2_ capture is a necessary part in CO_2_ conversion, while Bello et al. captured CO_2_ in a semichemical manner by dissolving pure CO_2_ in the IL to form a CO_2_–IL complex.^219^

**Scheme 7:**
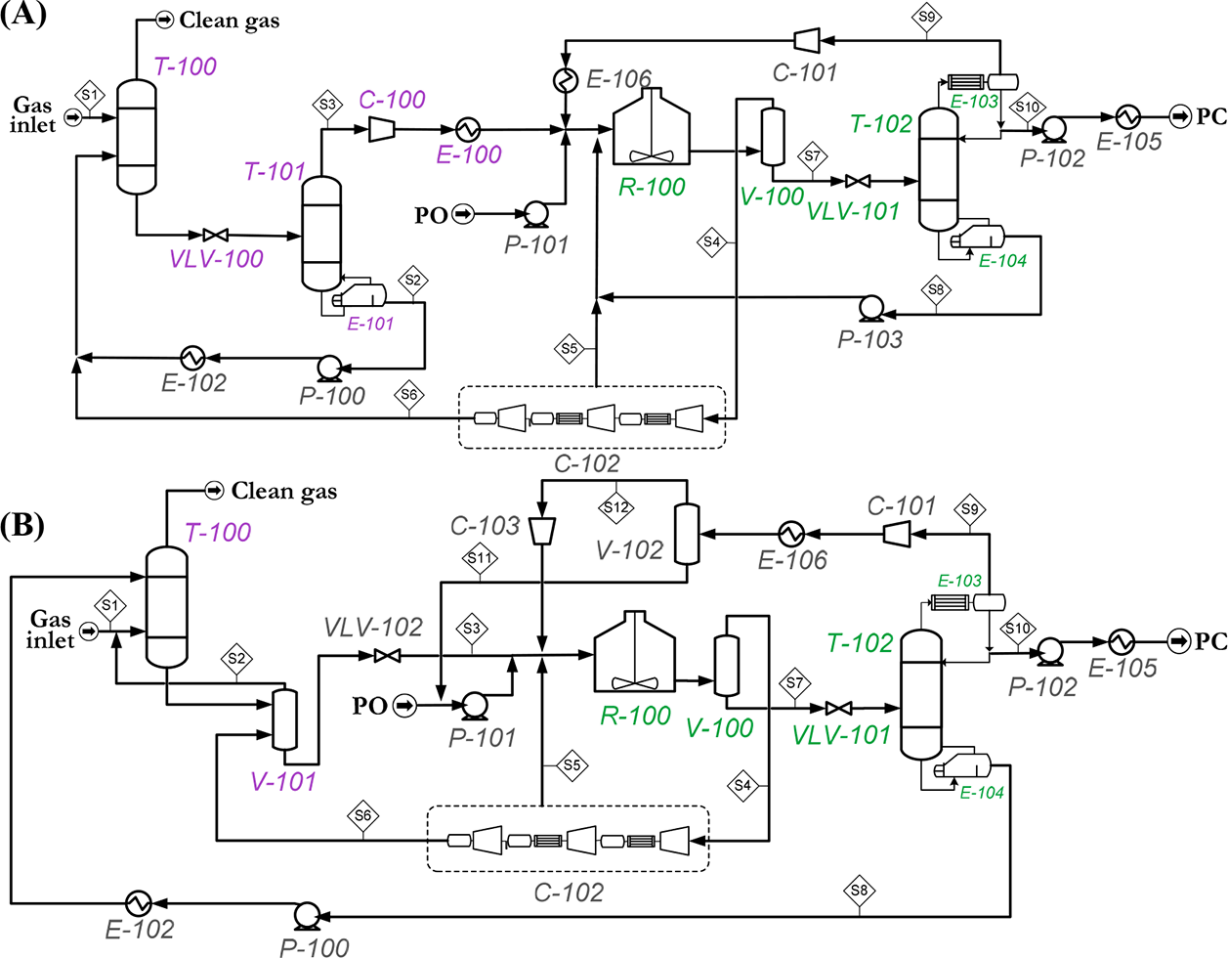
Flowsheet for (A) SCCU and (B) ICCU Processes. Reproduced from Ref (60). Copyright 2022 Elsevier.

**Figure 34:**
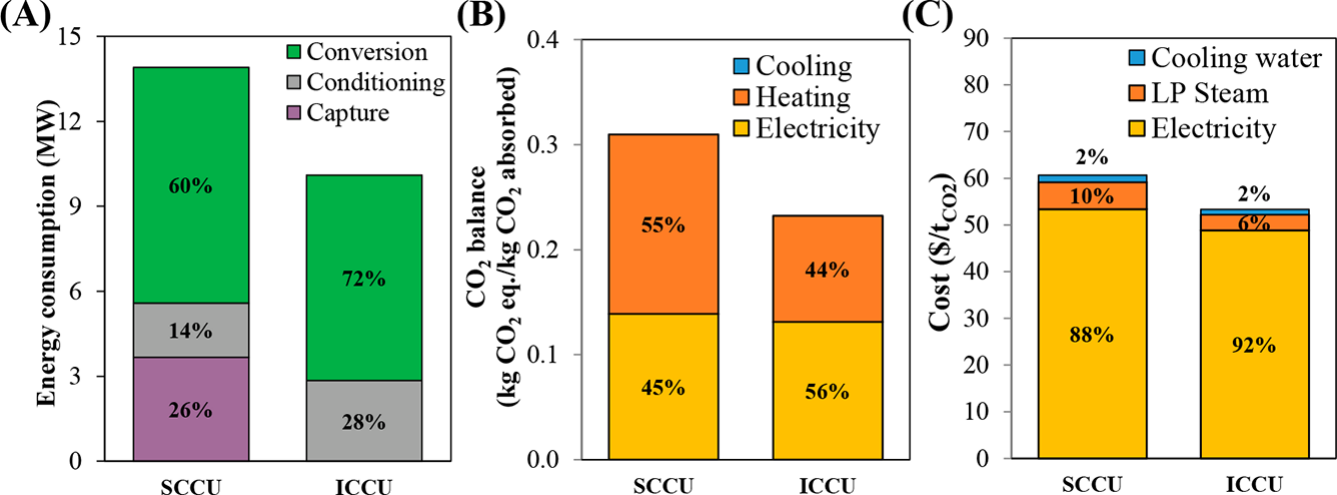
(A) Capture-, conversion-, and conditioning-related energy consumptions. Contributions of utilities to (B) CO_2_ balance and (C) cost for both SCCU and ICCU processes. Reproduced from ref (60). Copyright 2022 Elsevier. Inlet stream comprising 40 and 60 mol % CO_2_ and H_2_, respectively; CO_2_ absorption performed in RADFRAC (rate-based) model at 40 °C and 32.7 bar. CO_2_ converted in RSTOIC model at 120 °C, 15 bar, and fixed epoxide conversion rate of 80%.

Recently, Hernández et al. simulated the first process that integrated IL-based CO_2_ capture and conversion.^60^ In that study, two process designs were implemented. In the separated capture and conversion process (SCCU), CO_2_ was first chemically captured in the IL and then the IL was regenerated and the H_2_-impurity-containing CO_2_ was fed to the conversion section (Scheme 7A). In the other process design, both operations were integrated (ICCU) and the regeneration operation was removed, sending the CO_2_–IL complex and H_2_ impurities directly to the CO_2_ conversion section (Scheme 7B). The goal of the study was to assess the technical feasibility for developing IL-based CCU processes for producing cyclic carbonates. In addition, the potential energetic, economic, and environmental improvement in the ICCU was evaluated based on the utility costs with respect to the SCCU by avoiding costly absorbent regeneration. As shown in Figure 34A, the energy demands could be reduced by 27% by integrating the CO_2_ capture and conversion processes.^60^ Nevertheless, the CO_2_ conversion and product purification accounted for the largest part of the energy consumption in both the SCCU and ICCU processes owing to the high boiling point of the carbonates and the thermal stability of the ILs, two key factors in the future design of these processes. Both factors influenced the distillation-based IL/carbonate separation, which had to operate under vacuum to avoid compromising the IL stability.^60^ Therefore, the study provided insights into the design of ICCU processes toward other alternative products that are less challenging to separate or the search for other separation strategies, such as liquid–liquid extraction, because the benefit for removing the IL regeneration step would be retained after absorption. In addition, as shown in Figure 34B,C, the results revealed that the integration of both processes was beneficial for reducing the utility costs and CO_2_ emissions (−14 and −26%), respectively.^60^

#### 4.3.4 Outlook

In summary, the application of process simulation in the field of IL-catalyst-based CO_2_ conversion has enabled a paradigm shift in the design of these catalysts, as lower temperatures are not necessarily more beneficial at the process scale. Additionally, the design of an efficient IL recovery is as important as the conversion it provides; hence, the anion and cation combination of the IL should also be selected according to the recoverability. Furthermore, process simulation also helps to focus attention on other parts of the process beyond the reaction because the product purification, reactants, and catalyst recovery play important roles in different energy, economic, and environmental indicators. Thus, the key benefit is the ability to predict the process behavior under different conditions, which enables the early selection of IL catalysts, solvents, operating conditions, and equipment specifications. Furthermore, the simulation of the complete process for producing CO_2_-derived compounds enables LCAs to be conducted, which are crucial in these types of processes. If the process emits more CO_2_ than it consumes, pursuing that process is impractical.

Currently, however, studies on CO_2_ conversion are scarce at the process scale and are even more limited for integrating CO_2_ capture and conversion. Additionally, IL-based CO_2_ conversion simulation studies lack robustness for defining reactions, which is concerning owing to the numerous IL-based experimental CO_2_ conversion studies. The absence of reaction kinetics renders reactor sizing infeasible, which limits the estimation of capital costs. In addition, most processes focus on the study of energy demand or costs but not the study of process variables or reaction conditions, including byproducts. Furthermore, the lack of comparison between CO_2_-based production processes and conventional synthesis routes is remarkable, even though they could be compared in economic and/or environmental terms. Finally, few studies support the selection of IL catalysts, and IL compositions and prices are often neglected. All these factors hinder progress in the reproducibility and design of process simulations.

### 4.4 Gas-Separation Processes

**Scheme 8:**
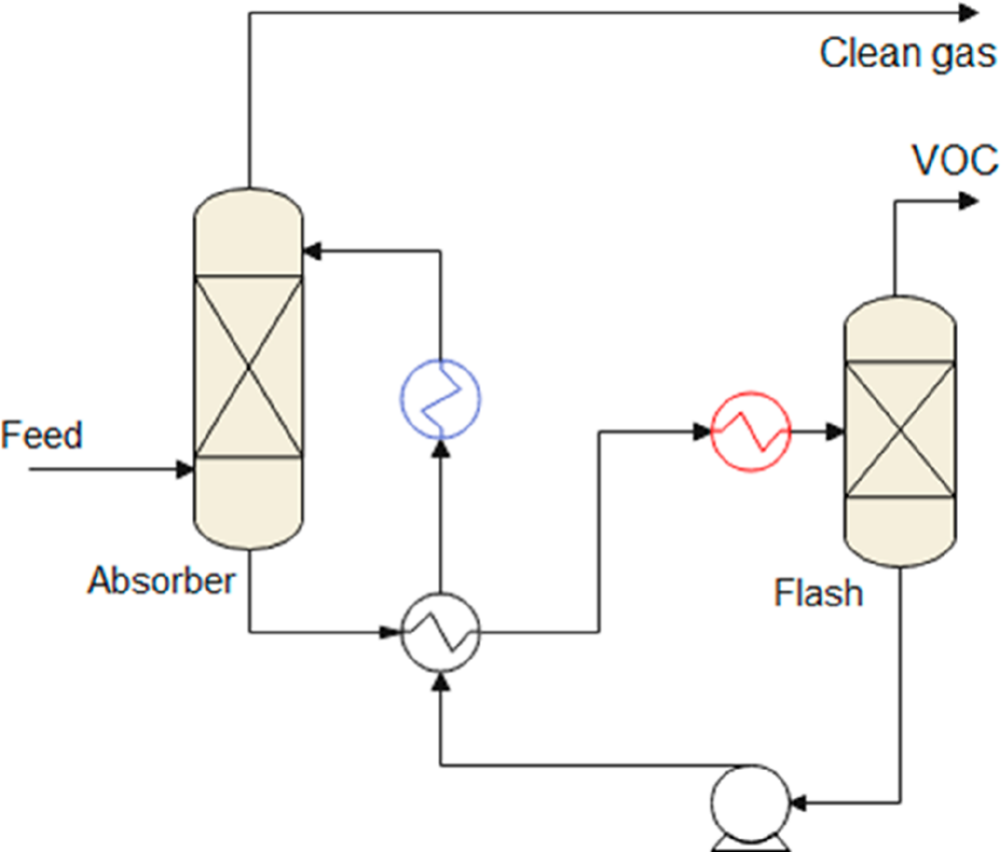
Typical Flowsheet of IL-Based VOC Absorption Process

This section reviews the impacts of process simulations on the scientific community for analyzing IL-based absorption processes to capture remarkably different gas solutes, including volatile organic compounds (VOCs), hydrogen sulfide (H_2_S), ammonia (NH_3_), and water (H_2_O), ranging from nonpolar condensable compounds to polar gases. In all these relevant cases, the different contributions from the literature have been synthesized considering thermodynamic equilibria, mass-transfer kinetics, operation designs, regeneration processes, and economic aspects (Table 5). The typical scheme of gas absorption processes is depicted in Scheme 8. This flowsheet mainly comprises an absorption column (typically modeled with a RADFRAC column in Aspen Plus) in which the IL absorbent countercurrently contacts and treats the gas. From this column, the purified gas can be obtained. Then, after preheating, the exhausted absorbent is fed to a desorption column in which the IL is regenerated and returned to the absorption column after conditioning to the operating pressure and temperature of the absorber. The upper stream of the regeneration stage enabled the removal of the gas. According to the literature, different proposals have been made for the regeneration stage, such as flash operations or stripping columns (usually modeled by FLASH2 or RADFRAC modules, respectively, in Aspen Plus).

**Table 5:**
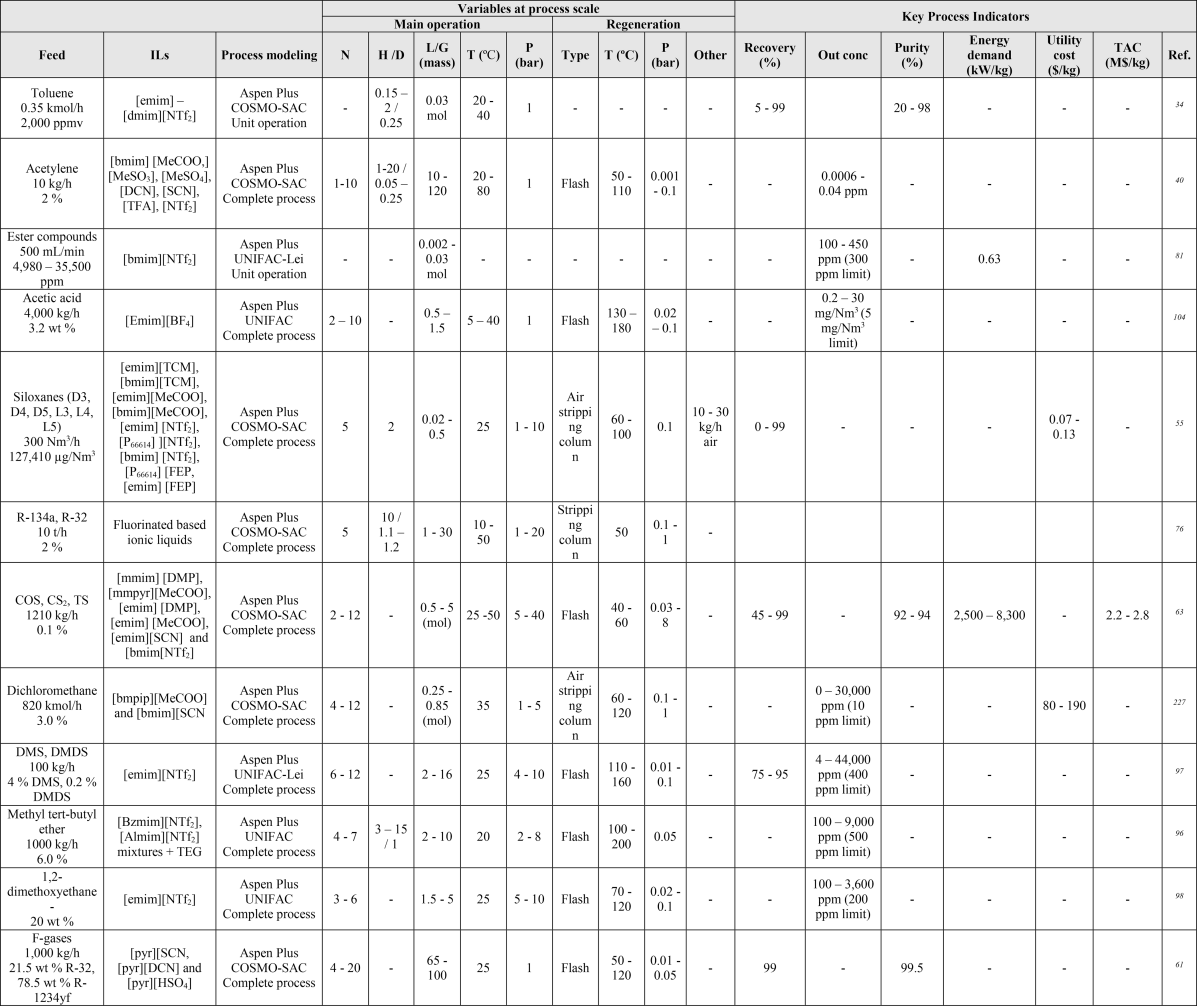
Summary of Variables at Process Scale and KPIs in IL-Based VOC Capture Processes

The contributions of process simulators to the research of IL-based gas absorption processes have included not only the selection of ILs based on the thermodynamics and kinetics of the absorber but also the regeneration stage and complete process by considering absorbent recycling. Moreover, the operating conditions of the process could be optimized. Toward this goal, the absorber pressure, temperature, and number of stages; IL mass flow; and desorption pressure and temperature have been studied. In this manner, gas recoveries and purities, energy consumption, CAPEX, and OPEX have been evaluated as KPIs, enabling gas absorption processes to be conceptually designed based on ILs at the industrial scale and process performances to be compared to those of the current benchmark industrial solvents. The reported process simulation analyses of IL-based gas absorption classified by gas solute type are summarized in Table 5, including the analyzed operating variables, main KPI values, and corresponding references. The solute types were classified as follows: VOCs, including numerous compounds studied because of their separation importance; H_2_S (as one of the main acidic gases that typically must be removed); the dehydration process because water can be easily removed using ILs; and NH_3_ because it is an important raw material and contaminant in different industries.

#### 4.4.1 Absorption of Volatile Organic Compounds

The use of ILs for absorbing VOCs in process simulators has been widely reported in the literature. Notably, numerous VOCs (toluene, acetylene, ethyl acetate, etc.) and various ILs (imidazolium-, acetate-, sulfonate-based, etc.) have been evaluated. Overall, high recoveries were observed under different operating conditions, revealing the excellent potential for tunable IL structures to design low-volatility absorbents that have a high absorption capacity and selectivity. This paragraph compiles the principal literature contributions of these solutes and solvents.

**Figure 35:**
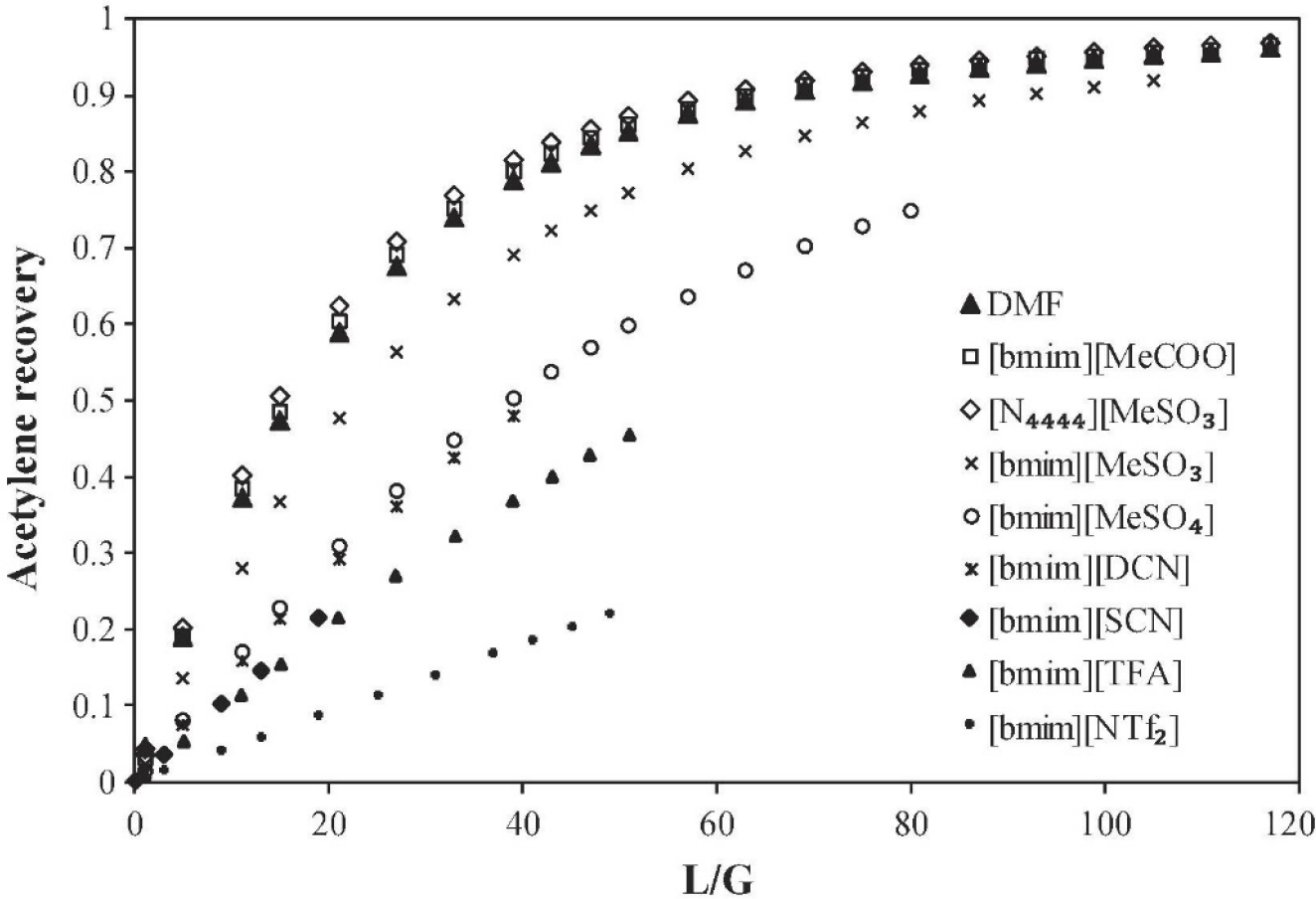
Acetylene recoveries using dimethylformamide, 8 ILs, and RADFRAC column in equilibrium mode plotted as functions of L/G ratio. Reproduced from ref (40). Copyright 2018 Elsevier. Process conditions: absorber pressure: 1 bar, absorber temperature 40 °C, L/G = 10–1,200 (mass), 2 stages, and equilibrium mode.

**Figure 36:**
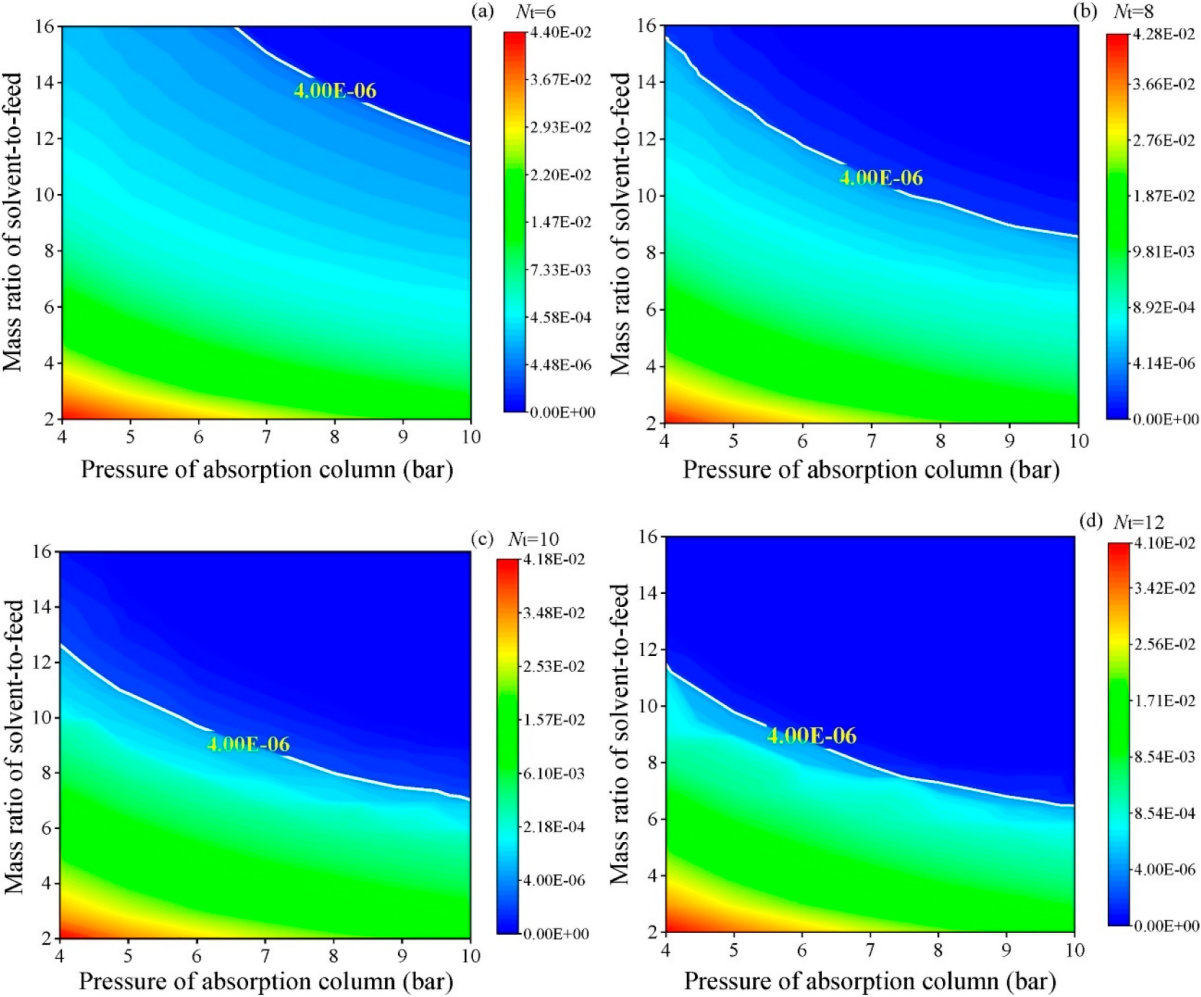
Mass fraction of DMS in purified gas plotted as functions of L/G ratio (2–16), pressure (4–10 bar), and number of absorber stages (6–12) in equilibrium mode. Reproduced from ref (97). Copyright 2021 Elsevier.

From the thermodynamic perspective, the optimal ILs have been widely selected by computing the gas recovery in an absorption column using an equilibrium-based model (usually a RADFRAC column in equilibrium mode in Aspen Plus). For example, Figure 35 shows the acetylene recovery plotted as a function of the L/G ratio for different ILs and an industrial reference solvent (dimethylformamide, DMF).^40^ First, an increase in the L/G ratio clearly implied higher gas recoveries. This behavior was easily extrapolated to IL-based gas absorption operations.^55,61,76,81,96−98,104,227^ Clearly, at least two ILs ([bmim][MeCOO] and [N_4444_][MeSO_3_]) were thermodynamically competitive with the benchmark, achieving nearly complete VOC recovery under fixed operating conditions, while the other evaluated ILs did not exhibit this behavior. Moreover, the study found that 4–6 absorber stages were sufficient for the task, which agreed with the findings of all the studies on ILs-based VOC absorption.^55,61,76,81,96−98,104,227^ The evaluation of the toluene absorption^34^ revealed that [dmim][NTf_2_] was the best candidate and competitive with the industrial solvent (*n*-hexadecane) for recoveries in the equilibrium mode. The eliminations of volatile ester compounds (ethyl acetate, ethyl propionate, and butyl acetate) were evaluated using [bmim][NTf_2_] in the equilibrium mode. The results revealed that an increase in the IL flow reduced the ester concentration in the purified gas stream and may be competitive with traditional solvents. Acetic acid was removed using [emim][BF_4_], which reduced the VOC concentration from 15,300 to 300 ppm under different operating conditions.^104^ Moreover, because an increase in the absorber temperature lowered the recoveries, the operating range was reduced to 5 °C–20 °C. In biogas upgrading, cyclic and linear siloxanes containing different numbers of silicon atoms were also efficiently captured using ILs.^55^ Nine different ILs were found at different operating pressures (from 1 to 10 bar), revealing that at 10 bar, FEP-based anions were the most suitable for almost completely removing siloxanes in the IL-based absorption process. Furthermore, the absorption of hydrofluorocarbons (R-134a and R-32) with fluorinated ILs was evaluated at the process scale.^76^ Twelve fluorinated ILs were found at partial pressures of 0.02 and 1 bar and different L/G ratios, which revealed that the [omim][NTf_2_] and [emim][CF_3_SO_3_] ILs had the highest R-134a and R-32 recovery rates, respectively. Moreover, hydrofluorocarbons (R-32/R-1234yf) were selectively removed using very different ILs, including DCN, SCN, and HSO_4_-based anions.^61^ The [DCN]-based IL required the lowest L/G ratio (66 mass basis) and fewest stages (16) to purify 99.5% of the R-1234yf and recover 99% of the R-32. Coke (COS, CS_2_, and TS) was desulfurized using six different ILs at different L/G ratios, absorber pressures, and numbers of stages.^63^ The [mmpyr][MeCOO] IL removed 99.05% of the total sulfur content in eight stages at 14 bar of total pressure. The use of the [bmpip][MeCOO] and [bmim][SCN] ILs to absorb dichloromethane from air streams was also evaluated at different numbers of absorber stages, pressures, and L/G ratios.^227^ The [bmpip][MeCOO] IL was the best candidate because it almost totally eliminated DCM (9 ppm in the purified gas stream) and had the minimum L/G (0.25) while operating in seven stages at 3 bar of total pressure. The use of the [emim][NTf_2_] IL to eliminate odorous sulfur-based VOCs was also evaluated at different operating pressures, L/G ratios, and number of stages.^97^ As shown in Figure 36, the increase in the number of stages led to higher recoveries. Similarly, higher absorber pressures and L/G ratios implied higher recoveries. The optimal number of stages was 10, and L/G ratios ranging from 8 to 12 at 10 and 4 bar, respectively, were sufficient to leave only 4 ppm of sulfur-based VOCs in the purified gas stream. The use of NTf_2_-based ILs to remove methyl *tert*-butyl ether was evaluated at different L/G ratios (2–10), operating pressures (2–8 bar), and numbers of stages (4–7).^96^ The [Bzmim][NTf_2_] IL was the best candidate because it reduced the VOC contents to below 500 ppm in the clean gas stream under different pressure conditions and for different numbers of stages. The removal of 1,2-dimethoxyethane with [emim][NTf_2_] required six absorber stages at 8.5–10 bar and an L/G ratio ranging from 1.5 to 5 to leave only 200 ppm of 1,2-dimethoxyethane in the clean gas stream.^98^ According to process simulation studies, operating temperatures between 10 and 40 °C have been the most common, while an increase in the operating pressure (between 10 and 20 bar) led to higher gas recoveries. An increase in the IL mass flow resulted in higher recoveries but preserved a reasonable L/G ratio.

**Figure 37:**
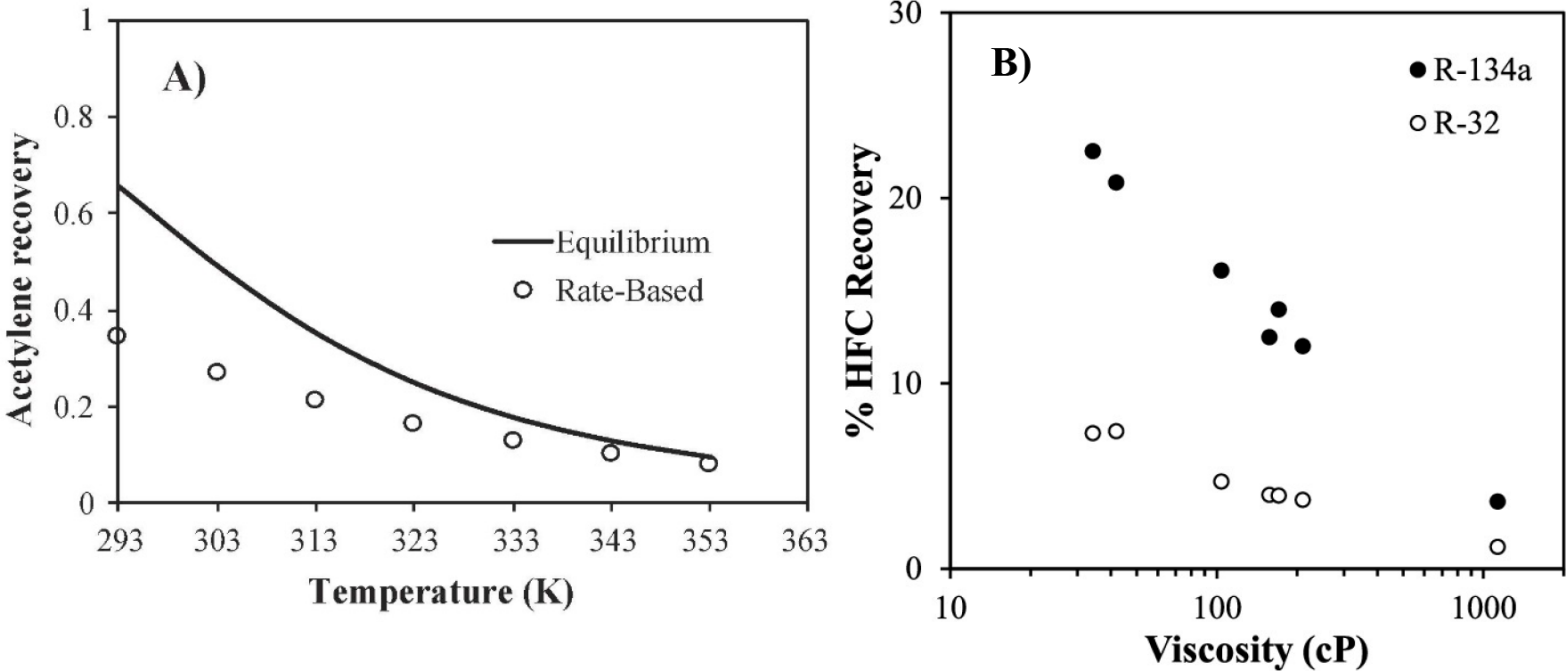
(A) Acetylene recoveries plotted as functions of temperature in equilibrium- and rate-based modes for [bmim][MeCOO] at 1 bar, 25 °C–120 °C, L/G = 10 (mass) for five stages and 1 m separation columns (rate-based). Reproduced from ref (40). Copyright 2018 Elsevier. (B) Hydrofluorocarbon recoveries in rate-based mode plotted as a function of viscosity (25 °C) for fluorinated ILs at L/G of 6, partial pressure of 1 bar, and 1 m high separation columns. Reproduced from ref (76). Copyright 2020 ACS.

Owing to the high viscosity of ILs, process simulations have revealed the importance for considering mass-transfer kinetics in absorption operations. Notably, all the simulations have been conducted using commercial packing for the absorption column design. Some studies have compared the behavior of ILs in VOC absorption by considering only the thermodynamics (equilibrium mode) and kinetics (rate-based mode) in the absorption column design. This enabled not only the evaluation of whether the IL thermodynamic or transport properties controlled the VOC absorption process but also the design of the absorption and desorption columns (required height and diameter). The first study that reported this concept for VOC absorption using commercial packed columns focused on toluene absorption and compared two reference solvents [*n*-hexadecane and di(2-ethyl) hexyl adipate (DEHA)].^34^ Initially, differences in recoveries were observed between the rate-based and equilibrium modes for ILs and traditional solvents, with deviations increasing for more viscous IL absorbents. However, the solvent thermodynamic performance increased with increasing absorption capacity. Compared with traditional solvents, the NTf_2_-based ILs were not competitive with DEHA in toluene absorption. Therefore, the thermodynamics prevailed over the kinetics. However, in acetylene absorption,^40^ the control of the mass-transfer kinetics was clear, especially at low temperatures and with high-capacity, high-viscosity ILs (such as [bmim][MeCOO]) (Figure 37A). Compared with the traditional reference solvent (dimethylformamide), the selected ILs required higher columns than DMF at all the operating temperatures and pressures in IL-based acetylene absorption because the absorbent viscosity was the main selection criterion and the acetylene absorption capacity of the selected ILs was competitive with that of the DMF. For siloxane absorption in biogas-upgrading technologies,^55^ both the thermodynamics and kinetics were crucial for selecting the best IL candidate, and the [emim][FEP] IL stood out for both its high mass solubility and moderately low viscosity. Additionally, IL-based hydrofluorocarbon absorption clearly revealed the control of the mass-transfer kinetics.^76^ Compared with the equilibrium-based calculations, the rate-based calculations revealed very low recoveries. Moreover, the IL absorption behavior could be ordered based on their viscosities (see Figure 37B), with the least viscous IL exhibiting the optimal absorption. These results revealed that the selection was based not only on high-solubility but also mainly low-viscosity ILs was essential. Considering mass-transfer kinetics, MTBE absorption with ILs and TEG (a benchmark conventional absorbent) revealed that using the pure ILs, higher columns were required for achieving the same separations as those accomplished using the traditional solvent.^96^ On the contrary, mixtures of ILs and TEG may be a good solution because shorter columns were required using the same absorbent mass flow. Wang et al.^103^ simultaneously removed SO_2_ and CO_2_ by modeling rigorous rate-based absorption columns for different ILs and found that low-viscosity ILs optimized the separation and that the [EtOHmim][NTf_2_] and [bpy][DCN] ILs were the best candidates. Reportedly, less energy was consumed during a stepwise separation in which SO_2_ was removed first and then a second absorption column was used to eliminate CO_2_. Process simulations revealed that the polar solute absorption operations exhibited remarkable kinetic control. However, for nonpolar compounds, such as toluene, the absorption process was controlled by the thermodynamic equilibria.

**Figure 38:**
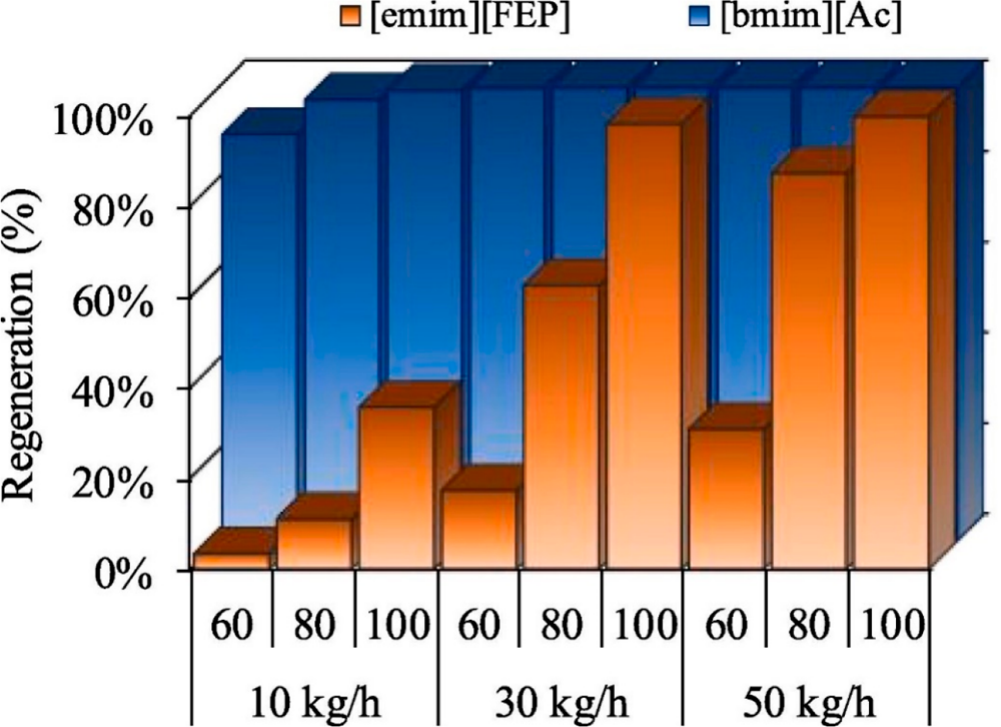
IL regeneration using air-stripping column at 0.1 bar and different temperatures (60 °C–100 °C), and air-stripping flows (10–50 kg/h) in siloxane capture process for two different ILs. Reproduced from ref (55). Copyright 2020 Elsevier.

Traditionally, IL regeneration processes have been implicitly simulated using flash distillation in commercial simulators. Most VOC-absorption-related studies have used flash distillation by changing the temperature or/and pressure to regenerate the IL.^40,61,63,96−98,103,104,227^ Different separation approaches have been discovered, including from only one flash distillation to a train of two or three flash tanks. Temperatures have been varied from 100 to 160 °C; vacuum pressures, from 0.01 to 0.10 bar. As expected, IL regeneration ratios and solute recoveries were both higher at the highest temperatures and lowest pressures. The simulations for the acetylene absorption processes revealed that compared with conventional organic solvents, nearly nonvolatile ILs resulted in no loss of IL absorbent in the gas/vapor stream of the regeneration flash in contrast to DMF, which required costly multistage distillation to produce high-purity acetylene. However, because the thermal stability of the ILs must be ensured before they degrade, other regeneration strategies, such as gas stripping^55,227^ or stripping distillation,^76^ have been developed to attempt to avoid high temperatures that would compromise the IL thermal stability. Figure 38 shows the IL regeneration percentage plotted as functions of the temperature and air-stripping flow used in the siloxane capture.^55^ Clearly, at temperatures that did not compromise the IL thermal stability (below 100 °C), the total IL regeneration showed the maximum recovery at the highest temperatures and air-stripping flows. In another study, flash distillation was used, and the purified gas stream functioned as a stripping agent in a second regeneration column.^227^ The optimal ratios of stripping gas were somewhere between 0.2 and 0.5. The stripping distillation column approach^76^ comprised a distillation column with only a kettle at the bottom of the column to regenerate the IL. The hydrofluorocarbons were completely recovered under milder vacuum pressure conditions (from 0.1 to 1 bar).

Process simulations have enabled the evaluation of the IL performance in complete processes, including absorption and regeneration stages. Thus, the IL performance could be compared with those of traditional solvents, and the technical and economic feasibility could be analyzed for different variables (chemical and energy consumptions, equipment sizes, operating and immobilized costs, etc.).

Few reported process simulation studies have performed energy consumption and cost estimations of IL-based VOC absorption processes. The acetic acid absorption process with [emim][BF_4_] showed a necessary heating duty of 0.63 kW/kg_acetic acid_ (considering the use of flash distillation and a heat exchanger) and a cooling duty of 0.12 kW/kg_acetic acid_ (two coolers) to achieve a VOC content of 300 ppm in the purified gas stream.^104^ Additionally, the computation of vacuum-associated electrical costs was considered as essential in flash distillation. Hydrofluorocarbon absorption with [emim][TFO] resulted in OPEX costs of 70 and 130 $/t_HFC_ and CAPEX costs of 1.5 and 1.2 M$ (for R-32 and R-134a, respectively), which approximated those of IL-based physical CO_2_ absorption.^76^ Notably, because the vacuum pump investment accounted for 55% of the total cost, the process cost was the key in the HFC absorption processes. The selective separation of hydrofluorocarbons with HSO_4_-based ILs showed an energy demand of 1.1 kW/kg and a pump energy of 2.2 W/kg.^61^ Notably, although the DCN-based IL was the best candidate from the thermodynamic perspective, the HSO_4_-based IL presented the lowest energy consumption among the solvents. Therefore, technoeconomic criteria should also be considered valuable for selecting ILs. IL-based sulfur–VOC processes have accounted for CAPEX costs of 12 M$ and OPEX costs of 2–4 M$/year, with [empyr][MeCOO] exhibiting the best behavior.^63^ Dichloromethane removal revealed a lower OPEX with [empip][MeCOO] than with DMSO (from 190 to 80 $/kg), which was used as a reference solvent, mainly owing to the lack of HP steam in the regeneration process because the distillation column was avoided.^227^ The technoeconomic results of the 1,2-dimethoxyethane absorption process using [emim][NTf_2_] were compared with those of the 1,2-dimethoxyethane absorption process using triethylene glycol.^98^ The reduction in OPEX costs for the IL-process was clear (from 61.1 to 46.4 k$/year), mainly owing to the reduction in the heating duty (no distillation column was required to regenerate the IL). However, the CAPEX costs were higher for using IL (42 k$/year) with respect to TEG (5.4 k$/year), principally owing to higher absorption columns because more IL was required to achieve the goal. For the TAC, IL-based process reached 60.4 k$/year, while the TAC of the TEG process reached 72.9 k$/year. Clearly, OPEX controlled the final TAC because the IL process was the most economical candidate. Therefore, the evaluation and full consideration of all the costs was important for making informed decisions.

In summary, the selection of ILs for process-simulation-based VOC absorption strongly depended on the nature of the gas compound that were being treated. For example, the selection of ILs for the VOC absorption of nonpolar compounds (such as toluene) was principally based on thermodynamics because the best candidates were nonpolar low-viscosity ILs (e.g., [dmim][NTf_2_]). Their low viscosity led to the thermodynamic control of the process. On the contrary, for polar VOC absorption, ILs were selected based on both thermodynamics (high solubility) and kinetics (low viscosity). This was demonstrated in acetylene ([N_4444_][MeSO_3_]) and F-gas absorption ([emim][FEP]). When the viscosity was the key, the operating pressure had to be increased in the absorption tower to obtain competitive recoveries. Therefore, valuable IL selection criteria should include the mass-transfer kinetics (rate-based mode) in the simulation model of the absorption column. Regarding the regeneration process, compared with traditional organic absorbents, ILs were very advantageous owing to their remarkably low volatility, which enabled the use of different separation approaches, such as flash units and stripping and distillation stripping columns. The distillation stripping column enabled the regeneration of ILs at mild temperatures (the thermal stability of the ILs had be maintained, typically at approximately 100 °C) and pressures (by avoiding strong vacuums and, thus, implying higher electricity costs). Regarding the complete process, compared with the traditional reference solvents for each VOC, ILs showed competitive OPEX costs despite CAPEX being higher in IL-based processes owing to the wider absorption columns (more IL mass flow was required to reach the specifications).

#### 4.4.2 H_2_S Absorption

**Figure 39:**
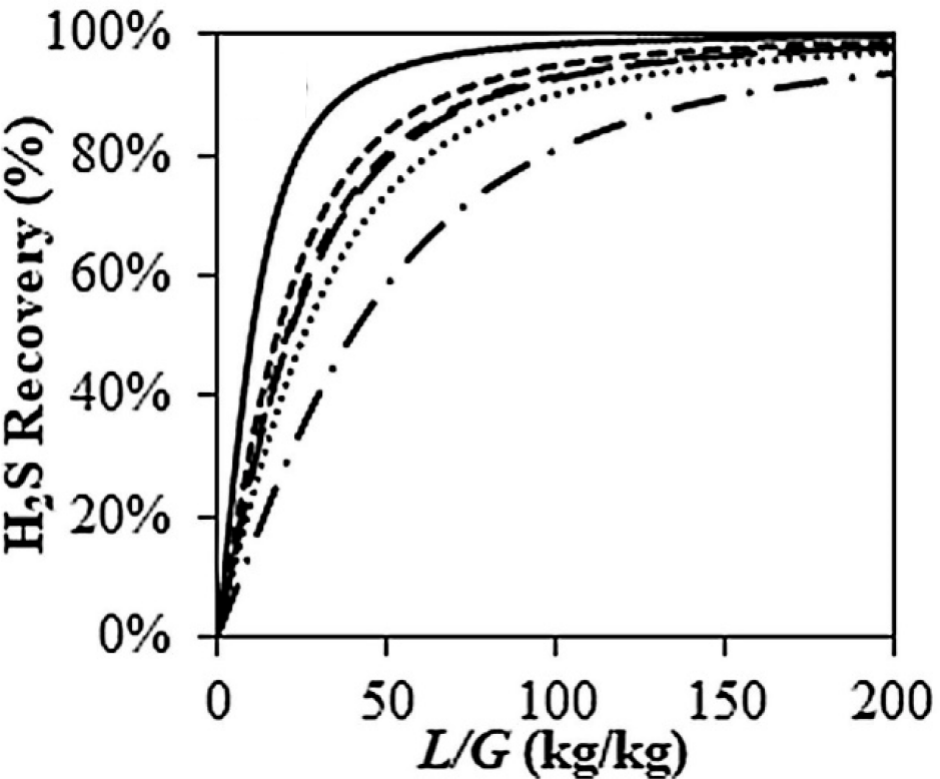
H_2_S recovery plotted as functions of L/G at 3 bar, 25 °C, and two stages for different ILs. Reproduced from ref (56). Copyright 2020 Elsevier.

Different studies have reported the use of computational tools to select the most promising ILs candidates for H_2_S absorption. The principal operating conditions and KPIs of the different processes are listed in Table 6. Figure 39 shows the H_2_S recovery under different L/G conditions at an absorber pressure of 1 bar. First, an increase in L/G could enable the almost total recovery of H_2_S under the studied operating conditions.^56^ Moreover, [emim][MePO_3_] stood out as the best candidate for the task considering only the thermodynamic aspects. Some additional experimentally studied IL absorbents have been computationally evaluated (such as [bmim][PF_6_],^141^ [bmim][NTf_2_],^228^ and [bmim][MeSO_4_]^193^) resulting in higher L/G ratios and operating pressures to reach H_2_S recoveries >95%. Although all these studies analyzed IL-based physical absorption, two process simulation studies^63,75^ reported the first use of [bmim][MeCOO] as a H_2_S chemical absorbent in process simulations, finding substantially higher H_2_S recoveries and, thus, lower IL consumption compared to those of [emim][MePO_3_] (the best physical absorption candidate).

**Table 6:**
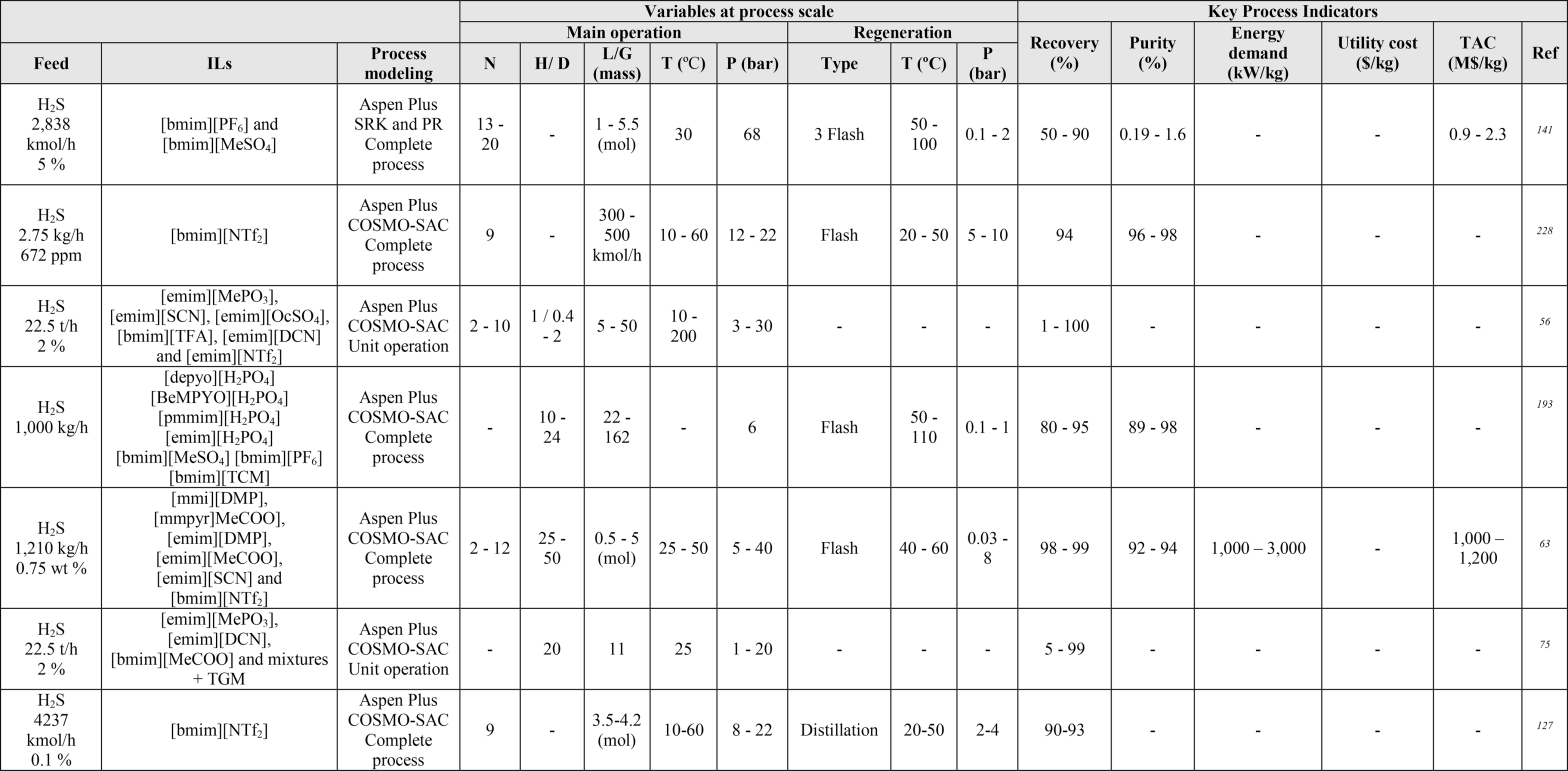
Summary of Variables at Process Scale and KPIs in IL-Based H_2_S Capture Processes

**Figure 40:**
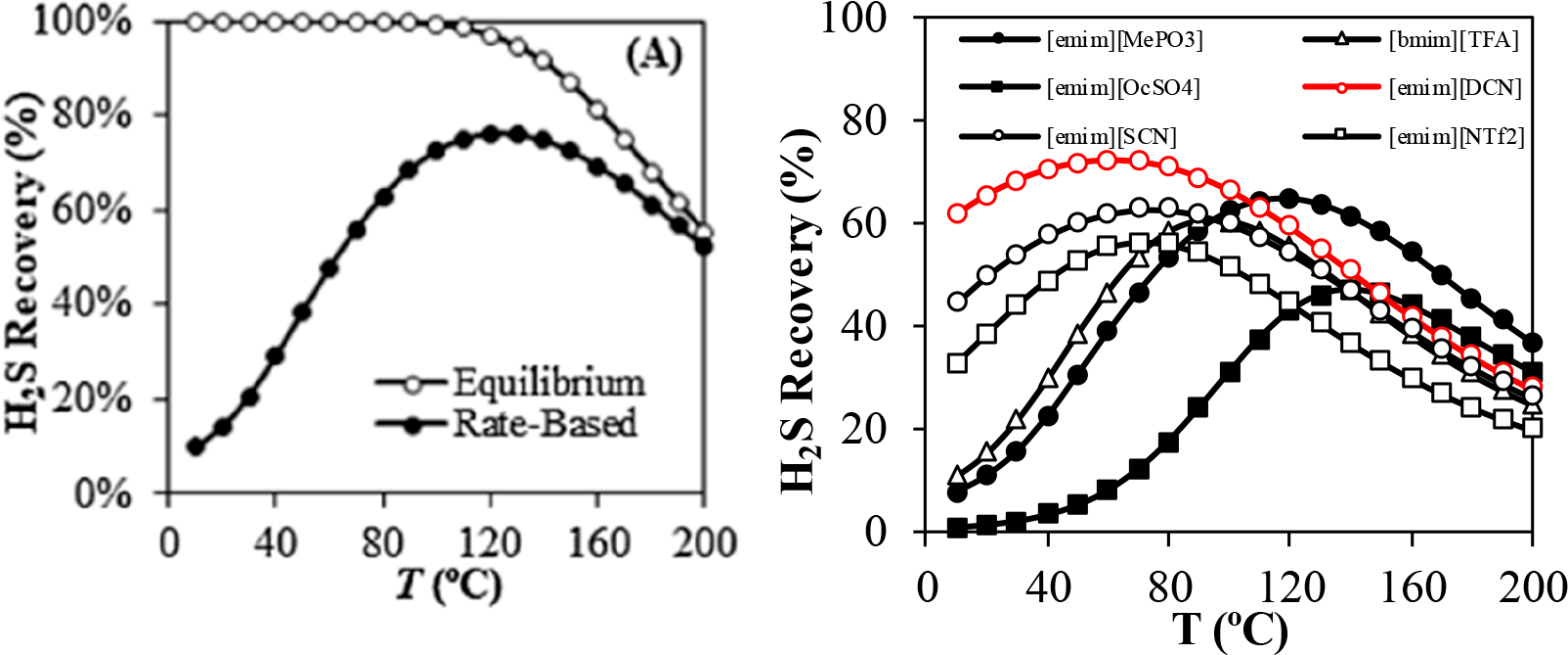
(A) H_2_S recoveries plotted as functions of temperature in both equilibrium and rate-based modes using [emim][MePO_3_] at 30 bar, 0 °C–200 °C, L/G = 30 (mass), 5 stages, and 1 m high columns, and (B) H_2_S recoveries plotted as functions of temperature in rate-based mode for different ILs at 30 bar, 0 °C–200 °C, L/G = 30 (mass), 5 stages, and 1 m high columns. Reproduced from ref (56). Copyright 2020 Elsevier.

**Figure 41:**
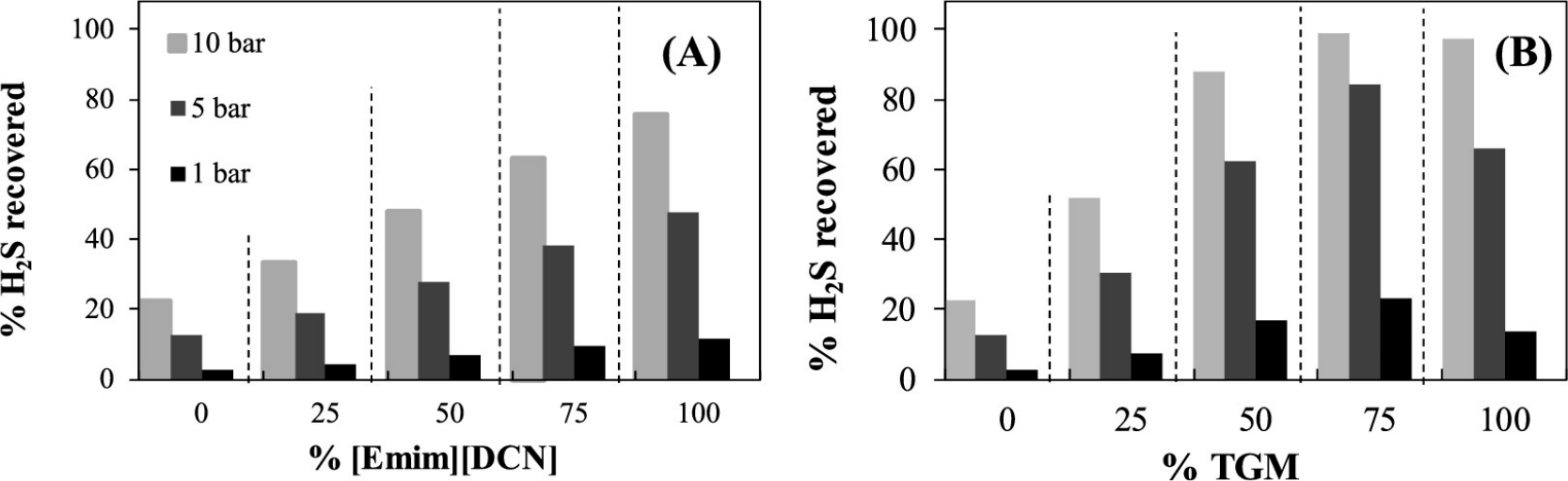
(A) H_2_S recovery using [bmim][MeCOO]–[emim][DCN], and (B) [bmim][MeCOO]–tetraglyme mixtures at 250 ton/h (L), 25 °C, 1–10 bar, 10 stages, and 20 m high columns. Reproduced from ref (75). Copyright 2021 ACS.

Three studies related to IL-based H_2_S absorption included rate-based calculations in their modeling of commercial packed absorption columns.^56,75,193^ These calculations could provide a realistic idea about the importance for selecting ILs based on thermodynamics, kinetics, or both criteria. Figure 40A shows the H_2_S recovery plotted as a function of the absorber temperature in both the equilibrium (only thermodynamics were included in the calculations) and rate-based (mass-transfer kinetic equations were also included) modes for [emim][MePO_3_] (the best candidate from the thermodynamic perspective).^56^ Interestingly, in the rate-based mode, the recoveries rose with increasing temperature to 120 °C and then slightly decreased until they merged with the equilibrium curve. This clearly indicated that the mass-transfer kinetics controlled the process up to 120 °C, above which the thermodynamic equilibrium started to control the process. Remarkably, the study of the performance of different ILs revealed that [emim][DCN] was the best candidate, which produced the highest H_2_S recoveries at lower absorber temperatures (Figure 40B) independent of its moderate absorption capacity, owing to the clear kinetic control of the absorption process in the packing column. Reportedly, [emim][TCM] was also a very good candidate because of its low viscosity and shorter columns than the other candidates.^193^ Finally, H_2_S chemical absorption with [bmim][MeCOO] was evaluated in the rate-based mode.^75^Figure 41 shows the H_2_S recoveries of two different mixtures: [bmim][MeCOO]–[emim][DCN] and [bmim][MeCOO]–tetraglyme. First, independent of its ability for chemically reacting with H_2_S, neat [bmim][MeCOO] clearly showed the lowest recoveries. Then, with increasing cosolvent (low viscosity) content, the recoveries increased until the mixture contained 75% [emim][DCN] or the highest TGM content. This means that, once again the mass-transfer kinetics controlled the operation until the point at which the viscosity was sufficiently reduced (75% mixture) to change the controlling stage to the thermodynamic equilibrium. In H_2_S absorption, the kinetic aspects must be considered for modeling absorption processes, including the design of absorbent blends that have enhanced transport properties and the use of low-viscosity cosolvents mixed with polar ILs that have a high absorption capacity for H_2_S and, consequently, have a relatively high viscosity.

Simulations of the complete H_2_S absorption process have scarcely been conducted simultaneously with CO_2_ removal. Therefore, aqueous amine solutions have been used as traditional solvents. The energy consumption and costs of IL-based processes have been compared with those of aqueous amine solutions.^141^ However, to date, H_2_S has been completely removed by modeling absorption columns in the equilibrium mode. The IL was regenerated using two or three flash distillations (via progressive pressure-swing desorption)^63,141,193,228^ for completely regenerating the IL at 0.1 bar and 80 °C. With respect to amines, the IL-based processes enabled TAC savings of 52% ([bmim][PF_6_]) and 60% ([bmim][MeSO_4_]). These savings were mainly attributed to the regeneration capital and operating costs (distillation for amines and flash for ILs). Acidic gas removal processes have also been compared in technical terms between [bmim][NTf_2_] and different solvents (Selexol, Rectisol, monoethylamine, or K_2_CO_3_) by proposing the simultaneous removal of CO_2_ and H_2_S.^127,228^ For H_2_S removal, the ILs were competitive with traditional solvents and improved the CH_4_ purity because of the high degree of H_2_S/CH_4_ selectivity that they presented compared with traditional solvents. Sulfur-containing compounds, which contained H_2_S, were eliminated from coke-oven gas streams with different ILs.^63^ In all the evaluated ILs, the H_2_S removal was almost complete. From the energy and economics perspectives, [empyr][MeCOO] and [mmpyr][MeCOO] stood out as the best candidates, respectively, because they could chemically react with H_2_S. In these steady-stage process simulations, both CAPEX and OPEX costs were reduced with respect to those of the other physical absorbents.

In summary, for H_2_S absorption processes, the selection of the most suitable ILs was strongly controlled by the velocity of the mass-transfer kinetics. Thus, the selection changed from high-solubility to low-viscosity ILs (physical absorption), such as [emim][FEP]. Notably, the recoveries could also be increased using ILs presenting chemical absorption, such as [bmim][MeCOO]. Owing to their high viscosity, mixtures of ILs and low-viscosity solvents (such as tetraglyme) revealed the best results. Compared with traditional-solvent-based processes, such as Selexol, IL-based processes had lower CAPEX and OPEX costs, as estimated based on process simulations and were, therefore, good alternatives. However, for H_2_S absorption, an IL-based technoeconomic analysis must be performed, including the use of a rate-based column model, to determine both the selection of IL-based absorbents and the overall process performance.

#### 4.4.3 Gas Dehydration

Different IL-based gas dehydration processes have been evaluated using process simulations and compared with the benchmark solvent (triethylene glycol, TEG). The operating variables and KPIs of the different studies reported in the literature are summarized in Table 7.

**Table 7:**
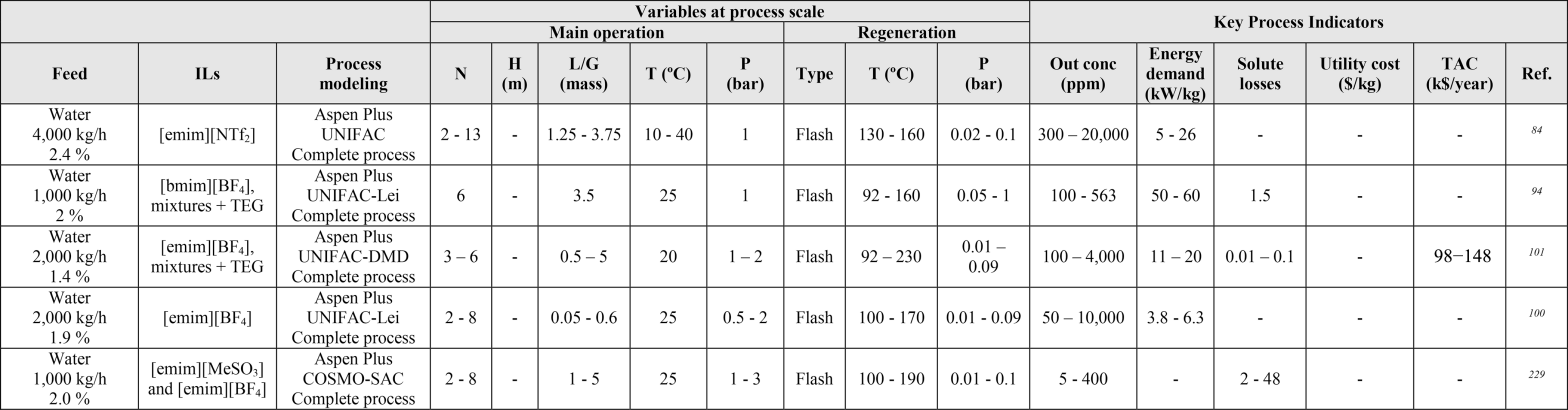
Summary of Variables at Process Scale and KPIs in IL-Based Gas Dehydration Processes

In all these studies, the process was modeled using absorption/regeneration columns in the equilibrium mode. CO_2_ was dehydrated using [emim][NTf_2_] under different operating conditions in the absorber and desorber.^84^ A water concentration limit of 500 ppm was established for the purified gas stream. From four to 12 absorber stages satisfied the limit, and a total of six stages was the best option. Again, L/G ratios of at least 2.5 were sufficient to satisfy the limit of 500 ppm, and the absorber temperatures had to be from 10 to 20 °C to satisfy the limit. The regeneration stage was accomplished using flash distillation and was necessary for operating at a minimum of 140 °C and 0.06 bar. The thermal stability had to be ensured under these conditions. Compared with TEG, IL had a lower L/G to satisfy the 500 ppm condition. The energy consumption comparison revealed a savings of 80% in heating and cooling duties, which was mainly attributed to the distillation column when TEG was used. However, the vacuum-related energy costs (0.049 kW/kg) had to be considered when ILs were used. Additionally, [bmim][BF_4_] was evaluated during CO_2_ dehydration, and it reduced the water content to 100 ppm compared to TEG (560 ppm) under the same operating conditions and at the same L/G ratio.^94^ Moreover, because the energy demands were reduced by 20% with respect to TEG, [bmim][BF_4_] was as good candidate. [bmim][BF_4_] + TEG mixtures were also analyzed because they showed a good balance between good removal ratios and moderate energy consumption. Because the BF_4_-based ILs were good candidates for gas dehydration, [emim][BF_4_] was applied to natural gas dehydration.^101^ The IL showed the best results in terms of the absorbent consumption with respect to the TEG and TEG + IL mixtures. Regarding the costs, the TAC was lower for IL + TEG (98 k$/year) than for pure IL (117 k$/year) and TEG (148 k$/year). Again, dehydration processes were evaluated using other gases, such as methyl chloride, and [emim][BF_4_].^100^ The process simulation results were validated by the experimental data, with very good correlations that reaffirmed the lower water concentrations in the purified gas stream with increasing IL mass flow. Again, [emim][BF_4_] was a good candidate and consumed less absorbent to reach the same recoveries. For the first time, this study considered the potential role of the mass-transfer kinetics in gas dehydration processes and found that although the low viscosity of the selected IL (36 mPa·s) should not imply mass-transfer limitations, rate-based calculations must still be done in future studies. Moreover, the study results reaffirmed that the thermal stability of the IL must be preserved. [bmim][MeSO_3_] was also evaluated for chlorine dehydration,^229^ and its performance was compared with that of [emim][BF_4_], as the reference IL in previous studies. The [emim][MeSO_3_] IL reduced the energy costs by 65%. Additionally, the electrical costs for the required vacuum were reduced from 0.13 to 0.04 kW mainly owing to the low IL mass flow required for separation using the [emim][MeSO_3_] IL.

In summary, gas dehydration processes evaluated using ILs revealed that the use of BF_4_-based ILs exhibited the best results, even compared with those of the traditional reference solvent (TEG). The OPEX costs were drastically reduced using ILs, which substantially facilitated the regeneration stage but was remarkably conditioned by the vacuum requirements. It should be noted that Proionic and Evonik companies have developed IL-based desiccant air conditioning at pilot plant scale, showing lowered energy input.^121^ Once again, simulations must still be performed considering mass-transfer equations for deeply analyzing the potential for using ILs in gas dehydration processes. Additionally, the mixture rules must be reviewed for properly modeling the viscosity when ILs contain a certain amount of water.

#### 4.4.4 NH_3_ Absorption

**Figure 42:**
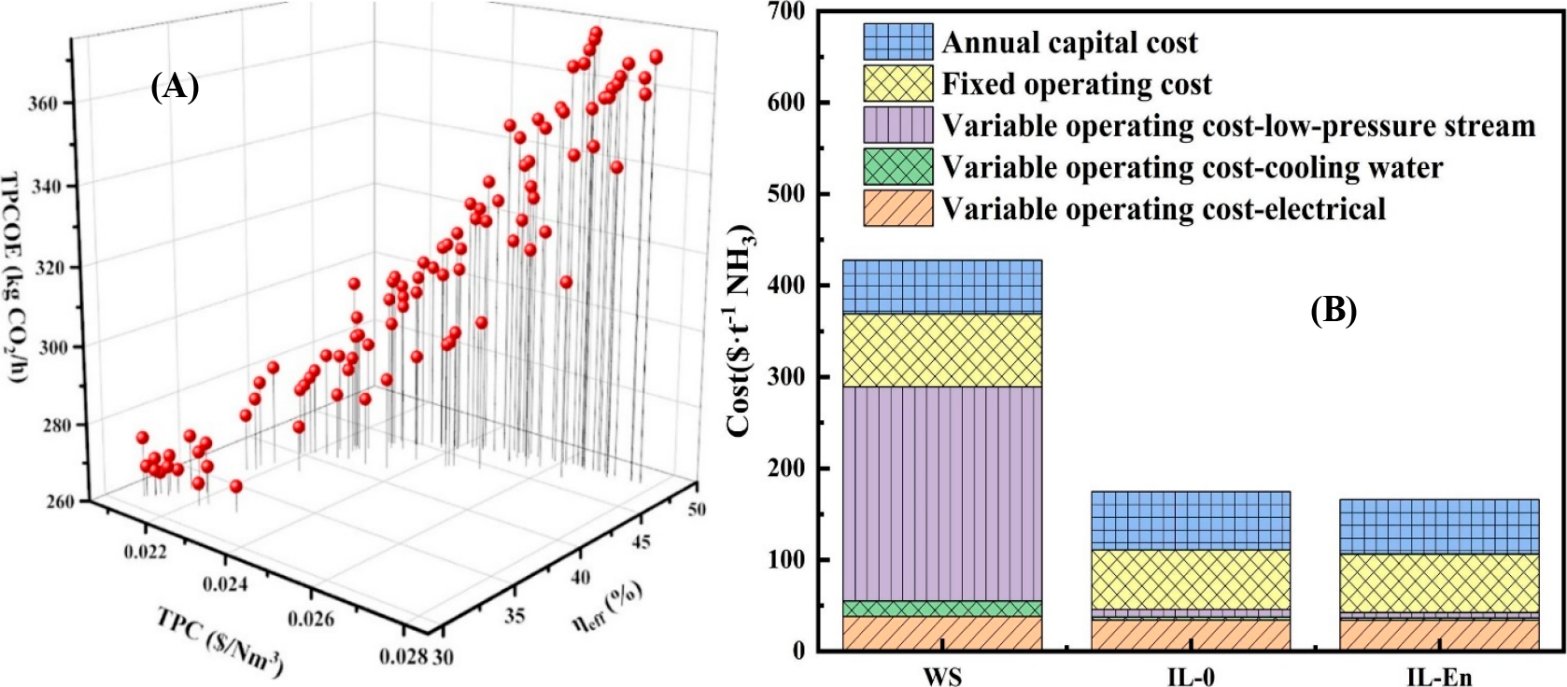
(A) Total purification cost plotted as functions of total process CO_2_ emissions and thermodynamic efficiency, Reproduced from ref (136). Copyright 2021 ACS. (B) Comparison of total costs for NH_3_ separation process using water, IL-based process, and IL-optimized process at 2 bar, 60 °C, 50 t/h (L), 8 stages, 0.02–0.1 bar, and 110 °C (desorbers). Reproduced from ref (131). Copyright 2022 Elsevier.

The IL-based ammonia absorption process has also been evaluated using commercial process simulators. The principal operating variables and KPIs of these processes are summarized in Table 8. Overall, the available studies modeled the absorption column without considering the mass-transfer kinetics (only in the equilibrium mode). The first study reported the NH_3_ absorption process simulation and optimization using two [NTf_2_]-based ILs compared with traditional water scrubbing.^136^ The authors use a multiobjetive optimization with three different objective functions (TAC, CO_2_ emissions and NH_3_ recovery). A genetic algorithm (NSGA-II) is used along several decision variables (theoretical column stages, feed stage, solvent flow, pressure and temperature of flash regeneration). The optimization of the operating conditions resulted in nine theoretical absorber stages and an operating pressure and temperature of 2 bar and 40 °C, respectively. The regeneration conditions were 120 °C and 0.1 bar in flash train columns. These operating conditions offered the possibility for satisfying the NH_3_ concentration standard of 1,000 ppm. A comparison of the technologies revealed that IL-based processes enabled energy consumptions of 58% and CO_2_ emissions reductions of 33%. The study included CO_2_ emission optimization processes as a selection criterion. Figure 42A shows the total purification cost plotted as functions of the total process CO_2_ emissions and thermodynamic efficiency. The lowest treatment costs occurred at the lowest efficiencies and CO_2_ emissions. This is principally because at that point, the least energy was consumed. Moreover, the thermodynamic efficiency decreased because the operating parameters affected the outlet streams. The study also showed how process simulations could be used to optimize processes and evaluate efficiencies and potential CO_2_ emissions. The separation of NH_3_ from CO_2_ in melamine tail gas streams was also evaluated using [bim][NTf_2_].^131^ For regeneration, different process configurations, including stripping columns, were analyzed. Figure 42B shows the total cost for both the proposed IL-based processes and the reduced costs with respect to the water-scrubbing process from 400 $/t (for water scrubbing) to 180 $/t. This reduction was principally attributed to the distillation regeneration train columns, which were necessary for water scrubbing. Specially, the process optimized using the stripping column with air for regeneration provided the optimal results of 165 $/t of NH_3_.

**Table 8:**
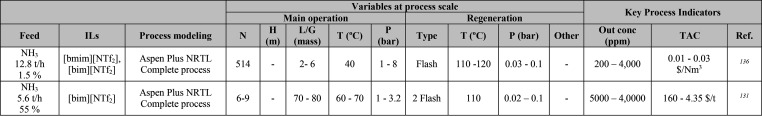
Summary of Variables at Process Scale and KPIs in IL-Based NH_3_ Capture Processes

Overall, [NTf_2_]-based ILs have been proposed for ammonia recovery, indicating that they may be a good alternative to water-scrubbing technologies because they decreased energy consumption and process costs. Again, the IL performance must still be evaluated considering mass-transfer kinetics to provide a more realistic approach to the operation.

### 4.5 Separation Processes by Liquid–Liquid Extraction

**Scheme 9:**
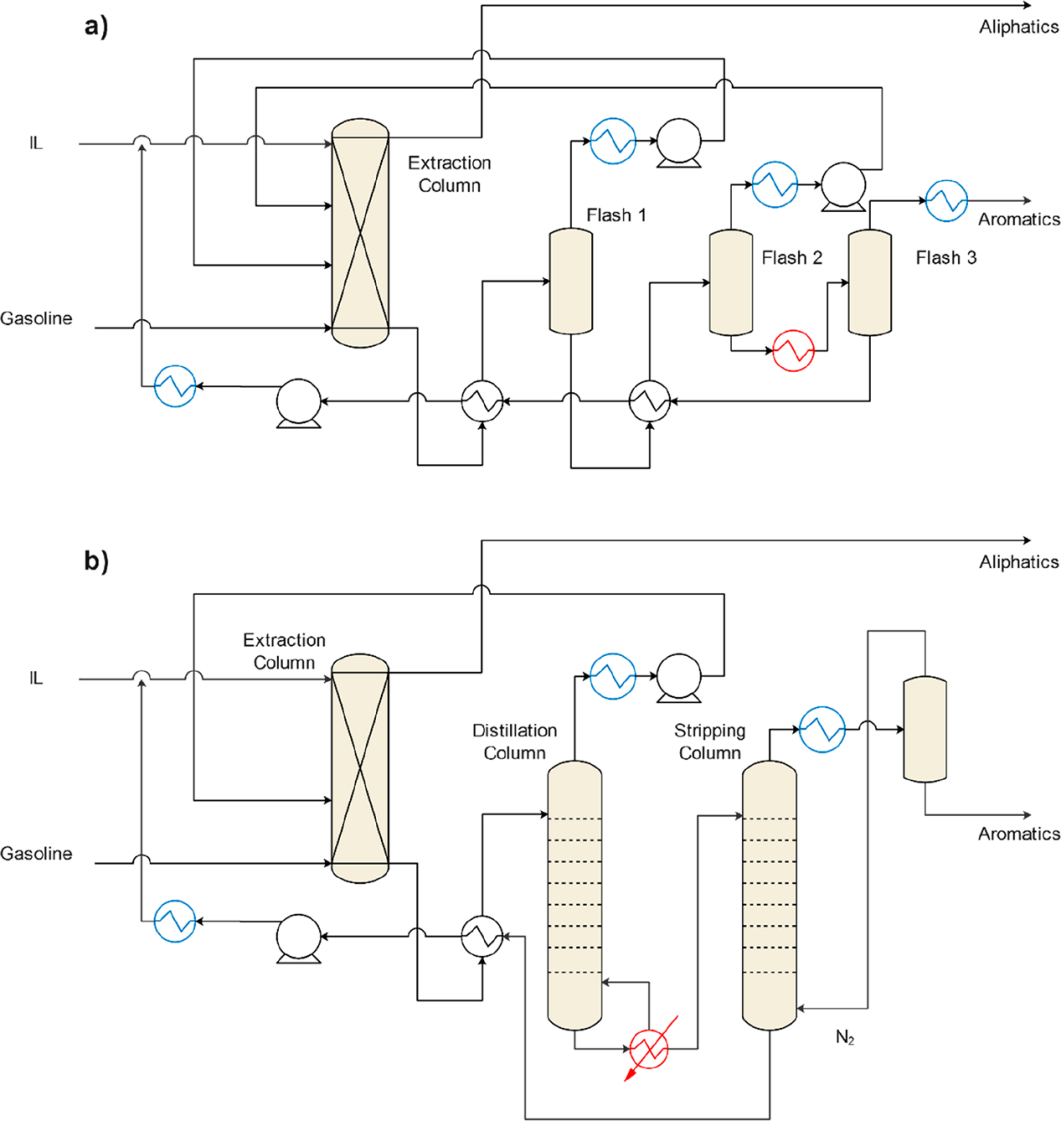
Representative Flow Diagrams of IL-Based Liquid–Liquid Extraction Process for Separating Aromatics from Gasoline

Liquid–liquid extraction is one of the most studied operations using ILs as solvents, covering extensive experimental and computational contributions. The main reason that this degree of development is supported is the lack of energy demand of liquid–liquid extraction itself. Experimental approaches have maximized the extractive properties, namely the distribution coefficient (ratio of solute compositions in the solvent-rich extract phase and solvent-poor raffinate phase) and selectivity (ratio of distribution coefficients of the extracted solute and a reference compound of the mixture to be separated), as the key indicators for discovering more desirable solvents.^230^ These indicators, typically named extractive properties, can be extended to multicomponent systems by considering the composition of all the desired compounds to be extracted (solutes of interest), and the rest of the compounds were collected as a family of other compounds. Process simulations have been applied in this field to evaluate the effects of IL extractive properties on the extraction performance to separate binary and multicomponent liquid mixtures, in terms of the product recovery and purity and solvent and energy consumptions required to reach a specific separation and, sometimes, include process cost estimations and environmental impacts. Table 9 lists the reported process simulation studies of IL-based liquid–liquid extractions. Owing to the relevant number of studies, ILs, and process schemes, aromatic separation from aliphatic hydrocarbons was set as the benchmark application for systematically presenting how process simulations help to develop IL-based liquid–liquid extraction, mainly for appropriately selecting adequate IL solvents and improving the process performance. The conceptual design of this process comprised an extraction step (decanter or multistage column) followed by two sections that promoted the extract stream purification (flash distillation units or distillation column) and solute/solvent separation (flash distillation unit or distillation/stripping column), as illustrated in the representative flow diagrams in Scheme 9. The conditioning stage, on the other hand, is relevant to heat the extract stream in the purification and recovery steps and cool the regenerated solvent to the extractor operating conditions. Finally, if substantial solvent is lost in the raffinate, this stream must be further purified, enabling solvent recycling (which is not considered in Scheme 9).

**Table 9:**
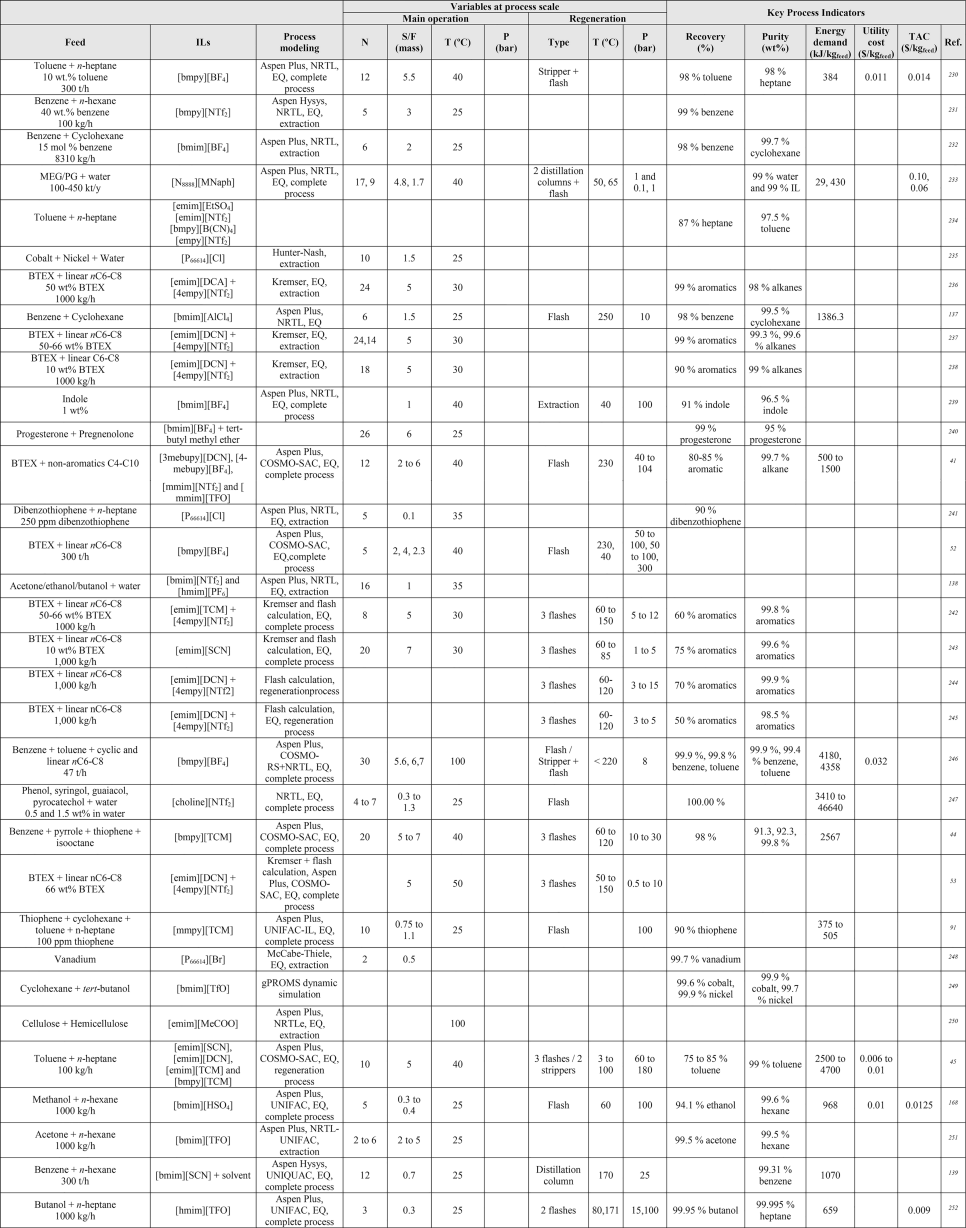

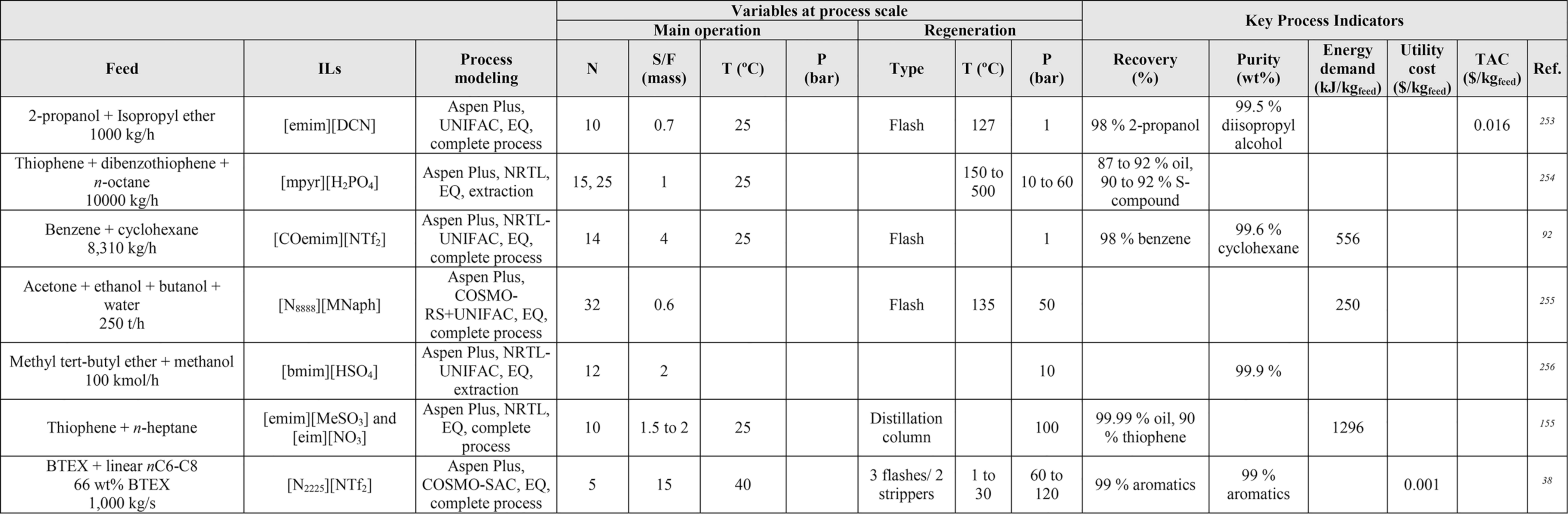
Summary of Variables at Process Scale and KPIs in IL-Based Liquid–Liquid Extraction Processes

#### 4.5.1 Ionic Liquid Performance in Liquid–Liquid Extraction Unit

The availability of ILs accounts for no fewer than 10^6^ species, which imposes a utopic challenge for experimentally solving the problem for selecting a proper extraction solvent. Some models, such as COSMO, also available in process simulators, have been widely applied as theoretical tools to evaluate the extractive properties of ILs and their role in separating aromatic–aliphatic mixtures, widening the transferability from extractive properties to the process scale.^257^ Both binary system (such as toluene extraction from *n*-heptane) and multicomponent (such as extraction of benzene, toluene, and xylenes from pyrolysis gasoline) models have been selected as guidance in process simulations.^230,238^ Liquid–liquid equilibrium (LLE) data at the infinite dilution of solutes have been used in simplified extraction models.^258^ The Kremser shortcut method implemented in spreadsheets was another alternative to, at least, translate extractive properties for extractor design parameters, namely the number of stages (N), solvent to feed (S/F) ratio, and IL properties,^236^ enabling recoveries and purities to be determined as functions of these design parameters. However, most studies predicted LLE values for realistic mixture compositions—considering the potential composition dependence of partial miscibility—to model extractions using a one-stage decanter^53^ or multistage countercurrent extractor column (as an EXTRACT model in the Aspen Plus simulator).^241^ Frequently, the research community has reported liquid–liquid equilibria on a molar basis, avoiding the potential impact of the MW on certain systems. By focusing on the extraction of toluene from *n*-heptane, several IL candidates, such as dicationic ILs,^259^ ILs comprising large anions (i.e., tetrathiocyanocobaltate),^260^ or functionalized ILs,^261^ have moved from high- to moderate-capacity solvents when considering toluene distribution coefficients on molar or mass bases, respectively, owing to the high molecular weights of the selected ILs. Therefore, mass-based extractive properties have been recommended for properly analyzing process behaviors in simulation studies. In a very recent study,^38^ Navarro et al. used the COSMO-SAC-based/Aspen model and ILUAM database^51^ to screen the mass-based extractive properties of 100 ILs for separating a toluene + *n*-heptane binary mixture and a multicomponent pyrolysis gasoline mixture to achieve commercial aromatic recoveries and purities.

**Figure 43:**
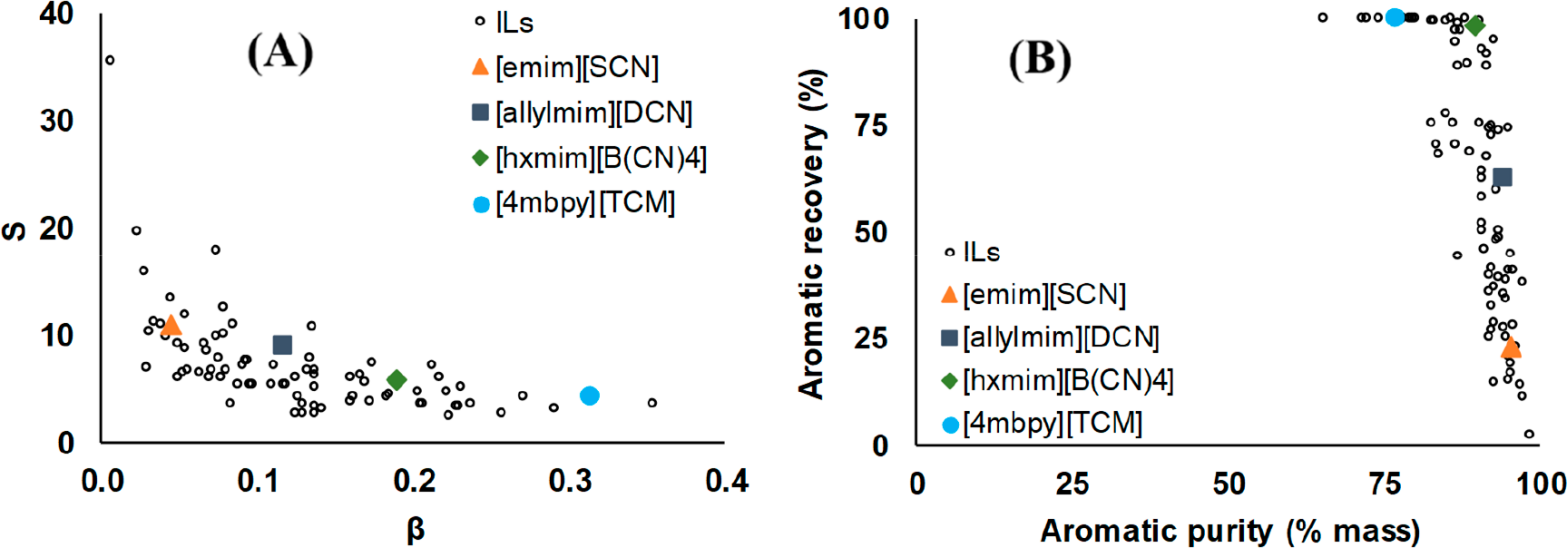
(A) Toluene/*n*-heptane selectivity (S) and toluene distribution ratio (β) (in mass units) between extract and raffinate phases for studied ILs in single equilibrium liquid–liquid extraction with S/F = 1 mass basis and (B) aromatic recoveries and purities in extract phase from liquid–liquid extraction in countercurrent extraction column (N = 5, S/F = 5) for binary mixture of (*n*-heptane + toluene) with mass fraction of 0.34 of *n*-heptane at 40 °C. Reproduced from ref (38). Copyright 2023 Elsevier.

**Figure 44:**
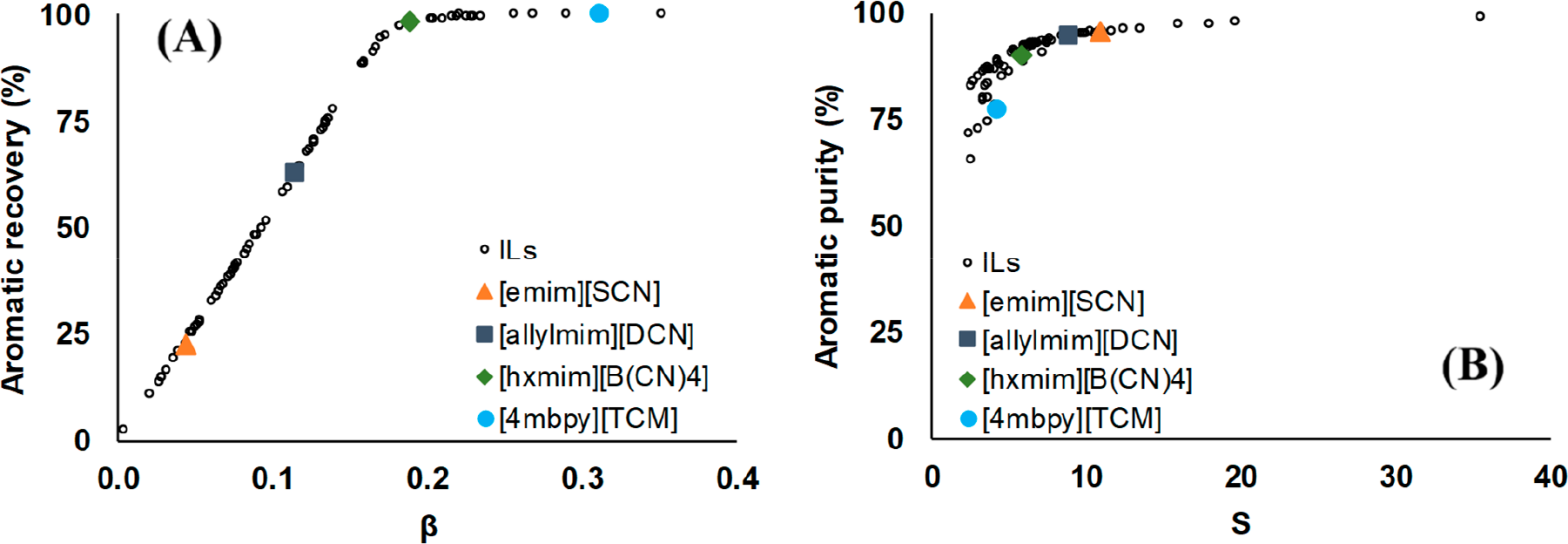
Relationships between (A) the aromatic recovery in extraction unit and aromatic distribution ratio (β) of IL and between (B) aromatic purity in extraction unit and toluene/*n*-heptane selectivity (S) of IL. Recoveries and purities obtained using countercurrent extraction column (N = 5, S/F = 5) and binary mixture of (*n*-heptane + toluene) containing mass fraction of 0.34 for *n*-heptane. Reproduced from ref (38). Copyright 2023 Elsevier.

Figure 43A compares the toluene/*n*-heptane selectivity (S) to the toluene distribution coefficient (β) for a one-stage simulation with a solvent-to-feed ratio (S/F) of 1, indicating the coupling inverse effect of extractive properties for obtaining ILs that had a high extractive capacity and low selectivity (such as [4mbpy][TCM], ILs highly selective but with a low separation capacity (such as [emin][SCN]) and several ILs with intermediate S and β values (such as [Almim][DCN] and [hmim][B(CN)_4_]). Figure 43B relates the calculated extractive properties of IL to the IL behavior as extraction solvent to separate a toluene + *n*-heptane mixture using an extract column comprising five stages (N) and S/F = 5. A clear relationship was observed between the aromatic recovery and purity depending on the IL characteristic, as highlighted by the four selected ILs comprising cyano-based anions, which are well-known candidates in the literature.^261^ Changing the IL extractive properties by varying the structural features, one can progressively move from a high-purity, low-recovery IL (such as [emim][SCN]) to a low-purity, high-recovery IL (such as [4mbpy][TCM]). Notably, literature lacks these systematic studies and researchers have selected solvents before moving to the process scale, which is one of the weak points. Figure 44 compares the calculated extractive properties and extractor performance. For a certain extractor specification, the higher the distribution coefficient, the higher the solute recovery. However, as clearly shown in Figure 44A, the asymptotic trend negligibly increased the distribution coefficient above 0.2 under the claimed conditions. Thus, even for a simple and well-known correlation between the extractive properties and extractor performance, the simulation is a necessary tool for determining the impact of changing an extractive property. This is even clearer in Figure 44B for the coupling of the toluene purity and toluene/*n*-heptane selectivity because a substantial increase in the S value slightly improved the toluene purity in the extract stream. Because these behaviors were only related to a single evaluated S/F ratio, the role of the extractive properties was clearly related to the operation design and solvent consumption.

**Figure 45:**
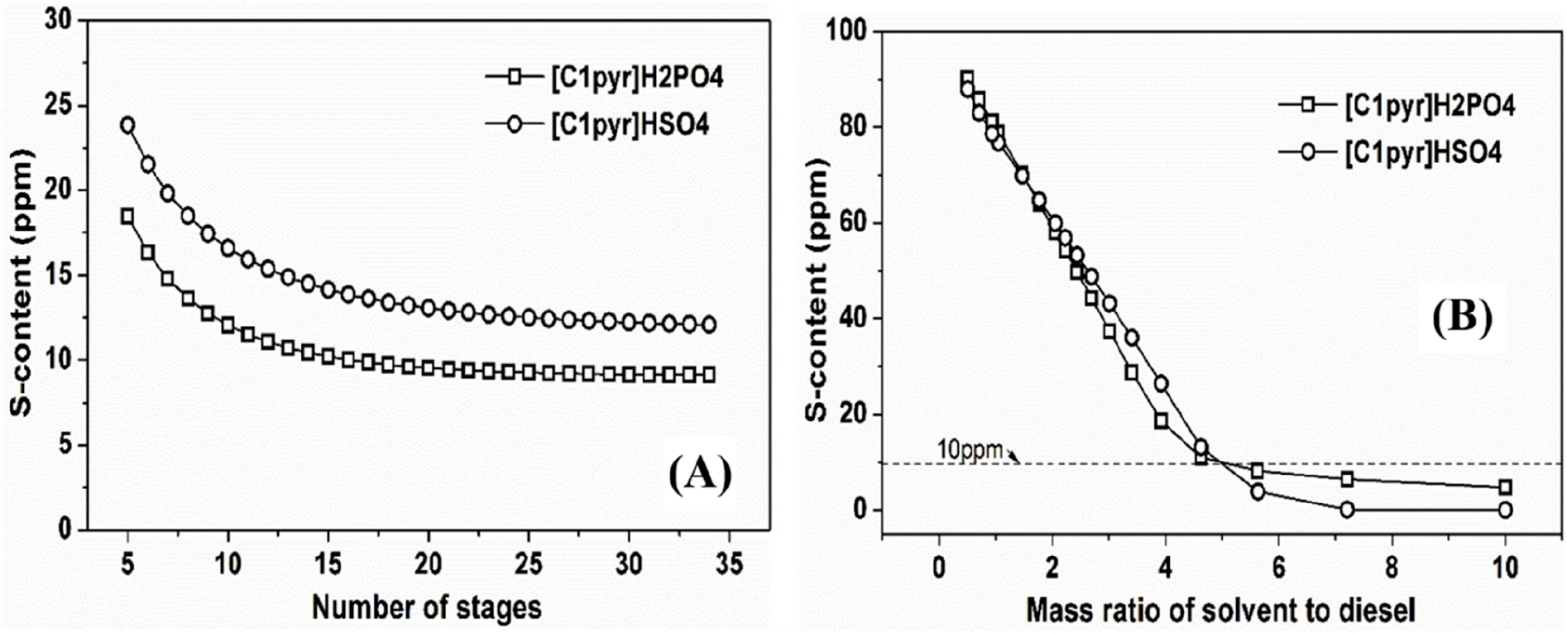
Correlation between extractor performance (thiophene content in treated diesel) and design parameters (A) N for S/F = 5 and (B) S/F for N = 15 in IL-based extraction of thiophene from diesel model at 25 °C and 1 bar. Reproduced from ref (254). Copyright 2021 Elsevier.

Sensitivity has been widely analyzed using process simulations to evaluate IL-based liquid–liquid extraction processes. Several studies have reported recoveries and purities as functions of N and S/F by considering a limited number of ILs.^138,230,234,236−238,240,241,254^ Thus, Figure 45 shows the solute content in treated diesel plotted as functions of the number of stages and solvent-to-diesel ratio. Although the main contribution of these studies was the design of the extraction stage to assess the solute recovery specification using the selected ILs, a clear limitation was that the previously selected IL conditioned the obtained results. Additional limitations of this type of analysis were the neglect of recycling streams and failure to evaluate the solvent regeneration and solute purification with the associated energy duties and operating costs. Accordingly, a description of not only the extractor but also the complete separation process is relevant.

#### 4.5.2 Complete Liquid–Liquid Extraction Process Modeling for Technoeconomical Analysis

Regarding the solute purification and IL regeneration in aromatic/aliphatic separations, many options were available to draw flow diagrams of extraction processes (Scheme 9). In aromatic separations, an aromatic/aliphatic selectivity over 440 ensured an extract that was sufficiently pure to avoid purification, as claimed by Meindersma et al.^262^ More efficient separation trains resulted in lower energy consumption, as they enabled the improved control of the recycled aromatics and mitigation of the S/F ratio. The literature has explored several schemes ranging from only one flash distillation unit to two distillation/stripping columns. Table 9 (in the process modeling column) lists the more extended separation trains associated with IL-based liquid–liquid extraction processes to purify the solute and regenerate the IL. Notably, although an extractor and a flash distillation unit have been widely proposed in flow diagrams and have been included in several studies,^168,231,247,248,253−256,263^ this represented more of an ideal process configuration rather than a plausible scenario. Other studies have displayed a flowsheet with two distillation units, one devoted to the IL/solute separation and the other to the separation of the IL dissolved in the raffinate.^232,251,252^ This approach had the same problems that had only one flash distillation unit for the extraction. The one-flash approach was only valid when the compound to be separated from the solute, in the IL matrix, showed a very high relative volatility because, otherwise, the solute recovery and purity in the flash would be inadequate. In fact, the evolution from one to two flash distillation units increased the recovery from approximately 10 to 70% in aromatic extractions from several refinery streams^244^ with the same aromatic purity specification of 99.9 wt %.^264^ Following this argument, a stripping column was the best device for performing the separation to obtain both high recoveries and purities of aromatics, as demonstrated elsewhere in the literature.^45,242^ In addition to this improvement, the paradigm for operating with ILs at atmospheric pressure throughout the process has been proposed elsewhere in the literature.^45^ Flash distillation units demanded high vacuum pressure to avoid elevating the temperature over the maximum operation temperatures (MOTs) of the ILs because ILs decomposed at moderate temperatures (i.e., approximately 100 °C–200 °C) and to address specifications because only the pressure was tunable when the temperature was set at the MOT. This was a clear contribution of process simulations to the conceptual design of IL processes. The first studies on IL-based liquid–liquid extraction suggested the difference in the volatility was an easy win in favor of ILs for designing processes; however, thermal stability issues have emerged, and the temperature clearly increased the vacuum demands. Canales and Brennecke^265^ suggested the combination of cyano-containing anions ([SCN]^−^, [DCN]^−^, and [TCM]^−^) and [NTf_2_]^−^ owing to their competitiveness with conventional solvents. These conclusions, together with the results of thermal stability studies,^266,267^ reduced this list to almost exclusively [NTf_2_]^−^ and [TCM]^−^ anions for solvents having adequate extractive properties and thermal stability. With respect to aromatic/IL separation, the use of a flash distillation unit implied extreme vacuum conditions, with an operating pressure in the 0.01–0.05 bar range to separate BTEX extracted from several refinery stream models. The use of a stripping column fed with a stripping agent, such as N_2_, clearly reduced the energy demand and improved the separation performance to compensate for higher investment-associated costs.^45^ Alternatively, Navarro et al.^45^ changed the separation train from three flash distillation units to two stripping columns and a flash distillation, which drastically reduced the energy consumption. Of all the distillation approaches and common systems that the aromatic/aliphatic separation represented, the solute/IL separation could be envisioned by liquid–liquid extraction within a back-extraction scheme. This could be useful for other kind of systems. For instance, Chen et al.^235^ simulated a complete [P_66614_][Cl]-extraction-based process to separate Co and Ni, and Singh et al.^248^ similarly extracted V with the same approach. Additionally, Jiao et al.^239^ extracted indole with [bmim][BF_4_] in an exclusively liquid–liquid extraction-based process.

**Figure 46:**
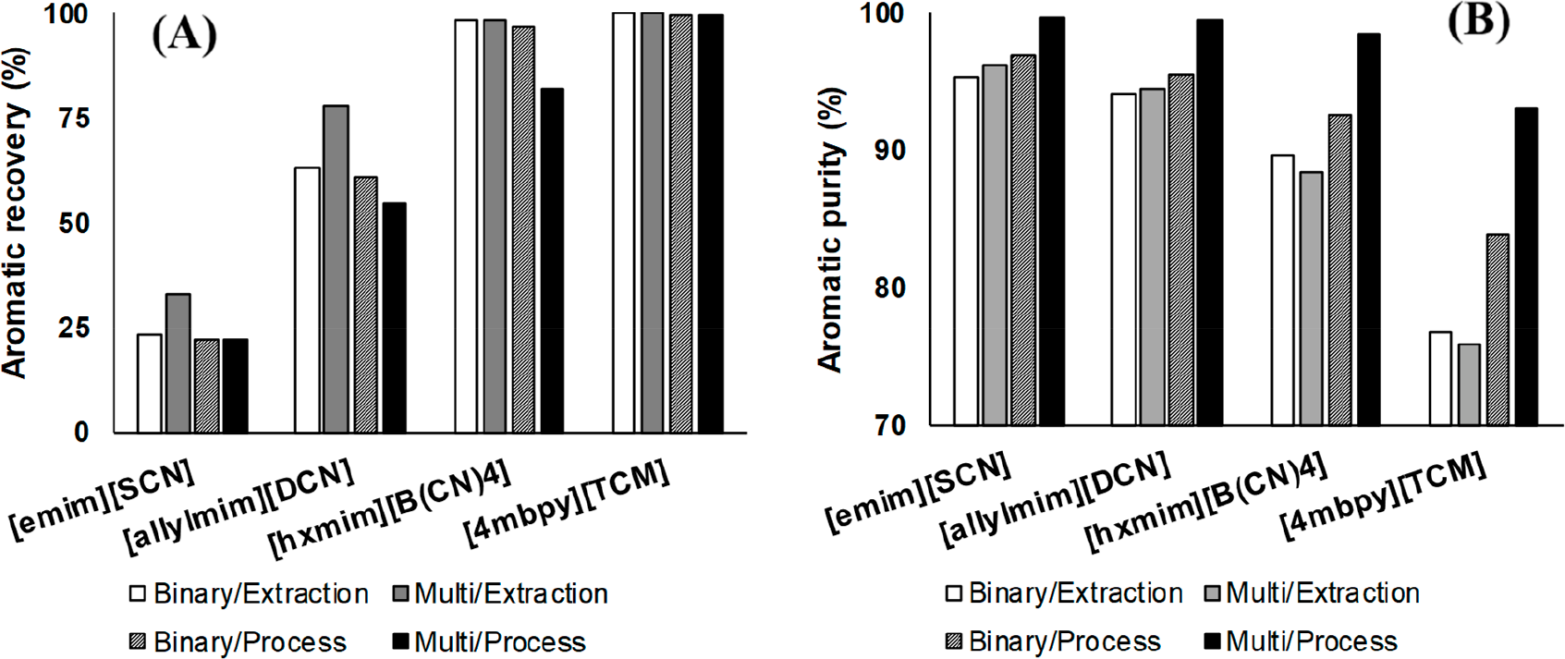
Comparison of aromatic (A) recovery and (B) purity as functions of process description (extraction unit/complete process) and refinery stream model (binary/multicomponent). In all cases, countercurrent extraction column with N = 5 and S/F = 5 was used. Reproduced from ref (38). Copyright 2023 Elsevier.

Figure 46 introduces an interesting picture that simultaneously moves from the extractor operation to the entire process and from binary to multicomponent systems, using four selected cyano-based ILs that have representative extraction properties at given N and S/F values in the extraction stage and a three-flash-distillation-based separation train. The resulting aromatic recoveries and purities were mainly determined by the extractive properties of the ILs rather than substantially influencing the regeneration process or feed conditions. Toluene was extracted more efficiently using ILs presenting high β values (such as [4mbpy][TCM]). In contrast, this could result in substantial *n*-heptane contamination in the aromatic product owing to the low IL selectivity. The opposite behavior was observed for ILs presenting a high selectivity and low extractive capacity for obtaining a low aromatic recovery but a high product purity. However, the IL regeneration train substantially increased the aromatic purity without severely decreasing the toluene recovery. The use of realistic multicomponent systems (pyrolysis gasoline) in process simulations, on the other hand, provided relevant results. Thus, the introduction of benzene to the multicomponent model systematically improved the aromatic recoveries in the extractor and deteriorated the aromatic recovery of the entire process (Figure 46A) owing to the improved aromatic distribution coefficients and lower aliphatic/aromatic relative volatility, respectively, compared with those of the toluene benchmark. In addition, by covering a more realistic C6–C8 aliphatic fraction, the aromatic purity progressively enhanced, first in the extractor because the solubility of *n*-octane was lower than that of *n*-heptane and then in the recovery section because the *n*-hexane volatility facilitated the aromatic purification (Figure 46B).^268,269^ Therefore, process simulation could help to select representative but more complex separation systems covering not only the extractor but also the entire process. Sometimes, a complex refinery cut could not be accurately represented or managed at the experimental level owing to the difficulty in analyzing the compositions, whereas a robust process simulation could serve as guide, representing an ideal solution to complex multicomponent mixtures. Larriba et al. studied multicomponent extractions by combining the experimental approach with the Kremser method,^236−238,243,260^ whereas Ferro et al.^41^ progressed to investigate naphtha comprising more than 28 compounds using the COSMO/Aspen methodology. Navarro et al., on the other hand, designed a separation train based on three flash distillation units to satisfy commercial specifications in the aromatic product, mimicking sulfolane standards.^243−245,260^ However, shortcut methods combined with experimental determination measurements imposed a clear limitation to deal with recycling streams and their impacts on the solvent consumption and energy expenses. To separate aromatics and aliphatics, de Riva et al.^52^ focused on recycling streams in the synthesis process and found that the separation performance was low at the end of the process.

**Figure 47:**
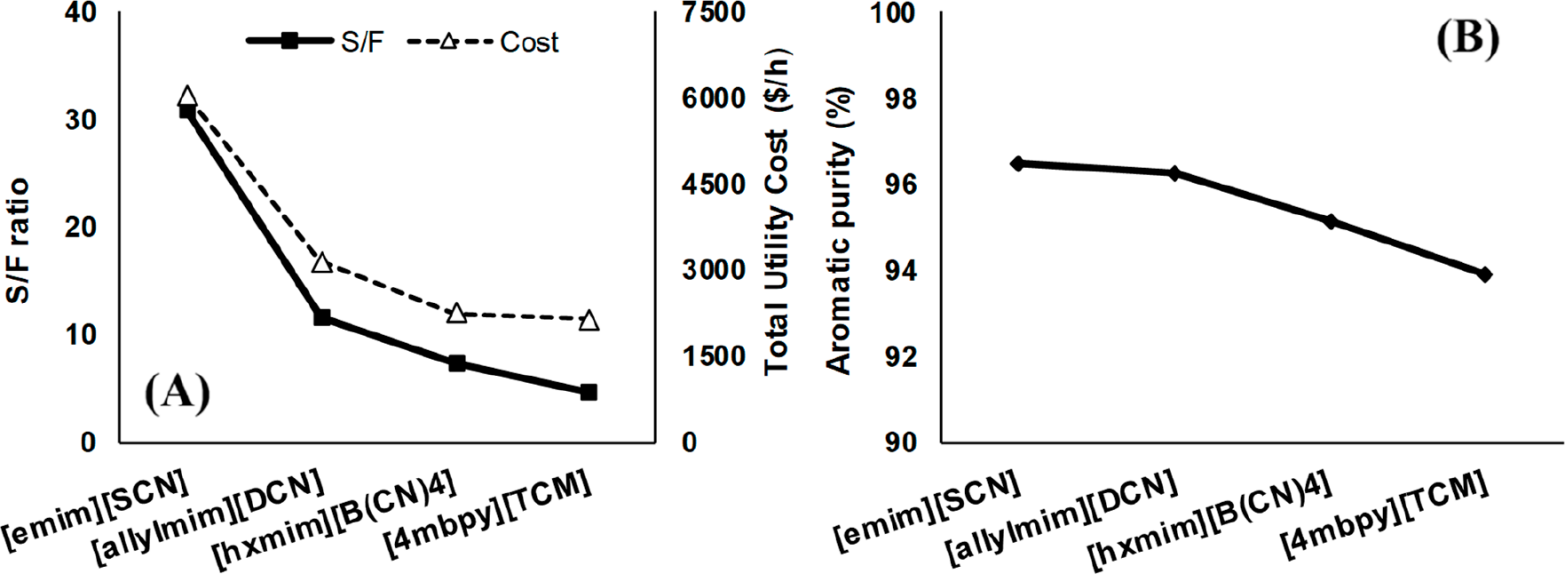
(A) S/F ratio and utility costs and (B) aromatic purity plotted as functions of IL calculated for specified aromatic recovery of 99% using countercurrent extraction column (N = 5) for separating multicomponent refinery stream. Reproduced from ref (38). Copyright 2023 Elsevier.

Another contribution of process simulations in the development of IL-based extraction processes was the evaluation of chemicals and energy consumptions to determine a separation specification. Figure 47 presents an interesting picture for evaluating the role of the ILs in terms of the solvent consumption, utility costs, and product purity for a 99% aromatic recovery specification using a multicomponent gasoline model and the entire process, including extraction and solvent regeneration stages. Clearly, more selective ILs (such as [emim][SCN]) promoted enhanced product purity but remarkably higher solvent flow (S/F) and, consequently, much higher utility consumption and utility costs. In contrast, ILs that had a high extraction capacity enabled efficient recovery with low solvent and energy consumption at the expense of low product purity. Therefore, process simulations facilitated the understanding of energy consumption and purity ranges and, accordingly, the IL-associated advantages and disadvantages for a single specification.

#### 4.5.3 Process Improvements in Liquid–Liquid Extraction Based on ILs

**Figure 48:**
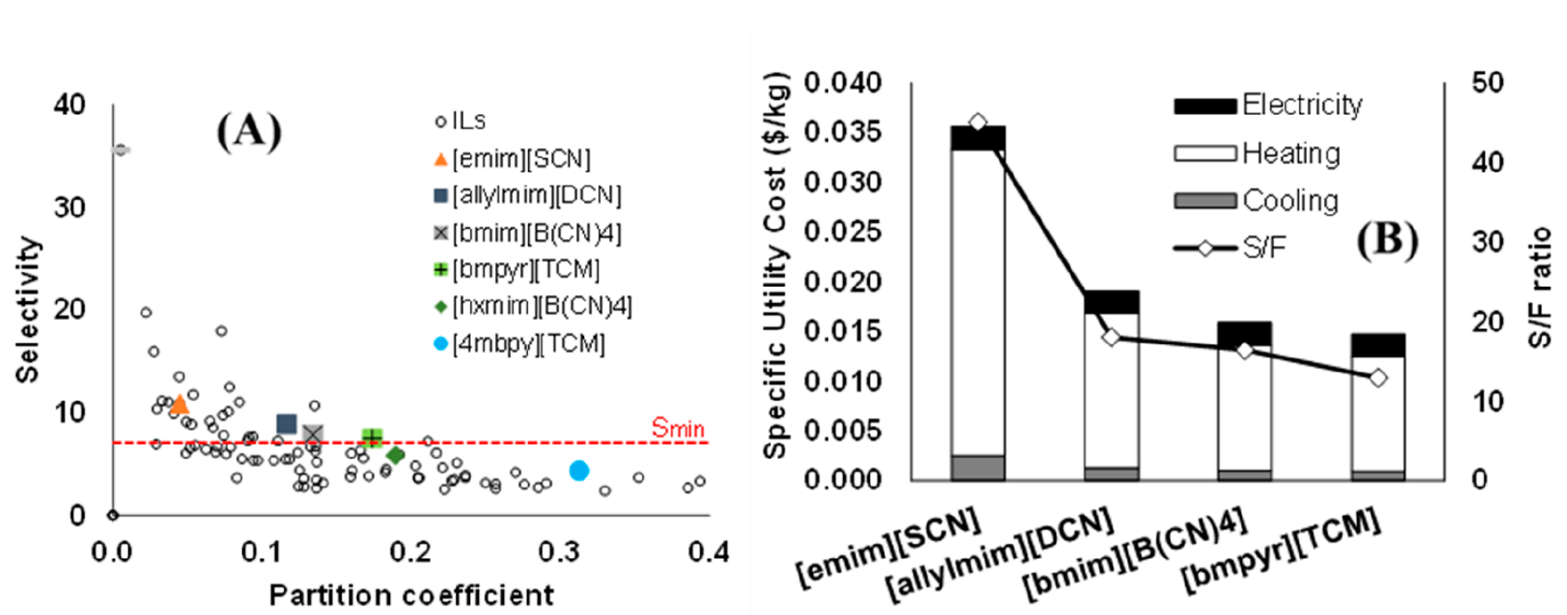
(A) Selectivity and distribution ratio calculated for single equilibrium stage and minimum selectivity calculated to recover 99% of aromatics in overall process at aromatic purity of 99 wt %. (B) S/F ratios and utility costs calculated to recover 99% of 99% pure aromatic product in complete separation process using countercurrent extraction column (N = 20) for separating multicomponent refinery stream. Reproduced from ref (38). Copyright 2023 Elsevier.

Furthermore, liquid–liquid extraction processes are usually driven by two main specifications in a classical approach that covers product recovery and purity (in this case, aromatic recovery, and purity). In Figure 48, the four IL candidates have been evaluated to address high recovery and purity specifications simultaneously. The BTX recovery and aromatic purity were set at 99 and 99 wt %, respectively. Although the two most selective ILs simultaneously satisfied both specifications, operation at S/F ratios over 20 and unaffordable utility costs were both required. On the contrary, the two ILs that had the highest distribution coefficients could not meet the purity specifications. This result has rarely been found elsewhere in the literature mainly because specifications were not the main objective of the research studies and were very different from commercial standards and because the separation train was not the aim of the study and lacked sufficient rigor (temperatures above the MOT or extreme vacuum pressures). However, other useful studies adopted similar approaches. Lyu et al.^137^ explored recoveries and purities together with energy consumption at the process scale for separating benzene + cyclohexane and suggested [bmim][AlCl_4_] after evaluating the extractive properties of other ILs. de Riva et al.^52^ showed the relationship between the recoveries, purities, and energy consumption within several process schemes and found that [bmpy][BF_4_] was the most suitable solvent to extract BTEX from a naphtha model. Oh et al.^246^ found the suitability of the same IL for a similar aromatic extraction process by computing interesting objective functions, such as S/F·N and TAC. Larriba et al.^53^ defined a new variable at the process scale for evaluating the composition of a mixture of ILs—[emim][DCN] + [4empy][NTf_2_]—through recovery, purity, and energy consumption criteria for recovering aromatics from pyrolysis gasoline. Song et al.^91^ found the S/F ratio, recovery, and energy consumption for desulfurizing fuels and simplified the purity constraint, as thiophene was an impurity at concentrations on the order of parts per million. Addouni et al.^139^ evaluated a mixture comprising an organic solvent and [bmim][SCN] to separate benzene from *n*-hexane by optimizing the reboiler duty and IL purity outputs for different S/F ratios. Peng et al.^92^ simplified the evaluation of the desulfurization of fuels following recoveries, purities, and S/F ratios, the latter of which indirectly represented the energy consumption. Other useful specifications to set were the concentration limit of the solute in the raffinate stream and the solute purity in the product stream, as found elsewhere in the literature.^53,241^ In addition, although the process could operate based on a recovery formulated for an individual compound that used to be the limiting solute, the operation depended on the envisioned technical solution and the product purity for all the extracted solutes.^243^

**Figure 49:**
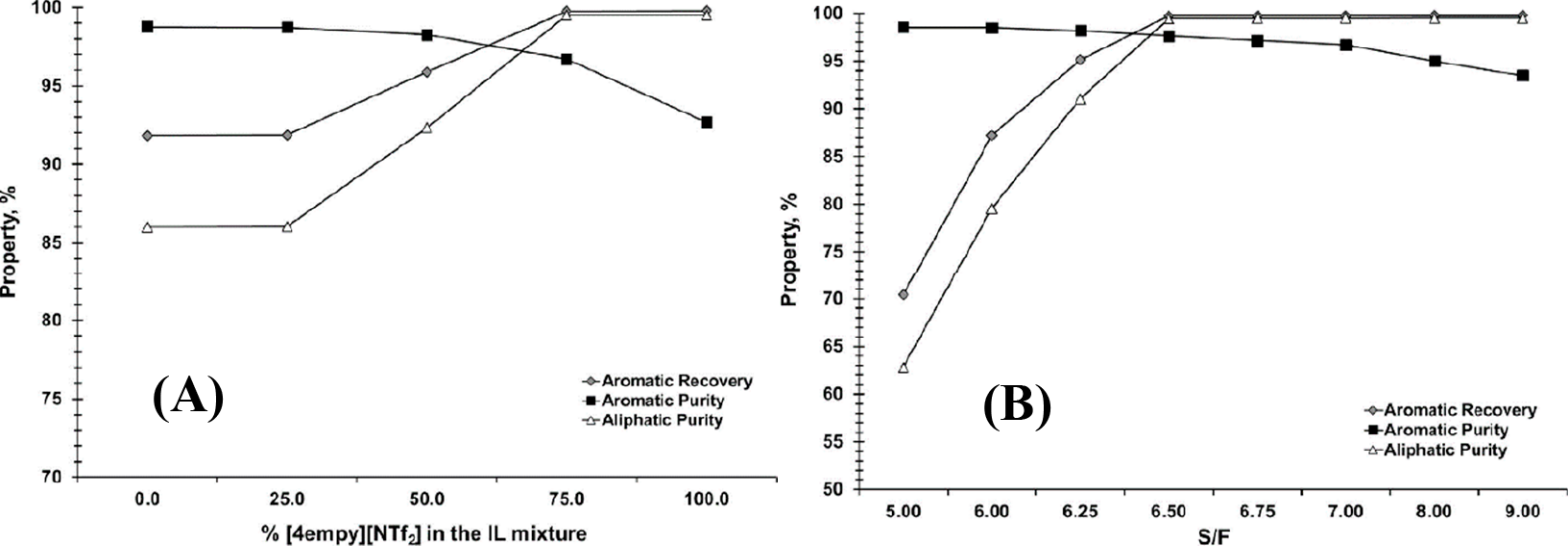
Optimization of IL-based binary mixture composition at process scale for [emim][DCN] + [4empy][NTf_2_] hybrid solvent in separation of BTX from pyrolysis gasoline. Reproduced from ref (53). Copyright 2018 Elsevier.

These results revealed the advantage for selecting IL structures based on process-scale results rather than merely extractive property criteria. Consequently, processes have been simulated to design IL-based solvents for enhancing the extraction performance at the process scale. The S and β values shown in Figure 43A suggested that the [B(CN)_4_]^−^ and [TCM]^−^ anions completed with other cations to modulate extractive properties. For instance, [hxmim]^+^ was replaced by [bmim]^+^. This shortened the alkyl chain and produced [bmim][B(CN)_4_], which was less energy demanding than the [emim][SCN] and [Almim][DCN] ILs (see results in Figure 48B). Another example involved replacing the pyridinium ion in [4mbpy][TCM] with a pyrrolidinium ion, which had the same butyl group substituent, [bmpyr][TCM], which optimized the results within the specifications for the treated IL sample (see results in Figure 48B). Some recent examples based on this strategy were found in the literature, including the evaluation of substituent positions at the process scale to desulfurize fuels^91^ and the evaluation of several ILs at the process scale to separate acetone and *n*-hexane.^251^ A different approach found in the literature involved the use of IL mixtures or [IL + solvent] hybrid systems to modulate the solvent properties and performance at the process scale. The concept of IL mixtures was developed for simultaneously modulating extractive and physical properties to design solvents that had the desired intermediate properties at the mesoscale. The most studied ILs mixture comprised [emim][DCN] and [4empy][NTf_2_] owing to the high selectivity and low viscosity of the former and the high capacity and high density of the latter. Favorable mixtures containing 0.7 mol % [emim][DCN] have been reported, and the extractive, physical, thermal, and relative volatility properties have been experimentally measured.^268,270^ Kremser-approximation-based simulations have been conducted to model the extractor and ad hoc simulation algorithm developed to simulate flash distillation units under stream extraction conditions. The results showed that the mixture was a feasible solvent compared to neat IL for separating aromatic–aliphatic mixtures.^236−238,244,245^ A subsequent study conducted by Larriba et al.^53^ analyzed the mixture composition in the complete process and clearly indicated that 0.3 mol % [emim][DCN] was correlated with enhanced process performance (energy demand, solvent consumption, and aromatic purity and recovery, see Figure 49).

Alternatively, mixtures comprising ILs and organic solvents could be considered to improve the process efficiency. In fact, this approach has been quite common in the literature for improving organic solvent processes by adding an IL that increased the selective interaction with aromatics. Addouni et al.^139^ evaluated sulfolane as an organic solvent and [bmim][SCN] in the dearomatization of refinery streams, whereas Vitasari et al.^240^ evaluated a mixture of [bmim][BF_4_] + *tert*-butyl methyl ether at the process scale to separate a mixture of progesterone + pregenolone.

**Figure 50:**
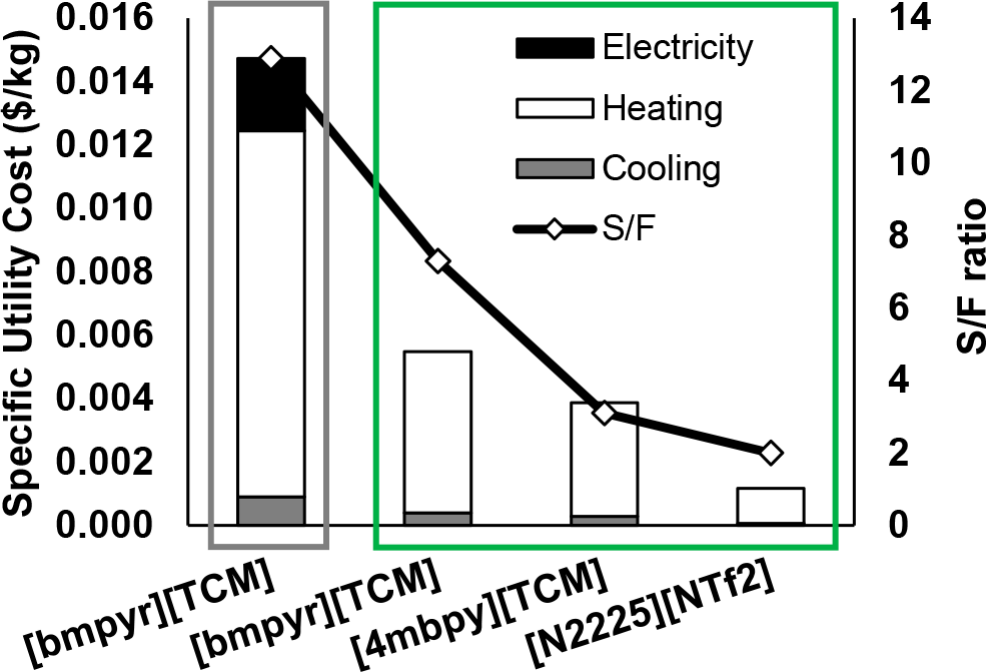
Specific energy consumptions and S/F ratios for selected ILs to recover 99% of aromatics in overall process at aromatic purity of 99 wt % via two process configurations shown in Scheme 9 (A in gray and B in green) using countercurrent extraction column (N = 20) for separating multicomponent refinery stream. Reproduced from ref (38). Copyright 2023 Elsevier.

All these results meant that coupling process simulations and IL design enabled the improvement of the process insights for a complex specified problem. Alternatively, efforts have been made to design more efficient separation trains by optimizing IL process pairs to maximize the product recovery and purity with minimal chemical and energy consumptions. Thus, the use of the first stripping column (Scheme 9B) instead of flash distillation (Scheme 9A) reduced the content of aromatics recycled to the extractor, whereas the use of the N_2_ stripping agent in the second column avoided the vacuum costs and limited the heating requirements. This process configuration enabled the re-evaluation of IL candidates that had high aromatic distribution ratios, such as [bmpyr][TCM], [4mbpy][TCM], and [N_2225_][NTf_2_]. The required S/F ratios and energy consumptions are displayed in Figure 50. Clearly, the redesign of the separation train enabled the use of more favorable high-capacity IL candidates to satisfy the product quality standards with minimal energy demands and avoid the vacuum requirements, which substantially lowered the utility costs. One of the ILs selected in this process simulation overview, [4mbpy][TCM], was among the most studied ILs in the literature, whereas [N_2225_][NTf_2_] emerged as a feasible structure that was easily synthesized and had thermophysical properties.^271^ This study has revealed that the use of process simulations could provide opportunities for developing more efficient IL-based systems to separate aromatic–aliphatic mixtures while simultaneously considering the product and process design.

Although process simulations have also enabled technoeconomic comparisons between IL-based technologies and current industrial standards, few examples that compared liquid–liquid extraction and extractive distillation were found in the literature^45^ (Section 4.6). Although some studies have used TAC criteria to approximate a comparison of liquid–liquid extraction and extractive distillation (see refs (168, 230, 233, 246,252, 253, 256, 263)), cost estimations were limited owing to the lack of kinetic data to model and size the extraction column and reliably price industrially produced ILs. Only Meindersma et al.^230^ and UOP compared IL-based processes against the sulfolane process. Because they claimed that CAPEX and OPEX could be reduced by 35–70% using ILs in a refinery stream containing approximately 10 wt % aromatics, the cost must soon be estimated because the use of ILs seems to improve costs, even considering conservative IL prices.^230^ Regarding the transport properties, the EXTRACT model from Aspen Plus, which is the more extended approach for simulating IL-based extractors, does not enable the effects of the mass transfer on the separation to be considered. A study by de Haan et al. have pointed out that the hydrodynamic behaviors of liquid–liquid contactors with either low-viscosity ILs or conventional solvents were equivalent.^272^ Delgado-Mellado et al., on the other hand, determined the diffusion coefficients of hydrocarbons in low-viscosity ILs and found that they were competitive with conventional solvents, such as sulfolane, in mass transfer features.^273^ Therefore, the absence of the rate-based dimension in the extractor simulation could be controlled by limiting IL candidates to those showing competitive viscosities. Although few studies have modeled and evaluated the mass transfer in an extractor, it was evaluated for the separation of ethanol + *n*-heptane with [bmim][MeSO_4_].^274−276^ However, the physical properties must be properly defined in the process model because they also affect distillation columns, heat exchangers, pumps, etc.

#### 4.5.4 Environmental Impacts Analysis of Liquid–Liquid Extraction Process Based on ILs

Finally, environmental impacts should be considered as an IL selection criterion to improve the sustainability of IL-based extractive separations, as proposed by Diaz et al. in aromatic extractions^277^ and extended by Clarke et al. to all solvents.^278^ Hernández et al.^48^ extracted IL solutes in the best example for understanding the LCA methodology and how not only the compound-related environmental impacts but also the compound-related mass flows were relevant in liquid–liquid extraction. In this regard, an IL-based liquid–liquid extraction process has two clear IL-amount-related calculations. First, in the process holdup, the IL that remains in the process to fill the equipment and pipes is relevant to the investment costs but negligible for evaluating the IL impact because this amount does not leave the process and is much lower than the amounts in the inlet and outlet streams in a representative time unit (i.e., one year). However, the second contribution is related to the IL losses and, thus, the IL composition of the process. IL losses are only related to losses by raffinate saturation in the solvent (the solubility of the IL in the raffinate) as the vaporization losses are negligible. The toxicity and other impacts imputable to the process, such as the GWP, are dependent on not only the IL features but also the IL losses.^48^ Thus, a less-toxic IL that shows a much higher solubility in the raffinate will have a stronger impact than using an almost immiscible solvent that has a higher toxicity profile and would comprise a negligible portion of the IL. Even a feature that is apparently unrelated to the process scale, such as toxicity, must be simulated for properly evaluating its real impact. Toxicity and LCA criteria are very scarce in the literature for liquid–liquid extraction processes in which the IL is a solvent. Vásquez et al.^250^ evaluated the LCA for cellulose production using [emim][MeCOO] as a solvent, and Zhang et al.^168^ monitored CO_2_ emissions and phosphate impacts when using [bmim][HSO_4_] for separating methanol + *n*-hexane. However, because the systematic use of the LCA methodology for scanning ILs is lacking in the literature, an effort should be made to compile LCA databanks for ILs.

In summary, process simulations could guide the selection of the most adequate extractive properties and processes by simultaneously fine-tuning the IL structure and separation train design. The distribution ratio was the vector for optimizing the energy consumption and utility costs, whereas the required selectivity was a function of the effectiveness of the regeneration train. In fact, the regeneration step was crucial for not only analyzing the energy consumption but also verifying the IL thermal properties, because the MOT of the IL will impact the vacuum consumption, which must be quantified, and even can limit the feasibility of the IL regeneration. In addition, the calculations were only valid for low-viscosity ILs because of the clear limitation of kinetic models in commercial simulators for evaluating the mass-transfer control. In addition to the viscosity, the IL immiscibility in the raffinate was a key point for not only OPEX (IL composition) but also sustainability criteria because the IL losses seemed to be more relevant than the IL toxicity.

### 4.6 Separation Processes by Extractive Distillation

**Scheme 10:**
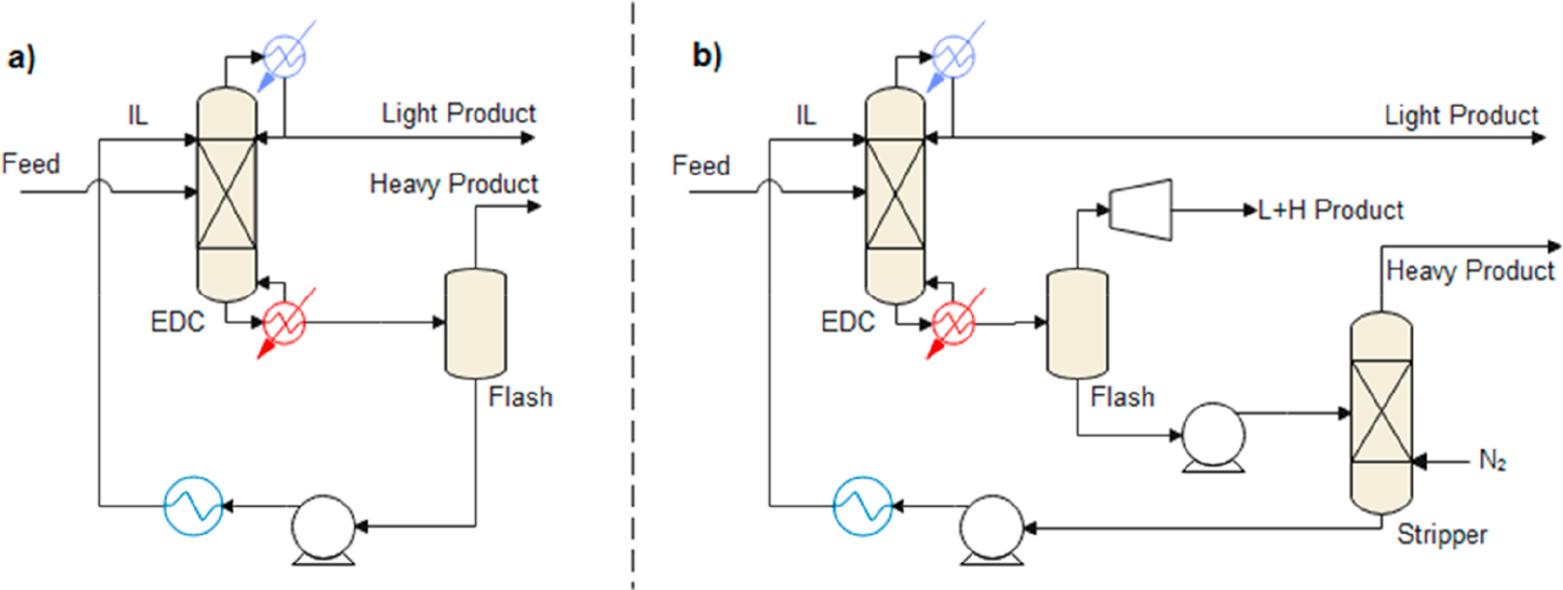
Representative Flow Diagram of IL-Based Extractive Distillation Process

Extractive distillation with ILs is another clear example of their extended use as solvents. owing to their nonvolatility, ILs are ideal for extractive distillation applications. This simplifies the process to residue separation, regenerating the solvent and isolating the key heavy and heavier compounds from the initial mixture. Scheme 10 displays the most representative and simplified flow diagrams. In addition to the main equipment, which is the extractive distillation column, the residue obtained demands the solvent regeneration, which can be accomplished by selecting flash distillation (Scheme 10A) or a flash distillation + stripper (Scheme 10B) as the recommended schemes to minimize the energy consumption. In this Review, process simulations were analyzed based on IL-based homogeneous and heterogeneous extractive distillations.

As in other separation methods, extractive distillation involves the number of ILs available for any desired separation. Table 10 lists the studied systems and ILs to facilitate the understanding of the different approaches through simulation, together with the main variables and key process indicators of each approach. Experimentally determined vapor–liquid or vapor–liquid–liquid equilibria (VLE or VLLE) maximized the light key/heavy key relative volatilities, whether in binary or multicomponent mixtures, and considering both liquid phases in triphasic systems. Process simulations also analyzed the role of the solvent dosage in increasing the relative volatility for enhancing the separation performance. Because the basis of this advanced separation is the same as that for distillation, any specification can be met using valid combinations of the number of stages (N) and reflux ratios (RR) for a given solvent-to-feed ratio (S/F). This aspect is crucial for emphasizing extractive distillation as an efficient separation option because the operation must only achieve the minimum relative volatility in the entire composition range for separation through conventional distillation.^279^

**Table 10:**
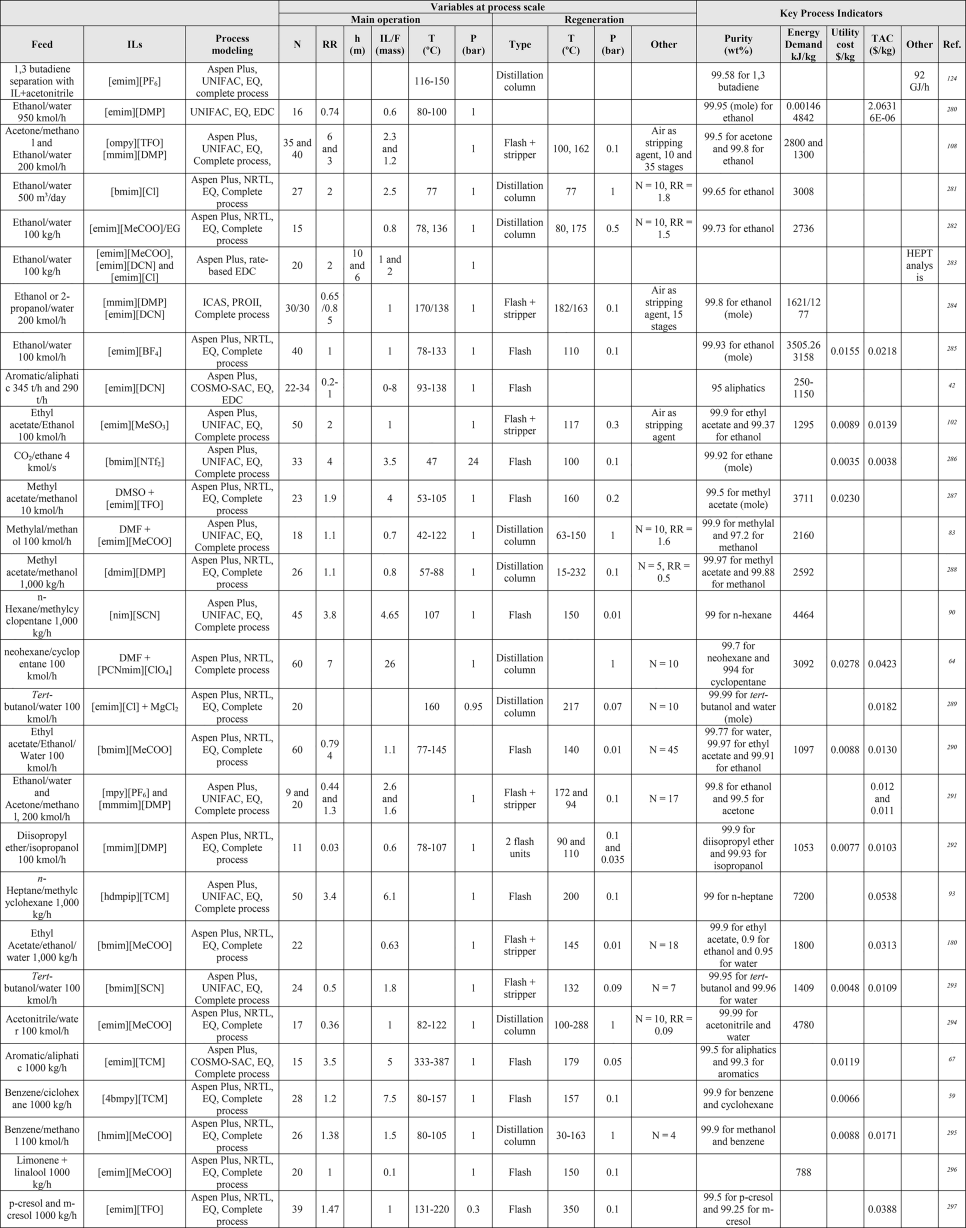

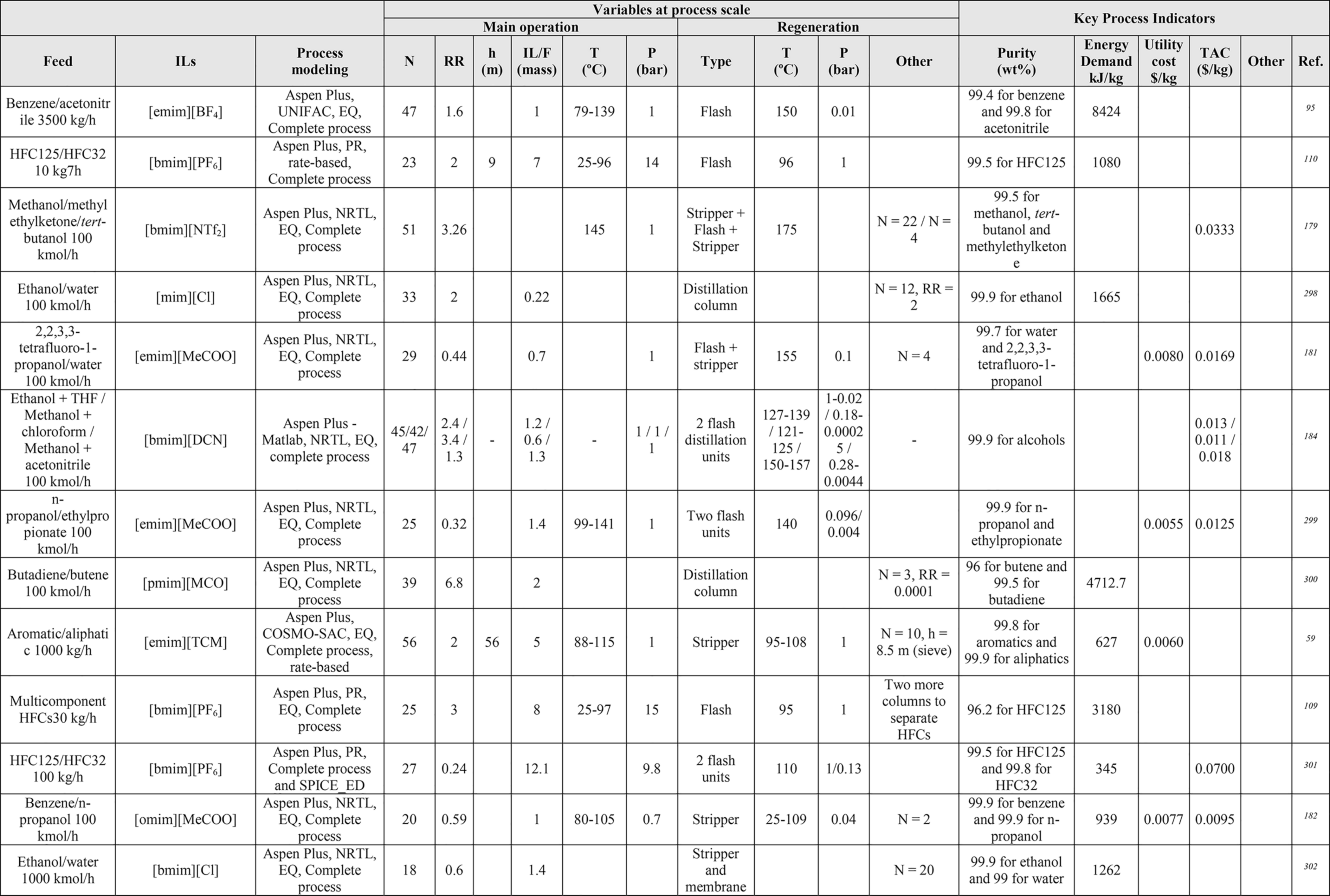
Summary of Variables at Process Scale and KPIs for IL-Based Extractive Distillation Processes

#### 4.6.1 Homogeneous Systems in IL-Based Extractive Distillation

**Figure 51:**
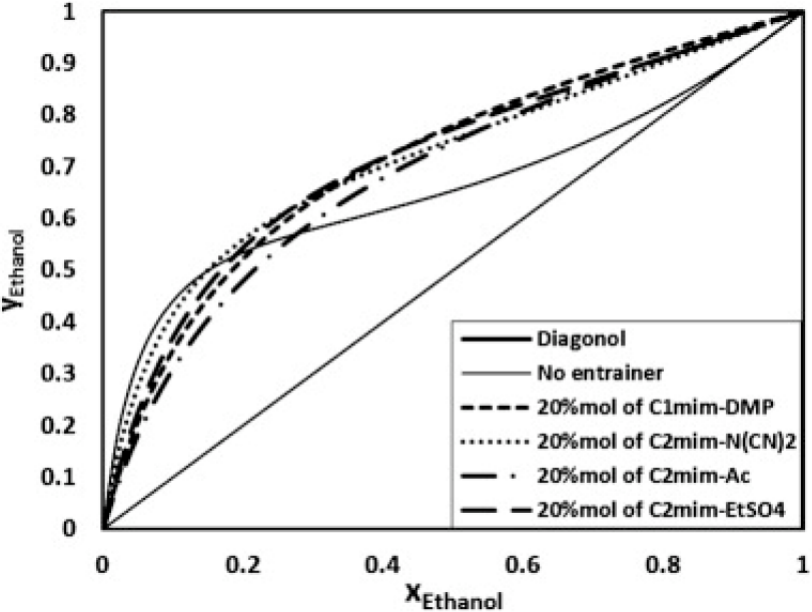
VLE data for ethanol + water in the presence of 20 mol % of each of four selected ILs compared to benchmark binary system (no entrainer) at 1 atm using ICAS-toolbox. Reproduced from ref (284). Copyright 2016 Elsevier.

Most efforts in IL-based homogeneous extractive distillation through the process simulation strategy have been devoted to break azeotropes, mainly in the dehydration of alcohols. The availability of the VLE for several IL + water/alcohol pairs^303,304^ promoted early studies developing this IL-based separation. The modeling of these IL-based systems is easier than others (the VLE of the ternary system, namely alcohol + water + ionic liquid, together with the VLE of the binary mixture of water + ionic liquid); thus, numerous studies that use ILs in this kind of separation are available at the process scale. In fact, systematic screenings of ILs are abundant and cover a wide range of cations and anions. Kulajanpeng et al.^284^ presented a systematic multiscale approach using the UNIFAC predictive model to provide didactic insights about the possibilities or contributions of process simulations in homogeneous extractive distillation. In fact, these authors analyzed the most representative separations, ethanol + water and 2-propanol + water, in a key state-of-the-art study. First, the authors attempted to find ILs that were miscible with the solute, which was water when dehydrating short-chain alcohols. Kulajanpeng et al.^284^ preselected 31 ILs that were completely miscible with water as the first requirement. The criteria could cover not only the miscibility in water but also the relative alcohol/water volatility. The thermal stability, viscosity, and toxicity must also be considered among the other properties proposed in the literature. The authors narrowed the number of ILs to four ([emim][DMP], [emim][DCN], [emim][MeCOO], and [emim][EtSO_4_]), arguing that the [emim] cation controlled the solubility in water, the absence of halide-based anions that could conflict with water,^305^ and the thermal stability. High thermal stability enabled operation at ambient pressure in the extractive distillation column and mitigated the vacuum pressure in the regeneration step. Notably, Kulajanpeng et al.^284^ did not evaluate the relative alcohol/water volatility as a design property but did assess the IL solubility in water and the VLE data of IL-free alcohol/water systems containing <20 mol % of the IL (Figure 51). Clearly, because equivalent IL dosages described equivalent thermodynamic behaviors, the comparison of ILs in extractive distillation must be discussed directly at the process scale because differences in relative volatility are usually inconclusive. A different strategy is the evaluation of the relative volatility together with other key parameters, considering that the solute–solvent interaction determines this relative volatility, as indicated by Quijada-Maldonado et al.^283^ (see Table 11). In these cases, a relative volatility over 1.5 is assumed to be reasonable to define a feasible distillation. Another difference between the Kulajanpeng et al.^284^ and Quijada-Maldonado et al.^283^ approaches is the second key IL property: moving from thermal stability to viscosity, respectively. The goal is to select an IL with a suitable thermal stability and viscosity, together with an effective role in terms of the relative volatility or adequate VLE for separation by distillation. Although both approaches should be complementary to provide a more rigorous methodology, Quijada-Maldonado et al.^283^ also observed that the equilibrium rather than the kinetics controlled the separation owing to higher viscosities. A common trend is clearly presented in the literature: compared with acetate or chloride-based ILs, dicyanamide-based ILs similarly impact the relative volatility but enabled the use of a lower-viscosity IL that compensated at the process scale. The IL toxicity is another property that can be evaluated as a criterion for preselecting ILs because in this kind of separation, the IL consumption is on the same order and because toxicity can be of interest for improving the process sustainability.^277,294^

**Table 11:** Comparison of Key Features of Mass Agents Evaluated for Separating Ethanol and Water before Process Simulation^283^

	α		η (mPa·s)	
Solvent	S/F = 1	S/F = 2	T = 25 °C	T = 80 °C
[emim][Cl]	2.62	3.98	2,597.69	65.18
[emim][MeCOO]	2.24	2.92	132.91	13.60
[emim][DCN]	1.89	2.58	14.90	4.66
Ethylene glycol	1.83	2.41	16.61	3.14

Once the ILs have been preselected, the sequence suggested by Kulajanpeng et al.^284^ continues as follows: a) validation of the model; b) suitability of the IL in terms of the main separation; c) feasibility of the ILs to be regenerated and reused in a continuous process; d) optimization of the process for each IL; e) comparison of energy consumption or costs (operating or TAC) as function of each IL. The evaluation of the S/F ratio on a mass basis is important when comparing the IL effectiveness; otherwise, the comparison can be affected by the well-known wide range of molecular weights. Effectively, the four solvents selected by Kulajanpeng et al.^284^ were compared within the same equipment designs by adapting the S/F ratio to meet the specifications. Table 12 compares the data collected for the four IL candidates and the corresponding key process indicators. In contrast, other examples for which the change was not related to the IL variation but to the process configuration or a combination of both are available in the literature.^109,110,289^

**Table 12:** Design and Separation Specifications and Key Process Indicators of Four Selected ILs Preselected by Kulajanpeng et al.^284^ for Separating Ethanol from Water

IL	[emim][DMP]	[emim][DCN]	[emim][EtSO_4_]	[emim][MeCOO]
EDC				
N	30			
N_IL_	2	2	2	2
N_feed_	23	22	23	23
IL purity(mol %)	>99.8			
IL (kmol/h)	53.48	120.00	85.00	108.70
P (bar)	1			
RR	0.65	0.62	0.85	0.99
T_reboiler_ (°C)	170	170	175	152
Reboiler duty (kW)	3,160	3,300	3,440	3,620
Flash				
P (bar)	10	10	10	–
T (°C)	182	200	251	–
Duty (kW)	470	620	750	–
Stripper				
N	15	10	10	–
N_feed_	1	1	1	–
P (bar)	1	1	1	–
T_bottom_ (°C)	78	78	78	–
Air (kg/h)	2,643	8,071	3,798	–
Overall heat duty (kW)	3,640	3,920	4,200	–

Kulajanpeng et al.^284^ analyzed the main variables, namely S/F, N, and RR, of the extractive distillation column. The authors set N and RR, whereas S/F was varied to adjust the IL features to the specifications. This approach is essential to have equivalent equipment to focus the analysis on the energy demand. A subsequent optimization can be conducted after selecting the optimal IL. Numerous possibilities are available, indicating the flexibility of process simulations as follows: (i) fix N and RR and liberate S/F,^284^ (ii) fix N and S/F and liberate RR,^102,110^ and (iii) evaluate several scenarios by changing together for N, S/F, and RR.^102,282^ The estimation of the CAPEX is more complex and less robust than that of the energy consumption and, thus, OPEX. Options (i) and (ii) are similar but depend on the features of the preselected ILs. For small differences and highly effective ILs, both approaches are valid; however, for difficult separations and large differences between ILs, fixing S/F and N can introduce problems to the extractive distillation column related to very high RR values, which can worsen the separation when the relative volatility is low at the selected S/F ratio.^59^ The most conservative approach is option (iii) because it enabled a suitable operation to be found for all the ILs.

**Figure 52:**
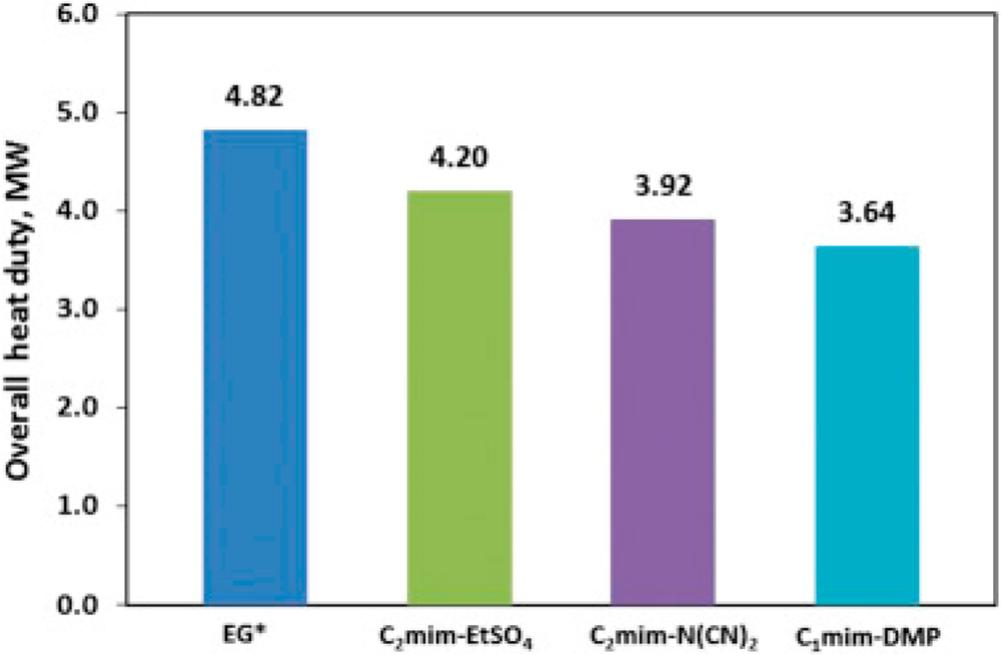
Evaluation of four ILs in terms of energy consumption for heating for set of specifications and equivalent equipment size detailed in Table 12 for ILs and for ethylene glycol (EG). Reproduced from ref (284). Copyright 2016 Elsevier.

The heating duty was the key decision in the study of Kulajanpeng et al.,^284^ and the results were compared against ethylene glycol as the benchmark solvent. The latter demands 4.82 MW to satisfy the specifications compared with the best IL-based approach, which demands 3.64 MW (see Figure 52). The main contribution of process simulation was the comparison of novel solvents with benchmark ones under realistic conditions. As listed in Table 10, kilojoules per kilogram should be used as the basis for comparing the energy demands of processes; however, because the feed and separation target are the same in this case, the specific energy consumption shows the same picture: 2,472 kJ/kg for ethylene glycol and 1,621 kJ/kg for [emim][DMP]. By reviewing all the studies, the sensitivity analyses include, in most cases, the energy consumption and costs.

**Figure 53:**
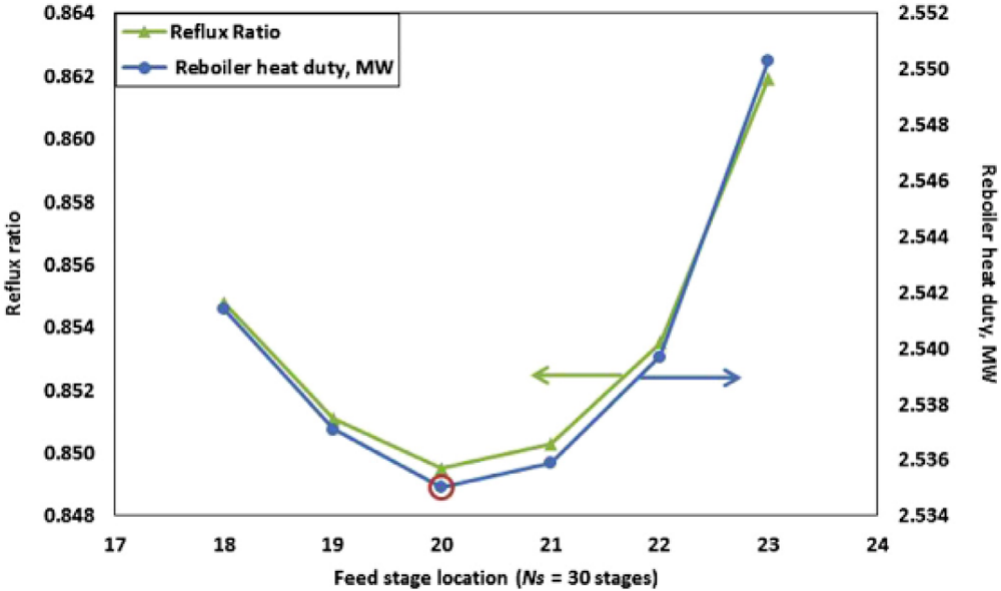
Reboiler duty dependence on feed stage and RR simultaneously evaluated for [emim][DCN] at N = 30 and 1 bar in EDC. Reproduced from ref (284). Copyright 2016 Elsevier.

Kulajanpeng et al.^284^ also evaluated RR in the short term, which enabled the minimum energy consumption to be determined for assessing the purity criterion when changing the feed stage (see Figure 53). Extractive distillation approaches for homogeneous systems range between low S/F ratios (on a mass basis) and RR, both of which are usually approximately 1, and relatively high values of N, approximately from 20 to 50.

**Figure 54:**
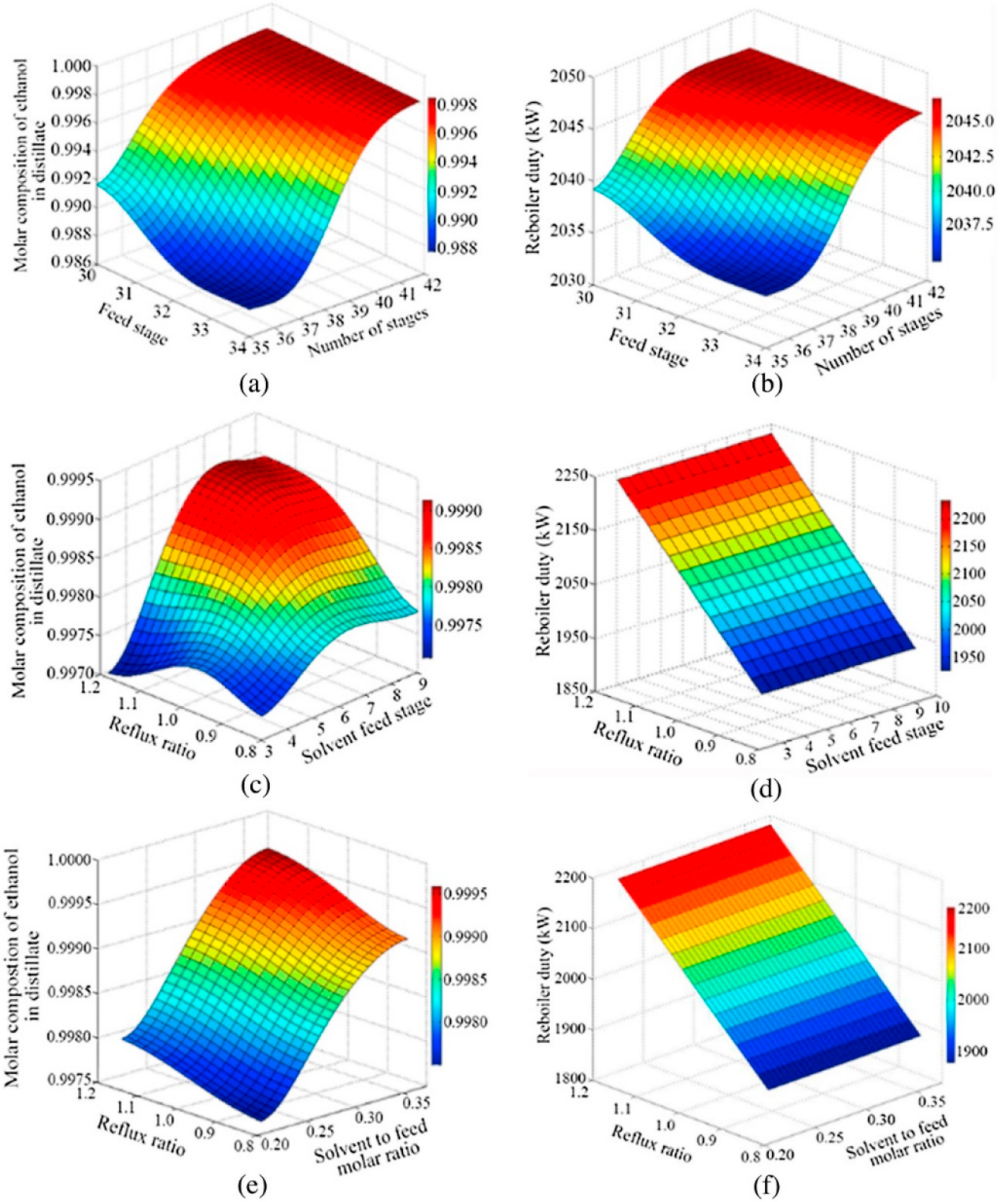
Surface diagrams for monitoring ethanol purities and reboiler duties versus RR, S/F, feed stage, solvent feed stage, and N during dehydration of 100 kmol/h feed comprising mixture of 85% pure alcohol (on molar basis) at 1 bar. Reproduced from ref (285). Copyright 2016 Elsevier.

For extended examples in suitable ranges, Figure 54 shows an example by Dai et al.^282^ for properly evaluating the obtained separation (purity of the compound collected in the distillate) and energy consumption. The variables are S/F, RR, N, feed stage, and solvent feed stage. Dai et al.^282^ showed that the S/F ratio impacted more than RR in the energy consumption and that it controlled the separation, which could be of interest in the optimization step. Another clear variable is the column pressure, which must be related to the use of cooling water in the condenser and, thus, to the compounds being separated. Condenser temperatures below 25 °C require more expensive refrigerants, such as chilled water or even propane. The vacuum pressure, on the other hand, must be considered as a key utility cost. In most cases, the pressure is fixed near atmospheric pressure (approximately 1 bar); however, the separation of refrigerants requires higher pressures owing to the high volatility of the solutes.^109,110^

Aside from the study of the extractive distillation column’s key variables, Kulajanpeng et al.^284^ completed the flowsheet with a previously optimized configuration scheme comprising a flash distillation unit and a stripping column for solvent regeneration, reducing the flow in the stripping column by exploiting the previous flash distillation.^306^ In other studies, a second distillation column,^124,281,282^ a flash distillation unit,^42,285,286^ a stripping column,^182^ two flash units,^292,301^ or flash + stripping column + flash^179^ were the most extended separation trains used to complete the process. The separation train must be optimized based on the TAC or utility consumption/cost. The separation train barely affects the process specifications because the extractive distillation column is the key to assess recovery of light compounds and purity of heavy compounds. The separation train only affects the regeneration expenses and the global separation when the purity of the recycled solvent is low. Grazcova et al.^287^ used process simulations to change the regeneration scheme and observed that IL-based processes could be better or worse than conventional ones, depending on the separation train. This should be evaluated in future studies for providing the best IL technology and fairly comparing it against other non-IL-based processes. Novel schemes are emerging for evaluating IL-based extractive distillation processes. Martinez et al.^302^ used membrane-based configurations in the separation train to control the energy consumption, whereas Aniya et al.^289^ evaluated separation schemes integrated and even thermally coupled with columns (extractive distillation and regeneration columns) with excellent results.

Another option that can be tuned at the process scale is the composition of a hybrid solvent to modulate key process indicators by changing the composition of the mixture, as stated in Section 4.5. For homogeneous extractive distillation, some studies involving a hybrid solvent: (i) [emim][MeCOO] + EG used in the separation of ethanol and water for simultaneously reducing the energy consumption and vacuum requirements;^282^ (ii) [emim][MeSO_3_] + DMSO in the separation of methyl acetate and methanol, achieving the best results with neat IL;^287^ (iii) [emim][MeCOO] + DMF in the methanol/methylal separation;^83^ (iv) [PCNmim][ClO_4_] + DMF in the neohexane/cyclopentane separation.^64^

**Figure 55:**
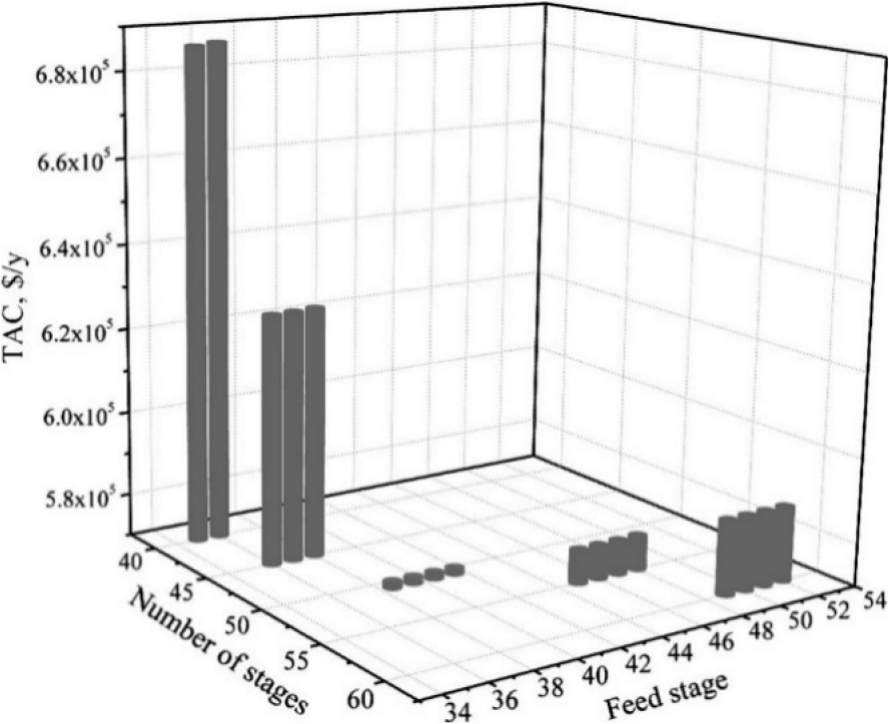
TAC versus N and feed stage to obtain 99.9% pure ethyl acetate, on molar basis, at 1 bar and considering feed flow of 100 kmol/h and equimolar compositions of ethanol and ethyl acetate. Reproduced from ref (102). Copyright 2017 Elsevier.

The optimization approach should focus on the energy consumption or cost (OPEX, CAPEX, or TAC). Some studies have reduced the energy consumption as the target,^83,108^ whereas others have focused on the TAC objective function exclusively.^179,297^ In addition, in many studies, the TAC was used as an optimization criterion with energy consumption having a relevant role in the analysis.^64,102,290^ In particular, Zhu et al.^102^ showed another option for addressing the TAC in the configuration of the main column by clearly defining the optimal number of stages and which are the possibilities to define the feed stage (Figure 55).

**Figure 56:**
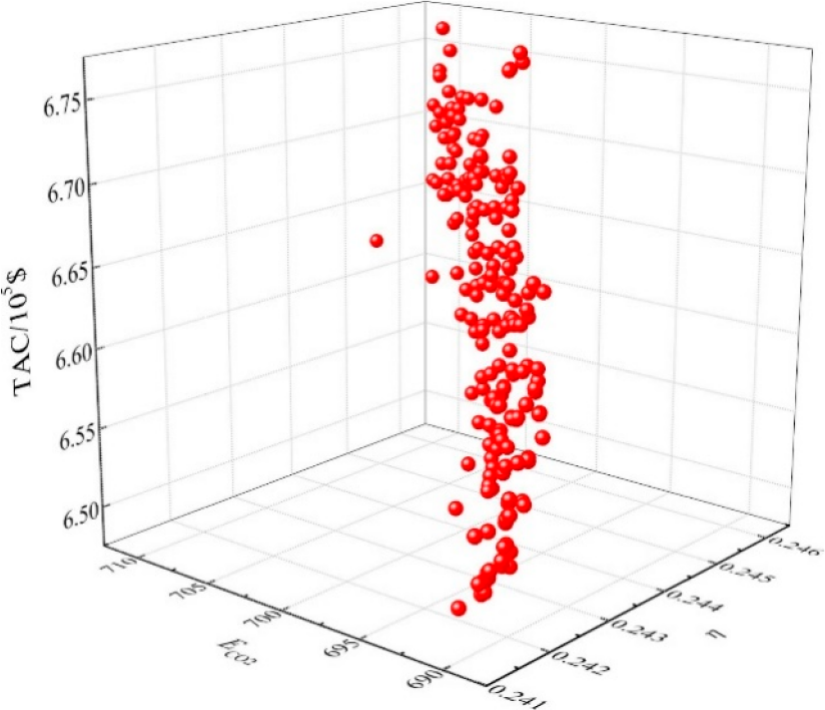
Scattering 3D diagram relating TAC, CO_2_ emissions (E_CO2_), and thermodynamic efficiency (η) for separating ethyl acetate–ethanol–water at 100 kPa and considering 100 kmol/h of feed containing molar compositions of 55.58, 18.52, and 27.90% for ethyl acetate, methanol, and water, respectively, by fixing ethyl acetate purity above 0.9995 (on molar basis) and varying S/F and RR. Reproduced from ref (290). Copyright 2019 Elsevier.

Presently, the criteria involving the costs and energy are reformulated under the sustainability criteria. Regarding LCA or CO_2_ emissions, few studies have evaluated them together with process simulations. Energy-consumption-related CO_2_ emissions can be monitored in the Aspen Plus process simulator. In fact, the evaluation of TAC versus CO_2_ emissions is interesting for analyzing not only energy expenses but also the impact of the energy on the GWP, as previously evaluated by Ma et al.^290^ (Figure 56). Additionally, Ma et al.^290^ monitored the thermodynamic efficiency to measure the work lost in the process, which aligns with the exergetic analysis and can help to select some configurations with equivalent TACs, energy consumption, and/or CO_2_ emissions. This approach leads to new criteria for the use of process simulations to enhance extractive distillation designs together with sustainable criteria. However, although these efforts are interesting, the challenge is advancing the use of LCA or equivalent methodologies as tools in an eco-design approach when selecting the key process variables.

Li et al.^184^ showed a comprehensive optimization-control approach for some examples of extractive distillation processes based on ILs. First, three objective functions are evaluated for three systems, namely ethanol + THF, methanol + chloroform, and methanol + acetonitrile, ranging from the TAC perspective to other formulations in which environmental issues are included. Besides, the authors analyzed the stability of the processes through the evaluation of proposed control of the dynamic simulation. The discussion of the authors is of high value since they presented how RR and column diameter condition TAC and control stability divergently. Control is easier using higher column diameters and RR, due to higher hold ups and flows, respectively; however, this impacts TAC values negatively. Therefore, dynamic simulation can help to rationalize TAC optimization to values in which the control is feasible for industrial implementation.

To the best of our knowledge, extractive distillation with ILs is the only representative process in which control is evaluated through process simulation. Peng et al.^307^ presented pioneer results in a distillation column in which the IL was flowing, within a reactive distillation process, following conventional control design. More recently and focusing on improving the control design, Ma et al.^183^ and Pan et al.^308^ showed a similar approach as that adopted by Li et al.,^294^ highlighting that the control of temperature in the top and bottom of the EDC is more stable for systems in which ILs act as entrainers. Wei et al.^181^ directly adopted the same strategy with success. The contribution of process simulation in the control of processes based on ILs is clear here, being against heuristics to control distillation columns.

**Figure 57:**
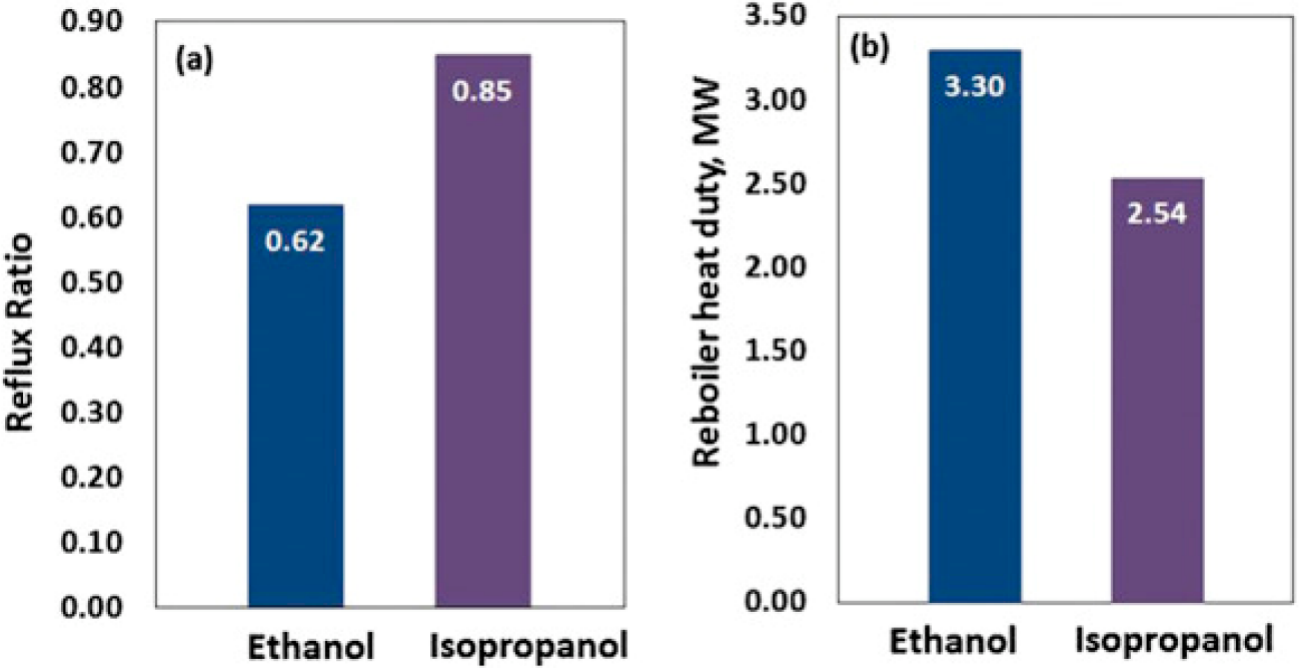
Changes in RR and in reboiler energy consumption when transferring behavior from ethanol/water to 2-propanol/water separation to meet equivalent specifications (N = 30, N_feed_ = 22, and 1 bar in EDC to achieve alcohol purity of 99.8% on molar basis). Reproduced from ref (284). Copyright 2016 Elsevier.

As a final contribution, and in line with the wide range of evaluated applications and available in the literature, Kulajanpeng et al.^284^ suggested the transferability from the designed process (ethanol + water) to others with common features (2-propanol + water). According to this concept, the use of process simulation is recommended to extend the knowledge in a series of systems, drastically reducing time and resources after the first findings. Nevertheless, an interesting picture (see Figure 57) emerges from Kulajanpeng et al.,^284^ who advise that changing the system can lead to a higher RR but a high reduction in energy consumption, which enables interesting conclusions to be drawn related to the changing behavior of a process from a product design perspective.

Once the contributions of process simulations to the development of this technology have been reviewed, the following briefly describes the most studied systems at the process scale with homogeneous extractive distillation together with the recommended ILs and S/F ratios. The binary azeotropic mixtures integrated with water and another compound, namely ethanol,^108,280−285,291,298,302^ 2-propanol,^284^ 2,2,3,3-tetrafluoro-1-propanol,^181^*tert*-butanol,^289,293,294^ and acetonitrile, have been extensively studied at the process scale, whereas the ternary azeotropic system comprising water + ethanol + ethyl acetate,^180,290^ is well described at the process scale but less so, with S/F ratios (on mass basis) approximately ranging from 0.3 to 2, with highlighted ILs as imidazolium-cation-based ones completed with short alkyl substituents and anions comprising dicyanamide, dimethylphosphate, and halides. Regarding binary azeotropic mixtures of alcohols and other polar compounds, several examples are available in the literature, including methanol with methyl ethyl ketone,^179^ methyl acetate,^287,288^ acetone,^108,291^ and methylal;^83^ ethanol with ethyl acetate;^102^*n*-propanol with ethyl propionate;^299^ and 2-propanol with diisopropyl ether,^292^ with S/F ratios (on mass basis) from 1 to 2 in almost all cases, leaving aside 0.03 for 2-propanol and diisopropyl ether,^292^ highlighting ILs based on dimethylphosphate and acetate anions completed with imidazolium cations having short alkyl groups. Another interesting family of binary azeotropic mixtures is that regarding benzene and polar compounds, such as methanol,^295^ 2-propanol,^182^ and acetonitrile,^95^ showing S/F ratios in mass in a narrow window (from 0.6 to 1.6) and ILs with imidazolium cations and acetate or tetrafluoroborate anions. The complete number of systems is completed with p-cresol/m-cresol separation,^297^ butadiene separation from acetonitrile^95^ or butene,^300^ and limonene/linalool^296^ separation. Finally, the separation of several mixtures of refrigerants have been simulated with extractive distillation with ILs,^109,110,301^ and the CO_2_/ethane separation.^286^

#### 4.6.2 Heterogeneous Systems in IL-Based Extractive Distillation

Compared with liquid–liquid extraction, extractive distillation operates based on one key property (relative volatility) instead of two (distribution ratio and selectivity). For homogeneous systems (describing VLE), the correlation of relative volatility is an independent function of selectivity from equivalent liquid–liquid extraction; however, in heterogeneous systems (showing totally or partially VLLE), relative volatility is correlated with the two extractive properties (selectivity and distribution ratio) from equivalent liquid–liquid extraction, respectively. Therefore, heterogeneous extractive distillation operates similarly to liquid–liquid extraction but with the advantage of the RR to achieve high purities in the main operation. Operation with only one liquid phase is relevant for systems that have partially heterogeneous regions, i.e., two liquid phases, because this will improve mass transfer in the column.

**Figure 58:**
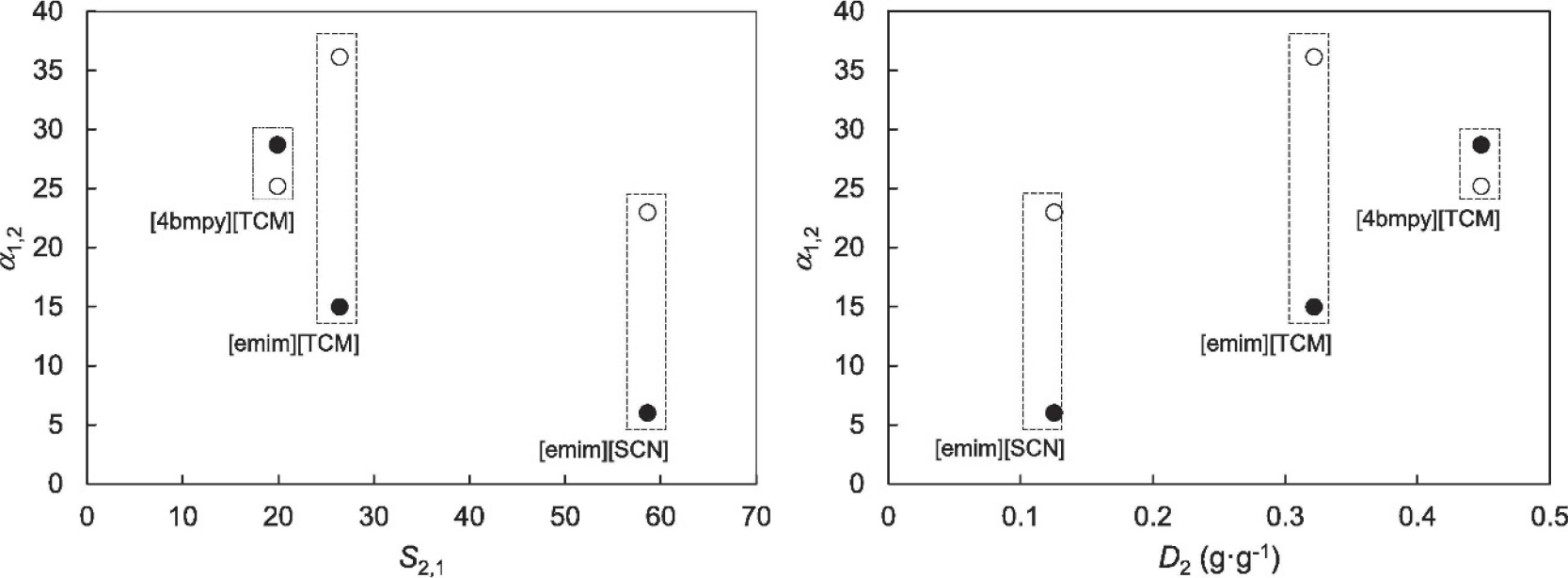
Relative volatility of *n*-heptane from toluene (α_1,2_) at 50 °C (full symbols) and 90 °C (empty symbols) and S/F ratio of 10 vs. toluene/*n*-heptane selectivities (*S*_2,1_) and toluene distribution ratios (*D*_2_) at 40 °C with the same hydrocarbon feed. Reproduced from ref (261). Copyright 2018 Elsevier.

**Figure 59:**
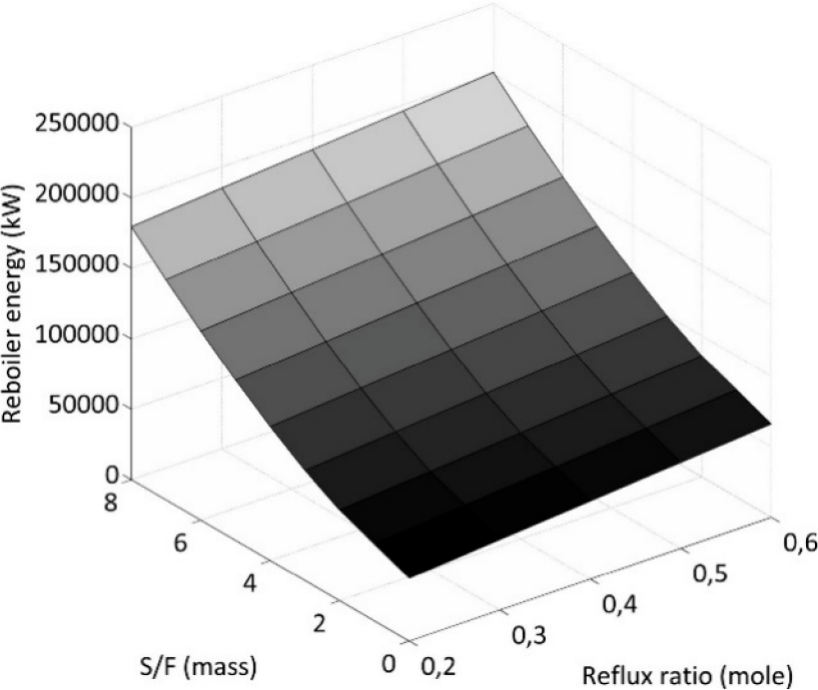
Reboiler duty as a function of S/F and RR to separate aromatics from aliphatics considering N = 34 and N_feed_ = 25. Reproduced from ref (42). Copyright 2016 Elsevier.

Navarro et al.^261^ tried to correlate extractive properties (toluene distribution ratio and toluene/*n*-heptane selectivity) with the *n*-heptane/toluene relative volatility for a representative number of ILs (ranging from those with high toluene distribution ratios and low toluene/*n*-heptane selectivities to those with the inverse properties), to transfer the liquid–liquid extraction knowledge from the aromatic/aliphatic separation to extractive distillation. As shown in Figure 58, there is a transition from VLLE to VLE when increasing the temperature or the S/F ratio, thus moving from toluene distribution ratio control within VLLE to a linear *n*-heptane/toluene relative volatility dependence on toluene/*n*-heptane selectivity in VLE. This fundamental knowledge enabled the design of an extractive distillation column that can operate without two liquid phases by preheating the feed and moderate-high S/F ratios, as Navarro et al.^67^ developed for [emim][TCM] and a multicomponent model of pyrolysis gasoline (see Section 4.5 for details). The complexity of these systems demands a multiscale development in which experimental evidence and computational tasks must be effectively combined. In fact, Diaz et al.^42^ proposed this approach for the first time using [emim][DCN] as the solvent. Diaz et al.^42^ used the COSMO-based/Aspen methodology and screened N, feed stage, RR, and S/F impacts. The contribution of this work was demonstrating the crosseffects of these variables on both aromatic separations and energy consumption, the latter of which is shown in Figure 59. However, the separation results were hindered because the feed and the solvent were introduced in the extractive distillation column at 25 °C, and the tested range of S/F ratios only covered low values.

**Figure 60:**
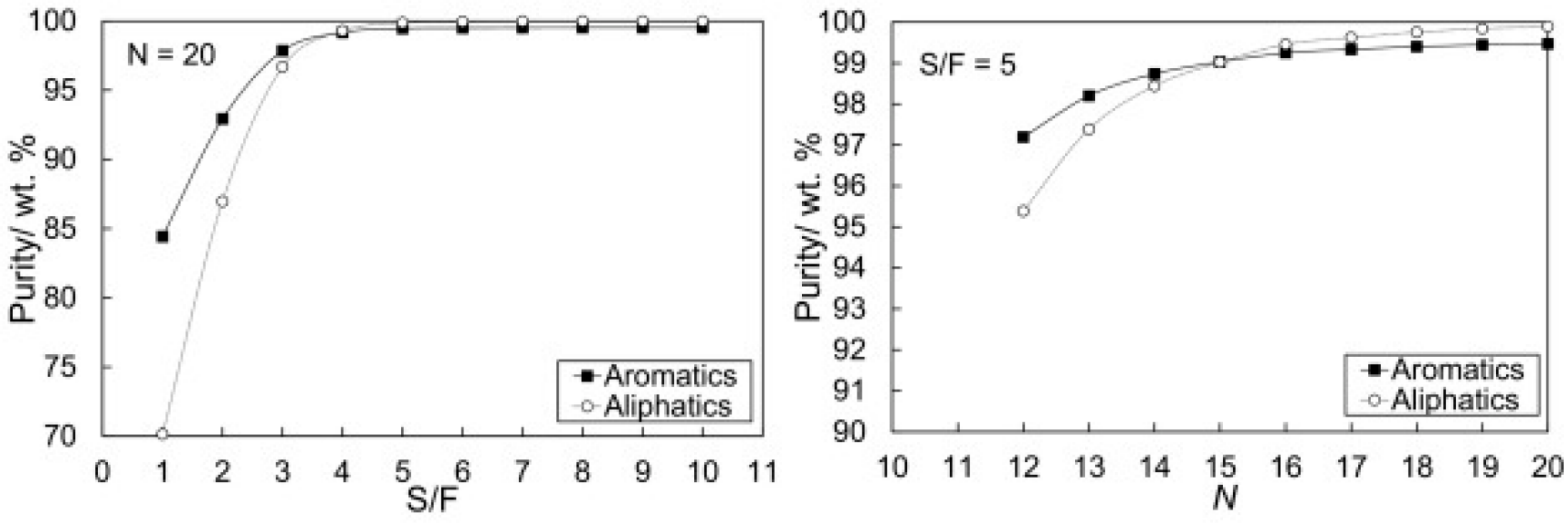
Evolution of purity of aromatics and aliphatics as a function of S/F (left) and N (right) to separate aromatics from aliphatics from pyrolysis gasoline model (see liquid–liquid extraction) at 1 bar and fixing RR as 1.3 times minimum. Reproduced from ref (67). Copyright 2019 Elsevier.

Navarro et al.^67^ used the FUGK (Fenske, Underwood, Gilliland, and Kirkbride) shortcut method to narrow N and RR for the extractive distillation column. Afterward, S/F and N were evaluated up to improved values of S/F = 5 and N = 15 (Figure 60), taking advantage of the well-known coupling of energy consumption and operating S/F ratio and fixing RR to that related to 1.4 times greater than the minimum. A favorable energy consumption of the extractive distillation process was found compared with an equivalent liquid–liquid extraction process. These results clearly show that process simulation can deal with complex problems, as heterogeneous systems in extractive distillation, and enabled the testing of new approaches as preheating the feed streams to enable a distillation process without mass transfer limitation associated with the presence of the two liquid phases in the operation.

Relevantly, the three allowed phases must be specified in the Aspen Plus process simulator. However, when two liquid phases are allowed and only one liquid phase is obtained, vapor–liquid specifications should be used instead of vapor–liquid–liquid specifications because the former are the only option that enables rate-based calculations. In summary, this is a fantastic example in which multiscale approaches are the most powerful designing routes.

Screening ILs properties is a relevant first step. In this case, instead of the preselection approach conducted for homogeneous systems, a full database screening at the process scale is introduced in the study by Ayuso et al.^59^ There are advantages and disadvantages with both approaches. The clear advantages for preselecting ILs before process simulations are that the simulated matrix is more easily handled and that the process-scale operation is more straightforward. On the contrary, relevant disadvantages are related to the consistency of the selection criteria at the process scale and more experimental features are required for a wide range of ILs; for instance, the selection of an IL based on the relative volatility may be not consistent at the process scale owing to the cross-effects of this variable and the solvent volatility, thermal stability, viscosity, etc. on the separation and energy consumption.

Either by preselecting ILs or screening an entire database, the first step is characterizing the extractive distillation column. Here, the details of the extractive distillation column are displayed as contribution of process simulation in the massive evaluation of ILs.

Ayuso et al.^59^ systematically studied the suitability of extractive distillation to separate BTX from a pyrolysis gasoline by screening the ILUAM database^51^ and covered all the representative partial studies to correlate solvent properties to extractive distillation key process indicators and overall process impacts.

**Figure 61:**
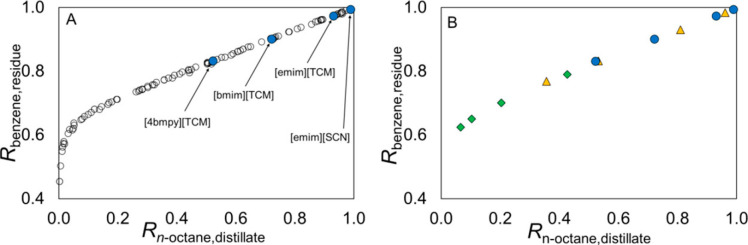
Benzene recovery in residue plotted as a function of IL and S/F ratio at 1 bar. (A) Screening of ILs in EDC. Empty circles denote ILUAM database a S/F ratio of 5, whereas full circles denote selected ionic liquids at same S/F ratio. (B) Triangles and diamonds represent S/F ratios of 3 and 1, respectively, for selected ILs, and full circles represent same points as those in panel A. Reproduced from ref (59). Copyright 2022 ACS.

Selection is preliminary made in terms of recoveries of light and heavy keys in distillate and residue, respectively, namely *n*-octane and benzene. Because both recoveries operate proportionally and similarly only one can be monitored. Figure 61 shows the ILUAM database screening on this separation, evidencing two big issues in the two subfigures: A) The IL role is essential to modulate the separation, governing the specific interaction between benzene and the IL (transferring extractive properties from liquid–liquid extraction approach, the selectivity is the key) and B) the S/F ratio can modulate recoveries for the same IL. In fact, the IL and S/F must be properly combined to enhance the separation parameters to values at which the separation is facilitated (highly efficient solvents and high S/F ratios).

The approach should be analyzed as follows for future studies: (i) for well-known separations with high availability of experimental data, it is recommended to go at the process scale with a reduce number of well-selected solvents; (ii) for novel separations or approaches, operation at the process scale with the entire database and the use of prediction models could be better.

**Figure 62:**
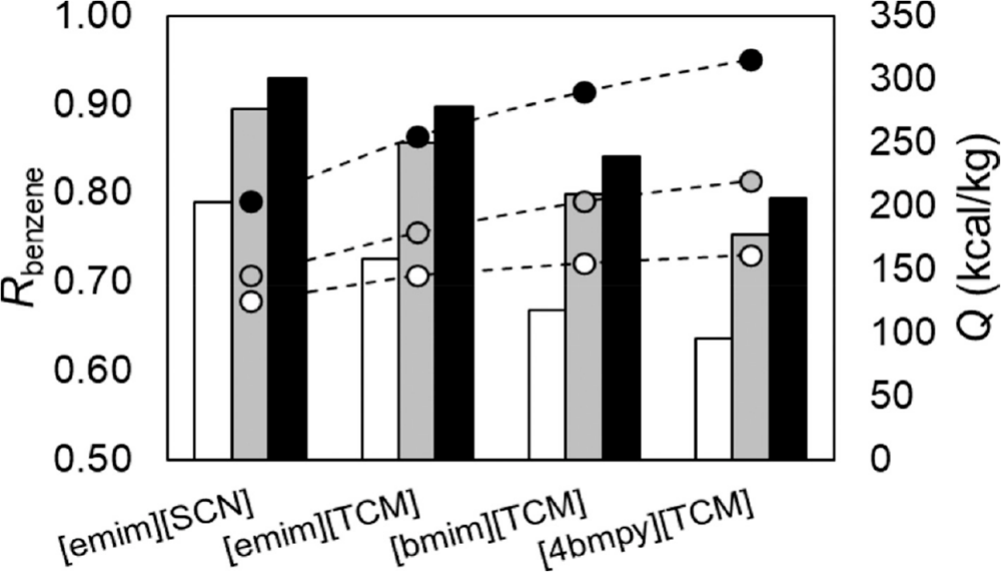
Benzene recovery in residue plotted as a function of IL and S/F ratio at 1 bar and N = 20, monitoring energy consumption. S/F = 1 white, 3 gray, and 5 black. Reproduced from ref (59). Copyright 2022 ACS.

Figure 62 shows, for the selected ILs, the fact that the highest recoveries and the lowest energy consumption are obtained with the most selective IL, as expected under operating conditions that promote the use of a column with only one liquid phase.

**Figure 63:**
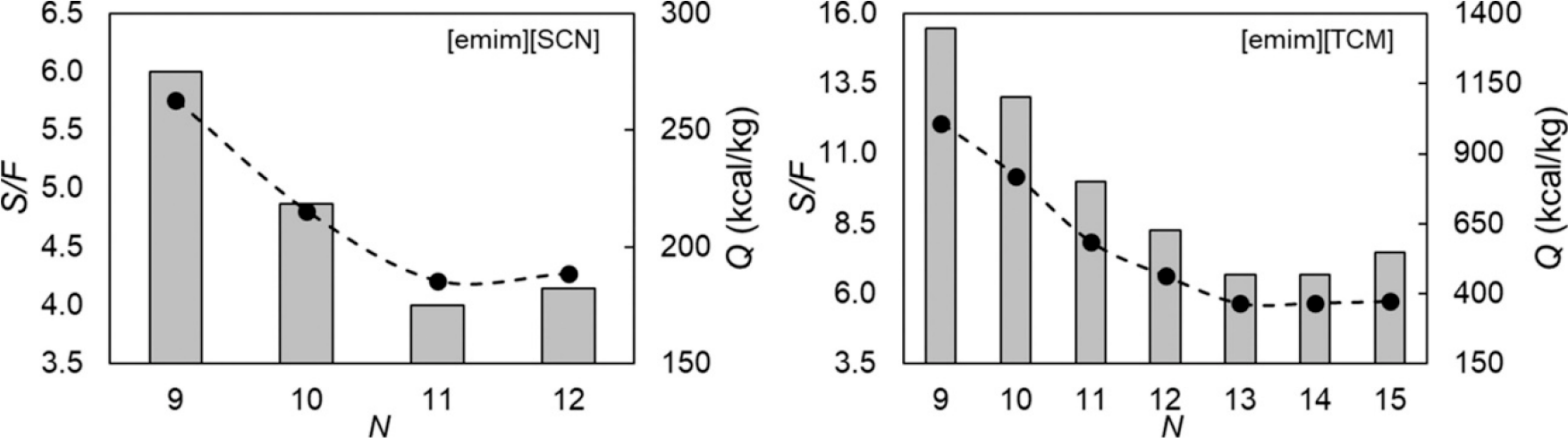
Minimized heat flow in reboiler plotted as functions of N–S/F pairs for RR = 2 and at 1 bar for recovering 99% of aliphatics (distillate) and aromatics (residue) feeding hydrocarbons and IL in 6th and 2nd stages, respectively, and evaluating [emim][SCN] and [emim][TCM] ILs. Reproduced from ref (59). Copyright 2022 ACS.

As shown in Figure 63, at commercial specifications, [emim][SCN] clearly consumed half the energy consumed by [emim][TCM], after optimizing the N–RR pair versus energy consumption for the same specification. The main problem with [emim][SCN] is the low thermal stability, whereas [emim][TCM] was selected as a strong solvent in terms of separation and properties (viscosity and thermal stability). Here, the ad hoc specification of thermal stability is highlighted as key at the process scale. Discretizing the thermal stability limitations shifts the focus from selecting the best candidate in terms of thermodynamic behavior to that with more compensatory features (extractive and thermophysical).

**Figure 64:**
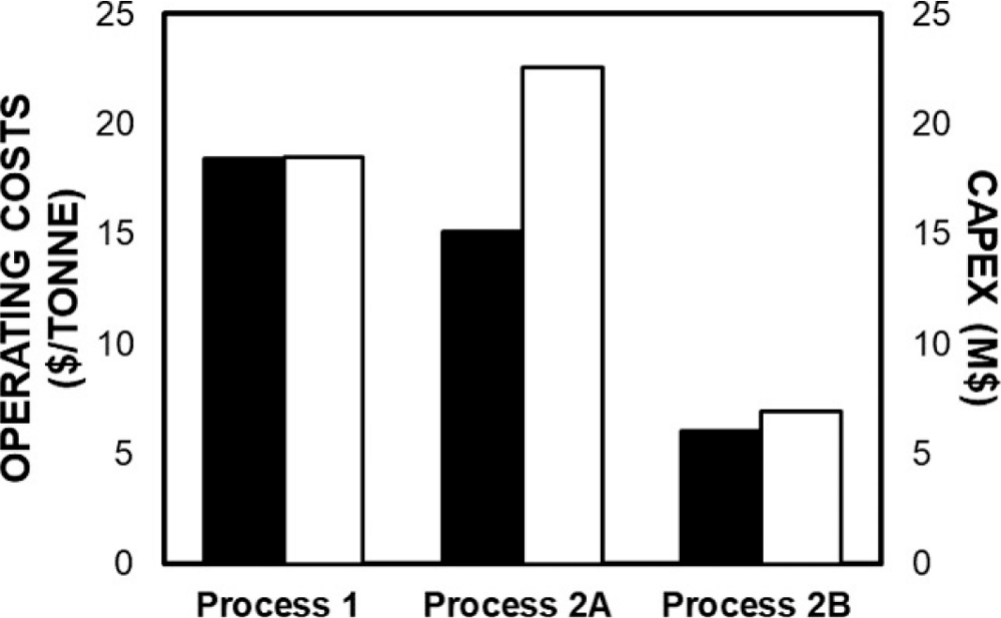
OPEX and CAPEX calculated for Process 1, Process 2A, and Process 2B to purify the aromatics >99 wt % with NFM (Process 1) and [emim][TCM] (Processes 2A and 2B). Reproduced from ref (59). Copyright 2022 ACS.

Another relevant point is that Figures 61–63 depict simulations in equilibrium, without considering the mass-transfer limitation. However, the flipping phenomenon related to the benzene/*n-*octane relative volatility is very successful, because the commercial process (Morphylane) cannot support the direct separation of the C6–C8 cut. The Morphylane process (named Process 1 by Ayuso et al.^59^) is compared with two configurations: the same scheme with the IL substituting *N*-formylmorpholine (Process 2A) and a new process integrating the two columns to separate BTX from C6–C8 aliphatics in the extractive distillation unit (Process 2B). Ayuso et al.,^59^ performed rate-based rigorous modeling with TAC as decision parameter to select the best process As shown in Figure 64, Process 2A using [emim][TCM] decreased OPEX, but slightly increased CAPEX compared with Process 1. However, when the process is designed exclusively attending to the properties of the IL (Process 2B) the impact of the IL is highly relevant, decreasing both OPEX and CAPEX from the commercial case. Therefore, process simulation has enhanced the role of the IL in the dearomatization of refinery streams, apart from the management of a multicomponent stream in a complex process, opening a new and improved process. Again, coupling the design of the process to the IL leads to a more competitive IL-based extractive distillation technology.

Other examples of heterogeneous systems in extractive distillation with ILs that were evaluated via process simulation are described as follows: the separation of alkanes (*n*-hexane, neohexane, and *n*-heptane) from cycloalkanes or alkylcycloalkanes and separation of benzene/cyclohexane using cyano-containing anion-based ILs or hybrid entrainers.^59,64,90,93^ In fact, only one hybrid solvent is found as adequate IL-based entrainer (DMF + [PCNmim][ClO_4_]) in the cyclopentane and neohexane separation that is not integrated by CN groups.^64^ In all cases, S/F ratios in mass basis are considerably higher than those in homogeneous systems, ranging from 3 to 7, whereas CN-containing anions are the most recommended ILs.

Diaz et al.^277^ pointed that toxicity can be a key property to preselect environmentally friendly ILs. However, no LCA developments are available for IL-based extractive distillation processes whether they are heterogeneous or homogeneous systems. This could be related to the reduced number of ILs available to deploy LCA studies.

In summary, process simulation can guide the selection of the most adequate ILs and operating conditions of extractive distillation process. Extractive distillation for homogeneous systems imposed an easier scenario because only selective interactions are demanded, whereas heterogeneous systems are more complex because they demanded not only specific interactions with the compound to be retained in the liquid but miscibilityin the liquid; however, preheating the feed simplified the task as the main contribution of process simulation in the treatment of heterogeneous cases. The S/F ratio must be the minimum to make the mixture distillable, showing much greater values in heterogeneous systems. In fact, S/F and RR of the EDC are the key variables to control energy consumption and cost, whereas the regeneration is secondary, in contrast with liquid–liquid extraction processes. Viscosity is less demanding in this operation than in others, owing to the higher temperatures at which it operates, and IL losses can be considered as negligible because all separations are distillation based and ILs are nonvolatile compounds.

### 4.7 Absorption Refrigeration Cycles

**Scheme 11:**
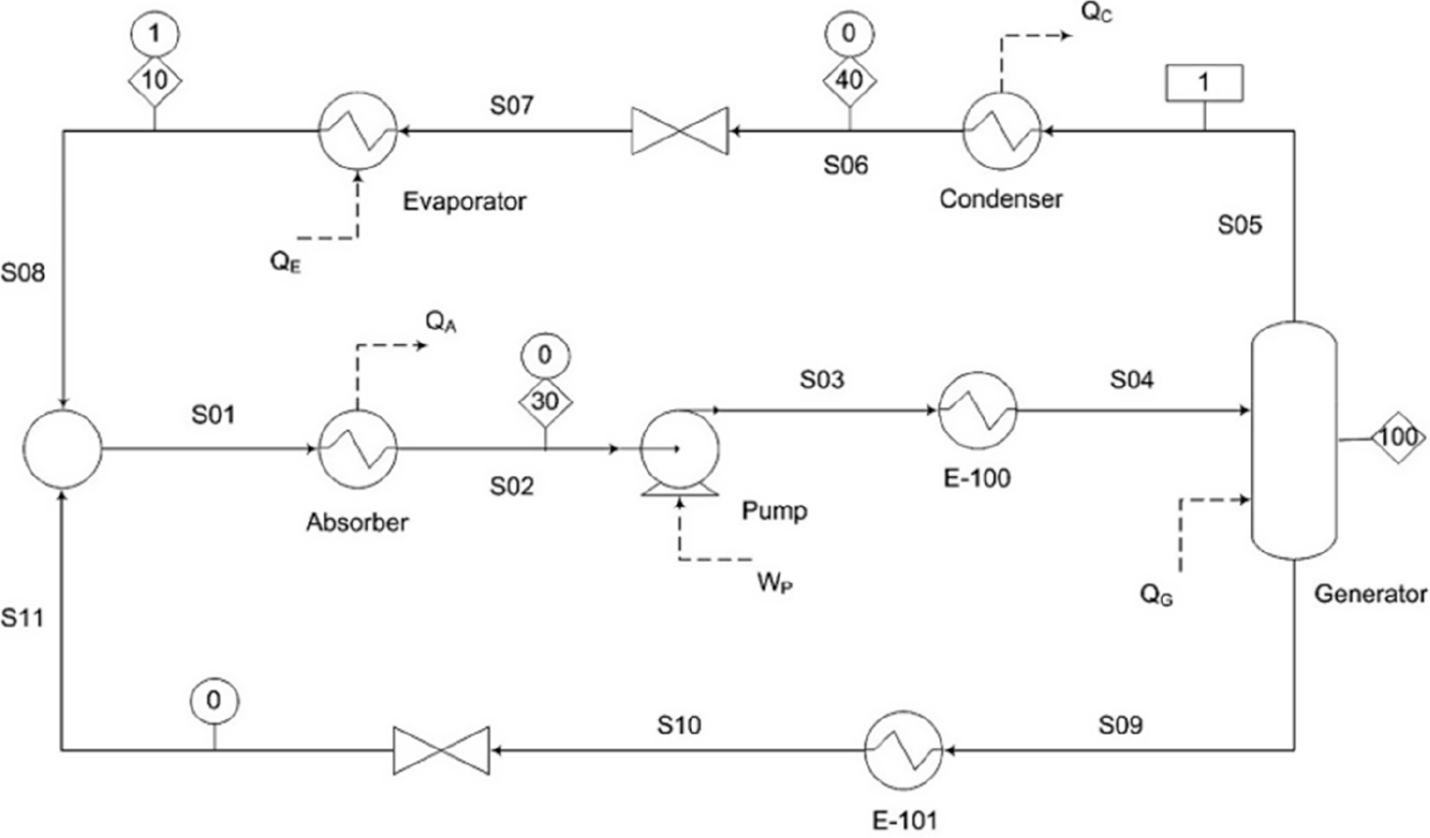
Process-Flow Diagram of Typical IL-Based Absorption/Refrigeration Cycle. Reproduced from Ref (69). Copyright 2014 Elsevier.

This section reviews the most important conclusions extracted from the literature referring to process simulation in absorption refrigeration cycles using ILs. Table 13 summarizes the studied process operating variables and KPIs of the studies presented in the literature. Additionally, process simulation can be used in more complex flowsheets and applications and is a useful tool for selecting the operating temperature and pressure ranges and the most favorable refrigerant/IL combinations depending on the requirements. The typical flowsheet of such processes mainly comprises an evaporator, absorber, generator, and condenser as principal equipment. The complete process flow diagram is shown in Scheme 11 and is based on a simple ideal cycle where the condenser, expansion valve, and evaporator comprise the refrigerant part of the circuit (lowest temperatures). The other part of the circuit comprises an absorber, a gas generator, a valve, and a pump and is typically known as the “solution section.” The generator temperature must be given (for instance low-quality vapor stream of 100 °C) and it hardly determines the refrigeration production, evaporator temperature (in this example chilled water of 10 °C). Therefore, the direct modulation of the temperature in generator implies the “cold production” as listed in Table 13 (check temperatures in the different parts of the circuit). Other different specifications must be considered when performing an absorption refrigeration cycle, such as the stream that comes from the condenser (S06) is the refrigerant-saturated liquid (vapor fraction = 0), and the stream that enters the absorber (S08) contains the refrigerant-saturated vapor (vapor fraction = 1). Regarding the operating total pressures in the cycle streams, the condenser and evaporator are those corresponding to refrigerant VLE pressures at the studied temperatures. Therefore, the degrees of freedom of the cycle are the compositions of the IL-poor (S01–S04) or -rich (S09–S11) solutions. Therefore, the refrigerant’s molar fraction determines the “operating window” of the cycle. The typical KPIs of absorption refrigeration cycles are the coefficient of performance (COP) and the solution circulation (f ratio). COP is defined as the division between the evaporator and generator heating duties that represent the efficiency of the cycle, while the f ratio is obtained by the mass-flow ratio between a weak IL solution and the refrigerant and enables the evaluation of the absorption operation. In the literature, various refrigerants (ammonia, water, R-134a, etc.) and ILs (imidazolium cations combined with chloride or NTf_2_ anions, etc.) were evaluated in the absorption-refrigeration-cycle application (see Table 13). For easily comparing the performance of the different refrigerant/IL pairs, the mass-cooling capacity (MCC) concept was included as the total mass flow pumped per unit of evaporator power. Process simulations can also provide insights into the operating window of refrigerants, such as the cooling capacity and mass concentrations in the absorber.

**Table 13:**
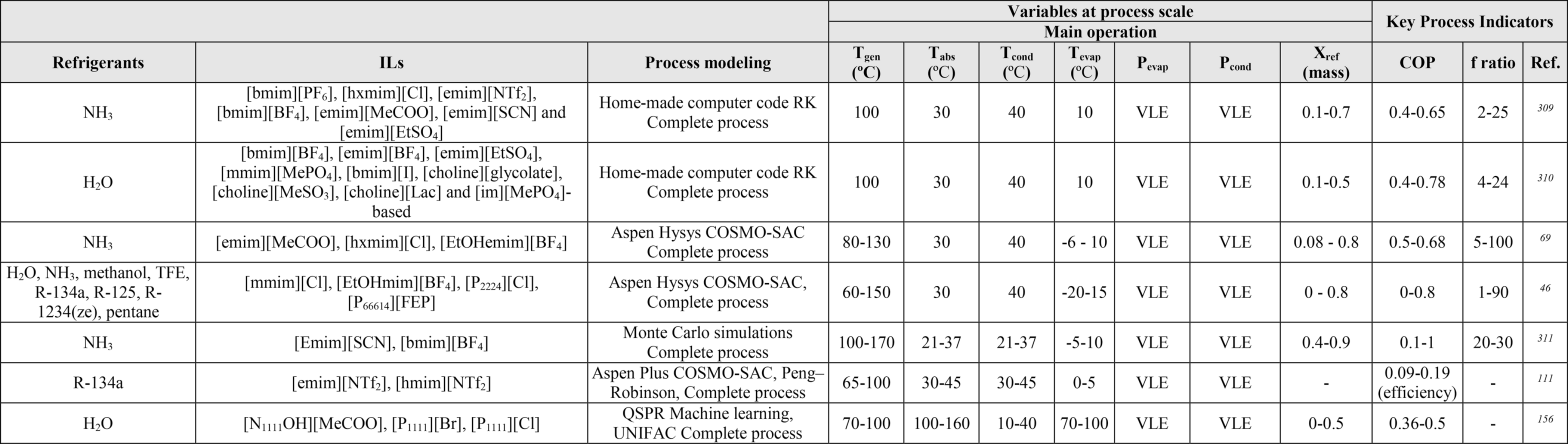
Summary of Variables at Process Scale and KPIs in IL-Based Absorption Refrigeration Cycles

**Figure 65:**
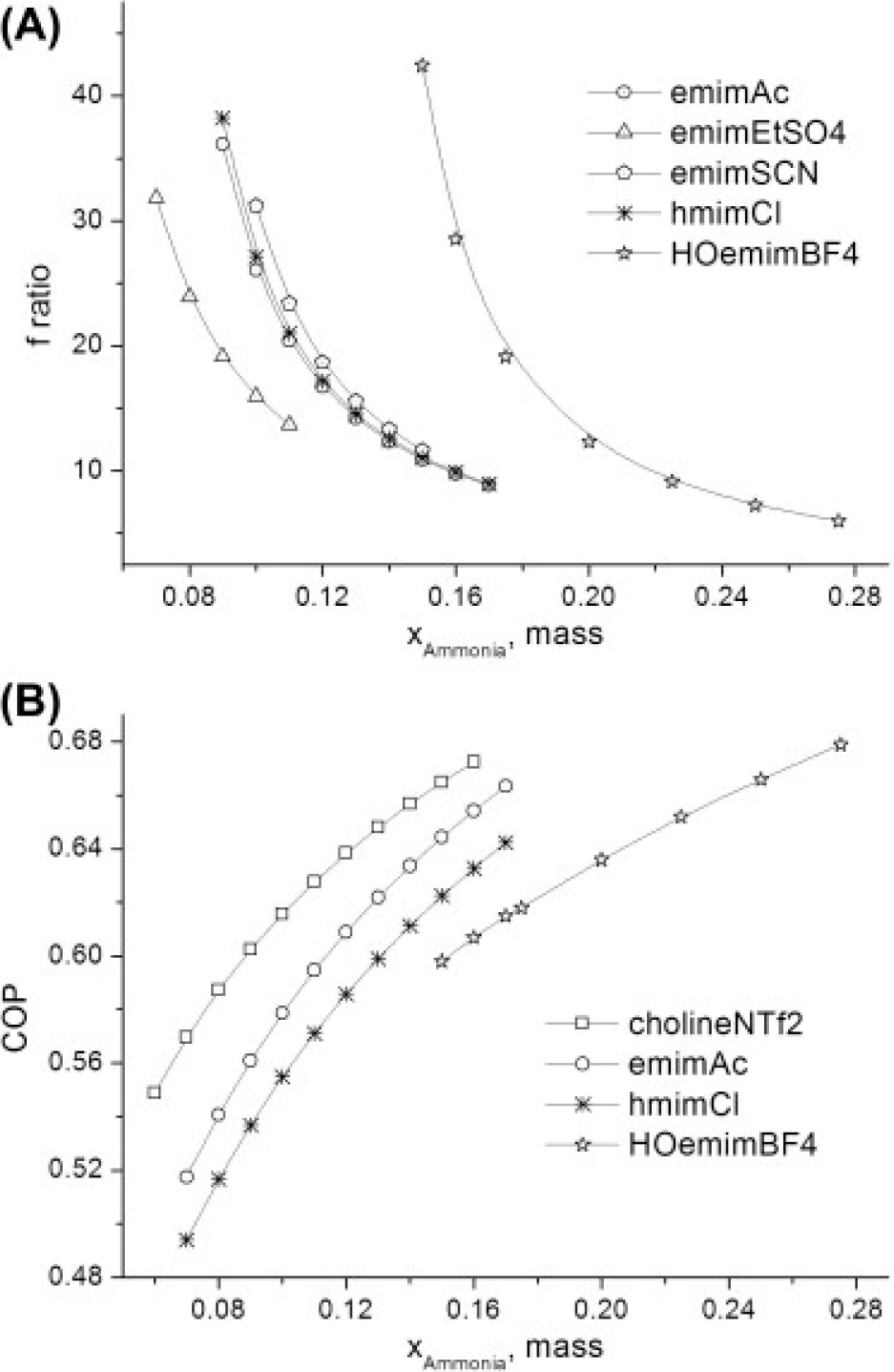
(A) f ratios and (B) COPs for ammonia refrigeration cycles using different ILs under conditions shown in Scheme 11. Reproduced from ref (69). Copyright 2014 Elsevier.

**Figure 66:**
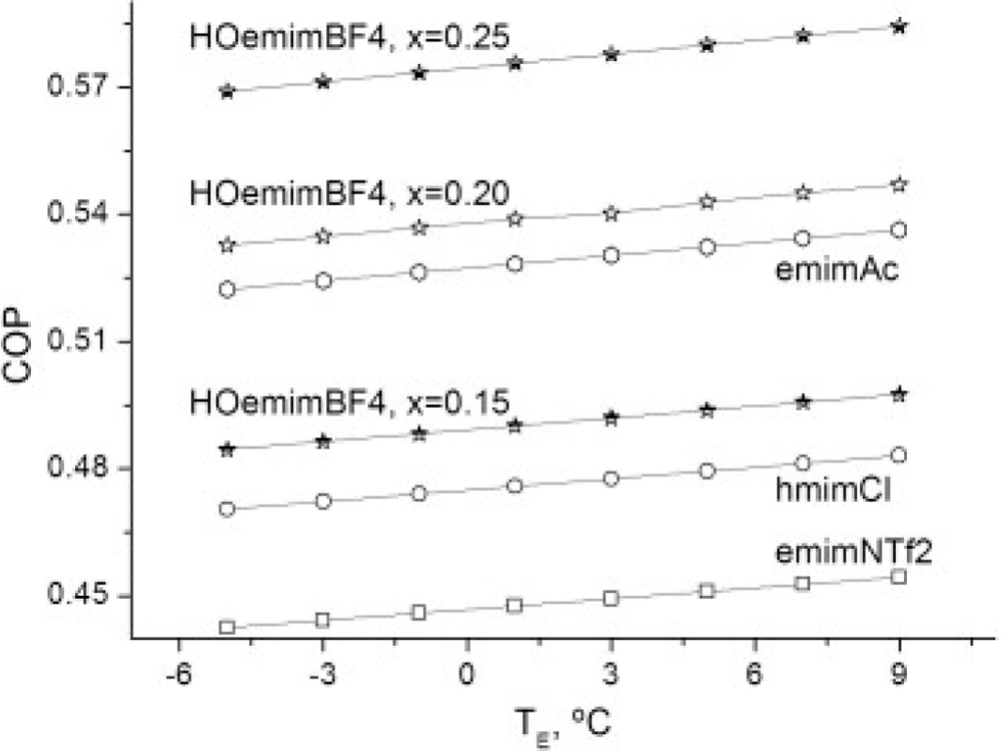
COP plotted as functions of temperature of refrigerated space obtained using different NH_3_/IL pairs and refrigerant concentrations under conditions shown in Scheme 11. Reproduced from ref (69). Copyright 2014 Elsevier.

In 2007, Yokozeki et al.^309^ initially reported the use of ILs in NH_3_ absorption refrigeration cycles. Ammonium acetate exhibited the best performance among those evaluated with a COP of 0.61 and an f ratio of 7.60. The chilled water temperature is produced (10 °C) using low-quality vapor in the generator (100 °C). However, compared with the NH_3_/water reference system, the proposed system had lower IL-based COPs and f ratios. Later, Ruiz et al.^69^ reproduced the same results as those in another study^309^ using the same NH_3_/IL pairs and used the COSMO/Aspen methodology to evaluate additional ILs. Different operating conditions, such as temperatures and NH_3_ concentrations, were evaluated to optimize the cycle performance. The authors demonstrated that the cycle performance could be optimized by changing the operating conditions. Thus, an increase in the NH_3_ (or refrigerant) concentration (weak IL solution) led to a higher COP and a reduced f ratio, which enhanced the cycle performance^46,69^ (see Figure 65). The enhancement of temperature generator led to lower cycle efficiencies (COPs) even though the f ratio decreased and, thus, the absorption operation was improved.^46,69,111,156,311^ Regarding “cold production,” Figure 66 shows COP plotted as functions of the evaporator temperature for different ILs and NH_3_ concentrations. Notably, although the four ILs could produce the same evaporator temperatures (up to −6 °C), they obtained different thermodynamic efficiencies and increased the COP while obtaining less “cold production” (up to 9 °C). Among the ILs evaluated in the NH_3_ refrigeration cycle, [(EtOH)mim][BF_4_] and [choline][NTf_2_] exhibited the best performance, with a COP of 0.668.^69^ Similarly, Moreno et al.^46^ reaffirmed [(EtOH)mim][BF_4_] as the best candidate, which even improved the water performance (0.65 as a reference). Wang et al.^311^ confirmed that BF_4_-based anions were the most suitable for NH_3_ refrigeration cycles, obtaining a COP of up to 1.1 for cooling at −5 °C in a double-effect cycle.

The use of other refrigerants (water, R-134a, pentane, etc.) in IL-based absorption refrigeration cycles has also been reported in the literature.^46,111,309^ Again, Yokozeki et al.^310^ initially published the use of ILs in water absorption refrigeration cycles compared with the traditional water/[Li][Br] system and found that some water/IL pairs may compete with the reference, with the [emim][MePO_4_] IL having the highest COP (0.691). The proper IL selection and optimization of the cycle’s operating variables enhanced the performance of the traditional water/[Li][Br] system. Thus, chloride based ILs reportedly enhanced the cycle’s COP^46^ because of the higher water absorption capacity compared with those of previously evaluated ILs and [Li][Br]. Ammonia- and water-refrigerant systems are the most extended industrial absorption refrigeration cycles. However, other refrigerants, such as R-134a, have also been evaluated using IL absorbents. For example, phosphonium chloride was reportedly the best candidate because of its high absorption capacity, reaching a COP of 0.4 under the optimized operating conditions.^46^ NTf_2_-based ILs have also been evaluated for an R-134a system under different conditions and compared with the traditional organic Rankine cycle (ORC) for cooling production.^111^ Without optimization, the ORC achieved better efficiencies than absorption refrigeration cycles using NTf_2_-based ILs. This was especially clear when operating with lower-grade heating sources. However, the system could be further optimized, and the cation and anion could be tuned, which may increase the cycle efficiencies.

**Figure 67:**
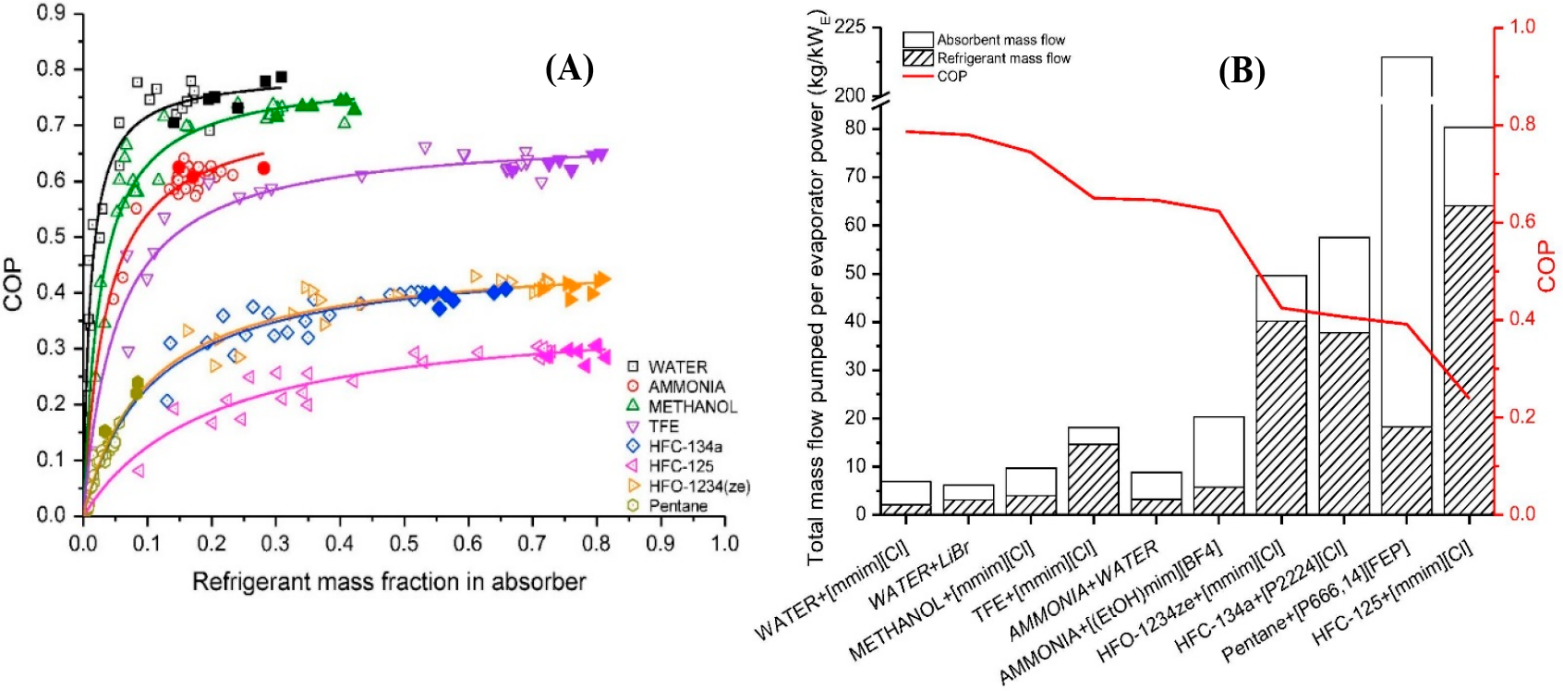
(A) COP plotted as functions of mass-fraction composition of absorber’s refrigerant for each refrigerant/IL pair. (B) Main efficiency parameters of absorption refrigeration cycles using different refrigerant/IL pairs for absorber, generator, condenser, and evaporator temperatures of 30 °C, 100 °C, 40 °C, and 10 °C, respectively. Reproduced from ref (46). Copyright 2018 Elsevier.

Very different refrigerant/IL pairs can be evaluated. The cycle’s operating-window conditions hardly depend on the refrigerant or the IL absorbent. Figure 67A shows the COP plotted as functions of the refrigerant concentration in the absorber for each refrigerant/IL pair (see the tremendous number of evaluated refrigerant/IL combinations).^46^ Notably, because the maximum COP was at the highest refrigerant concentrations, higher-solubility ILs would be the best for the task. In contrast, the operating window of each refrigerant was very different. Owing to the low MW of water, its operating window was quite narrow, while for R-134a or R-125, the operating window covered almost the entire range. Moreover, water, ammonia, and methanol all exhibited higher cycle efficiencies. The performance of the distinct pairs could be compared using MCC parameters. Figure 67B shows the MCC and COP values for the best IL candidates for each refrigerant.^46^ Clearly, water systems exhibited the best performance because they required the lowest refrigerant/IL mass flow and presented the highest COP and a relatively low f ratio. Methanol, TFE, and NH_3_ also constituted a high-performance group. Because typical hydrofluorocarbons, such as R-134a and R-125 systems, present lower COPs, they are inappropriate for application in absorption refrigeration cycles. However, these hydrofluorocarbons may be of interest in hybrid compression–absorption systems.

Li et al.^312^ used supercritical CO_2_ and NTf_2_-based ILs for vapor compression refrigeration cycles. The complete cycle was not modeled because only absorber and desorber units were simulated using Aspen Plus. Promising results require further evaluation of CO_2_/IL systems for refrigeration applications. The use of machine learning has also been proposed for optimizing the IL candidate in absorption heat transformers.^156^ Compared with cations, anions influenced the cycle performance more substantially. By optimizing the cycle operating conditions, the acetate-based IL reached a higher COP than the [Li][Br] system. This methodology could help to anticipate and propose the best ILs for different refrigerants in absorption refrigeration cycles.

Overall, ILs may present a competitive performance compared with those of traditional water/[Li][Br] and ammonia/water systems in terms of COP and MCC, even though the f ratios were higher (greater mass-flow requirements). Nevertheless, the use of ILs could avoid the potential technical problems that these systems present, such as crystallization or absorbent losses. Overall, the studies related to IL-based process simulations suggested that the cycle performance increased for ILs presenting higher refrigerant absorption capacities. Moreover, depending on the cooling demands, the selection of the refrigerant was crucial because, for instance, water cannot operate below its triple point (0 °C). Temperatures could be cooled below 0 °C using methanol or TFE, for instance. Process simulations enable the easy and rapid calculation of the refrigerant’s operating window independent of the IL absorbent (in terms of the cooling capacity, refrigerant concentration in the absorber, and generator temperature). To the best of our knowledge, no studies have reported the performance of ILs in absorption refrigeration cycles to date, including rate-based calculations, which would be very useful because the process could be preliminarily designed. However, ILs are currently used as operating fluids in the absorption refrigeration cycle at a pilot plant by Evonik (under a BASF license). New working pairs based on ILs and water or alcohols show less corrosion, lower toxicity, lower flammability, and no crystallization compared to conventional working pairs.^121^ Future studies on IL-based absorption refrigeration cycles must include an economic assessment and LCA to identify whether ILs may be competitive not only technically but also economically and environmentally.

### 4.8 Biomass Pretreatment

**Scheme 12:**
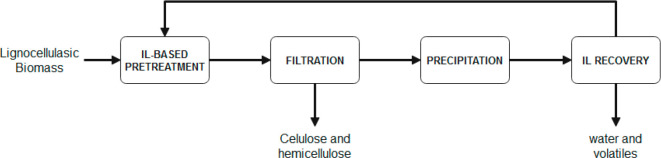
Usual Block Diagram for IL-Based Biomass Pretreatment, Including IL Recovery

One of the possible applications of ILs in the biorefinery field is the pretreatment of lignocellulosic biomass. In biorefinery processes, pretreatment is a necessary step to increase the digestibility of lignin, cellulose, and hemicellulose. Pretreatment also facilitates the fractionation of chemical bonds, as these biopolymers are inherently challenging to treat chemically and digest enzymatically.^313^ Among the various possible chemical or physicochemical pretreatments, in addition to IL-based pretreatments—also called ionoSolv—are those based on dilute acids, ammonia-fiber expansion, lime, steam explosion, autohydrolysis, and organic solvents.^314^ Because ionoSolv is a relatively new and underexplored technology, the application of process simulations in this field may enable pretreatment conditions to be optimized from a global process perspective and enhance the development of other key areas of this technology. However, despite considerable experimental studies on IL-pretreated biomass,^315^ few studies have been conducted for computationally modeling this operation. Although no single tested process flow diagram has emerged as a universally adopted standard, most studies in this field follow a structure like the one presented in Scheme 12. However, slight variations and modifications may exist among individual studies.

#### 4.8.1 Pretreatment Technoeconomic Assessments

**Figure 68:**
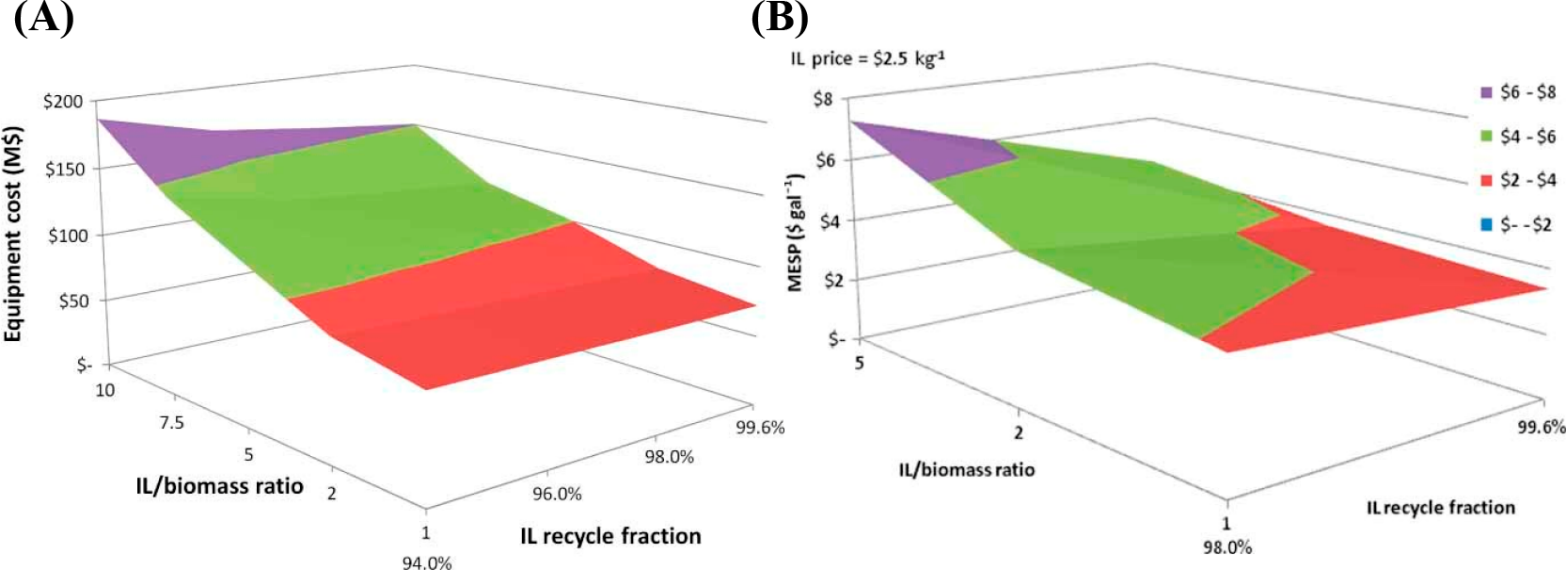
(A) Equipment cost plotted as functions of different IL loadings and recycling. (B) MESP for different IL/biomass loadings and IL recycling ratios at fixed IL price (2.50 $/kg). Pretreatment conducted at 120 °C and 1 bar for 30 min. Reproduced from ref (316). Copyright 2011 Wiley.

In 2011, Klein-Marcuschamer et al.^316^ conducted the first technoeconomic evaluation of a bioethanol production process that included an ionoSolv pretreatment. The model not only incorporated the block diagram presented in Scheme 12 but also included preceding and subsequent operations, such as biomass transportation and handling, enzymatic hydrolysis, fermentation, and product separations. This model was based on a previous one, developed by the same authors, that involved pretreatment with a diluted acid.^317^ To conduct this modeling, the authors selected the operating conditions and corresponding results based on the results of previous experiments,^316^ as listed in Table 14. By keeping all the process conditions constant, the authors studied the influences of the IL price, IL mass:biomass loading, and recycling ratio on the equipment costs and minimum selling price of ethanol (MESP).^316^ As expected, the authors found that the IL recycling ratio did affect the equipment cost, in contrast to the IL load, which did not influence the said cost, because a higher load implies larger equipment, as shown in Figure 68A. In addition, as shown in Figure 68B, for a specified IL price, MESP increases with either low recycling or high IL loading. However, at higher recycle rates or lower IL loadings, the effects are not as noticeable. These results suggest that in these processes, decreasing the IL loading is a more important factor than increasing the IL recycle rate because the IL loading affects the equipment size (as already anticipated), raw material costs, and utility costs of the process owing to the electricity consumed for the stirring and pumping associated with the increased viscosity owing to the higher IL content. Finally, the authors stated that the most crucial factor for improving the competitiveness of this process was reducing the IL price.^316^ Other developments would have minimal impacts without a reduction in IL costs because the high cost of the IL raw material itself accounts for a high proportion of the operating costs (∼25%).

**Table 14:**
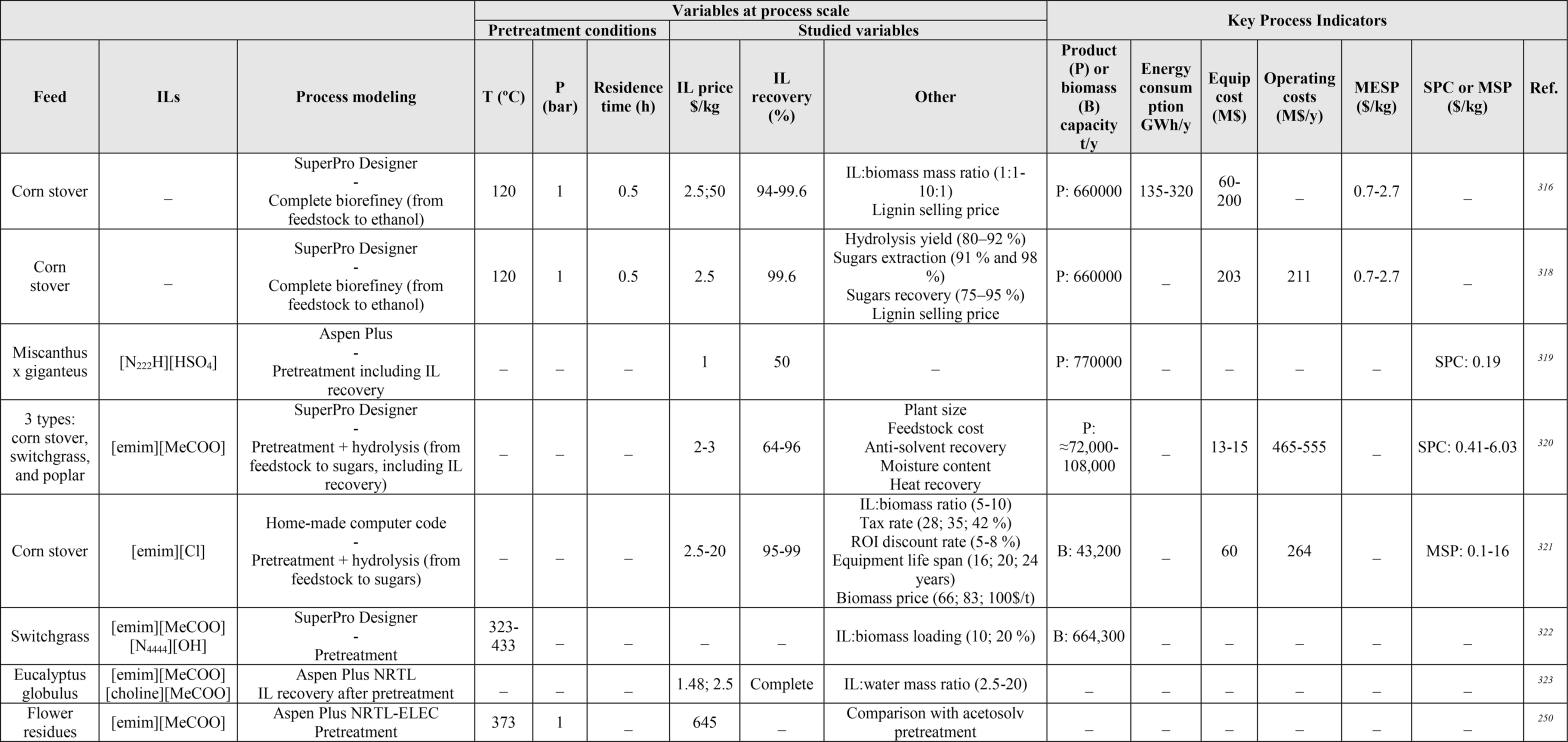
Main Process Indicators of Different Simulation Studies on IL-Pretreated Biomass^a^

a Unknown data were estimated assuming an operating time of 8,000 h/year. SPC and MSP refer to sugar production costs and minimum selling price, respectively.

**Figure 69:**
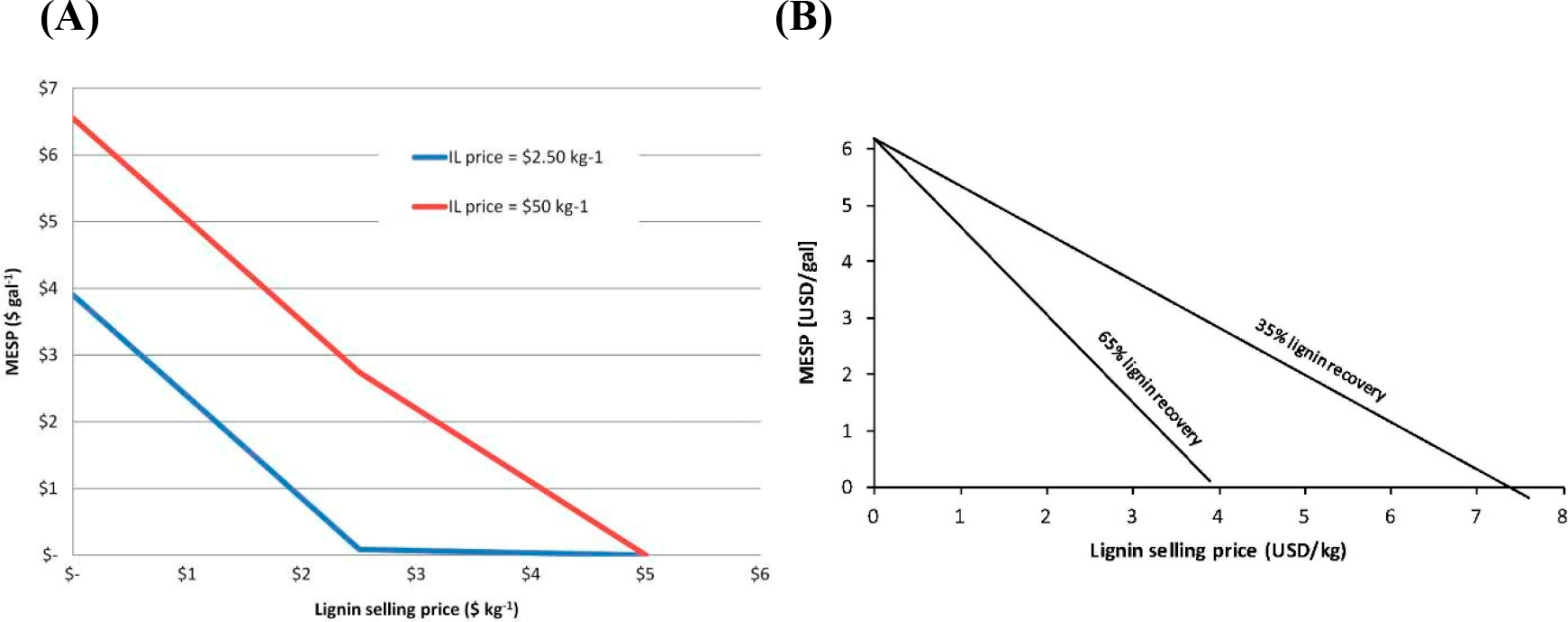
(A) Effect of lignin selling price on MESP at 1:1 IL/biomass ratio and IL recycle rate of 99.6%; pretreatment was conducted at 120 °C and 1 bar for 30 min. (B) Effects of lignin selling price on MESP at different lignin recovery rates, 2.5 $/kg IL price, 92% hydrolysis yield, 98% sugar extraction efficiency, 95% sugar recovery rate, and 99.5% organic phase recycling. Reproduced from refs (316, 318). Copyright 2011, 2016 Wiley, Elsevier.

Furthermore, to overcome the high IL cost, the authors proposed obtaining revenue from the remaining lignin.^316^ The authors analyzed the sensitivity of the influence of different lignin selling prices on the MESP and found that the MESP could be fully modulated, even decreasing to 0 at the highest IL price (50 $/kg), at an IL recycling ratio of 99.6% and an IL:biomass ratio of 1. Nevertheless, under these conditions, to achieve an MESP of 0, the required lignin selling price was excessively expensive (5 $/kg), while at a lower IL price, the lignin selling price decreased, as shown in Figure 69A. In a subsequent version of this model,^318^ the authors studied 35 and 65% lignin recovery scenarios in a three-phase separator and found that matching the ethanol market price required lignin selling prices of 4.87 and 2.62 $/kg at the lowest and highest recoveries.^318^ Therefore, the authors concluded that recoveries of at least 60% were necessary for both ethanol and lignin to be sellable products and the process to be economically feasible, as shown in Figure 69B, which once again highlights the importance of reducing IL prices and emphasizes the value of lignin as a byproduct that can influence the profitability of these processes. The findings of other authors reinforce the importance of lignin and hemicellulose valorization (in addition to heat recovery) for the process profitability.^319^

A subsequent process simulation study identified these same variables (IL price and recovery) as the key to the feasibility of these processes.^320^ Biomass preparation and pretreatment were modeled using [emim][MeCOO] and enzymatic hydrolysis to obtain fermentable sugars. Again, the pretreatment operating conditions were obtained from previous experimental studies. Moreover, the authors evaluated different feedstocks, analyzed the sensitivities of other variables, and concluded that for the technology to be competitive with sulfuric acid-based pretreatments, at least 97% of the IL must be recovered and that the IL price must be lower than 1 $/kg.^320^ In addition, the authors found that the heat recovery was the most critical factor that affected the capital investment and substantially affected the pretreatment operating time and number of reactors. Thus, the authors stated that a 90% heat recovery was necessary for these processes to be economically competitive. Furthermore, the analysis concluded that the ionoSolv pretreatment was economically feasible for all three evaluated feedstocks. In the base case—at an 80% IL recovery, 2.5 $/kg IL price, and 90% heat recovery—the sugar production cost was between 2.73 and 3.24 $/kg, depending on the feedstock.^320^ Finally, by optimizing these and other less-sensitive parameters, such as the plant size, moisture content, and antisolvent recovery, the sugar production cost was reduced to 0.4 $/kg_sugar_ for the three feedstocks. However, the cost for producing sugar through commercial sulfuric-acid pretreatment was only approximately two-thirds (0.26 $/kg) the cost of that for producing sugar through the proposed method.^324^ Furthermore, Brandt-Talbot et al. stated that using an inexpensive IL, such as ([N_222_H][HSO_4_]), could reduce operating costs by up to 30% compared with those for the dilute-acid pretreatment.^319^ In addition, the authors claimed that owing to the absence of pressure, the ionoSolv pretreatment presented lower capital costs than those of aqueous or organic pretreatments.

The authors of most studies have agreed that the high cost of ILs is the main barrier for implementing these processes.^316,320,321,325^ To overcome this disadvantage, process configurations that require lower IL consumption should be explored. For example, Sen et al. developed a process including initial dilute-acid and subsequent IL-based hydrolysis steps.^321^ The implementation would reduce the amount of IL required by 50% while still maintaining a high sugar yield because the IL was still used to hydrolyze the recalcitrant cellulose in the second step. In addition, the uncertainty regarding the acquisition cost of large-scale ILs was highlighted.

Parthasarathi et al. compared the performances of [emim][MeCOO] and [N_4444_][OH] at the process scale.^322^ The [N_4444_][OH]-based pretreatment could be conducted at lower temperatures than [emim][MeCOO], which prompted an analysis of its process-scale impact on energy consumption. The same process was modeled for both ILs at biomass loadings of 10 and 20% during pretreatments at 50 and 160 °C for [N_4444_][OH] and [emim][MeCOO], respectively. For both biomass loadings, the energy requirements at 50 °C were considerably lower—by approximately 75%—than those at 160 °C.^322^ Moreover, compared with [emim][MeCOO], [N_4444_][OH] had energy requirements that were less affected by the change in the biomass load, which rendered [N_4444_][OH] much more flexible than [emim][MeCOO]. The applicability of process simulations for the energy-term-based selection of ILs has also been demonstrated.

#### 4.8.2 IL Recovery

**Figure 70:**
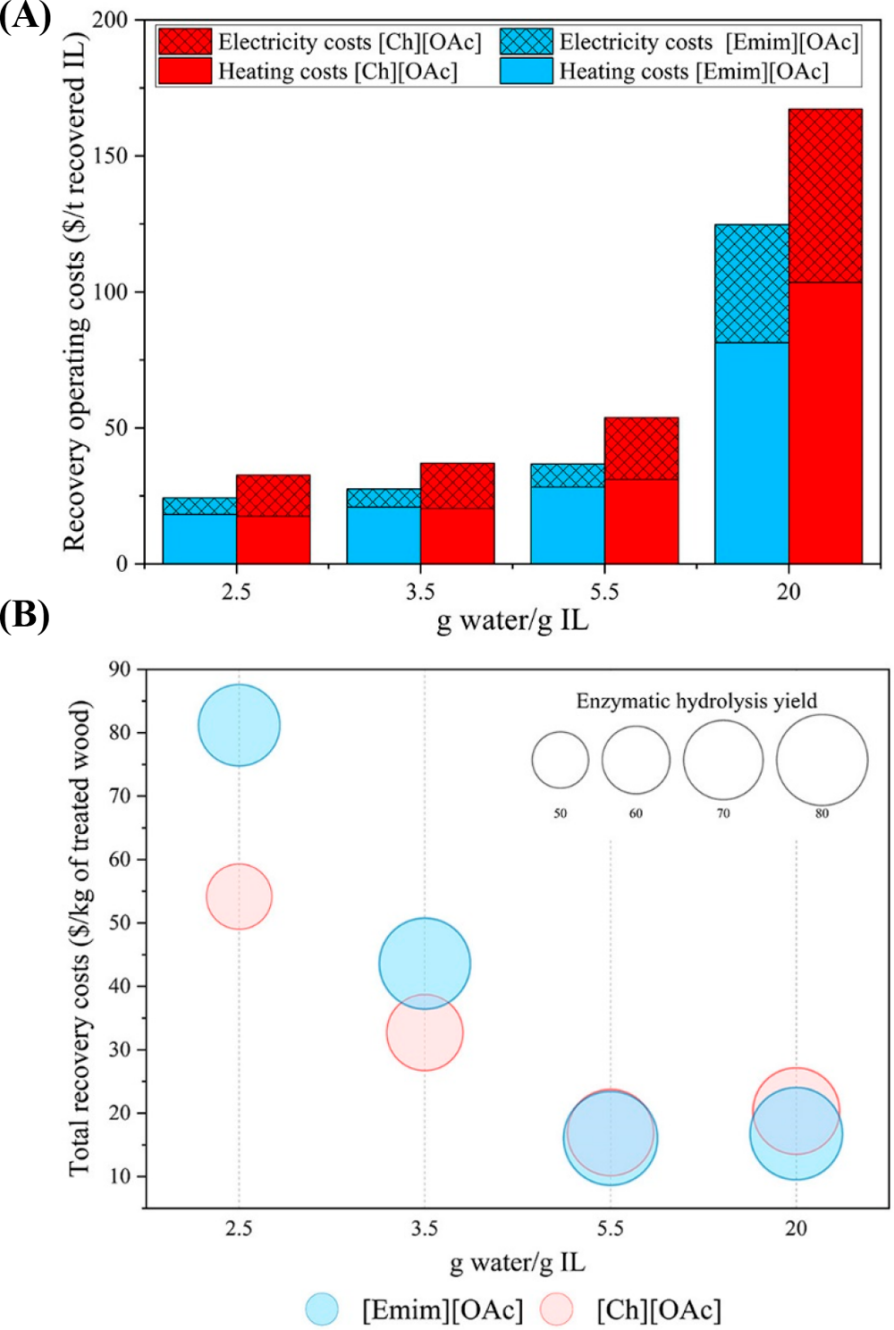
(A) Operating costs per tonne of recovered IL and (B) total recovery costs estimated for different ILs and water:IL loadings. Reproduced from ref (323). Copyright 2021 ACS. This evaluation was conducted using flash unit operating under optimized conditions, namely at 150 °C and 0.7 bar for [emim][MeCOO] and 160 °C and 0.5 bar for [choline][MeCOO].

[emim][MeCOO] has been one of the most studied ILs at the process scale.^250,320,322,323^ Nevertheless, it has been demonstrated that this cation and anion combination inhibits the growth of certain ethanol-producing yeasts due to toxicity; thus, its concentration must be reduced in biomass prior to fermentation.^326^ The use of water has been suggested for washing biomass because this lowered residual IL levels and did not inhibit yeast growth. Thus, Ovejero-Pérez et al.^323^ evaluated the influence of this water-based IL recovery from the pretreatment stream on the enzymatic hydrolysis and IL recovery costs of acetate-based [emim][MeCOO] and [choline][MeCOO] ILs. To optimize the simulation of this phase separation, the authors first gathered vapor–liquid equilibrium experimental data for recovered IL/water mixtures. As expected, when more water was used for washing the biomass, the pulp contained less IL. As a result more glucose was released during enzymatic hydrolysis.^323^ However, this increased use of water also increased the operating costs for the IL recovery. As shown in Figure 70A, increasing the water amount during washing substantially increased the heating and electricity costs. For example, for [choline][MeCOO], by increasing the water:IL mass ratio from 2.5 to 20, the heating cost rose from approximately $20 to $100 per tonne of recovered IL.^323^ Therefore, water:IL loads of 2.5 or 3 w/w may be the most appropriate for this process. However, the results in Figure 70B—including the effect of the water amount on the subsequent enzymatic hydrolysis stage—show that minimizing the water amount did not reduce the costs because low water amounts resulted in a low IL recovery, which implied a low enzymatic hydrolysis yield, and increased the cost per kilogram of treated biomass. High water loadings, on the other hand, implied higher operating costs than required for complete IL recovery.

#### 4.8.3 Sustainability Assessments

Hardly any studies are available on environmental evaluations. Vásquez et al.^250^ compared the ionoSolv process—using [emim][MeCOO]—with the acetoSolv process and found that the acetoSolv-based process displayed substantially greater environmental impacts on global warming, acidification, photochemical smog, and aquatic toxicity. In addition, the authors evaluated the economic feasibility of the process and revealed that the [emim][MeCOO]-based process (44.79 US$/kg) generated approximately 45% more profit than the acetoSolv process (30.78 US$/kg), which was surprising because the employed IL was more expensive than those used in previous studies (Table 14). This is further evidence of the interest in IL-based biomass pretreatments and the importance for studying their economic feasibility.

#### 4.8.4 Outlook

Although ILs have shown promising results in biomass pretreatment at the laboratory scale, additional research is required to make their use economically feasible at the commercial scale. The results summarized herein suggest that process simulations play a crucial role in the development of efficient and sustainable IL-based biomass pretreatment and identifying key parameters for designing these processes. The possibility for comparing the economic feasibility of this technology with other pretreatment technologies and identifying areas for improvement is crucial for determining the potential of this technology for becoming a commercially competitive option for biomass pretreatment. There are a wide range of applications related to or near biomass pretreatment with ILs in which process simulation must contribute in the near future more actively, from ethanol production^316,327^ to furanic compounds,^328^ cellulose or hemicellulose production,^329−334^ keratin production,^335^ eugenol and phenolic products^336^ and biofuels,^337,338^ among others.^121,339,340^

Furthermore, process simulations facilitate IL selection based on economic and energetic terms prior to pilot-plant-scale studies. Therefore, further process-scale studies considering several operational parameters must be conducted to develop a cost-effective approach for utilizing ILs for pretreatments and, thus, facilitating industrial-scale adoption. According to this, there are abundant efforts to develop new criteria selecting ILs based on LCA externalities,^341^ price,^325^ or even produced *in situ* related to the application.^342^

In any case, however, there are relevant pilot plants running nowadays in the biomass pretreatment by means of ILs. Rogers and Hallett are very active in spin-off creation, exemplified in 525 Solutions,^121,343^ or Lixea,^344^ with relevant developments in biomass pretreatment with ionic liquids and running pilot plants; complementary to this, the Ioncell^345,346^ project has developed more than 15 demos to-date. This can be traduced not only as a success by itself of the approach but also an urgent call to elevate current standards using the power of process simulations coupled to economic and sustainable tools.

In current computational studies on ionoSolv pretreatments, economic evaluations are limited by inaccurate IL prices^316,318^ and predictive capabilities because these simulations extensively rely on experimental data. Thus, few computational studies are available for various biomass pretreatment conditions because these conditions must first be experimentally evaluated. Process simulations have highlighted the importance for reusing ILs; however, research efforts should focus on switching pretreatment steps with different solvents or blending different solvents to reduce IL consumption; evaluating additional regeneration technologies, such as pervaporation, adsorption, extraction, or membrane separation;^347^ and identifying methods for synthesizing low-cost ILs.

## 5 Current Limitations and Future Challenges

Regarding the evaluation of ILs at the process scale, the main limitations are the availability of recently developed ILs and lack of representative simulated IL-based systems. Therefore, the following research opportunities are clear:

- The number of ILs available in open-source databases should be increased for utilization in process simulations.
- Many more ILs should be designed and characterized based on molecular simulations to increase the availability of ILs for use with COSMO-based thermodynamic models.
- The knowledge of ILs should be increased by experimental characterizations or computational predictions (COSMO-RS, MD, QSPR, machine learning, etc.) for more accurately describing new and functionalized ILs to improve the goodness of the prediction for GC methods or provide ad hoc regression parametrization for system–IL pairs.
- The development of accurate regressive classical thermodynamic models (UNIQUAC, NRTL, etc.), defined from experimental data, is a crucial step to increase the technology readiness level (from conceptual design to protype or pilot plant validation) of IL-based process applications.
- The viscosity, maximum operating temperatures, and melting points should be incorporated for ILs for rigorously describing the kinetic control, solvent stability, and liquid window, respectively. To this respect, the development of valuable data-driven QSPR models (using ML, AI, etc.) to accurately estimate these key IL properties is a current main challenge.
- The predicted properties that are relevant to the studied process design should be validated to ensure that the error in the process simulation data input is in the expected range for conceptually designing engineering processes.

For processes involving chemical reactions, few rigorous models that incorporate IL catalysts are available, which is relevant to the following points:

- The knowledge of the reaction enthalpy and equilibrium constants at different temperatures should be improved from both the experimental and theoretical approaches for rigorously evaluating the energy balance and reaction conversion in the reactor.
- The collection of kinetic data should be improved for implementation in process simulators instead of relying solely on experimental conversion. This will enable the evaluation of different reaction conditions at the process scale and facilitate reactor sizing.

At the process scale, the models used for describing the main operations and separation trains must align with rate-based calculations for adequately describing chemical and energy consumptions and process costs (CAPEX, OPEX, and TAC) for unfavorable fluid dynamics as follows:

- Rate-based modes should be simulated to evaluate the possible mass-transfer kinetic limitations for absorption and distillation (physical and reactive), including the separation train.
- The device type and internal configuration (packing or plates) should be systematically selected based on clear and well-accepted criteria, detailing the related decisions and results.
- User rate-based models should be developed for more accurately describing the sizing of extractors and one-stage models that have not implemented rate-based calculations in the main process simulators.

Regarding specifications, one of the main limitations is the anarchy when fixing the purity or recovery standards or IL losses in the process. Therefore, the following points should be considered:

- The sensitivity analysis of common operating variables (temperature, pressure, feed flows, etc.) should be analyzed for conveniently designing operations and sizing equipment.
- For individual operation units, the sensitivity analysis specifying the separation (recovery and/or purity) or conversion should be analyzed using different ILs to determine their role(s) in the operation performance.
- For all the simulated processes, the mass-based commercial purity should be selected to provide comparable results for the scientific community.
- The mass balance should be expressed based on the mass because the IL MW can obscure the results.
- The IL mass balance (recycling stream’s mass flow and purity and makeup stream’s composition and mass flow) should be indicated by considering different scenarios where the IL is lost during the process’s life cycle.

In addition, some studies lack sufficient information to evaluate the specific-consumption-based energy consumption and determine whether the methodology was appropriate; hence, future studies must cover the following points:

- Fully and properly characterize utilities, namely heating, cooling, and electricity, by specifying the partial energy consumption based on the specific energy consumption (kJ/kg) and always provide the details of the global mass and energy balance.
- Specify the cost, input, and output specifications of the heating and cooling utilities and resulting mass flow for each to ensure fairness when developing processes. Importantly, realistic utilities should be used to provide comparable energy profiles.
- To obtain reliable energy duties for IL-based processes, close complete processes, including the main operation and regeneration units and recycling and conditioning operations, should be used.
- Vacuum requirements should be included in the IL regeneration stages and a robust modeling approach should be developed for these unit operations so that the energy consumption, OPEX, and CAPEX computations are reliable.

Regarding CAPEX, OPEX, and TAC, the main limitations are the process capacity and reference values of the current technology; thus, for future research studies, the following points should be considered:

- The OPEX contributions should be specified by accurately showing the utilities, raw materials, and labor costs (when applicable) in addition to the methodology that was used.
- To consider possible limitations or boundaries of estimates, the estimated CAPEX should be provided with the cost and sizing for each piece of equipment.
- The CAPEX, OPEX, and TAC should be expressed in thousands of dollars per kilogram (k$/kg) of the recovered or produced compound.
- Attending to growing IL market, reasonable IL prices to be considered for process cost estimations are those currently provided by suppliers for scaled IL production, in the 15–30 $/kg range.
- Evaluating the potential use of different renewable energy sources in IL-based process may contribute to develop more sustainable new IL applications.

Regarding the analysis of the environmental impacts of IL-based processes, multiscale research must be conducted and include sustainability criteria through LCA methodologies rather than using toxicity data and solvent-to-feed ratios to determine the process’s sustainability; therefore, the following points are proposed:

- Bridge processes should be rigorously simulated based on LCAs to compute other environmental impacts in addition to equivalent CO_2_ emissions, which are referred to as the GWP.
- In LCA databanks, the IL inventory should be expanded through computational and/or experimental approaches.
- In a “cradle-to-gate” approach, the system boundaries should be defined for specifically analyzing the environmental impacts of IL synthesis and use and IL-based processes.

To facilitate the comparison between IL-based processes and benchmark industrial technologies and evaluate the competitiveness and sustainability of IL-based processes, the following points should be considered:

- For process specifications, commercial processes should be evaluated under comparable operating conditions, and the technoeconomic and environmental impact data should be made available for reliable comparisons.
- The effects of economies of scale on IL-based processes should be analyzed by performing simulations with increasing feed flows, evolving from the pilot to the industrial-plant scale.
- Processes should be evaluated based on multiple criteria, including the specific energy consumption, specific TAC, and main environmental impacts.
- Processes should be redesigned based on sustainable criteria to either reinforce or revise the IL selection criteria.
- Multiscale IL-product and IL-based process design optimization is key for developing cost-effective technologies. Robust COSMO-based or UNIFAC-based/Aspen methodologies are affordable and validated approaches for general users to perform process simulation studies on ILs.
- Efficient optimization methods, as pseudotransient modeling approach, are required when using complex IL-based process flowsheet models (multiscale, nonlinear, high-dimensional, dependent on IL structure, including thermodynamics and kinetics).
- Surrogate QSPR models, based on machine learning techniques, are promising alternative to thermodynamic models to predict the IL-based system properties in simultaneous product and process design optimizations.
- As key factor for scaling up and operating new technologies, process control of IL-based plants needs to be developed. Adequate dynamic simulations and experimental validations by testing IL-based system in industrial control equipment are required steps for this purpose.
- Dynamic simulations must emerge to computationally validate optimization criteria by studying control stability and operational behavior of the processes, rationally controlling hold ups and operating variables.

Usually, the connection between experimental findings and process simulation conclusions must be improved for the IL research community. Apart from research groups employing multiscale methodologies, future experimental studies must be aligned with main conclusions from process simulations to avoid the loss of time and resources by characterizing systems that are irrelevant to technological development. In addition, current laboratory research must evolve from focusing on experimentally characterizing the properties of pure IL compounds and IL mixtures to validating their operation performance, stability and compatibility in pilot plants or prototypes of the IL-based technology.

Noticeable and growing number of commercialized and pilot plant IL-based applications (>57), involving their use as activating solvents, catalysts, additives, electrolytes, lubricants, operating fluids, and solvents. These IL-based technologies have proven competitive within technical effectiveness, robustness, life cycle, safety, and environmental impact. The experience gathered in industry should enable a more open approach to academy, contributing to the cross-technological breakthrough of mass applications of ILs. In this respect, advanced commercialized processes for a sustainable industrial production of IL (Proionic currently reports an annual production capacity in the 700 t range) allow the economy of scale, thus making IL-based technologies more attractive to management and investors.

Through coordinated interdisciplinary studies conducted by experimental researchers and process engineers, the development of effective IL-based technologies should be accelerated for main current applications related industrial decarbonization, the circular economy, or residue valorization.

## 6 Outlook and Conclusions

In recent years, ILs have been increasingly evaluated using process simulations covering a wide range of applications and objectives. Therefore, in key industrial applications, various contributions have been driven by these nonvolatile solvents.

The first strategic and methodological contributions corresponded to the potential application of process simulations in the development of IL-based technologies and the challenge for effectively incorporating ILs into databases that were compatible with main commercial process simulators. Predictive models emerged as a more complex methodology for responding to this challenge by overcoming the limitations of classical thermodynamic models, namely the number of ILs, systematicity of calculations, and thermodynamic models of all the operation units of the entire process. According to the literature, most studies have been conducted using COSMO-based and UNIFAC models, which are state-of-the-art predictive models, to enable the use of ILs with commercial process simulators, such as Aspen Plus and Aspen Hysys, which are the most and second-most extended and used process simulators, respectively. Therefore, according to the literature, there are examples of IL databases that are compatible with several thermodynamic models are currently available for commercial process simulators, especially Aspen Plus. However, a representative number of ILs is required for effectively evaluating IL-based operations or processes and narrowing the range of potential ILs and operating conditions, and the target of the IL-based technology must be specified in response to existing or the development of industrial technologies, meaning that both the IL (product) and process design must respond to the IL features and process operating conditions. In addition to the advantages derived from the availability of ILs at the process scale, multiscale research opened the door to the ad hoc design of ILs functionalized to respond to process requirements, which changed the paradigm from the linear molecular-process simulation flow to a cyclic flow combining both iterative stages by feeding molecular simulations with process-simulation knowledge. Again, by extending this model to experimental efforts, another paradigm was developed, in which process simulations must be the leading stage to guide both molecular-simulation designs and experimental determinations.

These methodological advances enabled the effective screening of ILs at the process scale, as clearly shown in several fields, such as CO_2_ capture and conversion, liquid–liquid extraction, and extractive distillation. These massive screenings enabled the correlation of thermodynamic and kinetic properties, such as molality, distribution ratios and selectivity, and diffusion coefficients, with the behavior of the unit operation or entire process, depending on the problem definition. An approach based on databases listing thermodynamic and kinetic properties would help to evaluate the kinetic or thermodynamic control in the process. In fact, process simulations have revealed that IL-based CO_2_ capture is controlled by kinetics, indicating that highly viscous solvents are unsuitable for developing physical absorption technology. For capturing VOCs, the solute properties are the key because nonpolar solutes are thermodynamically controlled, whereas polar compounds exhibit increased resistance with increasing solvent viscosity. For some ILs, thermodynamics and kinetics both control the final solute recoveries. For dehydration processes, the proper selection of mixing rules between water and ILs is crucial because the system viscosity drastically changes when water is introduced. More complex applications, such as IL-based absorption refrigeration cycles, can also be modeled using process simulations by enabling the operating ranges (cold production) and most suitable IL/refrigerant pairs to be rapidly obtained for specific applications. Finally, extractive distillation offers greater viscosity flexibility during operation, as the operating temperatures are higher than those used in capture processes.

Regarding the solvent features and unit operation, the transition from the mesoscale to the process scale is easy, which facilitates a clear understanding of the substantial changes in solvent features at the process scale. IL-based chemical CO_2_ capture is drastically controlled by a narrow range of reaction enthalpies because these values minimize the energy consumption, whereas physical CO_2_ capture is controlled by the operation kinetics (viscosity). This process-simulation-based scientific knowledge can promote the design of IL-based carbon-capture processes with minimal solvent and energy consumptions and process costs. In addition, liquid–liquid extraction is driven by both the selectivity and distribution ratio, which are inversely coupled. For both extraction properties, low-efficiency purification and regeneration schemes require the selection of compensatory values, whereas efficient separation trains enable the use of high-capacity and low-selectivity solvents, thus minimizing the energy consumption. In fact, for different processes, process simulations rationalize IL properties, which enables the simultaneous design of the IL and separation train to emerge as the best strategy. Another representative example is extractive distillation because thermal stability can limit the application of ILs solvents at moderate operating temperatures, which excludes some potential ILs as feasible solvents. Studies on extractive distillation have revealed that the viscosity did not control the IL selection, operating conditions, or equipment design and could be combined with the IL selection at the process scale to enable the development of cost-effective solutions that consumed less energy.

Whether a process is energy intensive depends on the purity or recovery specifications, which can be assessed in the literature by comparing the parameters of a specific process to commercial standards or an arbitrary benchmark value. For instance, because the extraction and purification of aromatics to 99.9 wt % is not the same as fixing a tentative value of 95 wt %, the latter option will exhibit a falsely improved energy consumption compared to the former. In addition, purities and solvent-to-feed ratios must always be expressed by mass because, otherwise, the magnitude order cannot be properly compared. The dehydration of alcohols is a good example of this because in alcohol streams, the mass and molar purities are almost equivalent. However, the mass-based and molar solvent-to-feed ratios and water purities are substantially different.

Except for extractive distillation, energy consumption is related to the solvent separation train of the main operations, such as absorption or liquid–liquid extraction. Therefore, an entire process must be defined to provide useful energy-consumption information. In biorefinery or CO_2_ conversion, the subsequent operations following the biomass pretreatment or reaction must be thoroughly assessed, as the conditions of these two operations can substantially impact the subsequent steps. Furthermore, because the primary operations often account for only a fraction of the energy consumption, the design of downstream separation trains must be optimized. Additionally, the synthesis of impure compounds is futile, which emphasizes the requirement for efficient purification processes. Numerous papers have provided valid energy consumptions as benchmarks in IL technology compared to the available technology, such as amine-based CO_2_ capture, sulfolane, Morphylane, and other processes in which conventional organic solvents are used instead of ILs. Notably, all the utility costs and consumptions must be covered and properly computed, namely cooling, heating, and electricity, for which the latter is associated with not only the energy consumptions of pumping and compression but also vacuum expenses. As stated in the literature, the required vacuum expenses are inversely proportional to the maximum IL operating temperatures, which, implicitly, are the IL thermal stability limits. By neglecting to consider the vacuum consumption, the realistic heating consumption can be underestimated and the use of less-stable ILs can be promoted.

Although utility consumptions and costs are frequently used as IL selection criteria, process simulations are extensively used in biorefinery to evaluate the energy consumption and closed-cycle mass balance to help rationalize solvent and energy consumptions. All operations have representative selection criteria. In fact, although several authors have effectively fixed the equipment size or number of stages and operating variables for fairly comparing the energy consumptions of different processes, substantially different process descriptions have rendered the energy consumption or cost criteria as questionable. Regarding the decision between the consumption and cost, the research community has not yet established a clear criterion. Therefore, both factors should be monitored, and ILs should be selected based on specific study objectives, such as sustainable design, energy consumption, costs, and other relevant considerations.

However, the most extended approach for evaluating ILs, optimizing IL-based processes, and comparing IL-based and conventional technologies is the TAC or equivalent approaches. This option simultaneously computes operating costs (utilities and raw materials, when applicable) and investment expenses by fairly considering equipment sizing and energy and chemical demands. With respect to IL comparisons, several examples are available in the literature in which the TAC criteria of multiple ILs have been compared to select the most promising solvent. Another option that can be combined with the IL selection is the determination of the most favorable operating variables for a specific IL, thereby optimizing the process for that solvent, for which the commitment point is conditioned by the computational cost and optimization grade of the process. Finally, conventional, and current technologies, such as IL- and amine-based CO_2_-capture technologies or IL-based and Morphylane processes, have been rigorously compared to dearomatize refinery streams, and the technologies were competitive. In fact, the major limitation of IL-based processes is the IL price, which is frequently estimated for cost scenarios ranging from optimiztic to pessimistic. However, process simulations have enabled the comparison of solvent composition costs, which are important for solvent recycling, in positive scenarios for conventional solvents related to the nonvolatility of ILs (absorption and extractive distillation) and tunable solubility in outcome streams (as in liquid–liquid extraction operations).

In fact, for closed-cycle processes, process simulation contributions have been quite relevant and have changed the paradigm of IL selection. For instance, in CO_2_ conversion, process simulations have revealed that in closed processes, the design of efficient ionic ILs is as crucial as the conversion itself. To date, although almost all IL studies have been compared based on solvent-to-feed ratios or equivalent terms, IL make-ups and energy costs are the key process indicators that describe processes more accurately than the IL amount moving around in the process, leaving aside the process hold ups that, for long-term scenarios, are negligible.

Regarding sustainability criteria, most simulation studies report the equivalent CO_2_ emissions associated with the utilities supplied in the process. This consideration only covers global warming and neglects other impacts, such as human toxicity to humans, ecotoxicity, and terrestrial acidification. This is mainly attributed to the lack of IL production processes in popular LCA databases, such as Ecoinvent, which hinders rigorous LCA studies of IL-based processes when accounting for the environmental impacts of the amounts of IL solvents. Otherwise, the combination of LCA with process simulations is an efficient and useful method for generating LCA inventories for processes at the industrial scale and has demonstrably enabled robust technical, economic, and environmental assessments of IL-based chemical processes. Therefore, the generation of a database for synthesizing a wide range of ILs or incorporating ILs into existing databases would be an important advancement in the environmental-impact evaluation of IL-based processes.

Process-simulation-based strategic, methodological, and thematic advancements have decisively expanded the boundaries of IL knowledge at the process scale by highlighting the best practices, path forward, limitations of both past and present methods, and future challenges. Process simulations must guide the research community’s efforts for developing IL-based technologies to compete with current industrial standards or even innovative production routes for building a more sustainable future. The versatility of process simulations can help to not only provide multicriteria optimizations and analysis but also guide technological scalability by designing digital prototypes to help the design of pilot plants to exploit the digital twin concept. For process-simulation-driven ILs, the future, where scientific contributions and knowledge transfer align with industrial goals, is bright.
